# Synthesis of Indoloazepinone Scaffolds Using Sequential Photochemical and Photocatalytic Reactions

**DOI:** 10.1021/acs.orglett.5c02977

**Published:** 2025-08-25

**Authors:** Kate A. Ellis-Sawyer, Tomos Alderman, Kevin I. Booker-Milburn, Varinder K. Aggarwal, Adam Noble

**Affiliations:** School of Chemistry, 1980 University of Bristol, Cantock’s Close, Bristol, BS8 1TS, U.K.

## Abstract

Indoloazepinone scaffolds show promise as anticancer compounds; however, current methods for their synthesis rely on azepinone ring formation from prefunctionalized indoles. Herein, we report an alternative strategy for the rapid synthesis of indoloazepinones from dichloromaleimides and anilines using sequential photoinduced reactions, including a photochemical [5 + 2] cycloaddition and a photoredox-catalyzed dechlorinative indole formation. Construction of the indole core late in the synthesis allowed straightforward diversification of the benzenoid ring with a variety of functional groups.

## Graphical abstract

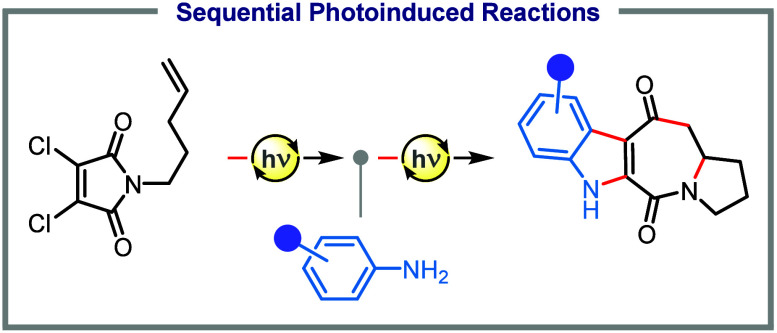

Indoloazepinones **1** are a subclass of fused azepinones that form the core structure of a range of bioactive molecules (Scheme 1a).^1^ For example, indole-analogues of the pyrroloazepinone natural product hymenialdisine were found to be potent protein kinase and cytokine inhibitors, therefore showing potential for the treatment of Alzheimer’s disease and cancer.^1–3^ Additionally, indoloazepinones have recently shown promise as crop protectants, due to their antiviral, fungicidal, and insecticidal activities.^4^

**Scheme 1:**
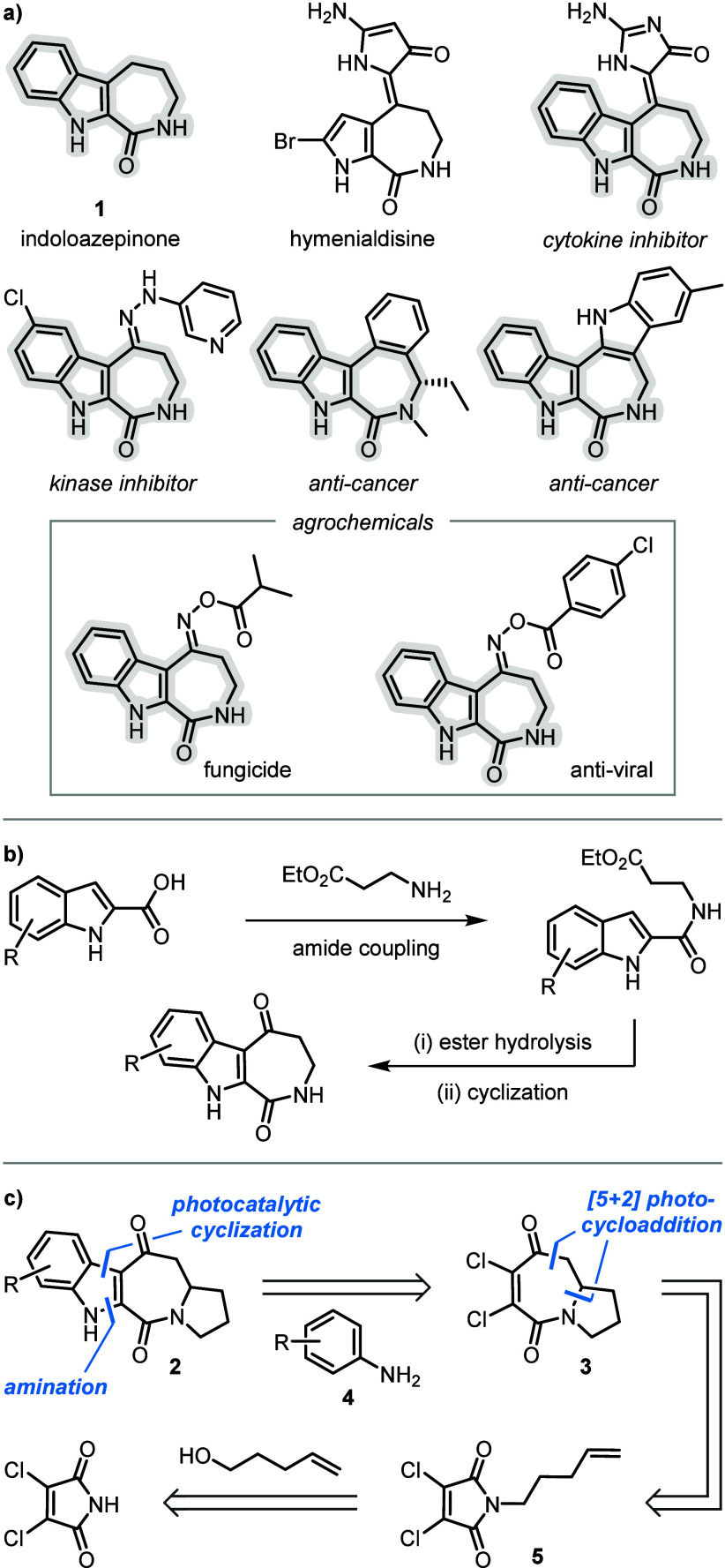
(a) Structures of Bioactive Fused Indoloazepinones; (b) Previous Synthesis; (c) Proposed Synthesis

Previous syntheses of indoloazepinone derivatives generally involve construction of the azepinone around an indole core.^5^ This utilizes amide coupling of indole-2-carboxylic acid with an ester derivative of β-alanine followed by hydrolysis and cyclodehydration to form an azepinedione ring (Scheme 1b). While this strategy allows facile synthesis of indoloazepinone analogues through diversification of the azepinone ring, the requirement for prefunctionalized indoles means that modifying the benzenoid ring of the indole is much more challenging. We considered that an alternative strategy involving late-stage construction of the indole core would represent a more versatile approach for the synthesis of diversely functionalized indoloazepinones. Specifically, we reasoned that the indole ring of indoloazepinedione **2** could be readily installed onto dichloro-azepinedione **3** in a two-step process involving amination with aniline **4** and photocatalytic dechlorinative cyclization onto the aromatic ring.^6,7^ Importantly, **3** is readily accessed by an intramolecular [5 + 2] photocycloaddition of dichloro-maleimide **5**,^8^ thus providing a concise synthesis of **2** via sequential photoinduced reactions of simple and readily accessible substrates.^9^ Herein, we report the successful realization of this strategy, wherein late-stage assembly of the indole core provides access to indoloazepinediones with readily modifiable substitution around the benzenoid ring.

Construction of the dichloro-azepinedione **3** was achieved in two steps from dichloro-maleimide **6** (Scheme 2). Alkylation of **6** through a Mitsunobu reaction with 4-penten-1-ol provided alkene-tethered maleimide **5**, which was transformed to **3** using the [5 + 2] photocycloaddition methodology developed by the Booker-Milburn group.^8a^ Subsequent regioselective nucleophilic substitution of the more electrophilic β-keto-chloride of **3** with aniline provided the aminated chloro-azepinedione **7a** in 85% yield.^10^

**Scheme 2:**
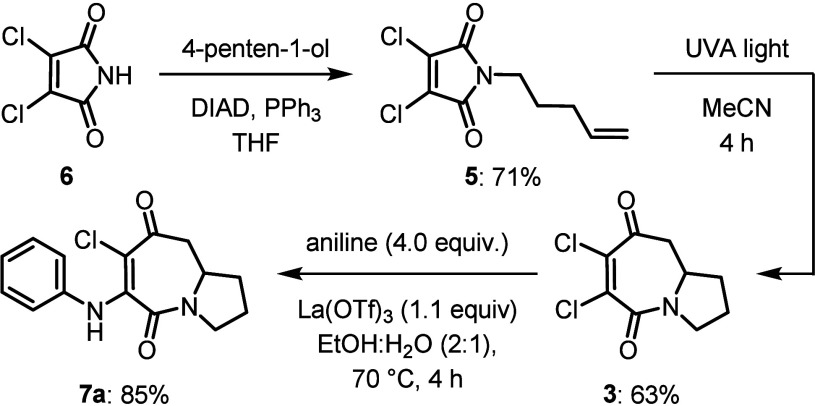
Synthesis of the Indoloazepinedione Precursor

With key intermediate **7a** in hand, we moved to the development of the photocatalytic cyclization to construct the indole ring (Table 1). Inspired by previous reports of photocatalytic oxidative indole formation from *N*-aryl enamines,^6b,6c^ we initially investigated cyclization of **7a** in DMSO-*d*_6_ using Ir­(ppy)_3_ as the photocatalyst. We were pleased to observe a 50% yield of **2a** after 24 h of irradiation with blue LEDs, along with 36% of unreacted starting material **7a** (entry 1). Addition of sodium acetate to neutralize the HCl byproduct resulted in the full consumption of **7a** and increased the yield of **2a** to 79% (entry 2). Further improvements were made upon reducing both the reaction time (entry 3) and catalyst loading (entry 4), providing **2a** in 89% yield. In addition, the concentration could be increased from 0.05 to 0.1 M without impacting the result (entry 5); however, further increasing the concentration to 0.2 M led to a minor decrease in the yield of **2a** (entry 6). Finally, control reactions showed that both the photocatalyst and light were essential for the reaction (entries 7–8).

**Table 1:**
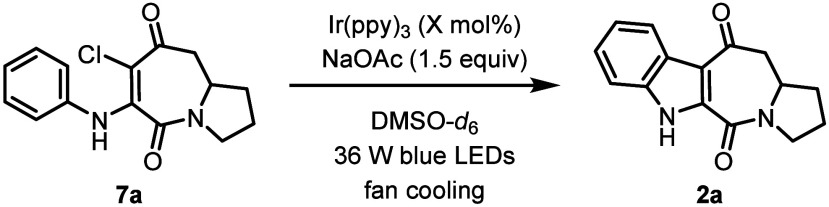
Optimization of the Indole Formation^a^

Entry	X mol %	Time (h)	[**7a**] (M)	% **2a** ^a^	% **7a** ^a^
1^b^	2.5	24	0.05	50	36
2	2.5	24	0.05	79	0
3	2.5	1	0.05	86	0
4	1.0	1	0.05	89	0
5	1.0	1	0.1	89 (85)^c^	0
6	1.0	1	0.2	80	0
7^d^	1.0	1	0.1	0	89
8	0	1	0.1	0	95

a Yields were determined by ^1^H NMR analysis using 1,3,5-trimethoxybenzene as an internal standard.

b Reaction performed without NaOAc.

c Yield of isolated product.

d Reaction carried out in the dark.

We then proceeded to investigate the scope of this indoloazepinedione synthesis by variation of the aniline used for the construction of aminated chloroazepinediones **7** (Scheme 3). It is important to note that we unexpectedly observed a small drop in yield of **2a** upon changing the solvent from DMSO-*d*_6_ to anhydrous, nondeuterated DMSO.^11^ Since this trend was also observed for several other substrates, we continued the investigations into the scope of the reaction using DMSO-*d*_6_ as the solvent. A variety of 5-substituted indole derivatives were successfully synthesized (**2a**–**2k**). The reaction was found to be relatively insensitive to the electronic effects of the substituent, with electron-donating (**2b**–**2c**, **2g**) and electron-withdrawing groups (**2h**–**2j**) tolerated. Products substituted with synthetically useful halides (**2d**–**2e**), boronic esters (**2f**), ketones (**2h**), carboxylate esters (**2i**), and nitriles (**2j**–**2k**), were formed in moderate to good yields (54–81%). While the *para*-methoxy substrate **7c** reacted successfully to form **2c**, the unprotected phenol derivative **7l** failed to cyclize, instead providing only a low yield of 37% of hydrodechlorination product **7l**′.

**Scheme 3:**
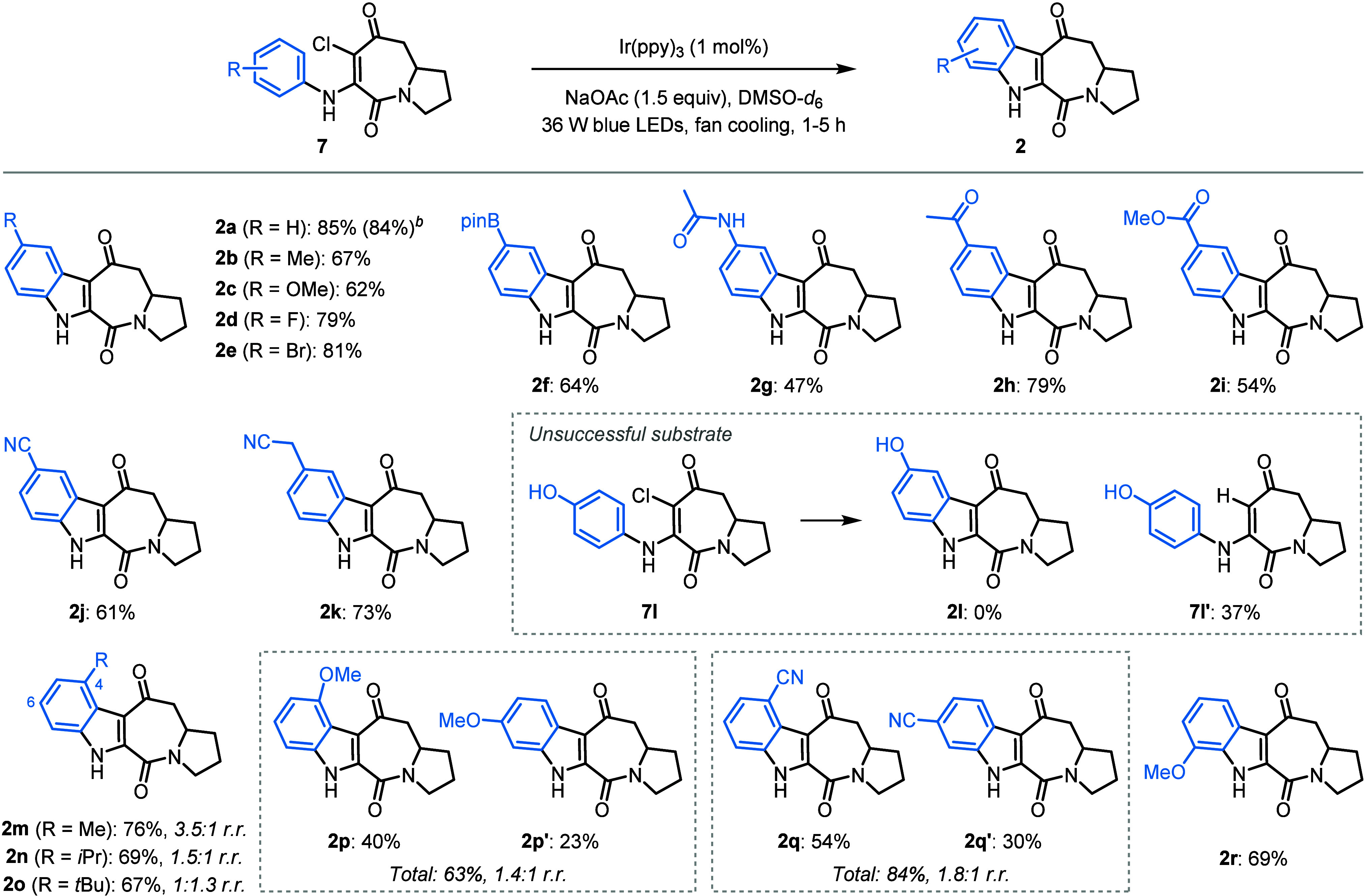
Substrate Scope^a^

a Yields are of isolated products. The regioisomeric ratios (r.r.) were determined by ^1^H NMR analysis.
b Reaction performed using 1 mmol of **7a**.

Having investigated the scope of the reaction for *para*-substituted anilines, we then turned our attention to substrates with *meta*-substitution. A mixture of 4- and 6-substituted indole derivatives (**2m**–**2q**) were obtained in good overall yields (63–84%) as a mixture of 4- and 6-substituted indole regioisomers. While the regioisomers of alkyl-substituted products **2m**–**2o** were found to be inseparable, it was possible to separate the regioisomers of the methoxy (**2p**/**2p′**) and nitrile (**2q**/**2q′**) derivatives. Generally, a small preference for the 4-substituted regioisomer was observed, except for the sterically demanding *tert*-butyl product **2o**, which displayed a slight preference for the formation of 6-substituted regioisomer **2o′**. These results indicate that sterics have a greater (see **2m** vs **2o**), albeit still relatively minor, impact on the regioselectivity compared to electronics (see **2p** vs **2q**). Finally, 7-methoxy-substituted indole **2r** was formed in a 68% yield.

Next, we investigated the mechanism of the indole forming reaction from chloro-azepinediones **7**. Previous reports of indole synthesis via photoinduced cyclization of *N*-aryl enamines have been proposed to proceed through both single electron transfer (SET),^6b,6c^ energy transfer,^6d^ and direct photoexcitation pathways.^12^ While the essential role of the photocatalyst rules out a direct photoexcitation pathway, activation of **7** by excited state Ir­(ppy)_3_ could occur by both single electron reduction or energy transfer.^13^ The feasibility of these two pathways was supported by comparison of the reduction potentials and triplet energies of **7a** and Ir­(ppy)_3_, which show the excited state photocatalyst is a strong enough reductant (*E*_1/2_ [Ir^IV^/*Ir^III^] = −1.7 V and *E*_p_ [**7a**/**7a** ^**•–**^] = −1.1 V vs SCE in MeCN)^14^ and has a high enough triplet energy (*E*_T_ = 243 kJ/mol for Ir­(ppy)_3_ and 236 kJ/mol for **7a**)^13^ for SET and energy transfer, respectively. Evidence for the SET mechanism was provided by the successful formation of **2a** in 73% yield when the photocatalyst was changed from Ir­(ppy)_3_ to eosin Y (**EY**), which is a strong enough excited state reductant (*E*_1/2_ [**EY** ^+•^/*EY] = −1.1 V vs SCE in MeCN/H_2_O), but has a significantly lower triplet energy (*E*_T_ = 182 kJ/mol) than **7a** (Scheme 4a).^15^ This suggests that the reaction likely proceeds through a SET pathway;^16,17^ however, we cannot rule out the possibility of an energy transfer pathway also operating when Ir­(ppy)_3_ is used as the photocatalyst.^18,19^ Our proposed mechanism for the SET pathway involves single electron reduction of chloroazepinedione **7a** by photoexcited Ir­(ppy)_3_ to form radical anion **8** and the reduced photocatalyst (Ir^IV^) (Scheme 4b). Elimination of a chloride anion from **8** generates vinylic radical **9**, which cyclizes onto the phenyl ring to give cyclohexadienyl radical **10**. Finally, the oxidation of **10** to cation **11** by Ir^IV^ and deprotonation produces indole **2a**.

**Scheme 4:**
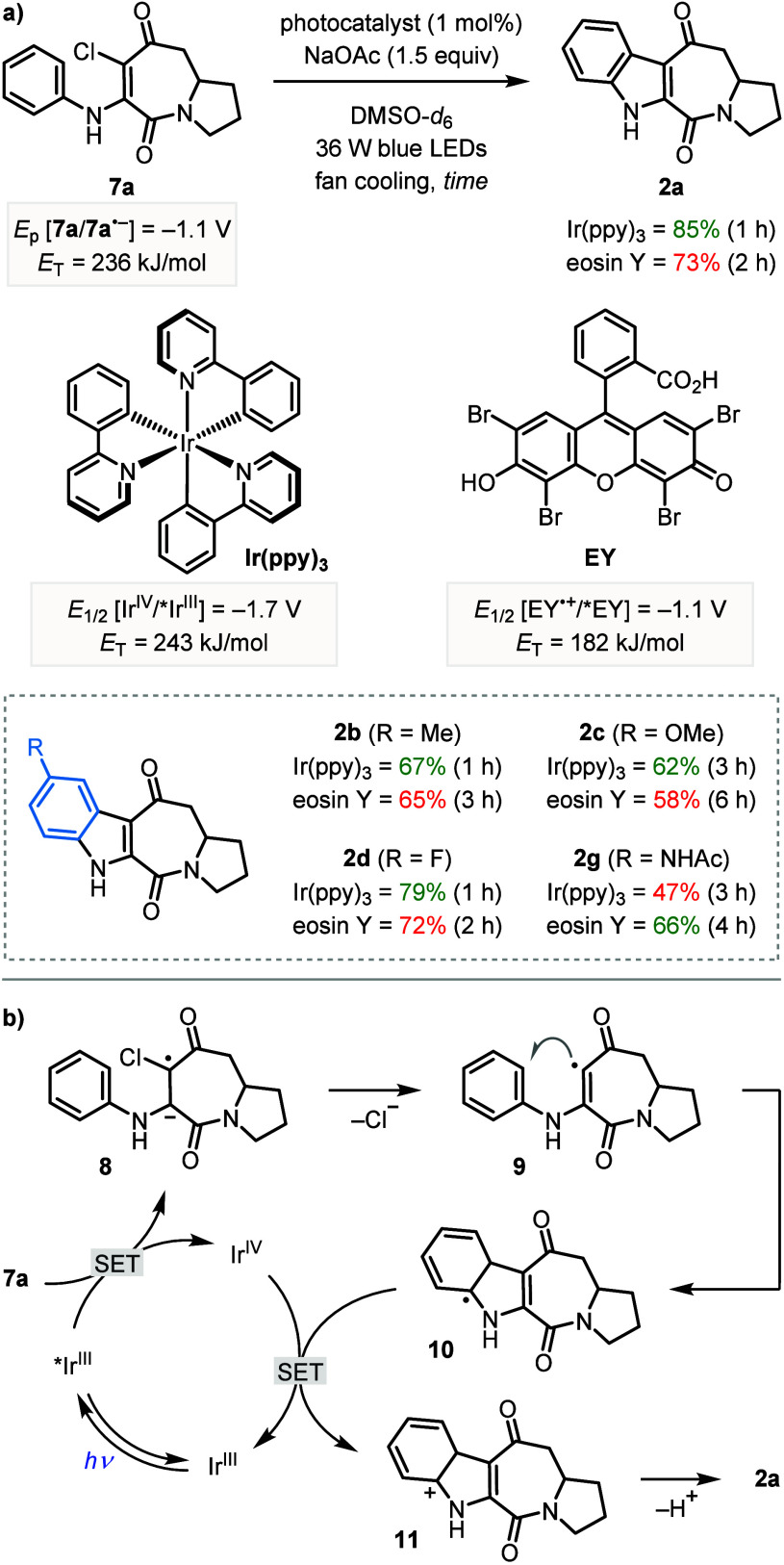
(a) Comparison of catalyst efficiencies. (b) Proposed mechanism

These mechanistic studies also revealed that eosin Y was an effective photocatalyst for indole formation and, therefore, showed potential as a cheaper alternative to Ir­(ppy)_3_. To further demonstrate this, several other indoloazepinediones (**2b**–**2d** and **2g**) were synthesized using eosin Y as the photocatalyst (Scheme 4a). Although slower reaction rates were observed with eosin Y, products **2b**–**2d** were formed in only slightly reduced yields compared to those obtained using Ir­(ppy)_3_. Interestingly, the yield of acetamide **2g** increased from 47% to 66% when using eosin Y; however, the origin of this increase in efficiency is currently unclear.

In conclusion, we have developed a method for the synthesis of indoloazepinone scaffolds using sequential photoinduced reactions, including a photochemical intramolecular [5 + 2] cycloaddition of a dichloro-maleimide and photoredox-catalyzed indole formation from aminated chloro-azepinediones. Formation of the indole relies on single-electron-reduction-induced dechlorinative vinylic radical formation and cyclization onto a tethered aniline. Notably, construction of the indole at a late-stage in the synthesis allows facile diversification of the benzenoid ring, with products substituted in the 4-, 5-, 6- and 7-positions readily accessible.

## Supplementary Material



## Data Availability

The data underlying this study are available in the published article and its Supporting Information.

## Notes

Experimental procedures, characterization data, ^1^H and ^13^C NMR spectra for all novel compounds, crystal data for **7e**, and DFT calculations (PDF)

## Notes

**Safety Statement:** ***Caution!*** Ultraviolet light is damaging to biological tissues. Caution is required when working with the lamp to prevent exposure and protective eyewear must be used at all times.
