# Supplementary material for: Synthesis of Indoloazepinone Scaffolds Using Sequential Photochemical and Photocatalytic Reactions
Source: Org Lett. 2025 Aug 25;27(35):9727–31. doi: 10.1021/acs.orglett.5c02977 (PMC12418503; doi:10.1021/acs.orglett.5c02977)
Supplement: Supplementary file 1 [file ol5c02977_si_001.pdf]

SUPPORTING INFORMATION

Synthesis of Indoloazepinone Scaffolds Using Sequential Photochemical and Photocatalytic Reactions

Kate A. Ellis-Sawyer, Tomos Alderman, Kevin I. Booker-Milburn, Varinder K. Aggarwal* and Adam Noble*

School of Chemistry, University of Bristol, Cantock's Close, Bristol BS8 1TS, United Kingdom

*e-mail: v.aggarwal@bristol.ac.uk, a.noble@bristol.ac.uk

TABLE OF CONTENTS

LIST OF CHARACTERISED PRODUCTS 2

1. MATERIALS AND GENERAL METHODS 4
   1.1. Solvents, Reagents and Reaction Setup 4
   1.2. Photochemical Equipment 4
   1.3. Visible-Light Photoredox Catalysis Equipment 4
   1.4. Chromatography and Instrumentation 5
   1.5. Naming of Compounds 6

2. EXPERIMENTAL DATA 7
   2.1. Synthesis of Azepinedione Starting Material 7
   2.2. Conjugate Substitution Reaction 9
      2.2.1. General Procedure A 9
   2.3. Synthesis of Conjugate Substitution Compounds 10
   2.4. Visible Light Photoredox Catalysis Reaction 22
      2.4.1. General Procedure B 22
      2.4.2. General Procedure C 22
   2.5. Synthesis of Indoloazepinones 24
   2.6. Comparison of Yields in DMSO-d_6 and Anhydrous DMSO-H_6 38

3. X-RAY CRYSTALLOGRAPHIC ANALYSIS 39
   3.1. Aminated Chloro-Azepinedione **7e** (CCDC 2473598) 39

4. MECHANISTIC EXPERIMENTS 41
   4.1. Cyclic Voltammogram of Aminated Chloro-Azepinedione **7a** 41
   4.2. Fluorescence Quenching Studies and UV/vis Spectroscopy 42

5. DFT CALCULATIONS 44
   5.1. Calculated Triplet Energy of **7a** 44

6. SPECTROSCOPIC DATA 46

7. REFERENCES 91

LIST OF CHARACTERISED PRODUCTS

2,3-Dichloromaleimide (**6**) 7

3,4-Dichloro-1-(pent-4-en-1-yl)-1*H*-pyrrole-2,5-dione (**5**) 7

6,7-Dichloro-2,3,9,9a-tetrahydro-1H-pyrrolo[1,2-a]azepine-5,8-dione (**3**) 8

7-Chloro-6-(phenylamino)-2,3,9,9a-tetrahydro-1*H*-pyrrolo[1,2-*a*]azepine-5,8-dione (**7a**) 10

7-Chloro-6-(*p*-tolylamino)-2,3,9,9a-tetrahydro-1*H*-pyrrolo[1,2-*a*]azepine-5,8-dione (**7b**) 10

7-Chloro-6-((4-methoxyphenyl)amino)-2,3,9,9a-tetrahydro-1*H*-pyrrolo[1,2-*a*]azepine-5,8-dione (**7c**) 11

7-Chloro-6-((4-fluorophenyl)amino)-2,3,9,9a-tetrahydro-1*H*-pyrrolo[1,2-*a*]azepine-5,8-dione (**7d**) 11

6-((4-Bromophenyl)amino)-7-chloro-2,3,9,9a-tetrahydro-1*H*-pyrrolo[1,2-*a*]azepine-5,8-dione (**7e**) 12

7-Chloro-6-((4-(4,4,5,5-tetramethyl-1,3,2-dioxaborolan-2-yl)phenyl)amino)-2,3,9,9a-tetrahydro-1*H*-pyrrolo[1,2-*a*]azepine-5,8-dione (**7f**) 13

N-(4-((7-Chloro-5,8-dioxo-2,3,5,8,9,9a-hexahydro-1*H*-pyrrolo[1,2-*a*]azepin-6-yl)amino)phenyl)acetamide (**7g**) 14

6-((4-Acetylphenyl)amino)-7-chloro-2,3,9,9a-tetrahydro-1*H*-pyrrolo[1,2-*a*]azepine-5,8-dione (**7h**) 14

Methyl-4-((7-chloro-5,8-dioxo-2,3,5,8,9,9a-hexahydro-1*H*-pyrrolo[1,2-*a*]azepin-6-yl)amino)benzoate (**7i**) 15

4-((7-Chloro-5,8-dioxo-2,3,5,8,9,9a-hexahydro-1*H*-pyrrolo[1,2-*a*]azepin-6-yl)amino)benzonitrile (**7j**) 16

2-(4-((7-Chloro-5,8-dioxo-2,3,5,8,9,9a-hexahydro-1*H*-pyrrolo[1,2-*a*]azepin-6-yl)amino)phenyl)acetonitrile (**7k**) 16

7-Chloro-6-((4-hydroxyphenyl)amino)-2,3,9,9a-tetrahydro-1*H*-pyrrolo[1,2-*a*]azepine-5,8-dione (**7l**) 17

7-Chloro-6-(*m*-tolylamino)-2,3,9,9a-tetrahydro-1*H*-pyrrolo[1,2-*a*]azepine-5,8-dione (**7m**) 18

7-Chloro-6-((3-isopropylphenyl)amino)-2,3,9,9a-tetrahydro-1*H*-pyrrolo[1,2-*a*]azepine-5,8-dione (**7n**) 18

6-((3-(*tert*-Butyl)phenyl)amino)-7-chloro-2,3,9,9a-tetrahydro-1*H*-pyrrolo[1,2-*a*]azepine-5,8-dione (**7o**) 19

7-Chloro-6-((3-methoxyphenyl)amino)-2,3,9,9a-tetrahydro-1*H*-pyrrolo[1,2-*a*]azepine-5,8-dione (**7p**) 20

3-((7-Chloro-5,8-dioxo-2,3,5,8,9,9a-hexahydro-1*H*-pyrrolo[1,2-*a*]azepin-6-yl)amino)benzonitrile (**7q**) 20

7-Chloro-6-((2-methoxyphenyl)amino)-2,3,9,9a-tetrahydro-1*H*-pyrrolo[1,2-*a*]azepine-5,8-dione (**7r**) 21

2,3,12,12a-Tetrahydropyrrolo[1',2':1,7]azepino[3,4-*b*]indole-5,11(1*H*,6*H*)-dione (**2a**) 24

9-Methyl-2,3,12,12a-tetrahydropyrrolo[1',2':1,7]azepino[3,4-*b*]indole-5,11(1*H*,6*H*)-dione (**2b**) 25

9-Methoxy-2,3,12,12a-tetrahydropyrrolo[1',2':1,7]azepino[3,4-*b*]indole-5,11(1*H*,6*H*)-dione (**2c**) 26

9-Fluoro-2,3,12,12a-tetrahydropyrrolo[1',2':1,7]azepino[3,4-*b*]indole-5,11(1*H*,6*H*)-dione (**2d**) 26

9-Bromo-2,3,12,12a-tetrahydropyrrolo[1',2':1,7]azepino[3,4-*b*]indole-5,11(1*H*,6*H*)-dione (**2e**) 27

9-(4,4,5,5-Tetramethyl-1,3,2-dioxaborolan-2-yl)-2,3,12,12a-tetrahydropyrrolo[1',2':1,7]azepino[3,4-*b*]indole-5,11(1*H*,6*H*)-dione (**2f**) 28

N-(5,11-Dioxo-1,2,3,5,6,11,12,12a-octahydropyrrolo[1',2':1,7]azepino[3,4-*b*]indol-9-yl)acetamide (**2g**) 28

9-Acetyl-2,3,12,12a-tetrahydropyrrolo[1',2':1,7]azepino[3,4-*b*]indole-5,11(1*H*,6*H*)-dione (**2h**) 29

Methyl-5,11-dioxo-1,2,3,5,6,11,12,12a-octahydropyrrolo[1',2':1,7]azepino[3,4-*b*]indole-9-carboxylate (**2i**) 30

5,11-Dioxo-1,2,3,5,6,11,12,12a-octahydropyrrolo[1',2':1,7]azepino[3,4-*b*]indole-9-carbonitrile (**2j**) 30

2-(5,11-Dioxo-1,2,3,5,6,11,12,12a-octahydropyrrolo[1',2':1,7]azepino[3,4-*b*]indol-9-yl)acetonitrile (**2k**) 31

6-((4-Hydroxyphenyl)amino)-2,3,9,9a-tetrahydro-1*H*-pyrrolo[1,2-*a*]azepine-5,8-dione (**7l'**) 31

10-Methyl-2,3,12,12a-tetrahydropyrrolo[1',2':1,7]azepino[3,4-*b*]indole-5,11(1*H*,6*H*)-dione and 8-methyl-2,3,12,12a-tetrahydropyrrolo[1',2':1,7]azepino[3,4-*b*]indole-5,11(1*H*,6*H*)-dione (**2m**) 32

10-Isopropyl-2,3,12,12a-tetrahydropyrrolo[1',2':1,7]azepino[3,4-*b*]indole-5,11(1*H*,6*H*)-dione and 8-isopropyl-2,3,12,12a-tetrahydropyrrolo[1',2':1,7]azepino[3,4-*b*]indole-5,11(1*H*,6*H*)-dione (**2n**) 33

10-(*tert*-Butyl)-2,3,12,12a-tetrahydropyrrolo[1',2':1,7]azepino[3,4-*b*]indole-5,11(1*H*,6*H*)-dione and 8-(*tert*-butyl)-2,3,12,12a-tetrahydropyrrolo[1',2':1,7]azepino[3,4-*b*]indole-5,11(1*H*,6*H*)-dione (**2o**) 34

10-Methoxy-2,3,12,12a-tetrahydropyrrolo[1',2':1,7]azepino[3,4-*b*]indole-5,11(1*H*,6*H*)-dione (**2p**) and 8-methoxy-2,3,12,12a-tetrahydropyrrolo[1',2':1,7]azepino[3,4-*b*]indole-5,11(1*H*,6*H*)-dione (**2p'**) 35

5,11-Dioxo-1,2,3,5,6,11,12,12a-octahydropyrrolo[1',2':1,7]azepino[3,4-*b*]indole-10-carbonitrile (**2q**) and 5,11-dioxo-1,2,3,5,6,11,12,12a-octahydropyrrolo[1',2':1,7]azepino [3,4-*b*]indole-8-carbonitrile (**2q'**)........................ 36

7-Methoxy-2,3,12,12a-tetrahydropyrrolo[1',2':1,7]azepino[3,4-*b*]indole-5,11(1*H*,6*H*)-dione (**2r**) 37

1. MATERIALS AND GENERAL METHODS

1.1. Solvents, Reagents and Reaction Setup

All anhydrous solvents were commercially supplied or dried using an Anhydrous Engineering alumina column drying system (THF, toluene, Et_2O, CH_2Cl_2). Reagents were purchased from commercial sources and used as received. Oil baths were used for heating reactions.

1.2. Photochemical Equipment

A 125 W medium pressure mercury lamp (λ_{max} = 366 nm), placed in a Pyrex® cooling jacket, was used to irradiate the reaction solution with UVA light. The lamp and cooling jacket were submerged in the reaction solution and nitrogen gas bubbled through the solution for the duration of the reaction.

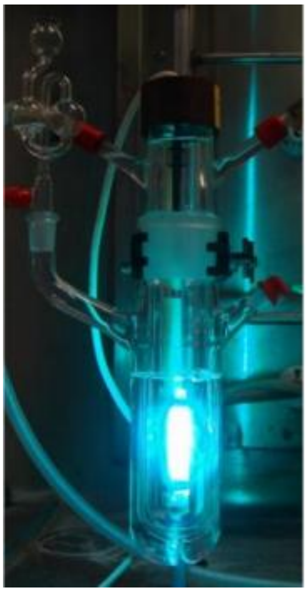

1.3. Visible-Light Photoredox Catalysis Equipment

A modified heater stirrer block was used to stir the reaction mixture and irradiate the solution with blue light (450–460 nm) using a single 36 W COB LED manufactured by Citizen Electronics (CLU048-1212-B455). The reaction vials or round bottom flask were held approximately 1.5 cm above the light source. The reaction mixture was cooled from the side using an electric fan.

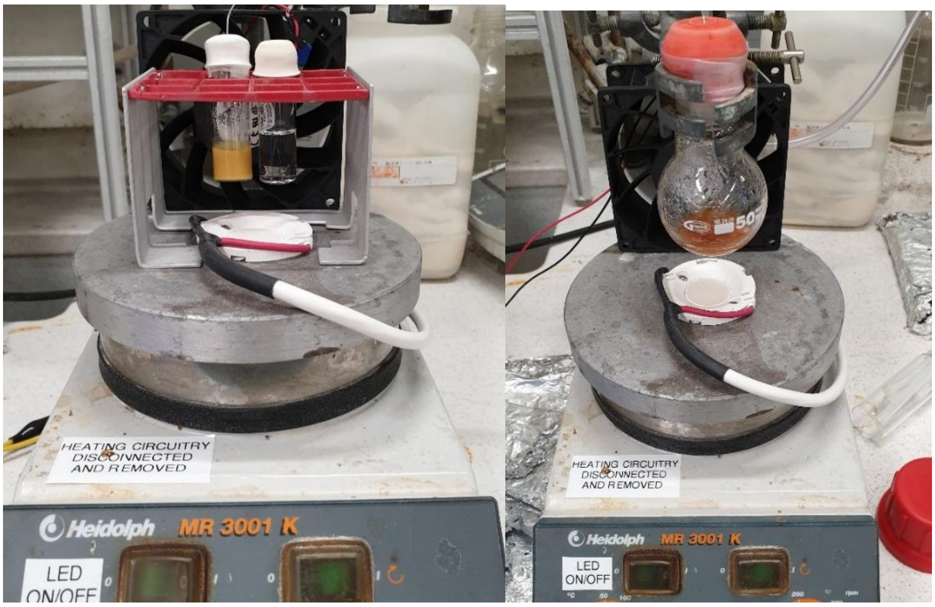

1.4. Chromatography and Instrumentation

Thin layer chromatography (TLC) was performed using Merck Kieselgel 60 F254 fluorescent treated silica, which was visualised under UV light, or by staining with aqueous basic potassium permanganate followed by heating, as stated.

Flash column chromatography (FCC) was carried out using Sigma-Aldrich silica gel (60 Å, 230–400 mesh, 40–63 μm).

NMR spectra were recorded at various field strengths, as indicated, using Bruker 400 MHz, Varian VNMR 400 MHz, Varian VNMR 500 MHz, or Bruker Cryo 500 MHz for ^{1}H, ^{11}B, ^{13}C and ^{19}F acquisitions. All NMR spectra were recorder at 25 °C unless otherwise stated. Chemical shifts (δ) are reported in parts per million (ppm) and referenced $CDCl_3$ (^{1}H: 7.26 ppm; ^{13}C: 77.0 ppm) or DMSO-d_6 (^{1}H: 2.50 ppm; ^{13}C: 39.5 ppm). Coupling constants (J) are given in Hertz (Hz) and refer to apparent multiplicities (s = singlet, d = doublet, t = triplet, q = quartet, quin = quintet, sex = hextet, h = heptet, m = multiplet, br = broad signal, dd = doublet of doublets, etc.). The ^{1}H NMR spectra are reported as follows: chemical shift (multiplicity, coupling constants, number of protons)

High resolution mass spectra (HRMS) were recorded on a Bruker Daltonics MicrOTOF II by Electrospray Ionisation (ESI).

IR spectra were recorded neat as a thin film on a Perkin Elmer Spectrum One FT-IR. Selected absorption maxima (ν_{max}) are reported in wavenumbers (cm^{-1}).

Cyclic voltammograms were recorded at room temperature using a PalmSens EmStat4S with a cylindrical glassy carbon working electrode (surface area = 7.1 mm^2), a 0.1 M Ag/$AgNO_3$ reference electrode and a platinum wire counter electrode. The glassy carbon electrode was mechanically polished using a cloth polishing pad. An alumina-water slurry was added to the pad and the electrode polished using a figure of eight movement. The cyclic voltammogram was plotted using the IUPAC convention.

1.5. Naming of Compounds

Compound names are those generated by ChemDraw Professional 20.0 software (PerkinElmer), following the IUPAC nomenclature.

2. EXPERIMENTAL DATA

2.1. Synthesis of Azepinedione Starting Material

2,3-Dichloromaleimide (6)

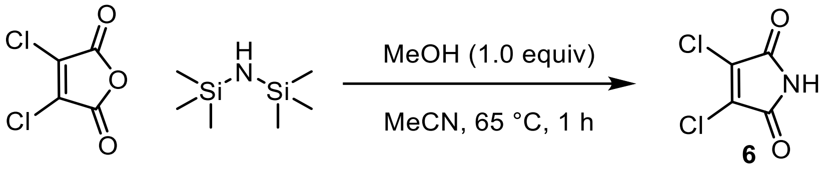

To a solution of 2,3-dichloromaleic anhydride (12.0 g, 71.9 mmol, 1.0 equiv) in MeCN (125 mL) was added HMDS (30.1 mL, 144 mmol, 2.0 equiv). MeOH (2.92 mL, 71.9 mmol, 1.0 equiv) was added dropwise (CAUTION - EXOTHERM) and a precipitate was formed. The suspension was stirred at 65 °C for 1 h and then the solvent removed *in vacuo*. The crude product was purified by flash column chromatography (20:80 to 30:70, EtOAc:Pet. Ether 40-60 °C) to give **6** (1.23 g, 49%, 3.54 mmol) as a pink amorphous solid.[1]

Mp: 177-179 °C (recrystallised from EtOH).

NMR Spectroscopy (see spectra):

^{1}**H NMR** (400 MHz, DMSO-d_6): δ_H 11.71 (1H, s) ppm;

^{13}**C NMR** (101 MHz, DMSO-d_6): δ_C 164.0, 132.8 ppm.

IR (film): ν_{max} 3212, 1734, 1610 cm^{-1}.

3,4-Dichloro-1-(pent-4-en-1-yl)-1*H*-pyrrole-2,5-dione (5)

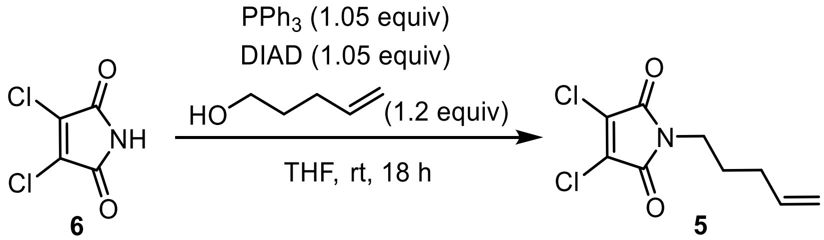

Under an atmosphere of N_2, a solution of triphenylphosphine (9.13 g, 34.8 mmol, 1.05 equiv) in anhydrous THF (133 mL) was cooled to -78 °C. DIAD (6.85 mL, 34.8 mmol, 1.05 equiv) was added dropwise, and the solution stirred for 1 h at -78 °C. 4-Pentene-1-ol (4.11 mL, 39.8 mmol, 1.2 equiv) was added and the suspension stirred at -78 °C for 0.5 h. **6** (5.51 g, 33.2 mmol, 1.0 equiv) was added and the suspension allowed to warm to room temperature. The reaction mixture was stirred overnight and then the solvent removed *in vacuo*. The crude product was triturated with Et2O:Pet. Ether 40-60 °C (50 mL, 80:20). The precipitate was removed by filtration and the solvent removed from the filtrate *in vacuo*. The crude product was purified by flash column chromatography (5:95, EtOAc:Pet. Ether 40-60 °C) to give **5** (5.48 g, 71%, 23.4 mmol) as a yellow oil.[1]

NMR Spectroscopy (see spectra):

^{1}H NMR (400 MHz, $CDCl_3$): δ_H 5.77 (ddt, J = 16.9, 10.2, 6.6 Hz, 1H), 5.04 (ddd, J = 16.9, 3.5, 1.7 Hz, 1H), 5.00 (ddd, J = 10.2, 3.54, 1.35 Hz, 1H), 3.63–3.59 (m, 2H), 2.10–2.05 (m, 2H), 1.76– 1.69 (m, 2H) ppm;

^{13}C NMR (101 MHz, $CDCl_3$): δ_C 163.2, 136.9, 133.4, 115.8, 39.1, 30.9, 27.5 ppm.

All recorded spectroscopic data matched those previously reported in the literature.[2]

6,7-Dichloro-2,3,9,9a-tetrahydro-1H-pyrrolo[1,2-a]azepine-5,8-dione (3)

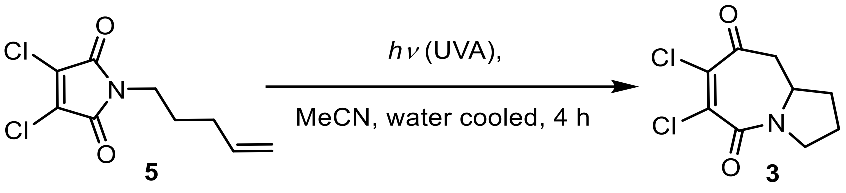

A solution of **5** (2.55 g, 10.9 mmol, 1.0 equiv) in MeCN (150 mL) was degassed under reduced pressure and then backfilled with N_2 gas. The reaction mixture was irradiated for 4 h using a 125 W medium pressure mercury lamp and N_2 bubbled through the solution. The solvent was removed in vacuo and the crude product purified by flash column chromatography (40:60 to 70:30, EtOAc:Pet. Ether 40-60 °C) to give **3** (1.61 g, 63%, 6.89 mmol) as a pale yellow amorphous solid.[1]

Mp: 177-179 °C (recrystallised from EtOH).

NMR Spectroscopy (see spectra):

[1]**H NMR** (400 MHz, $CDCl_3$): δ_H 4.38 (dddd, J = 9.2, 8.2, 5.8, 3.9 Hz, 1H), 3.74–3.65 (m, 2H), 2.98–2.88 (m, 2H), 2.42–2.24 (m, 1H), 2.07–1.95 (m, 2H), 1.85–1.78 (m, 1H);

[13]**C NMR** (101 MHz, $CDCl_3$): δ_C 190.8, 158.4, 139.4, 136.9, 52.6, 50.7, 47.5, 31.6, 23.2.

All recorded spectroscopic data matched those previously reported in the literature.[2]

2.2. Conjugate Substitution Reaction

2.2.1. General Procedure A

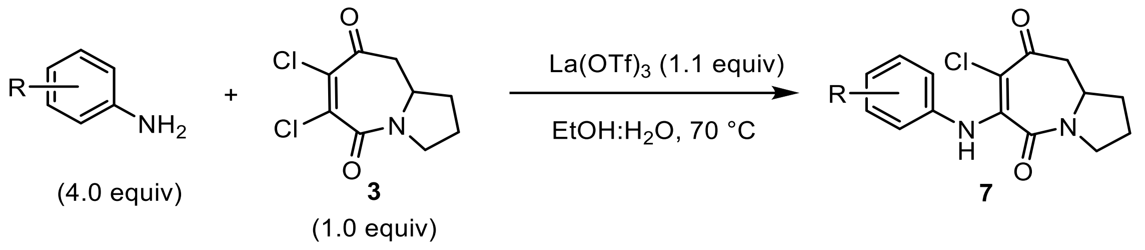

The specified aniline (4.0 equiv) and $La(OTf)_3$ (1.1 equiv) were added to a suspension of **3** (1.0 equiv) in EtOH (abs.):H_2O (2:1, 0.1 M) and the reaction mixture stirred at 70 °C for the stated time. The solution was diluted with toluene (5.0 mL/mmol) and EtOH removed *in vacuo*. The reaction mixture was diluted with EtOAc (5.0 mL/mmol) and the organic layer washed with H_2O (2 × 5.0 mL/mmol). The aqueous layers were then combined and extracted with EtOAc (2 × 15 mL/mmol). All organic layers were combined, washed with sat. brine (30 mL/mmol), dried over $MgSO_4$, filtered and the solvent removed *in vacuo*.

2.3. Synthesis of Conjugate Substitution Compounds

7-Chloro-6-(phenylamino)-2,3,9,9a-tetrahydro-1*H*-pyrrolo[1,2-*a*]azepine-5,8-dione (7a)

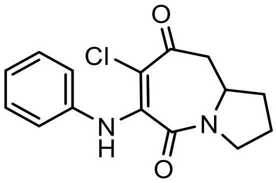

7a

Prepared following **General Procedure A**, using aniline (312 µL, 3.4 mmol), $La(OTf)_3$ (551 mg, 0.94 mmol) and **3** (200 mg, 0.85 mmol) with a reaction time of 4 h. Purification by flash column chromatography (90:10 toluene:acetone) gave **7a** (211 mg, 85%, 0.73 mmol) as a yellow solid foam.

Mp: 170-171 °C (recrystallised from EtOH).

NMR Spectroscopy (see spectra):

^{1}**H NMR** (400 MHz, $CDCl_3$): δ_H 7.32–7.28 (m, 3H), 7.15–7.11 (m, 1H), 6.94–6.92 (m, 2H), 4.46 (ddt, *J* = 10.7, 7.3, 3.2 Hz, 1H), 3.79 (ddd, J = 12.8, 7.6, 5.8 Hz, 1H), 3.60 (dt, *J* = 12.8, 7.2 Hz, 1H), 2.90 (dd, *J* = 18.4, 10.7 Hz, 1H), 2.83 (dd, *J* = 18.4, 3.2, 1H) 2.39 (ddt, *J* = 13.6, 7.8, 7.3 Hz, 1H), 2.11–1.95 (m, 2H), 1.88–1.81 (m, 1H);

^{13}**C NMR** (101 MHz, $CDCl_3$): δ_C 189.6, 158.6, 145.0, 139.6, 129.2, 124.9, 121.2, 114.1, 53.1, 49.9, 46.9, 32.3, 23.1.

IR (film): ν_{max} 3298, 2956, 2925, 1652 cm^{-1}.

HRMS (ESI$^+$): m/z calc'd for $C_{15}H_{16}{}^{35}ClN_2O_2$ $[M+H]^+$, 291.0895; found, 291.0903.

7-Chloro-6-(*p*-tolylamino)-2,3,9,9a-tetrahydro-1*H*-pyrrolo[1,2-*a*]azepine-5,8-dione (7b)

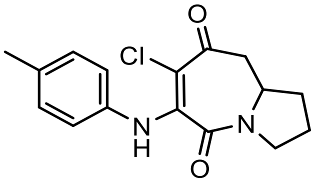

7b

Prepared following **General Procedure A**, using *p*-toluidine (183 mg, 1.7 mmol), $La(OTf)_3$ (275 mg, 0.47 mmol) and **3** (100 mg, 0.43 mmol) with a reaction time of 2 h. Purification by flash column chromatography (90:10 to 100:0 Et_2O:Pet. Ether) gave **7b** (203 mg, 79%, 0.34 mmol) as a yellow amorphous solid.

Mp: decomposition 180 °C (recrystallised from EtOH).

NMR Spectroscopy (see spectra):

^{1}**H NMR** (400 MHz, $CDCl_3$): δ_H 7.29 (s, 1H), 7.11–7.09 (m, 2H), 6.85–6.82 (m, 2H), 4.46 (ddt, *J* = 10.7, 7.8, 3.0 Hz, 1H), 3.78 (ddd, *J* = 12.6, 7.6, 5.8, 1H), 3.58 (dt, J = 12.6, 7.6 Hz, 1H), 2.89 (dd, *J* = 18.4, 10.7 Hz,

1H), 2.81 (dd, *J* = 18.4, 3.0 Hz, 1H), 2.39 (ddt, *J* = 12.8, 7.8, 7.6 Hz, 1H), 2.31 (s, 3H), 2.10–1.94 (m, 2H), 1.87–1.80 (m, 1H);

^{13}C NMR (101 MHz, $CDCl_3$): δ_C 189.5, 158.6, 145.5, 137.1, 134.9, 129.8, 121.4, 113.3, 53.2, 49.9, 46.8, 32.3, 23.1, 21.1.

IR (film): ν_{max} 3296, 2928, 1652, 1591, 1557 cm^{-1}.

HRMS (ESI$^+$): m/z calc'd for $C_{16}H_{18}{}^{35}ClN_2O_2$ [M+H]$^+$, 305.1051; found, 305.1054.

7-Chloro-6-((4-methoxyphenyl)amino)-2,3,9,9a-tetrahydro-1*H*-pyrrolo[1,2-*a*]azepine-5,8-dione (7c)

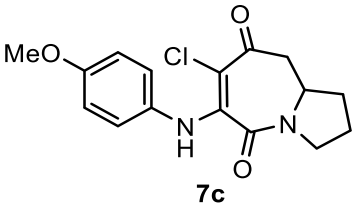

7c

Prepared following **General Procedure A**, using *p*-anisidine (211 mg, 1.7 mmol), $La(OTf)_3$ (275 mg, 0.47 mmol) and **3** (100 mg, 0.43 mmol) with a reaction time of 1 h. Purification by flash column chromatography (0:100 to 20:80 EtOAc:Et_2O) gave **7c** (126 mg, 92%, 0.39 mmol) as a yellow solid foam.

Mp: 167-168 °C (recrystallised from EtOH).

NMR Spectroscopy (see spectra)**:**

^{1}H NMR (400 MHz, $CDCl_3$): δ_H 7.27 (s, 1H), 6.92–6.89 (m, 2H), 6.86–6.82 (m, 2H), 4.43 (ddt, *J* = 10.7, 7.8, 2.9 Hz, 1H), 3.81–3.74 (m, 4H), 3.56 (dt, *J* = 12.5, 7.2 Hz, 1H), 2.88 (dd, *J* = 18.4, 10.7 Hz, 1H), 2.80 (dd, *J* = 18.4, 2.9, 1H), 2.37 (ddt, *J* = 12.8, 7.8, 7.3 Hz, 1H), 2.08–1.94 (m, 2H), 1.86–1.79 (m, 1H);

^{13}C NMR (101 MHz, $CDCl_3$): δ_C 189.3, 158.6, 157.3, 145.9, 132.7, 123.3, 114.5, 112.6, 55.6, 53.2, 49.8, 46.8, 32.3, 23.0.

IR (film): ν_{max} 3314, 2928, 1653, 1557, 1511 cm^{-1}.

HRMS (ESI$^+$): m/z calc'd for $C_{16}H_{17}{}^{35}ClN_2NaO_3$ [M+Na]$^+$, 343.0820; found, 343.0826.

7-Chloro-6-((4-fluorophenyl)amino)-2,3,9,9a-tetrahydro-1*H*-pyrrolo[1,2-*a*]azepine-5,8-dione (7d)

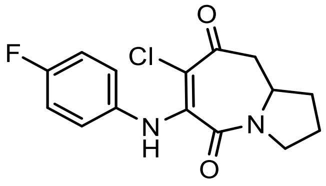

7d

Prepared following **General Procedure A**, using 4-fluoroaniline (809 µL, 8.5 mmol), $La(OTf)_3$ (1.38 g, 2.4 mmol)

and **3** (500 mg, 2.1 mmol) with a reaction time of 5 h. Purification by flash column chromatography (90:10, toluene:acetone) gave **7d** (398 mg, 60%, 1.3 mmol) as a pale yellow amorphous solid.

Mp: 180-182 °C (recrystallised from EtOH).

NMR Spectroscopy (see spectra):

^{1}**H NMR** (400 MHz, $CDCl_3$): δ_H 7.02–7.97 (m, 2H), 6.93–6.90 (m, 2H), 4.43 (ddt, *J* = 10.4, 7.4, 3.4 Hz, 1H), 3.78 (ddd, *J* = 12.2, 7.5, 5.8 Hz, 1H), 3.59 (dt, *J* = 12.2, 7.2 Hz, 1H), 2.89 (dd, *J* = 18.5, 10.4 Hz, 1H), 2.83 (dd, *J* = 18.5, 3.4 Hz, 1H), 2.38 (ddt, *J* = 13.7, 7.7, 7.4 Hz, 1H), 2.09–1.93 (m, 2H), 1.88–1.80 (m, 1H);

^{19}**F NMR,** ^{1}H decoupled (377 MHz, $CDCl_3$): δ_F -117.3;

^{13}**C NMR** (126 MHz, $CDCl_3$): δ_C 189.5, 160.1 (d, *J* = 244.8 Hz), 158.5, 145.1, 135.7 (d, *J* = 3.0 Hz), 123.2 (d, *J* = 8.3 Hz), 116.0 (d, *J* = 23.0 Hz), 113.9, 53.2, 49.8, 47.0, 32.3, 23.0.

IR (film): ν_{max} 3282, 2969, 1653, 1607, 1559, 1509 cm^{-1}.

HRMS (ESI$^+$): m/z calc'd for $C_{15}H_{15}{}^{35}ClFN_2O_2$ [M+H]$^+$, 309.0801; found, 309.0802.

6-((4-Bromophenyl)amino)-7-chloro-2,3,9,9a-tetrahydro-1*H*-pyrrolo[1,2-*a*]azepine-5,8-dione (7e)

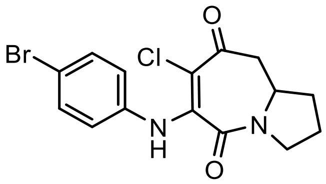

7e

Prepared following **General Procedure A**, using 4-bromoaniline (1.03 g, 6.0 mmol), $La(OTf)_3$ (0.964 g, 1.6 mmol) and **3** (0.350 g, 1.5 mmol) with a reaction time of 24 h. Purification by flash column chromatography (100%, Et_2O) gave **7e** (0.270 g, 49%, 0.73 mmol) as a yellow amorphous solid.

Mp: decomposition 178 °C (recrystallised from EtOH).

NMR Spectroscopy (see spectra):

^{1}**H NMR** (400 MHz, $CDCl_3$): δ_H 7.42–7.39 (m, 2H), 6.81–6.77 (m, 2H), 4.44 (ddt, *J* = 10.1, 7.7, 3.4 Hz, 1H), 3.78 (ddd, *J* = 12.5, 7.7, 5.9, 1H), 3.62 (dt, J = 12.5, 7.2 Hz, 1H), 2.90 (dd, *J* = 17.6, 10.1 Hz, 1H), 2.84 (dd, *J* = 17.6, 3.4, 1H), 2.39 (ddt, *J* = 12.9, 7.7, 7.6 Hz, 1H), 2.10–1.96 (m, 2H), 1.88–1.81 (m, 1H);

^{13}**C NMR** (126 MHz, $CDCl_3$): δ_C 189.5, 158.5, 144.2, 138.7, 132.1, 122.7, 117.7, 114.9, 53.1, 49.8, 47.0, 32.3, 23.0.

IR (film): ν_{max} 3293, 2971, 1654, 1585, 1557 cm^{-1}.

HRMS (ESI$^+$): m/z calc'd for $C_{15}H_{15}{}^{79}Br^{35}ClN_2O_2$ [M+H]$^+$, 369.0000; found, 368.9996.

7-Chloro-6-((4-(4,4,5,5-tetramethyl-1,3,2-dioxaborolan-2-yl)phenyl)amino)-2,3,9,9a-tetrahydro-1*H*-pyrrolo[1,2-*a*]azepine-5,8-dione (7f)

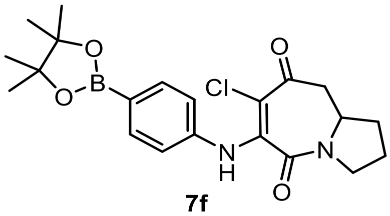

7f

4-Aminophenylboronic acid pinacol ester (935 mg, 4.3 mmol, 2.0 equiv) and La(OTf)$_3$ (1.38 g, 2.4 mmol, 1.1 equiv) were added to a suspension of **3** (500 mg, 2.1 mmol, 1.0 equiv) in EtOH (abs.):H_2O (14.3 mL:7.1 mL, 0.1 M) and the reaction mixture stirred at 70 °C for 2 h. Another portion of 4-aminophenylboronic acid pinacol ester (468 mg, 2.1 mmol, 1.0 equiv) was added and the reaction mixture stirred at 70 °C for 2 h. A final portion of 4-aminophenylboronic acid pinacol ester (468 mg, 2.1 mmol, 1.0 equiv) was added and the reaction mixture stirred at 70 °C for 4 h.

The solution was diluted with toluene (15 mL) and EtOH removed *in vacuo*. The reaction mixture was diluted with EtOAc (15 mL) and washed with H_2O (2 × 15 mL). The aqueous layers were combined and extracted with EtOAc (3 × 40 mL). All organic layers were combined, washed with sat. brine (75 mL), dried over $MgSO_4$, filtered and the solvent removed *in vacuo*. The crude product was purified by flash column chromatography (90:10, toluene:acetone). Some impurities still remained and the compound was repurified by flash column chromatography (95:5, Et_2O:EtOAc) to give **7f** (302 mg, 34%, 0.72 mmol) as a yellow solid foam.

Mp: decomposition 203 °C (recrystallised from EtOH).

NMR Spectroscopy (see spectra)**:**

^{1}H NMR (400 MHz, $CDCl_3$): δ_H 7.75–7.73 (m, 2H), 7.35 (s, 1H), 6.89–6.87 (m, 2H), 4.47 (ddt, *J* = 9.9, 7.7, 2.8 Hz, 1H), 3.77 (ddd, *J* = 12.3, 7.3, 6.2 Hz, 1H), 3.60 (dt, *J* = 12.3, 7.0 Hz, 1H), 2.90 (dd, *J* = 18.5, 9.9 Hz, 1H), 2.83 (dd, *J* = 18.5, 2.8, 1H), 2.39 (ddt, *J* = 12.8, 7.7, 7.6 Hz, 1H), 2.05–1.97 (m, 2H), 1.86–1.81 (m, 1H), 1.33 (s, 12H);

^{11}B NMR (128 MHz, $CDCl_3$): δ_C 30.8;

^{13}C NMR (126 MHz, $CDCl_3$): δ_C 189.7, 158.7, 144.4, 142.0, 135.9, 119.9, 115.0, 83.9, 53.0, 50.0, 46.9, 32.2, 25.02, 24.97, 23.1. The carbon attached to boron was not observed due to quadrupolar relaxation.

IR (film): ν_{max} 3295, 2977, 1654, 1649, 1607, 1580, 1555 cm^{-1}.

HRMS (ESI$^+$): m/z calc'd for $C_{21}H_{27}{}^{11}B^{35}ClN_2O_4$ [M+H]$^+$, 417.1752; found, 417.1752.

N-(4-((7-Chloro-5,8-dioxo-2,3,5,8,9,9a-hexahydro-1H-pyrrolo[1,2-a]azepin-6-yl)amino)phenyl)acetamide (7g)

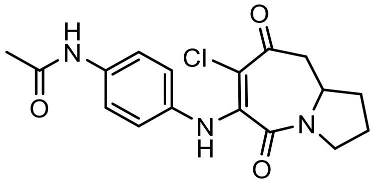

7g

4′-Aminoacetanilide (257 mg, 1.7 mmol, 4.0 equiv) and La(OTf)$_3$ (275 mg, 0.47 mmol, 1.1 equiv) were added to a suspension of **3** (100 mg, 0.43 mmol, 1.0 equiv) in EtOH (abs.):H_2O (2.7 mL:1.4 mL, 0.1 M) and the reaction mixture stirred at 70 °C for 2 h. A precipitate was formed and the product was isolated using vacuum filtration to give **7g** (97.0 mg, 65%, 0.28 mmol) as a yellow amorphous solid.

Mp: decomposition 236 °C (recrystallised from EtOH).

NMR Spectroscopy (see spectra):

^{1}**H NMR** (400 MHz, DMSO-d_6): δ_H 9.86 (s, 1H), 8.79 (s, 1H), 7.46–7.42 (m, 2H), 6.97–6.93 (m, 2H), 4.56–4.51 (m, 1H), 3.59 (ddd, J = 12.4, 7.9, 4.8 Hz, 1H), 3.30–3.26 (m, 1H), 2.92 (dd, J = 18.6, 11.6 Hz, 1H), 2.66 (dd, J = 18.6, 1.7 Hz, 1H), 2.30–2.20 (m, 1H), 2.02 (s, 3H), 1.98–1.93 (m, 1H), 1.89–1.84 (m, 1H), 1.76–1.69 (m, 1H);

^{13}**C NMR** (126 MHz, DMSO-d_6): δ_C 190.0, 168.0, 157.7, 146.8, 135.9, 135.5, 122.0, 119.1, 110.6, 52.6, 49.5, 45.8, 31.0, 23.9, 22.2.

IR (film): ν_{max} 3303, 2987, 1650, 1604, 1558, 1514 cm^{-1}.

HRMS (ESI$^+$): m/z calc'd for $C_{17}H_{19}{}^{35}ClN_3O_3$ [M+H]$^+$, 348.1109; found, 348.1105.

6-((4-Acetylphenyl)amino)-7-chloro-2,3,9,9a-tetrahydro-1H-pyrrolo[1,2-a]azepine-5,8-dione (7h)

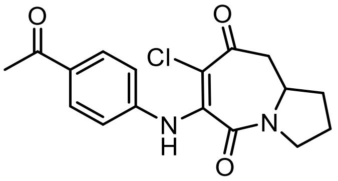

7h

Prepared following **General Procedure A**, using 4'-aminoacetophenone (1.15 g, 8.5 mmol), La(OTf)$_3$ (1.38 g, 2.4 mmol) and **3** (0.500 g, 2.1 mmol) with a reaction time of 48 h. Purification by flash column chromatography (90:10 to 80:20, toluene:acetone) gave **7h** (0.270 g, 49%, 0.73 mmol) as a yellow amorphous solid.

Mp: 180-182 °C (recrystallised from EtOH).

NMR Spectroscopy (see spectra):

^{1}**H NMR** (400 MHz, $CDCl_3$): δ_H 7.92–7.89 (m, 2H), 7.40 (s, 1H), 6.92–6.89 (m, 2H), 4.49 (ddt, J = 10.1, 7.7,

3.7 Hz, 1H), 3.80 (ddd, *J* =12.7, 7.6, 5.8 Hz, 1H), 3.65 (dt, *J* = 12.7, 7.3 Hz, 1H), 2.91 (dd, *J* = 18.3, 10.1 Hz, 1H), 2.87 (dd, *J* = 18.3, 3.7 Hz, 1H), 2.56 (s, 3H), 2.41 (ddt, *J* = 12.9, 7.8, 7.7 Hz, 1H), 2.11–2.01 (m, 2H), 1.90–1.84 (m, 1H);

^{13}**C NMR** (126 MHz, $CDCl_3$): δ_C 196.7, 189.8, 158.6, 143.7, 143.2, 132.8, 129.8, 119.7, 117.0, 53.0, 50.0, 47.1, 32.2, 26.6, 23.1.

IR (film): ν_{max} 3297, 2924, 1712, 1654, 1605, 1587, 1560 cm^{-1}.

HRMS (ESI$^+$): m/z calc'd for $C_{17}H_{18}{}^{35}ClN_2O_3$ [M+H]$^+$, 333.1006; found, 333.1005.

Methyl-4-((7-chloro-5,8-dioxo-2,3,5,8,9,9a-hexahydro-1*H*-pyrrolo[1,2-*a*]azepin-6-yl)amino)benzoate (7i)

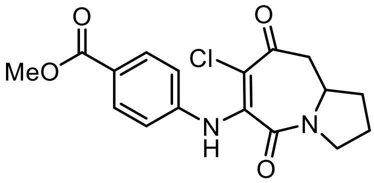

7i

Prepared following **General Procedure A**, using methyl 4-aminobenzoate (2.58 g, 17.1 mmol), La(OTf)$_3$ (2.75 g, 4.7 mmol) and **3** (1.00 g, 4.3 mmol) with a reaction time of 48 h. Purification by flash column chromatography (95:5 to 90:10, toluene:acetone) gave **7i** (0.423 g, 28%, 1.2 mmol) as a yellow amorphous solid.

Mp: decomposition 217 °C (recrystallised from EtOH).

NMR Spectroscopy (see spectra)**:**

^{1}**H NMR** (400 MHz, $CDCl_3$): δ_H 7.99–7.96 (m, 2H), 7.40 (s, 1H), 6.91–6.88 (m, 2H), 4.48 (ddt, *J* = 10.9, 7.5, 3.7 Hz, 1H), 3.89 (s, 3H), 3.83–3.76 (m, 1H), 3.68–3.62 (m, 1H), 2.92 (dd, *J* = 17.5, 10.9 Hz, 1H), 2.87 (dd, *J* = 17.5, 3.7 Hz, 1H), 2.40 (ddt, *J* = 10.4, 7.8, 7.5 Hz, 1H), 2.11–1.98 (m, 2H), 1.91–1.82 (m, 1H);

^{13}**C NMR** (126 MHz, $CDCl_3$): δ_C 189.8, 166.6, 158.6, 143.6, 143.3, 130.9, 125.6, 119.7, 116.7, 53.0, 52.2, 49.9, 47.1, 32.2, 23.1.

IR (film): ν_{max} 3298, 2952, 1713, 1655, 1605, 1587, 1515 cm^{-1}.

HRMS (ESI$^+$): m/z calc'd for $C_{17}H_{18}{}^{35}ClN_2O_4$ [M+H]$^+$, 349.0955; found, 349.0956.

4-((7-Chloro-5,8-dioxo-2,3,5,8,9,9a-hexahydro-1*H*-pyrrolo[1,2-*a*]azepin-6-yl)amino)benzonitrile (7j)

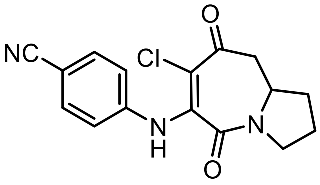

7j

Prepared following **General Procedure A**, using 4-aminobenzonitrile (2.02 g, 17.1 mmol), La(OTf)$_3$ (2.75 g, 4.7 mmol) and **3** (1.00 g, 4.3 mmol) with a reaction time of 72 h. Purification by flash column chromatography (95:5 to 90:10, toluene:acetone) gave **7j** (0.140 g, 10%, 0.44 mmol) as a yellow amorphous solid.

Mp: decomposition 197 °C (recrystallised from EtOH).

NMR Spectroscopy (see spectra)**:**

^{1}**H NMR** (500 MHz, CDCl$_3$): δ_H 7.58–7.55 (m, 2H, 7.41 (s, 1H), 6.91–6.88 (m, 2H), 4.45 (ddt, J = 10.4, 7.8, 3.6 Hz, 1H), 3.80 (ddd, J = 12.7, 7.6, 5.7 Hz, 1H), 3.67 (dt, J = 12.7, 7.2 Hz, 1H), 2.92 (dd, *J* = 18.5, 10.4 Hz, 1H), 2.88 (dd, *J* = 18.5, 3.3 Hz, 1H), 2.40 (ddt, *J* = 12.7, 7.9, 7.8 Hz, 1H), 2.11–2.00 (m, 2H), 1.90–1.84 (m, 1H);

^{13}**C NMR** (126 MHz, CDCl$_3$): δ_C 189.7, 158.5, 143.4, 142.3, 133.2, 120.1, 118.9, 118.1, 106.7, 53.0, 49.9, 47.2, 32.2, 23.1.

IR (film): ν_{max} 3289, 2924, 2222, 1654, 1606, 1584, 1557 cm^{-1}.

HRMS (ESI$^+$): m/z calc'd for $C_{16}H_{15}{}^{35}ClN_3O_2$ [M+H]$^+$, 316.0853; found, 316.0857.

2-(4-((7-Chloro-5,8-dioxo-2,3,5,8,9,9a-hexahydro-1*H*-pyrrolo[1,2-*a*]azepin-6-yl)amino)phenyl)acetonitrile (7k)

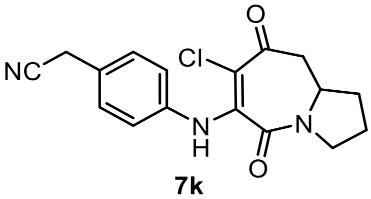

7k

4-Aminobenzyl cyanide (452 mg, 3.4 mmol, 4.0 equiv) and La(OTf)$_3$ (551 mg, 0.94 mmol, 1.1 equiv) were added to a suspension of **3** (200 mg, 0.85 mmol, 1.0 equiv) in EtOH (abs.):H$_2$O (5.7 mL:2.8 mL, 0.1 M) and the reaction mixture stirred at 70 °C for 24 h. A precipitate was formed and the product was isolated using vacuum filtration to give **7k** (180 mg, 64%, 0.55 mmol) as a yellow amorphous solid.

Mp: decomposition 246 °C (recrystallised from EtOH).

NMR Spectroscopy (see spectra)**:**

^{1}**H NMR** (400 MHz, CDCl$_3$): δ_H 7.30 (s, 1H), 7.27–7.25 (m, 2H), 6.94–6.91 (m, 2H), 4.45 (ddt, *J* = 10.2, 7.7,

3.2 Hz, 1H), 3.79 (ddd, *J* =12.6, 7.5, 5.8 Hz, 1H), 3.72 (s, 2H), 3.61 (dt, *J* = 12.6, 7.2 Hz, 1H), 2.91 (dd, *J* = 18.2, 10.2 Hz, 1H), 2.84 (dd, *J* = 18.2, 3.2 Hz, 1H), 2.40 (ddt, *J* = 14.6, 7.8, 7.7 Hz, 1H), 2.10–1.97 (m, 2H), 1.89–1.81 (m, 1H);

^{13}C NMR (126 MHz, $CDCl_3$): δ_C 189.6, 158.5, 144.4, 139.5, 128.8, 126.0, 121.6, 117.8, 114.9, 53.1, 49.9, 47.0, 32.3, 23.3, 23.1.

IR (film): ν_{max} 3283, 2954, 2249, 1654, 1591, 1560, 1516 cm^{-1}.

HRMS (ESI$^+$): m/z calc'd for $C_{17}H_{17}{}^{35}ClN_3O_2$ [M+H]$^+$, 330.1004; found, 330.0998.

7-Chloro-6-((4-hydroxyphenyl)amino)-2,3,9,9a-tetrahydro-1*H*-pyrrolo[1,2-*a*]azepine-5,8-dione (7l)

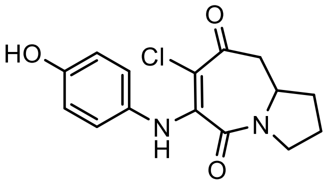

7l

4-Aminophenol (45.8 mg, 0.42 mmol, 2.1 equiv) was added to a suspension of **3** (46.8 mg, 0.20 mmol, 1.0 equiv) in EtOH (abs.):H_2O (1.4 mL:0.6 mL, 0.1 M) and the suspension was stirred at rt for 24 h. The solution was diluted with toluene (2.0 mL) and EtOH removed *in vacuo*. The reaction mixture was diluted with EtOAc (10 mL) and washed with H_2O (2 × 10 mL). The aqueous layers were combined and extracted with EtOAc (3 × 30 mL). All organic layers were combined, washed with sat. brine (100 mL), dried over $MgSO_4$, filtered and the solvent removed *in vacuo*. The crude product was purified by flash column chromatography (70:30 to 100:0, EtOAc:Pet. Ether 40-60 °C) to give **7l** (37.0 mg, 60%, 0.12 mmol) as an orange amorphous solid.

Mp: decomposition 227 °C (recrystallised from EtOH).

NMR Spectroscopy (see spectra)**:**

^{1}H NMR (400 MHz, DMSO-*d*$_6$): δ_H 9.28 (s, 1H), 8.71 (s, 1H), 6.86–6.83 (m, 2H), 6.66–6.63 (m, 2H), 4.51 (ddt, *J* = 11.5, 8.7, 1.7 Hz, 1H), 3.58 (ddd, *J* = 12.1, 8.0, 4.9 Hz, 1H), 3.26 (dt, *J* = 12.1, 7.5 Hz, 1H), 2.89 (dd, *J* = 18.6, 11.5 Hz, 1H), 2.63 (dd, *J* = 18.6, 1.7 Hz, 1H), 2.25–2.17 (m, 1H), 2.01–1.90 (m, 1H), 1.87–1.80 (m, 1H), 1.75–1.68 (m, 1H);

^{13}C NMR (101 MHz, DMSO-*d*$_6$): δ_C 189.5, 157.8, 154.3, 147.7, 132.3, 123.6, 115.0, 109.1, 52.7, 49.4, 45.6, 31.0, 22.2.

IR (film): ν_{max} 3672, 3249, 2988, 2904, 1647, 1550, 1514 cm^{-1}.

HRMS (ESI$^+$): m/z calc'd for $C_{15}H_{15}{}^{35}ClN_2NaO_3$ [M+Na]$^+$, 329.0663; found, 329.0651.

7-Chloro-6-(*m*-tolylamino)-2,3,9,9a-tetrahydro-1*H*-pyrrolo[1,2-*a*]azepine-5,8-dione (7m)

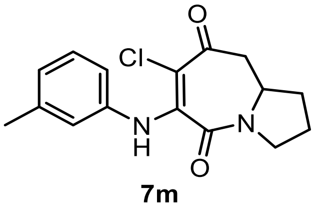

7m

Prepared following **General Procedure A**, using *m*-toluidine (550 µL, 5.1 mmol), La(OTf)$_3$ (827 mg, 1.4 mmol) and **3** (300 mg, 1.3 mmol) with a reaction time of 4 h. Purification by flash column chromatography (95:5 to 90:10, toluene:acetone) gave **7m** (344 mg, 88%, 1.1 mmol) as a yellow amorphous solid.

Mp: 149-150 °C (recrystallised from EtOH).

NMR Spectroscopy (see spectra):

^{1}**H NMR** (400 MHz, CDCl$_3$): δ_H 7.29 (s, 1H), 7.17 (t, *J* = 7.7 Hz, 1H), 6.94 (d, *J* = 7.7 Hz, 1H), 6.77 (s, 1H), 6.70 (dd, *J* = 7.7, 1.9 Hz, 1H), 4.46 (ddt, *J* = 10.5, 7.7, 2.9 Hz, 1H), 3.78 (ddd, *J* = 12.5, 7.6, 5.8 Hz, 1H), 3.59 (dt, *J* = 12.5, 7.2 Hz, 1H), 2.89 (dd, *J* = 18.4, 10.5 Hz, 1H), 2.82 (dd, *J* = 18.4, 2.9 Hz, 1H), 2.38 (ddt, *J* = 12.9, 7.7, 7.6 Hz, 1H), 2.32 (s, 3H), 2.09–1.95 (m, 2H), 1.87–1.80 (m, 1H);

^{13}**C NMR** (126 MHz, CDCl$_3$): δ_C 189.6, 158.6, 145.2, 139.4, 139.1, 128.9, 125.8, 122.0, 118.2, 113.8, 53.1, 49.9, 46.8, 32.3, 23.1, 21.6.

IR (film): ν_{max} 3301, 2924, 1654, 1608, 1560 cm^{-1}.

HRMS (ESI$^+$): m/z calc'd for $C_{16}H_{18}{}^{35}ClN_2O_2$ [M+H]$^+$, 305.1051; found, 305.1055.

7-Chloro-6-((3-isopropylphenyl)amino)-2,3,9,9a-tetrahydro-1*H*-pyrrolo[1,2-*a*]azepine-5,8-dione (7n)

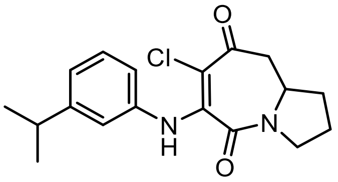

7n

Prepared following **General Procedure A**, using 3-isopropylaniline (1.23 mL, 8.5 mmol), La(OTf)$_3$ (1.33 g, 2.3 mmol) and **3** (0.500 g, 2.1 mmol) with a reaction time of 3 h. Purification by flash column chromatography (95:5, toluene:acetone) gave **7n** (0.600 g, 84%, 1.8 mmol) as a yellow solid foam.

Mp: 136-138 °C (recrystallised from EtOH).

NMR Spectroscopy (see spectra):

^{1}**H NMR** (500 MHz, CDCl$_3$): δ_H 7.32 (s, 1H), 7.19 (t, *J* = 7.7 Hz, 1H), 6.97 (d, *J* = 7.7 Hz, 1H), 6.77 (dd, *J* = 2.4, 1.8 Hz, 1H), 6.73 (dd, *J* = 7.7, 2.4 Hz, 1H), 4.49–4.44 (m, 1H), 3.74 (ddd, *J* = 12.7, 7.6, 5.9 Hz, 1H), 3.56 (dt, *J* = 12.7, 7.1 Hz, 1H), 2.89–2.77 (m, 3H), 2.36 (ddt, *J* = 12.9, 7.7, 7.6 Hz, 1H), 2.05–1.95 (m, 2H),

1.84–1.78 (m, 1H), 1.21 (d, J = 7.0 Hz, 6H);

^{13}C NMR (126 MHz, $CDCl_3$): δ_C 189.6, 158.5, 149.8, 145.3, 139.5, 128.9, 123.1, 119.1, 118.4, 113.6, 53.0, 49.9, 46.7, 33.9, 32.1, 24.0, 23.6, 23.0.

IR (film): ν_{max} 3286, 2959, 1652, 1593, 1557 cm^{-1}.

HRMS (ESI$^+$): m/z calc'd for $C_{18}H_{22}{}^{35}ClN_2O_2$ [M+H]$^+$, 333.1364; found, 333.1370.

6-((3-(*tert*-Butyl)phenyl)amino)-7-chloro-2,3,9,9a-tetrahydro-1*H*-pyrrolo[1,2-*a*]azepine-5,8-dione (7o)

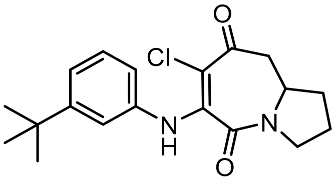

7o

Prepared following **General Procedure A**, using 3-(*tert*-butyl)aniline (1.07 mL, 6.8 mmol), La(OTf)$_3$ (1.10 g, 1.9 mmol) and **3** (0.400 g, 1.7 mmol) with a reaction time of 2 h. Purification by flash column chromatography (95:5, toluene:acetone) gave **7o** (506 mg, 85%, 1.5 mmol) as a yellow amorphous solid.

Mp: 164-165 °C (recrystallised from EtOH).

NMR Spectroscopy (see spectra):

^{1}H NMR (400 MHz, $CDCl_3$): δ_H 7.30 (s, 1H), 7.23 (t, J = 7.8 Hz, 1H), 7.15 (ddd, J = 7.8, 2.4, 1.1 Hz, 1H), 6.92 (dd, J = 2.4, 2.0 Hz, 1H), 6.75 (ddd, J = 7.8, 2.0, 1.1 Hz, 1H), 4.50 (ddt, J = 10.5, 7.7, 2.9 Hz, 1H), 3.77 (ddd, J = 12.7, 7.6, 6.0 Hz, 1H), 3.59 (dt, J = 12.7, 7.1 Hz, 1H), 2.89 (dd, J = 18.4, 10.5 Hz, 1H), 2.83 (dd, J = 18.4, 2.9 Hz, 1H), 2.39 (ddt, J = 12.9, 7.8, 7.6 Hz, 1H), 2.07–1.96 (m, 2H), 1.88–1.80 (m, 1H), 1.29 (s, 9H);

^{13}C NMR (126 MHz, $CDCl_3$): δ_C 189.6, 158.5, 152.3, 145.4, 139.3, 128.9, 122.0, 118.4, 118.2, 113.8, 53.1, 50.0, 46.7, 34.8, 32.3, 31.3, 23.1.

IR (film): ν_{max} 3294, 2961, 1654, 1560 cm^{-1}.

HRMS (ESI$^+$): m/z calc'd for $C_{19}H_{24}{}^{35}ClN_2O_2$ [M+H]$^+$, 347.1521; found, 347.1527.

7-Chloro-6-((3-methoxyphenyl)amino)-2,3,9,9a-tetrahydro-1*H*-pyrrolo[1,2-*a*]azepine-5,8-dione (7p)

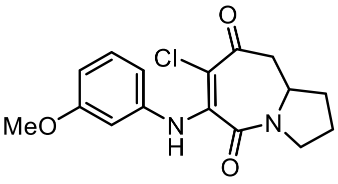

7p

Prepared following **General Procedure A**, using *m*-anisidine (1.44 mL, 12.8 mmol), $La(OTf)_3$ (2.05 g, 3.5 mmol) and **3** (0.750 g, 3.2 mmol) with a reaction time of 6 h. Purification by flash column chromatography (90:10, toluene:acetone) gave **7p** (0.813 g, 79%, 2.5 mmol) as a yellow solid foam.

Mp: 61-63 °C (recrystallised from EtOH).

NMR Spectroscopy (see spectra):

^{1}**H NMR** (400 MHz, $CDCl_3$): δ_H 7.19 (t, *J* = 8.1 Hz, 1H), 6.67 (dd, *J* = 8.1, 2.4 Hz, 1H), 6.52–6.48 (m, 2H), 4.46 (ddt, *J* = 10.6, 7.4, 2.8 Hz, 1H), 3.82–3.76 (m, 4H), 3.60 (dt, *J* = 12.5, 7.2 Hz, 1H), 2.89 (dd, *J* = 18.5, 10.6 Hz, 1H), 2.82 (dd, *J* = 18.4, 2.8 Hz, 1H), 2.41–2.34 (m, 1H), 2.09–1.95 (m, 2H), 1.87–1.80 (m, 1H);

^{13}**C NMR** (126 MHz, $CDCl_3$): δ_C 189.7, 160.3, 158.6, 145.0, 140.8, 129.9, 114.4, 113.6, 110.2, 107.4, 55.4, 53.1, 50.0, 46.8, 32.2, 23.1.

IR (film): ν_{max} 3293, 2955, 1654, 1599,1559 cm^{-1}.

HRMS (ESI$^+$): m/z calc'd for $C_{16}H_{18}{}^{35}ClN_2O_3$ $[M+H]^+$, 321.1006; found, 321.1009.

3-((7-Chloro-5,8-dioxo-2,3,5,8,9,9a-hexahydro-1*H*-pyrrolo[1,2-*a*]azepin-6-yl)amino)benzonitrile (7q)

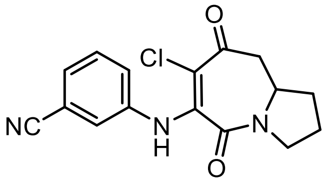

7q

Prepared following **General Procedure A**, using 3-aminobenzonitrile (2.02 g, 17.1 mmol), $La(OTf)_3$ (2.75 g, 4.7 mmol) and **3** (1.00 g, 4.3 mmol) with a reaction time of 72 h. Purification by flash column chromatography (95:5 to 90:10, toluene:acetone) gave **7q** (0.334 g, 25%, 1.1 mmol) as a pale yellow amorphous solid.

Mp: 219 °C (recrystallised from EtOH).

NMR Spectroscopy (see spectra):

^{1}**H NMR** (400 MHz, $CDCl_3$): δ_H 7.43–7.36 (m, 2H), 7.33 (s, 1H), 7.15–7.13 (m, 1H), 7.12–7.11 (m, 1H), 4.46 (ddt, *J* = 8.5, 8.3, 4.1 Hz, 1H), 3.79 (ddd, *J* = 12.7, 7.1, 5.9 Hz, 1H), 3.65 (dt, *J* = 12.7, 7.2 Hz, 1H), 2.97–2.86 (m, 2H), 2.45–2.38 (m, 1H), 2.11–2.01 (m, 2H), 1.91–1.83 (m, 1H);

^{13}**C NMR** (126 MHz, $CDCl_3$): δ_C 189.6, 158.5, 143.1, 140.5, 130.0, 127.7, 125.1, 123.7, 118.4, 116.7, 113.2,

53.1, 49.8, 47.2, 32.3, 23.1.

IR (film): ν_{max} 3290, 2967, 2230, 1651, 1562 cm^{-1}.

HRMS (ESI$^+$): m/z calc'd for $C_{16}H_{15}{}^{35}ClN_3O_2$ [M+H]$^+$, 316.0853; found, 316.0846.

7-Chloro-6-((2-methoxyphenyl)amino)-2,3,9,9a-tetrahydro-1*H*-pyrrolo[1,2-*a*]azepine-5,8-dione (7r)

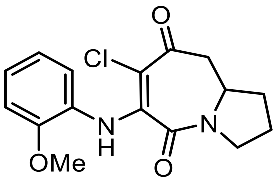

7r

Prepared following **General Procedure A**, using *o*-anisidine (386 µL, 3.4 mmol), La(OTf)$_3$ (554 mg, 0.94 mmol) and **3** (200 mg, 0.85 mmol) with a reaction time of 30 h. Purification by flash column chromatography (95:5 to 90:10, toluene:acetone) gave **7r** (187 mg, 68%, 0.58 mmol) as a yellow amorphous solid.

Mp: 169-170 °C (recrystallised from EtOH).

NMR Spectroscopy (see spectra)**:**

^{1}**H NMR** (400 MHz, $CDCl_3$): δ_H 7.47 (s, 1H), 7.10–7.06 (m, 1H), 6.90–6.86 (m, 2H), 6.81 (dd, *J* = 8.1, 1.6 Hz, 1H), 4.46 (ddt, *J* = 10.9, 7.8, 2.6 Hz, 1H), 3.86 (s, 3H), 3.79 (ddd, *J* = 12.6, 7.7, 5.5 Hz, 1H), 3.60 (dt, *J* = 12.6, 7.4 Hz, 1H), 2.89 (dd, *J* = 18.4, 10.9 Hz, 1H), 2.81 (dd, *J* = 18.4, 2.6 Hz, 1H), 2.41–2.31 (m, 1H), 2.09–1.95 (m, 2H), 1.87–1.80 (m, 1H);

^{13}**C NMR** (126 MHz, $CDCl_3$): δ_C 189.6, 158.5, 150.6, 145.3, 128.8, 125.2, 120.9, 120.7, 113.6, 111.1, 55.8, 53.0, 50.0, 46.7, 32.2, 23.1.

IR (film): ν_{max} 3329, 2926, 1654, 1598, 1557 cm^{-1}.

HRMS (ESI$^+$): m/z calc'd for $C_{16}H_{18}{}^{35}ClN_2O_3$ [M+H]$^+$, 321.1000; found, 321.0995.

2.4. Visible Light Photoredox Catalysis Reaction

2.4.1. General Procedure B

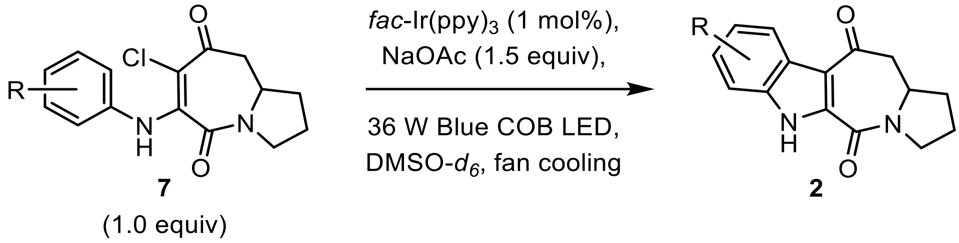

Specified starting material (0.20 mmol, 1.0 equiv), NaOAc (24.6 mg, 0.30 mmol, 1.5 equiv) and *fac*-Ir(ppy)$_3$ (1.3 mg, 0.002 mmol, 1.0 mol%) were suspended in DMSO-*d*$_6$ (2.0 mL, 0.1 M). The reaction mixture was purged by bubbling N_2 gas through the suspension for 10 minutes. The reaction mixture was fan cooled and irradiated with blue light (36 W COB LED) under nitrogen for the stated time. The solvent was removed *in vacuo* and the crude solid was suspended in H_2O (2.0 mL). The suspension was sonicated for 5 minutes and the solid separated from aqueous liquid *via* centrifugation (7000 rpm, 10 minutes). The majority of the aqueous liquid was removed from the solid using a needle and syringe. The process was repeated a further two times, starting from the suspension of the solid in water. The solid was then resuspended in acetone (0.5 mL) and the suspension was sonicated for 5 minutes. The solid was separated from the organic solvent *via* centrifugation (7000 rpm, 10 minutes) and the majority of the organic solvent was removed from the solid using a needle and syringe. The process was repeated a further two times, starting from the suspension of the solid in acetone. The solid was resuspended in EtOH (1.0 mL), the suspension was sonicated for 5 minutes and EtOH was then removed *in vacuo*. The process was repeated a further two times, starting from the suspension of the solid in EtOH to give purified product.

2.4.2. General Procedure C

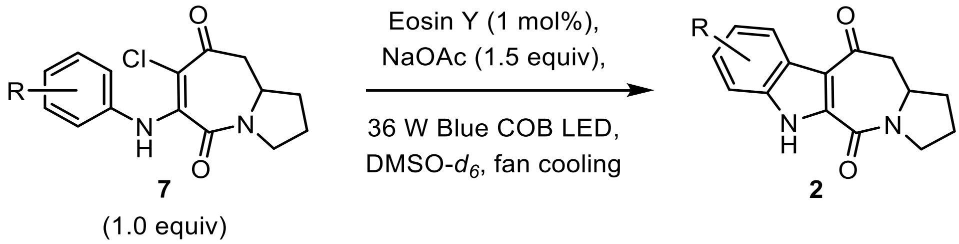

Specified starting material (0.20 mmol, 1.0 equiv), NaOAc (24.6 mg, 0.30 mmol, 1.5 equiv) and eosin Y (1.3 mg, 0.002 mmol, 1.0 mol%) were suspended in DMSO-*d*$_6$ (2.0 mL, 0.1 M). The reaction mixture was purged by bubbling N_2 gas through the suspension for 10 minutes. The reaction mixture was fan cooled and irradiated with blue light (36 W COB LED) under nitrogen for the stated time. The solvent was removed *in vacuo* and the crude solid was suspended in H_2O (2.0 mL). The suspension was sonicated for 5 minutes and the solid separated from aqueous liquid *via* centrifugation (7000 rpm, 10 minutes). The majority of the aqueous liquid was removed from the solid using a needle and syringe. The process was repeated a further two times, starting from the suspension of the solid in water. The solid was then resuspended in acetone (0.5 mL) and the suspension was

sonicated for 5 minutes. The solid was separated from the organic solvent *via* centrifugation (7000 rpm, 10 minutes) and the majority of the organic solvent was removed from the solid using a needle and syringe. The process was repeated a further two times, starting from the suspension of the solid in acetone. The solid was resuspended in EtOH (1.0 mL), the suspension was sonicated for 5 minutes and EtOH was then removed *in vacuo*. The process was repeated a further two times, starting from the suspension of the solid in EtOH to give purified product.

2.5. Synthesis of Indoloazepinones

2,3,12,12a-Tetrahydropyrrolo[1',2':1,7]azepino[3,4-*b*]indole-5,11(1*H*,6*H*)-dione (2a)

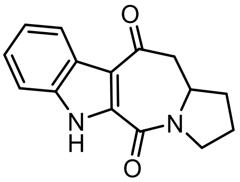

2a

Procedure 1

Prepared following **General Procedure B**, using **7a** (58.2 mg) with a reaction time of 1 h, gave **2a** (43.0 mg, 85%, 0.17 mmol) as a cream amorphous solid.

Procedure 2

Prepared following **General Procedure C**, using **7a** (58.2 mg) with a reaction time of 2 h, gave **2a** (37.0 mg, 73%, 0.15 mmol) as a pale pink amorphous solid.

Procedure 3 – 1 mmol Scale

7a (291 mg, 1.0 mmol, 1.0 equiv), NaOAc (123 mg, 1.5 mmol, 1.5 equiv) and *fac*-Ir(ppy)$_3$ (6.5 mg, 0.01 mmol, 1.0 mol%) were suspended in DMSO-*d*$_6$ (10 mL). The reaction mixture was purged by bubbling N_2 gas through the suspension for 45 minutes. The reaction mixture was fan cooled and irradiated with blue light (36 W COB LED) under nitrogen for the 1 h. N_2 gas was continually bubbled through the reaction mixture for the duration of the reaction. The solvent was removed *in vacuo* and the crude solid was suspended in H_2O (10 mL). The suspension was sonicated for 5 minutes and the solid separated from aqueous liquid *via* centrifugation (7000 rpm, 10 minutes). The majority of the aqueous liquid was removed from the solid using a needle and syringe. The process was repeated a further two times, starting from the suspension of the solid in water. The solid was then resuspended in acetone (2.5 mL) and the suspension was sonicated for 5 minutes. The solid was separated from the organic solvent *via* centrifugation (7000 rpm, 10 minutes) and the majority of the organic solvent was removed from the solid using a needle and syringe. The process was repeated a further two times, starting from the suspension of the solid in acetone. The solid was resuspended in EtOH (5 mL), the suspension was sonicated for 5 minutes and EtOH was then removed *in vacuo*. The process was repeated a further two times, starting from the suspension of the solid in EtOH, giving **2a** (213 mg, 84%, 0.84 mmol) as a cream amorphous solid.

Mp: decomposition 286 °C.

NMR Spectroscopy (see spectra)**:**

^{1}**H NMR** (400 MHz, DMSO-*d*$_6$): δ_H 12.47 (s, 1H), 8.29 (d, *J* = 8.0 Hz, 1H), 7.53 (d, *J* = 8.2 Hz, 1H), 7.32 (td, *J* = 8.2, 1.4 Hz, 1H), 7.25 (td, *J* = 8.0, 1.2 Hz, 1H), 4.26–4.19 (m, 1H), 3.92–3.86 (m, 1H), 3.59–3.52 (m, 1H), 3.11 (dd, *J* = 17.2, 11.6 Hz, 1H), 2.72 (d, *J* = 17.2 Hz, 1H), 2.31–2.26 (m, 1H), 1.97–1.93 (m, 1H), 1.85–1.76 (m, 2H);

^{13}**C NMR** (151 MHz, DMSO-*d*$_6$): δ_C 193.8, 158.7, 135.40, 135.38, 126.2, 124.9, 122.81, 122.76, 113.7,

112.7, 53.8, 48.9, 47.5, 33.5, 22.6.

IR (film): ν_{max} 3176, 2951, 1636, 1615, 1575 cm^{-1}.

HRMS (ESI$^+$): m/z calc'd for $C_{15}H_{14}N_2NaO_2$ [M+Na]$^+$, 277.0947; found, 277.0947.

9-Methyl-2,3,12,12a-tetrahydropyrrolo[1',2':1,7]azepino[3,4-*b*]indole-5,11(1*H*,6*H*)-dione (2b)

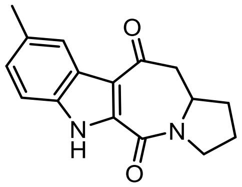

2b

Procedure 1

Prepared following **General Procedure B**, using **7b** (61.0 mg) with a reaction time of 1 h, gave **2b** (39.0 mg, 67%, 0.13 mmol) as a cream amorphous solid.

Procedure 2

Prepared following **General Procedure C**, using **7b** (61.0 mg) with a reaction time of 3 h, gave **2b** (35.0 mg, 65%, 0.12 mmol) as a pale pink amorphous solid.

Mp: decomposition 279 °C.

NMR Spectroscopy (see spectra):

^{1}**H NMR** (500 MHz, DMSO-d_6): δ_H 12.36 (s, 1H), 8.11–8.10 (m, 1H), 7.41 (d, J = 8.3 Hz, 1H), 7.15 (dd, J = 8.3, 1.7 Hz, 1H), 4.23–4.17 (m, 1H), 3.90–3.86 (m, 1H), 3.57–3.52 (m, 1H), 3.08 (dd, J = 17.2, 11.5 Hz, 1H), 2.71 (d, J = 17.2 Hz, 1H), 2.41 (s, 3H), 2.31–2.26 (m, 1H), 1.97–1.91 (m, 1H), 1.83–1.74 (m, 2H);

^{13}**C NMR** (126 MHz, DMSO-d_6): δ_C 193.7, 158.7, 135.3, 133.8, 131.8, 126.6, 126.5, 122.2, 113.3, 112.4, 53.8, 48.9, 47.5, 33.5, 22.6, 21.4.

IR (film): ν_{max} 3212, 2951, 1651, 1615, 1583 cm^{-1}.

HRMS (ESI$^+$): m/z calc'd for $C_{16}H_{17}N_2O_2$ [M+H]$^+$, 269.1285; found, 269.1284.

9-Methoxy-2,3,12,12a-tetrahydropyrrolo[1',2':1,7]azepino[3,4-*b*]indole-5,11(1*H*,6*H*)-dione (2c)

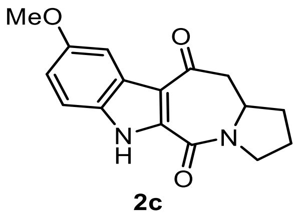

2c

Procedure 1

Prepared following **General Procedure B**, using **7c** (64.2 mg) with a reaction time of 3 h, gave **2c** (35.0 mg, 62%, 0.12 mmol) as a cream amorphous solid.

Procedure 2

Prepared following **General Procedure C**, using **7c** (64.2 mg) with a reaction time of 6 h, gave **2c** (33.0 mg, 58%, 0.12 mmol) as a pale pink amorphous solid.

Mp: decomposition 267 °C.

NMR Spectroscopy (see spectra)**:**

^{1}**H NMR** (500 MHz, DMSO-d_6): δ_H 12.37 (s, 1H), 7.78 (d, *J* = 2.5 Hz, 1H), 7.43 (dd, *J* = 8.9, 0.6 Hz, 1H), 6.96 (dd, *J* = 8.9, 2.6 Hz, 1H), 4.23–4.18 (m, 1H), 3.90–3.86 (m, 1H), 3.79 (s, 3H), 3.57–3.52 (m, 1H), 3.09 (dd, *J* = 17.2, 11.6 Hz, 1H), 2.71 (d, *J* = 17.2 Hz, 1H), 2.30–2.26 (m, 1H), 1.97–1.92 (m, 1H), 1.82–1.75 (m, 2H);

^{13}**C NMR** (126 MHz, DMSO-d_6): δ_C 193.7, 158.7, 156.1, 135.3, 130.4, 127.0, 115.7, 113.7, 113.5, 103.2, 55.3, 53.8, 48.9, 47.5, 33.6, 22.6.

IR (film): ν_{max} 3184, 2978, 1643, 1612, 1523 cm^{-1}.

HRMS (ESI$^+$): m/z calc'd for $C_{16}H_{17}N_2O_3$ [M+H]$^+$, 286.1234; found, 286.1240.

9-Fluoro-2,3,12,12a-tetrahydropyrrolo[1',2':1,7]azepino[3,4-*b*]indole-5,11(1*H*,6*H*)-dione (2d)

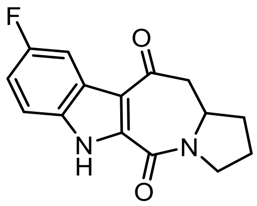

2d

Procedure 1

Prepared following **General Procedure B**, using **7d** (61.7 mg) with a reaction time of 1 h, gave **2d** (43.0 mg, 79%, 0.16 mmol) as a cream amorphous solid.

Procedure 2

Prepared following **General Procedure C**, using **7d** (61.7 mg) with a reaction time of 2 h, gave **2d** (39.0 mg, 72%, 0.14 mmol) as a pale pink amorphous solid.

Mp: decomposition 277 °C.

NMR Spectroscopy (see spectra):

^{1}**H NMR** (400 MHz, DMSO-d_6): δ_H 12.60 (s, 1H), 7.95 (dd, *J* = 9.9, 2.6 Hz, 1H), 7.54 (dd, *J* = 8.9, 4.7 Hz, 1H), 7.20 (ddd, *J* = 9.9, 8.9, 2.6 Hz, 1H), 4.26–4.19 (m, 1H), 3.91–3.87 (m, 1H), 3.58–3.52 (m, 1H), 3.11 (dd, *J* = 17.1, 11.6 Hz, 1H), 2.72 (d, *J* = 17.2 Hz, 1H), 2.31–2.26 (m, 1H), 1.96–1.92 (m, 1H), 1.84–1.73 (m, 2H);

^{19}**F NMR,** ^{1}H decoupled (377 MHz, DMSO-d_6): δ_F −119.8;

^{13}**C NMR** (126 MHz, DMSO-d_6): δ_C 193.8, 158.4, 158.3 (d, *J* = 236.6 Hz), 136.8, 132.1, 126.6 (d, *J* = 11.2 Hz), 114.4 (d, *J* = 9.9 Hz), 113.8, 113.7 (d, *J* = 32.6 Hz), 107.2 (d, *J* = 24.6 Hz), 53.8, 48.7, 47.6, 33.5, 22.6.

IR (film): ν_{max} 3168, 2952, 1637, 1619, 1583, 1526 cm^{-1}.

HRMS (ESI$^+$): m/z calc'd for $C_{15}H_{14}FN_2O_2$ [M+H]$^+$, 273.1034; found, 273.1039.

9-Bromo-2,3,12,12a-tetrahydropyrrolo[1',2':1,7]azepino[3,4-*b*]indole-5,11(1*H*,6*H*)-dione (2e)

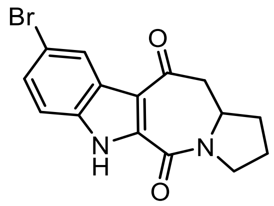

2e

Prepared following **General Procedure B**, using **7e** (73.9 mg) with a reaction time of 1 h, gave **2e** (54.0 mg, 81%, 0.16 mmol) as a cream amorphous solid.

Mp: decomposition 297 °C.

NMR Spectroscopy (see spectra):

^{1}**H NMR** (400 MHz, DMSO-d_6): δ_H 12.68 (s, 1H), 8.43 (d, *J* = 1.8 Hz, 1H), 7.50 (d, *J* = 8.7 Hz, 1H), 7.46 (dd, *J* = 8.7, 1.8 Hz, 1H), 4.26–4.20 (m, 1H), 3.91–3.86 (m, 1H), 3.59–3.52 (m, 1H), 3.12 (dd, *J* = 17.2, 11.6 Hz, 1H), 2.73 (d, *J* = 17.2 Hz, 1H), 2.32–2.26 (m, 1H), 1.97–1.92 (m, 1H), 1.85–1.76 (m, 2H);

^{13}**C NMR** (126 MHz, DMSO-d_6): δ_C 193.7, 158.3, 136.4, 134.1, 127.8, 127.6, 124.7, 115.5, 114.9, 113.1, 53.7, 48.7, 47.6, 33.5, 22.6.

IR (film): ν_{max} 3231, 2969, 1649, 1624, 1528 cm^{-1}.

HRMS (ESI$^+$): m/z calc'd for $C_{15}H_{14}{}^{79}BrN_2O_2$ [M+H]$^+$, 333.0233; found, 333.0231.

9-(4,4,5,5-Tetramethyl-1,3,2-dioxaborolan-2-yl)-2,3,12,12a-tetrahydropyrrolo[1',2':1,7]azepino[3,4-*b*]indole-5,11(1*H*,6*H*)-dione (2f)

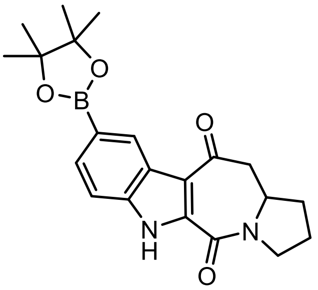

2f

Prepared following **General Procedure B**, using **7f** (83.3 mg) with a reaction time of 2 h, gave **2f** (49.0 mg, 64%, 0.13 mmol) as a cream amorphous solid.

Mp: decomposition 296 °C.

NMR Spectroscopy (see spectra)**:**

^{1}**H NMR** (400 MHz, DMSO-*d*$_6$): δ_H 12.56 (s, 1H), 8.72 (s, 1H), 7.60 (dd, *J* = 8.3, 1.1 Hz, 1H), 7.51 (d, *J* = 8.3 Hz, 1H), 4.26–4.19 (m, 1H), 3.91–3.86 (m, 1H), 3.59–3.52 (m, 1H), 3.11 (dd, *J* = 17.2, 11.6 Hz, 1H), 2.75 (d, *J* = 17.2 Hz, 1H), 2.33–2.27 (m, 1H), 1.97–1.93 (m, 1H), 1.82–1.76 (m, 2H), 1.32 (s, 12H);

^{11}**B NMR** (128 MHz, DMSO-*d*$_6$): δ_B 33.8;

^{13}**C NMR** (126 MHz, DMSO-*d*$_6$): δ_C 193.9, 158.6, 137.3, 135.8, 130.6, 130.4, 125.8, 113.9, 112.2, 83.5, 53.8, 48.9, 47.6, 33.5, 24.74, 24.71, 22.6. The carbon attached to boron was not observed due to quadrupolar relaxation.

IR (film): ν_{max} 3231, 2976, 1652, 1613, 1576 cm^{-1}.

HRMS (ESI$^+$): m/z calc'd for $C_{21}H_{25}{}^{11}BN_2O_4$ [M+H]$^+$, 381.1980; found, 381.1990.

***N*-(5,11-Dioxo-1,2,3,5,6,11,12,12a-octahydropyrrolo[1',2':1,7]azepino[3,4-*b*]indol-9-yl)acetamide (2g)**

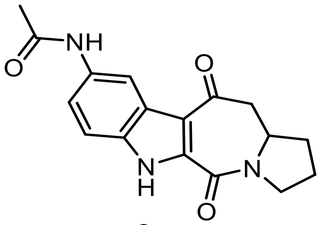

2g

Procedure 1

Prepared following **General Procedure B**, using **7g** (69.6 mg) with a reaction time of 3 h, gave **2g** (29.0 mg, 47%, 0.09 mmol) as a cream amorphous solid.

Procedure 2

Prepared following **General Procedure C**, using **7g** (64.2 mg) with a reaction time of 4 h, gave **2g** (39.0 mg, 66%, 0.13 mmol) as a pale pink amorphous solid.

Mp: decomposition 297 °C.

NMR Spectroscopy (see spectra):

^{1}**H NMR** (400 MHz, DMSO-*d*$_6$): δ_H 12.40 (s, 1H), 9.96 (s, 1H), 8.48 (d, *J* = 2.1 Hz, 1H), 7.62 (dd, *J* = 8.9, 2.1 Hz, 1H), 7.43 (d, *J* = 8.9 Hz, 1H), 4.24–4.18 (m, 1H), 3.91–3.85 (m, 1H), 3.58–3.51 (m, 1H), 3.09 (dd, *J* = 17.1, 11.6 Hz, 1H), 2.71 (d, *J* = 17.2 Hz, 1H), 2.30–2.26 (m, 1H), 2.04 (s, 3H), 1.96–1.92 (m, 1H), 1.81–1.76 (m, 2H);

^{13}**C NMR** (126 MHz, DMSO-*d*$_6$): δ_C 193.5, 167.9, 158.7, 135.6, 135.0, 131.9, 126.3, 118.5, 113.6, 112.54, 112.48, 53.7, 48.9, 47.5, 33.5, 23.9, 22.6.

IR (film): ν_{max} 3338, 3213, 2978, 1693, 1649, 1613, 1557, 1522 cm^{-1}.

HRMS (ESI$^+$): m/z calc'd for $C_{17}H_{17}N_3NaO_3$ [M+Na]$^+$, 334.1168; found, 334.1161.

9-Acetyl-2,3,12,12a-tetrahydropyrrolo[1',2':1,7]azepino[3,4-*b*]indole-5,11(1*H*,6*H*)-dione (2h)

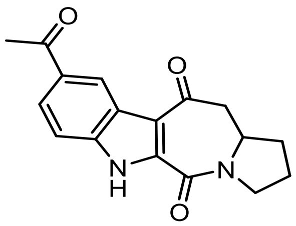

2h

Prepared following **General Procedure B**, using **7h** (66.6 mg) with a reaction time of 5 h, gave **2h** (47.0 mg, 79%, 0.16 mmol) as a cream amorphous solid.

Mp: decomposition 290 °C.

NMR Spectroscopy (see spectra):

^{1}**H NMR** (400 MHz, DMSO-*d*$_6$): δ_H 12.77 (s, 1H), 8.94 (d, *J* = 1.7 Hz, 1H), 7.93 (dd, *J* = 8.7, 1.7 Hz, 1H), 7.60 (d, *J* = 8.7 Hz, 1H), 4.29–4.22 (m, 1H), 3.92–3.86 (m, 1H), 3.60–3.53 (m, 1H), 3.16 (dd, *J* = 17.2, 11.6 Hz, 1H), 2.76 (d, *J* = 17.2 Hz, 1H), 2.63 (s, 3H), 2.33–2.27 (m, 1H), 1.98–1.93 (m, 1H), 1.86–1.75 (m, 2H);

^{13}**C NMR** (126 MHz, DMSO-*d*$_6$): δ_C 197.4, 193.9, 158.3, 137.9, 137.1, 132.0, 125.7, 124.69, 124.66, 114.7, 112.8, 53.8, 48.8, 47.6, 33.5, 26.7, 22.6.

IR (film): ν_{max} 3185, 2973, 1670, 1628, 1577, 1529 cm^{-1}.

HRMS (ESI$^+$): m/z calc'd for $C_{17}H_{17}N_2O_3$ [M+H]$^+$, 297.1234; found, 297.1238.

Methyl-5,11-dioxo-1,2,3,5,6,11,12,12a-octahydropyrrolo[1',2':1,7]azepino[3,4-*b*]indole-9-carboxylate (2i)

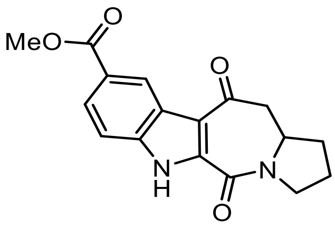

2i

Prepared following **General Procedure B**, using **7i** (69.8 mg) with a reaction time of 3 h, gave **2i** (34.0 mg, 54%, 0.11 mmol) as a cream amorphous solid.

Mp: decomposition 297 °C.

NMR Spectroscopy (see spectra)**:**

^{1}**H NMR** (500 MHz, DMSO-d_6): δ_H 12.78 (s, 1H), 8.98 (d, *J* = 1.7 Hz, 1H), 7.92 (dd, *J* = 8.7, 1.7 Hz, 1H), 7.61 (dd, J = 8.7, 0.8 Hz, 1H), 4.28–4.23 (m, 1H), 3.91–3.87 (m, 4H), 3.59–3.54 (m, 1H), 3.15 (dd, *J* = 17.3, 11.7 Hz, 1H), 2.76 (d, *J* = 17.3 Hz, 1H), 2.32–2.27 (m, 1H), 1.98–1.93 (m, 1H), 1.85–1.75 (m, 2H);

^{13}**C NMR** (126 MHz, DMSO-d_6): δ_C 193.9, 166.7, 158.3, 137.9, 137.1, 125.7, 125.5, 125.3, 124.1, 114.4, 112.9, 53.7, 52.0, 48.8, 47.6, 33.5, 22.6.

IR (film): ν_{max} 3222, 2950, 1715, 1650, 1612, 1582, 1529 cm^{-1}.

HRMS (ESI$^+$): m/z calc'd for $C_{17}H_{17}N_2O_4$ [M+H]$^+$, 313.1183; found, 313.1188.

5,11-Dioxo-1,2,3,5,6,11,12,12a-octahydropyrrolo[1',2':1,7]azepino[3,4-*b*]indole-9-carbonitrile (2j)

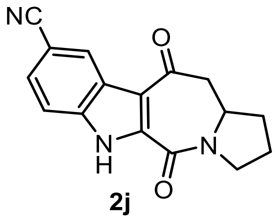

2j

Prepared following **General Procedure B**, using **7j** (55.9 mg) with a reaction time of 4 h, gave **2j** (34.0 mg, 61%, 0.12 mmol) as a cream amorphous solid.

Mp: decomposition 297 °C.

NMR Spectroscopy (see spectra)**:**

^{1}**H NMR** (400 MHz, DMSO-d_6): δ_H 12.93 (s, br, 1H), 8.66 (dd, *J* = 1.4, 1.2 Hz, 1H), 7.69 (m, 2H), 4.30–4.24 (m, 1H), 3.92–3.87 (m, 1H), 3.60–3.53 (m, 1H), 3.17 (dd, *J* = 17.3, 11.6 Hz, 1H), 2.78 (d, *J* = 17.3 Hz, 1H), 2.33–2.27 (m, 1H), 1.99–1.94 (m, 1H), 1.84–1.76 (m, 2H);

^{13}**C NMR** (126 MHz, DMSO-d_6): δ_C 193.8, 158.1, 137.6, 137.2, 128.1, 127.4, 125.8, 119.9, 114.3, 113.8, 105.0, 53.7, 48.6, 47.7, 33.4, 22.6.

IR (film): ν_{max} 3204, 2978, 2217, 1649, 1625, 1613, 1578, 1535 cm^{-1}.

HRMS (ESI$^+$): m/z calc'd for $C_{16}H_{14}N_3O_2$ $[M+H]^+$, 280.1081; found, 280.1084.

2-(5,11-Dioxo-1,2,3,5,6,11,12,12a-octahydropyrrolo[1',2':1,7]azepino[3,4-*b*]indol-9-yl)acetonitrile (2k)

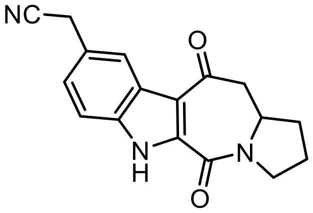

2k

Prepared following **General Procedure B**, using **7k** (66.0 mg) with a reaction time of 2 h, gave **2k** (43.0 mg, 73%, 0.15 mmol) as a cream amorphous solid.

Mp: decomposition 290 °C.

NMR Spectroscopy (see spectra):

^{1}**H NMR** (500 MHz, DMSO-d_6): δ_H 12.55 (s, 1H), 8.32 (s, 1H), 7.54 (d, *J* = 8.4 Hz, 1H), 7.29 (dd, *J* = 8.4, 1.9 Hz, 1H), 4.25–4.20 (m, 1H), 4.14 (s, 2H), 3.91–3.86 (m, 1H), 3.58–3.53 (m, 1H), 3.12 (dd, *J* = 17.3, 11.6 Hz, 1H), 2.74 (d, *J* = 17.3 Hz, 1H), 2.32–2.27 (m, 1H), 1.97–1.93 (m, 1H), 1.84–1.75 (m, 2H);

^{13}**C NMR** (126 MHz, DMSO-d_6): δ_C 193.8, 158.5, 136.1, 134.7, 126.4, 125.7, 125.2, 122.1, 119.7, 113.5, 113.3, 53.8, 48.8, 47.6, 33.5, 22.59, 22.56.

IR (film): ν_{max} 3240, 2978, 2882, 2950, 1651, 1596, 1559, 1522 cm^{-1}.

HRMS (ESI$^+$): m/z calc'd for $C_{17}H_{16}N_3O_2$ $[M+H]^+$, 294.1237; found, 294.1244.

6-((4-Hydroxyphenyl)amino)-2,3,9,9a-tetrahydro-1*H*-pyrrolo[1,2-*a*]azepine-5,8-dione (7l')

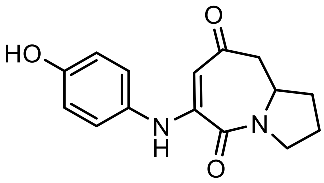

7l'

7l (61.4 mg, 0.20 mmol, 1.0 equiv), NaOAc (24.6 mg, 0.30 mmol, 1.5 equiv) and *fac*-Ir(ppy)$_3$ (1.3 mg, 0.002 mmol, 1.0 mol%) were suspended in DMSO-d_6 (2.0 mL). Oxygen was removed from the reaction mixture by bubbling N_2 gas through the suspension for 10 minutes. The reaction mixture was stirred under nitrogen and irradiated with blue light (36 W COB LED) for 1 h. The crude product was purified by flash column chromatography (EtOAc 100%) to give **7l'** (20.0 mg, 37%, 0.07 mmol) as a yellow amorphous solid.

Mp: 222 °C (recrystallised from EtOH).

NMR Spectroscopy (see spectra):

^{1}**H NMR** (400 MHz, $CDCl_3$): δ_H 7.63 (s, 1H), 7.06 (s, 1H), 6.99–6.96 (m, 2H), 6.79–6.75 (m, 2H), 5.79 (d, *J* = 1.5 Hz, 1H), 4.27–4.21 (m, 1H), 3.95–3.88 (m, 1H), 3.77–3.70 (m, 1H), 2.77 (dd, *J* = 17.7, 11.4 Hz, 1H), 2.68 (ddd, *J* = 17.7, 2.0, 1.5 Hz, 1H), 2.37–2.31 (m, 1H), 2.06–2.01 (m, 1H), 1.96–1.79 (m, 2H);

^{13}**C NMR** (151 MHz, $CDCl_3$): δ_C 196.9, 161.0, 154.9, 148.1, 129.2, 126.3, 116.2, 101.3, 53.9, 48.8, 48.0, 33.3, 22.9.

IR (film): ν_{max} 3278, 2935, 1638, 1574, 1515 cm^{-1}.

HRMS (ESI$^+$): m/z calc'd for $C_{15}H_{17}N_2O_3$ [M+H]$^+$, 273.1239; found, 273.1232.

10-Methyl-2,3,12,12a-tetrahydropyrrolo[1',2':1,7]azepino[3,4-*b*]indole-5,11(1*H*,6*H*)-dione and 8-methyl-2,3,12,12a-tetrahydropyrrolo[1',2':1,7]azepino[3,4-*b*]indole-5,11(1*H*,6*H*)-dione (2m)

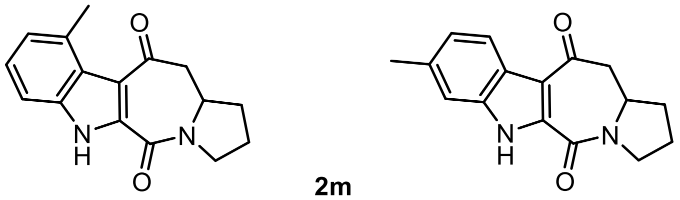

2m

3.5:1 r.r.

7m (61.0 mg, 0.20 mmol, 1.0 equiv), NaOAc (24.6 mg, 0.30 mmol, 1.5 equiv) and *fac*-Ir(ppy)$_3$ (1.3 mg, 0.002 mmol, 1.0 mol%) were suspended in DMSO-d_6 (2.0 mL). Oxygen was removed from the reaction mixture by bubbling N_2 gas through the suspension for 10 minutes. The reaction mixture was stirred under nitrogen and irradiated with blue light (36 W COB LED) for 2 h. The solvent was removed *in vacuo* and the crude solid purified by flash column chromatography (toluene:acetone 90:10). After column purification, an unknown aliphatic impurity appeared in the ^{1}H-NMR spectra. The source of the impurity could not be determined. The isolated solid was suspended in hexane (1.0 mL × 3), sonicated for 5 minutes and the hexane removed with needle and syringe to give a mixture of regioisomers **2m** *3.5:1 r.r.* (4:1, 41.0 mg, 76%, 0.15 mmol) as a cream amorphous solid.

Mp: decomposition 258 °C.

NMR Spectroscopy (see spectra):

^{1}**H NMR** (400 MHz, DMSO-d_6): δ_H 12.43 (s, 0.7H), 12.35 (s, 0.2H), 8.16 (d, *J* = 8.3 Hz, 0.2H), 7.36 (d, *J* = 8.1 Hz, 0.8H), 7.31 (s, 0.2H), 7.20 (t, *J* = 7.8 Hz, 0.8H), 7.08 (d, *J* = 8.3 Hz, 0.2H), 6.98 (d, *J* = 7.1 Hz, 0.8H), 4.34–4.28 (m, 0.8H), 4.23–4.17 (m, 0.2H), 3.91–3.86 (m, 0.2H), 3.80–3.74 (m, 0.8H), 3.61–3.58 (m, 1H), 3.08 (dd, *J* = 17.2, 11.6 Hz, 0.2H), 2.98 (dd, *J* = 18.4, 11.5 Hz, 0.8H), 2.87 (dd, *J* = 18.4, 2.1 Hz, 0.8H), 2.70 (d, *J* = 17.2 Hz, 0.2H), 2.58 (s, 2.3H), 2.41 (s, 0.7H), 2.29–2.21 (m, 1H), 2.00–1.91 (m, 1H), 1.86–1.73 (m, 2H);

^{13}**C NMR** (151 MHz, DMSO-d_6): δ_C [*a = major regioisomer; b = minor regioisomer*] 193.7 (b), 193.5 (a), 159.0 (a), 158.8 (b), 136.3 (a), 135.8 (b), 135.2 (a), 134.9 (b), 134.4 (b), 132.8 (a), 124.9 (a), 124.9 (a),

124.7 (b), 124.2 (a), 124.1 (b), 122.4 (b), 117.2 (a), 113.8 (b), 112.2 (b), 110.4 (a), 53.7 (b), 53.7 (a), 50.3 (a), 48.8 (b), 47.5 (b), 47.0 (a), 33.5 (b), 32.3 (a), 22.8 (a), 22.6 (b), 22.4 (a), 21.4 (b).

IR (film): v_{max} 3230, 2961, 1617, 1580, 1506 cm^{-1}.

HRMS (ESI$^+$): m/z calc'd for $C_{16}H_{17}N_2O_2$ [M+H]$^+$, 269.1290; found, 269.1293.

10-Isopropyl-2,3,12,12a-tetrahydropyrrolo[1',2':1,7]azepino[3,4-*b*]indole-5,11(1*H*,6*H*)-dione and 8-isopropyl-2,3,12,12*a*-tetrahydropyrrolo[1',2':1,7]azepino[3,4-*b*]indole-5,11(1*H*,6*H*)-dione (2n)

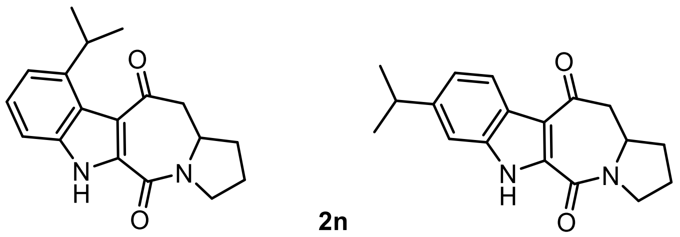

2n
1.5:1 r.r.

7n (66.6 mg, 0.20 mmol, 1.0 equiv), NaOAc (24.6 mg, 0.30 mmol, 1.5 equiv) and *fac*-Ir(ppy)$_3$ (1.3 mg, 0.002 mmol, 1.0 mol%) were suspended in DMSO-*d*$_6$ (2.0 mL). Oxygen was removed from the reaction mixture by bubbling N_2 gas through the suspension for 10 minutes. The reaction mixture was stirred under nitrogen and irradiated with blue light (36 W COB LED) for 2 h. The solvent was removed *in vacuo* and the crude solid purified by flash column chromatography (toluene:acetone 90:10). After column purification, an unknown aliphatic impurity appeared in the ^{1}H-NMR spectra. The source of the impurity could not be determined. The isolated solid was suspended in hexane (1.0 mL × 3), sonicated for 5 minutes and the hexane removed with needle and syringe to give a mixture of regioisomers **2n** *1.5:1 r.r.* (1.5:1, 41.0 mg, 69%, 0.14 mmol) as a cream amorphous solid.

Mp: 228-230 °C.

NMR Spectroscopy (see spectra)**:**

^{1}H NMR (400 MHz, DMSO-*d*$_6$): δ_H 12.45 (s, 0.6H), 12.35 (s, 0.4H), 8.19 (d, *J* = 8.4 Hz, 0.4H), 7.38 (d, *J* = 7.7 Hz, 0.6H), 7.35 (s, 0.4H), 7.28 (t, *J* = 7.7 Hz, 0.6H), 7.17 (d, *J* = 8.4 Hz, 0.4H), 7.13 (d, *J* = 7.7 Hz, 0.6H), 4.34–4.29 (m, 0.6H), 4.22–4.16 (m, 0.4H), 4.00–3.93 (m, 0.6H), 3.91–3.85 (m, 0.4H), 3.79–3.72 (m, 0.6H), 3.61–3.51 (m, 1H), 3.08 (dd, *J* = 17.2, 11.6 Hz, 0.4H), 3.03–2.99 (m, 1.6H), 2.71 (d, *J* = 17.2 Hz, 0.4H), 2.31–2.21 (m, 1H), 2.00–1.92 (m, 1H), 1.85–1.73 (m, 2H), 1.33 (d, *J* = 7.7 Hz, 1.8H), 1.24 (d, *J* = 6.9 Hz, 2.4H) 1.04 (d, *J* = 6.9 Hz, 1.8H);

^{13}C NMR (151 MHz, DMSO-*d*$_6$): δ_C [*a = major regioisomer; b = minor regioisomer*] 194.2 (a), 193.6 (b), 159.0 (a), 158.8 (b), 145.6 (b), 143.9 (a), 136.4 (a), 135.8 (b), 135.5 (a), 135.0 (b), 125.1 (a), 124.5 (b), 123.6 (a), 122.6 (b), 122.3 (b), 118.5 (a), 117.4 (a), 113.8 (b), 110.4 (a), 109.4 (b), 53.8 (b), 53.6 (a), 50.6 (a), 48.9 (b), 47.5 (b), 46.9 (a), 33.7 (b), 33.5 (b), 32.1 (a), 28.9 (a), 24.2 (a), 24.1 (b), 22.9 (a), 22.8 (b), 22.6 (a).

IR (film): v_{max} 3229, 2961, 1617, 1572, 1504 cm^{-1}.

HRMS (ESI+): m/z calc'd for $C_{18}H_{21}N_2O_2$ [M+H]+, 297.1603; found, 297.1601.

10-(*tert*-Butyl)-2,3,12,12a-tetrahydropyrrolo[1',2':1,7]azepino[3,4-*b*]indole-5,11(1*H*,6*H*)-dione and 8-(*tert*-butyl)-2,3,12,12a-tetrahydropyrrolo[1',2':1,7]azepino[3,4-*b*]indole-5,11(1*H*,6*H*)-dione (2o)

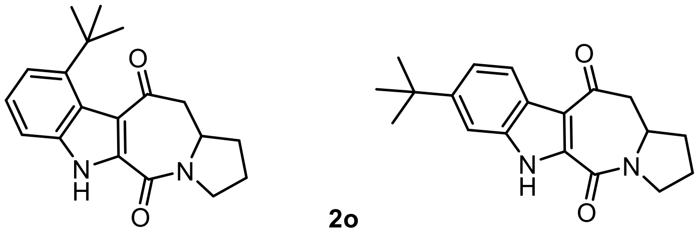

2o
1:1.3 r.r.

3 (69.4 mg, 0.20 mmol, 1.0 equiv), NaOAc (24.6 mg, 0.30 mmol, 1.5 equiv) and *fac*-Ir(ppy)3 (1.3 mg, 0.002 mmol, 1.0 mol%) were suspended in DMSO-*d6* (2.0 mL). Oxygen was removed from the reaction mixture by bubbling N_2 gas through the suspension for 10 minutes. The reaction mixture was stirred under nitrogen and irradiated with blue light (36 W COB LED) for 3 h. The solvent was removed *in vacuo* and the crude solid purified by flash column chromatography (toluene:acetone 90:10). After column purification, an unknown aliphatic impurity appeared in the ^{1}H-NMR spectra. The source of the impurity could not be determined. The isolated solid was suspended in hexane (1.0 mL × 3), sonicated for 5 minutes and the hexane removed with needle and syringe to give a mixture of regioisomers **2o** *1:1.3 r.r.* (1:1.5, 42.0 mg, 67%, 0.13 mmol) as a cream amorphous solid.

Mp: decomposition 260 °C.

NMR Spectroscopy (see spectra):

^{1}**H NMR** (400 MHz, DMSO-*d6*): δ_H 12.42 (s, br, 0.4H), 12.32 (s, br, 0.6H), 8.19 (d, *J* = 8.5 Hz, 0.6H), 7.49 (s, 0.6H), 7.39 (dd, *J* = 5.4, 3.8 Hz, 0.4H), 7.36 (dd, *J* = 8.5, 1.8 Hz, 0.6H), 7.23 (m, 0.8H), 4.43–4.36 (m, 0.4H), 4.22–4.16 (m, 0.6H), 3.91–3.86 (m, 0.6H), 3.70–3.64 (m, 0.4H), 3.61–3.51 (m, 1H), 3.11–3.03 (m, 1H), 2.94 (dd, *J* = 18.5, 12.0 Hz, 0.4H), 2.70 (d, *J* = 17.2 Hz, 0.6H), 2.31–2.18 (m, 1H), 2.01–1.91 (m, 1H), 1.88–1.75 (m, 2H), 1.44 (s, 3.6H), 1.32 (s, 5.4H);

^{13}**C NMR** (151 MHz, DMSO-*d6*): δ_C [*a = major regioisomer; b = minor regioisomer*] 197.4 (a), 193.6 (b), 159.3 (a), 158.8 (b), 147.9 (b), 145.9 (a), 137.7 (a), 135.6 (b), 135.0 (b), 133.9 (a), 124.5 (a), 124.0 (b), 122.3 (b), 122.1 (a), 121.3 (b), 120.2 (a), 119.7 (a), 113.7 (b), 110.9 (a), 108.3 (b), 53.8 (b), 53.5 (a), 52.6 (a), 48.9 (b), 47.5 (b), 46.6 (a), 36.1 (a), 34.6 (b), 33.5 (a), 31.3 (b), 31.2 (a), 31.0 (a), 22.9 (a), 22.6 (b).

IR (film): ν_{max} 3236, 2963, 1648, 1615, 1569, 1526 cm^{-1}.

HRMS (ESI+): m/z calc'd for $C_{19}H_{23}N_2O_2$ [M+H]+, 311.1760; found, 311.1761.

10-Methoxy-2,3,12,12a-tetrahydropyrrolo[1',2':1,7]azepino[3,4-*b*]indole-5,11(1*H*,6*H*)-dione (2p) and 8-methoxy-2,3,12,12a-tetrahydropyrrolo[1',2':1,7]azepino[3,4-*b*]indole-5,11(1*H*,6*H*)-dione (2p')

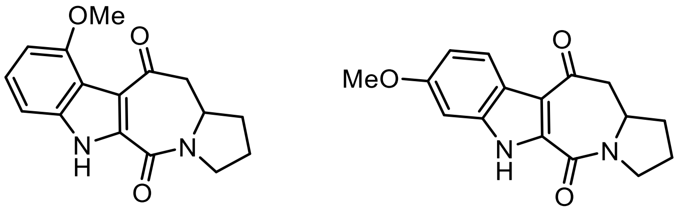

3 (64.2 mg, 0.20 mmol, 1.0 equiv), NaOAc (24.6 mg, 0.30 mmol, 1.5 equiv) and *fac*-Ir(ppy)$_3$ (1.3 mg, 0.002 mmol, 1.0 mol%) were suspended in DMSO-d_6 (2.0 mL). Oxygen was removed from the reaction mixture by bubbling N_2 gas through the suspension for 10 minutes. The reaction mixture was stirred under nitrogen and irradiated with blue light (36 W COB LED) for 2 h. The solvent was removed *in vacuo* and the crude solid purified by flash column chromatography (toluene:acetone 90:10 to 80:20). After column purification, an unknown aliphatic impurity appeared in the ^{1}H-NMR spectra. The source of the impurity could not be determined. The isolated solid was suspended in hexane (1.0 mL × 3), sonicated for 5 minutes and the hexane removed with needle and syringe to give **2p** (23.0 mg, 40%, 0.08 mmol) as a cream amorphous solid and **2p'** (13.0 mg, 23%, 0.05 mmol) as a cream amorphous solid.

*4-Substituted (**2p**)*

Mp: 278 °C.

NMR Spectroscopy (see spectra):

^{1}**H NMR** (400 MHz, DMSO-d_6): δ_H 12.37 (s, 1H), 7.24 (t, *J* = 8.0 Hz, 1H), 7.10 (dd, *J* = 8.0, 0.8 Hz, 1H), 6.67 (d, *J* = 8.0, 0.8 Hz, 1H), 4.32–4.25 (m, 1H), 3.81 (s, 3H), 3.81–3.73 (m, 1H), 3.58–3.52 (m, 1H), 2.92 (dd, *J* = 18.4, 10.7 Hz, 1H), 2.85 (dd, *J* = 18.4, 3.1 Hz, 1H), 2.25–2.19 (m, 1H), 1.99–1.93 (m, 1H), 1.85–1.72 (m, 2H);

^{13}**C NMR** (151 MHz, DMSO-d_6): δ_C 191.8, 159.1, 154.7, 137.6, 133.9, 126.0, 116.9, 115.6, 105.4, 102.8, 55.3, 53.8, 50.4, 46.9, 32.2, 22.8.

IR (film): ν_{max} 3226, 2953, 1724, 1670, 1612, 1578 cm^{-1}.

HRMS (ESI$^+$): m/z calc'd for $C_{16}H_{17}N_2O_3$ [M+H]$^+$, 285.1234; found, 285.1224.

*6-Substituted (**2p'**)*

Mp: decomposition 280 °C.

NMR Spectroscopy (see spectra):

^{1}**H NMR** (400 MHz, DMSO-d_6): δ_H 12.27 (s, 1H), 8.14 (d, *J* = 8.8 Hz, 1H), 6.97 (d, *J* = 2.3 Hz, 1H), 6.89 (dd, *J* = 8.8, 2.3 Hz, 1H), 4.22–4.16 (m, 1H), 3.91–3.85 (m, 1H), 3.79 (s, 3H), 3.57–3.50 (m, 1H), 3.07 (dd, *J* = 17.2, 11.6 Hz, 1H), 2.70 (d, *J* = 17.2 Hz, 1H), 2.31–2.23 (m, 1H), 1.98–1.92 (m, 1H), 1.83–1.73 (m, 2H);

^{13}C NMR (151 MHz, DMSO-d_6): δ_C 193.7, 158.8, 157.7, 136.5, 134.3, 123.6, 120.2, 114.0, 113.6, 94.6, 55.2, 53.7, 48.8, 47.5, 33.5, 22.6.

IR (film): v_{max} 3230, 2957, 1647, 1612, 1578, 1528 cm^{-1}.

HRMS (ESI$^+$): m/z calc'd for $C_{16}H_{17}N_2O_3$ [M+H]$^+$, 285.1234; found, 285.1223.

5,11-Dioxo-1,2,3,5,6,11,12,12a-octahydropyrrolo[1',2':1,7]azepino[3,4-*b*]indole-10-carbonitrile (2q) and 5,11-dioxo-1,2,3,5,6,11,12,12a-octahydropyrrolo[1',2':1,7]azepino [3,4-*b*]indole-8-carbonitrile (2q')

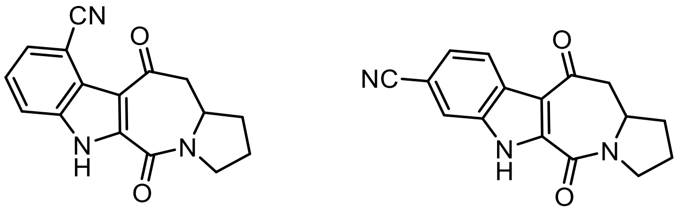

2q **2q'**

3 (63.2 mg, 0.20 mmol, 1.0 equiv), NaOAc (24.6 mg, 0.30 mmol, 1.5 equiv) and *fac*-Ir(ppy)$_3$ (1.3 mg, 0.002 mmol, 1.0 mol%) were suspended in DMSO-d_6 (2.0 mL). Oxygen was removed from the reaction mixture by bubbling N_2 gas through the suspension for 10 minutes. The reaction mixture was stirred under nitrogen and irradiated with blue light (36 W COB LED) for 2 h. The solvent was removed *in vacuo* and the crude solid purified by flash column chromatography (toluene:acetone 90:10). After column purification, an unknown aliphatic impurity appeared in the ^{1}H-NMR spectra. The source of the impurity could not be determined. The isolated solid was suspended in hexane (1.0 mL × 3), sonicated for 5 minutes and the hexane removed with needle and syringe to give **2q** (30.0 mg, 54%, 0.11 mmol) as a cream amorphous solid and **2q'** (17.0 mg, 30%, 0.07 mmol) as a cream amorphous solid.

*4-Substituted (**2q**)*

Mp: decomposition 268 °C.

NMR Spectroscopy (see spectra):

^{1}H NMR (400 MHz, DMSO-d_6): δ_H 13.03 (s, 1H), 7.87 (dd, J = 8.3, 1.0 Hz, 1H), 7.77 (dd, J = 7.4, 1.0 Hz, 1H), 7.48 (dd, J = 8.3, 7.4 Hz, 1H), 4.35–4.29 (m, 1H), 3.87–3.82 (m, 1H), 3.61–3.55 (m, 1H), 3.10 (dd, J = 18.0, 11.7 Hz, 1H), 2.86 (d, J = 18.0 Hz, 1H), 2.32–2.25 (m, 1H), 2.00–1.93 (m, 1H), 1.86–1.76 (m, 2H);

^{13}C NMR (126 MHz, DMSO-d_6): δ_C 192.5, 158.0, 136.9, 136.1, 131.1, 124.8, 124.0, 118.6, 118.2, 114.4, 105.2, 53.5, 48.9, 47.5, 32.9, 22.7.

IR (film): v_{max} 3191, 2972, 2215, 1658, 1616, 1528 cm^{-1}.

HRMS (ESI$^+$): m/z calc'd for $C_{16}H_{14}N_3O_2$ [M+H]$^+$, 280.1081; found, 280.1071.

*6-Substituted (**2q'**)*

Mp: decomposition 290 °C.

NMR Spectroscopy (see spectra):

^{1}**H NMR** (400 MHz, DMSO-*d*$_6$): δ_H 12.95 (s, br, 1H), 8.44 (d, *J* = 8.4 Hz, 1H), 7.95 (s, 1H), 7.61 (dd, *J* = 8.0, 2.3 Hz, 1H), 4.30–4.24 (m, 1H), 3.92–3.87 (m, 1H), 3.60–3.54 (m, 1H), 3.17 (dd, *J* = 17.2, 11.6 Hz, 1H), 2.76 (d, *J* = 17.2 Hz, 1H), 2.32–2.26 (m, 1H), 1.98–1.92 (m, 1H), 1.83–1.76 (m, 2H);

^{13}**C NMR** (151 MHz, DMSO-*d*$_6$): δ_C 193.7, 158.0, 138.3, 134.2, 129.2, 125.2, 123.9, 119.5, 117.6, 113.7, 106.5, 53.7, 48.7, 47.7, 33.4, 22.6.

IR (film): ν_{max} 3211, 2982, 2223, 1650, 1624, 1566, 1528 cm^{-1}.

HRMS (ESI$^+$): m/z calc'd for $C_{16}H_{14}N_3O_2$ [M+H]$^+$, 280.1081; found, 280.1083.

7-Methoxy-2,3,12,12a-tetrahydropyrrolo[1',2':1,7]azepino[3,4-*b*]indole-5,11(1*H*,6*H*)-dione (2r)

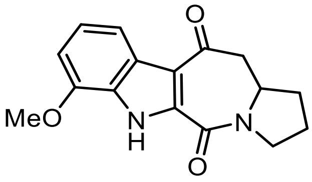

2r

3 (64.2 mg, 0.20 mmol, 1.0 equiv), NaOAc (24.6 mg, 0.30 mmol, 1.5 equiv) and *fac*-Ir(ppy)$_3$ (1.3 mg, 0.002 mmol, 1.0 mol%) were suspended in DMSO-*d*$_6$ (2.0 mL). Oxygen was removed from the reaction mixture by bubbling N_2 gas through the suspension for 10 minutes. The reaction mixture was stirred under nitrogen and irradiated with blue light (36 W COB LED) for 2 h. The solvent was removed *in vacuo* and the crude solid purified by flash column chromatography (toluene:acetone 90:10) to give **2r** (39.0 mg, 69%, 0.14 mmol) as a cream amorphous solid.

Mp: decomposition 237 °C.

NMR Spectroscopy (see spectra):

^{1}**H NMR** (400 MHz, DMSO-*d*$_6$): δ_H 12.21 (s, 1H), 7.87 (dd, *J* = 7.9, 0.9 Hz, 1H), 7.17 (t, *J* = 7.9 Hz, 1H), 6.87 (dd, *J* = 7.9, 0.9 Hz, 1H), 4.25–4.19 (m, 1H), 3.92 (s, 3H), 3.87–3.89 (m, 1H), 3.61–3.53 (m, 1H), 3.10 (dd, *J* = 17.4, 11.9 Hz, 1H), 2.71 (d, *J* = 17.4 Hz, 1H), 2.32–2.24 (m, 1H), 1.98–1.91 (m, 1H,), 1.85–1.72 (m, 2H);

^{13}**C NMR** (126 MHz, DMSO-*d*$_6$): δ_C 194.0, 158.3, 146.6, 135.4, 127.6, 126.0, 123.7, 114.9, 114.3, 105.2, 55.5, 53.5, 49.2, 47.4, 33.3, 22.6.

IR (film): ν_{max} 3218, 2957, 1624, 1528, 1505 cm^{-1}.

HRMS (ESI$^+$): m/z calc'd for $C_{16}H_{17}N_2O_3$ [M+H]$^+$, 285.1234; found, 285.1227.

2.6. Comparison of Yields in DMSO-d_6 and Anhydrous DMSO-H_6

Investigations into using anhydrous DMSO-H_6 rather than DMSO-d_6 found that in all cases the isolated yield was lower when DMSO-H_6 was used (Scheme S1). It was hypothesized that different amounts of water in DMSO-d_6 and anhydrous DMSO-H_6 was the cause of the difference in yields; however, investigations into this effect through the addition of water to the reactions proved inconclusive.

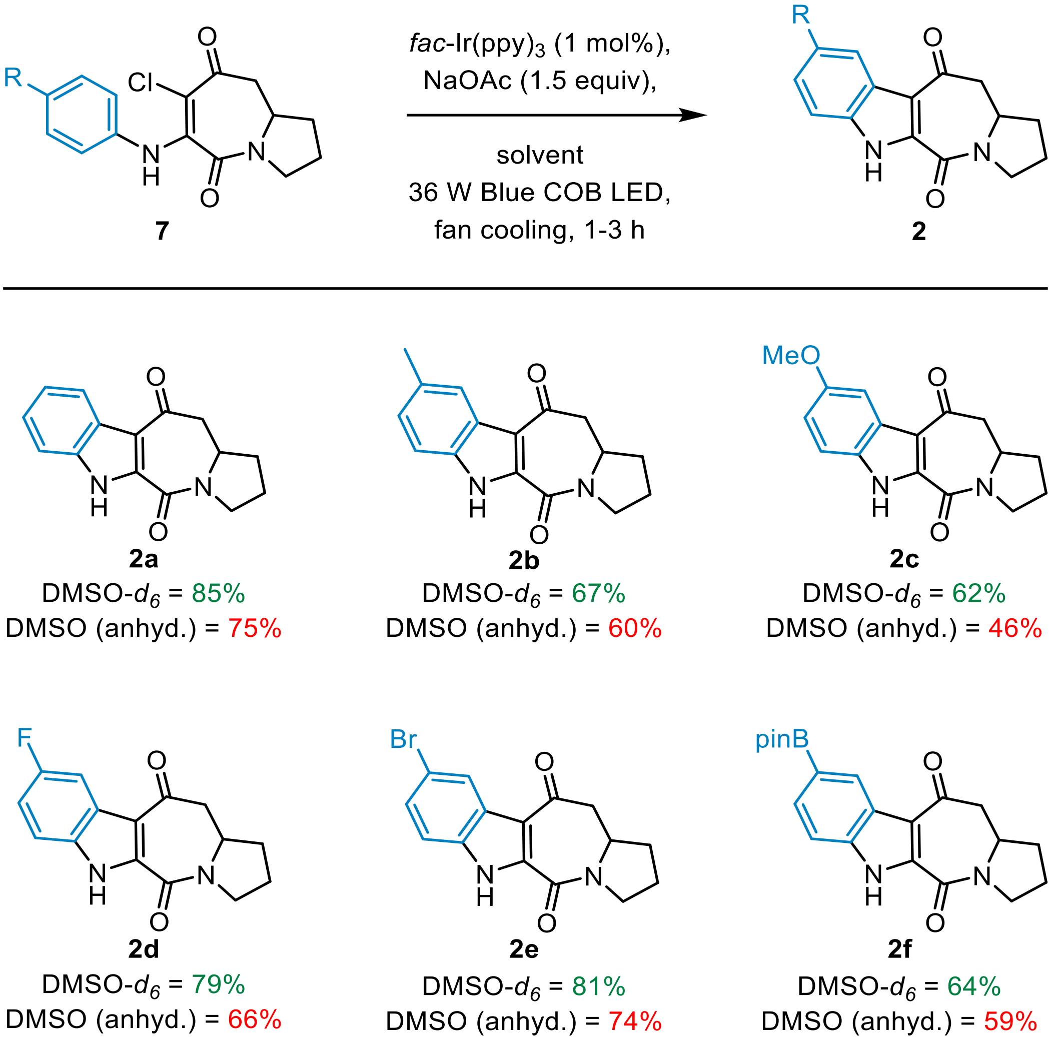

Scheme S1. Comparison of isolated yields for indoloazepinedione substrates in anhydrous DMSO-H_6 and DMSO-d_6.

3. X-RAY CRYSTALLOGRAPHIC ANALYSIS

3.1. Aminated Chloro-Azepinedione 7e (CCDC 2473598)

Vapor diffusion crystallization method was used to produce a crystal of **7e** for x-ray crystallographic analysis. **7e** (10 mg, 0.03 mmol) was dissolved in minimal MeCN. The solution, in a 1.75 ml vial with no lid, was placed in a larger vial containing cyclohexane. The larger vial was sealed with a lid and left to stand for over a week at room temperature until a crystal was formed.

X-ray diffraction experiments on **7e** were carried out at 100(2) K on a Bruker APEX II diffractometer using Mo-Kα radiation (λ = 0.71073 Å). Intensities were integrated in SAINT1 and absorption corrections based on equivalent reflections were applied using SADABS.2 The structure was solved using ShelXS3 and refined by full matrix least squares against F2 in ShelXL3, 4 using Olex25. The structure contained two unique molecules in the asymmetric unit. All of the non-hydrogen atoms were refined anisotropically. All of the hydrogen atoms were located geometrically and refined using a riding model, apart from the N-H protons which were located in the difference map and refined freely. The structure was refined as a two component twin with a twin scale fraction of 0.43 for the second component. Crystal structure and refinement data are given in Table S1.[3-7] The ellipsoid plot for **7e** with 50% probability level is shown below.

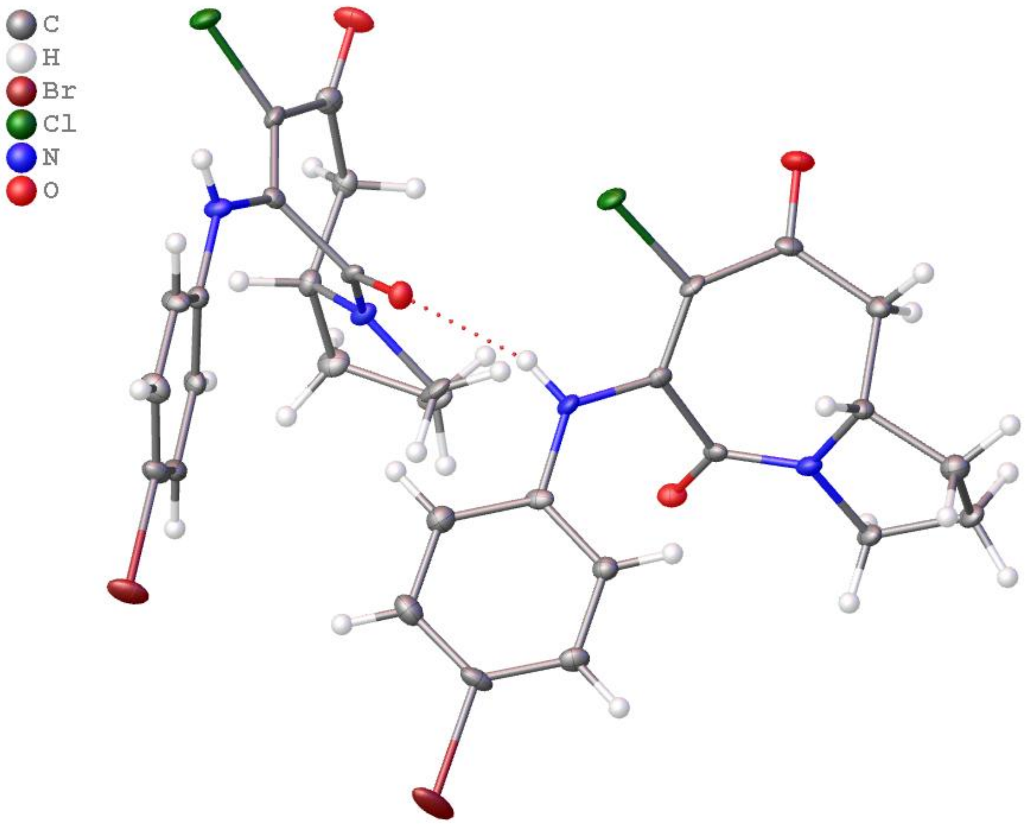

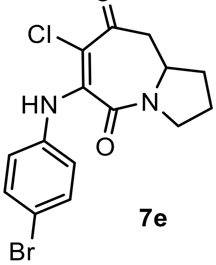

7e

Table S1. Crystal data and structure refinement for KES475BK9 (7e).

Identification code	KES475BK9
Empirical formula	$C_{15}H_{14}BrClN_2O_2$
Formula weight	369.64
Temperature/K	100(2)
Crystal system	monoclinic
Space group	P21/c
a/Å	9.7848(10)
b/Å	20.193(2)
c/Å	14.778(2)
α/°	90
β/°	90.230(6)
γ/°	90
Volume/Å^3	2920.0(6)
Z	8
ρ_{calc}g/cm^3	1.682
μ/mm^{-1}	3.004
F(000)	1488.0
Crystal size/mm^3	0.493 × 0.407 × 0.3
Radiation	MoKα (λ = 0.71073)
2θ range for data collection/°	2.016 to 55.928
Index ranges	-12 ≤ h ≤ 12, -26 ≤ k ≤ 26, -19 ≤ l ≤ 19
Reflections collected	41236
Independent reflections	6980 [R_{int} = 0.0809, R_{sigma} = 0.0593]
Data/restraints/parameters	6980/1/389
Goodness-of-fit on F^2	1.081
Final R indexes [I>=2σ (I)]	R_1 = 0.0396, wR_2 = 0.0785
Final R indexes [all data]	R_1 = 0.0540, wR_2 = 0.0830
Largest diff. peak/hole / e Å^{-3}	0.59/-0.79

4. MECHANISTIC EXPERIMENTS

4.1. Cyclic Voltammogram of Aminated Chloro-Azepinedione 7a

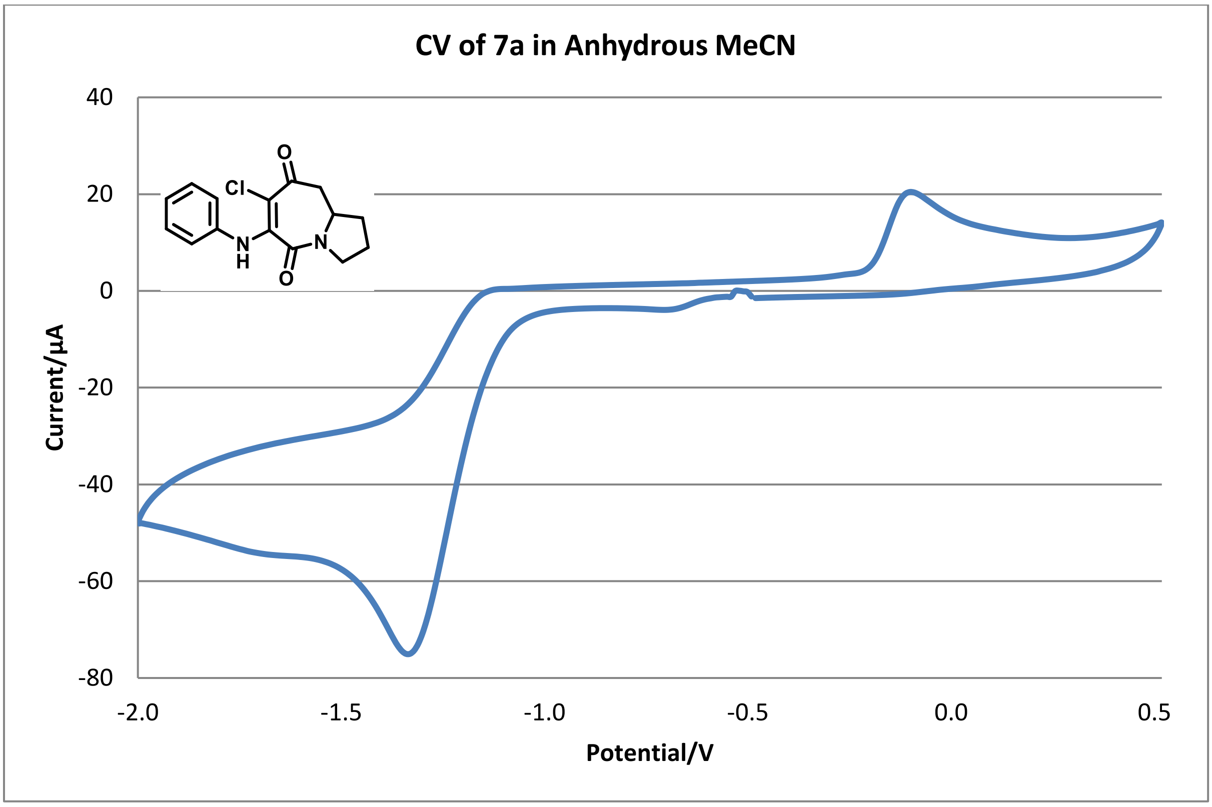

7a was dissolved in MeCN (anhydrous) at a concentration of 5.0 mM. Tetrabutylammonium hexafluorophosphate was used as the electrolyte (0.1 M). Nitrogen gas was bubbled through the solution for 10 minutes to ensure that the solution was deoxygenated. Starting at –0.5 V and scanning initially in the reductive direction, the cyclic voltammetry analysis of **7a** was run with a scan rate of 0.1 V s^{-1}.

The peak reduction potential (referenced to ferrocene/ferrocenium) of **7a** was calculated as follows:

$$E_p = -1.35 \text{ V} - ((0.15 \text{ V} + 0 \text{ V})/2) = -1.43 \text{ V vs. Fc/Fc}^+$$

The Fc/Fc^+ redox couple was converted to saturated calomel electrode (SCE) using the conversion: E (Fc/Fc^+) = +0.38 V vs. SCE.

$$E_p = -1.05 \text{ V vs. SCE}$$

4.2. Fluorescence Quenching Studies and UV/vis Spectroscopy

To investigate the reaction of the excited state photocatalysts with aminated chloro-azepinedione **7a**, we performed fluorescence quenching studies and UV/vis spectroscopy.

Fluorescence quenching studies were performed at 298 K on a Horiba Fluoromax spectrofluorometer in a quartz cuvette (3 mL, 10 mm path length). Studies were conducted on 1 μM solutions of photocatalyst in dry protic DMSO. Photocatalyst solutions were freshly prepared and degassed by sparging with N_2 for 5 mins before readings were taken.

Ir(ppy)₃: A 1 μM solution of $Ir(ppy)_3$ in DMSO was irradiated at 377 nm and the emission was recorded between 420 and 700 nm. In the absence of quencher (**7a**), an emission at λ_{max} = 520 nm was observed. This emission signal was completely quenched at 4.54 mM of quencher **7a**.

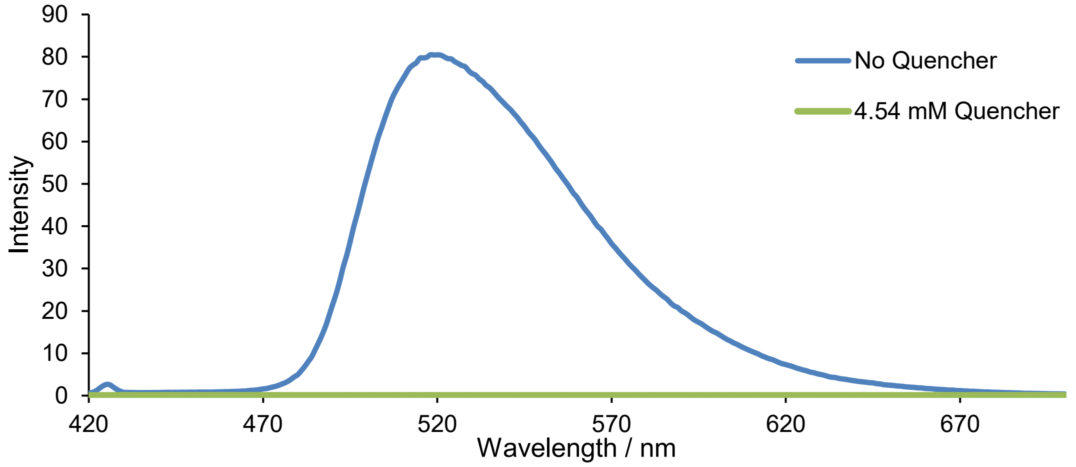

Eosin Y disodium salt (Na_2EY): A 1 μM solution of Na_2EY in DMSO was irradiated at 450 nm, and emission spectra were recorded between 470 and 800 nm. In the absence of quencher **7a**, and at low quencher concentrations (<0.2 mM), an emission at λ_{max} = 550 nm was observed. Increased quencher concentrations led to a non-linear decrease in emission intensity and a shift in λ_{max} to 559 nm.

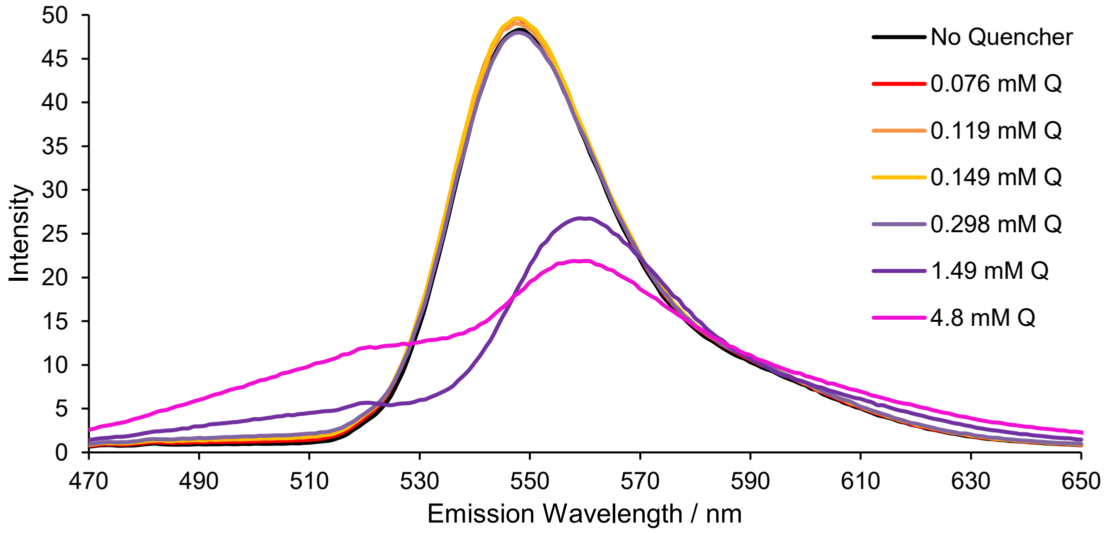

UV/Vis studies were performed on a PerkinElmer LAMBDA 750 UV/Vis/NIR Spectrophotometer at 298 K in a quartz cuvette (10 mm path length). A 34 µM solution of quencher **7a** in dry protic DMSO was used to obtain its absorption spectrum. This was normalised with respect to its λ_{max} value at 350 nm.

Ir(ppy)₃: A 50 µM solution of the catalyst in DMSO was used with increasing concentrations of quencher **7a**. The spectra were normalised relative to the maximum absorption intensity at 288 nm. The above fluorescence quenching results can be explained by the greater absorbance of **7a** compared to $Ir(ppy)_3$ at the excitation wavelength of 377 nm.

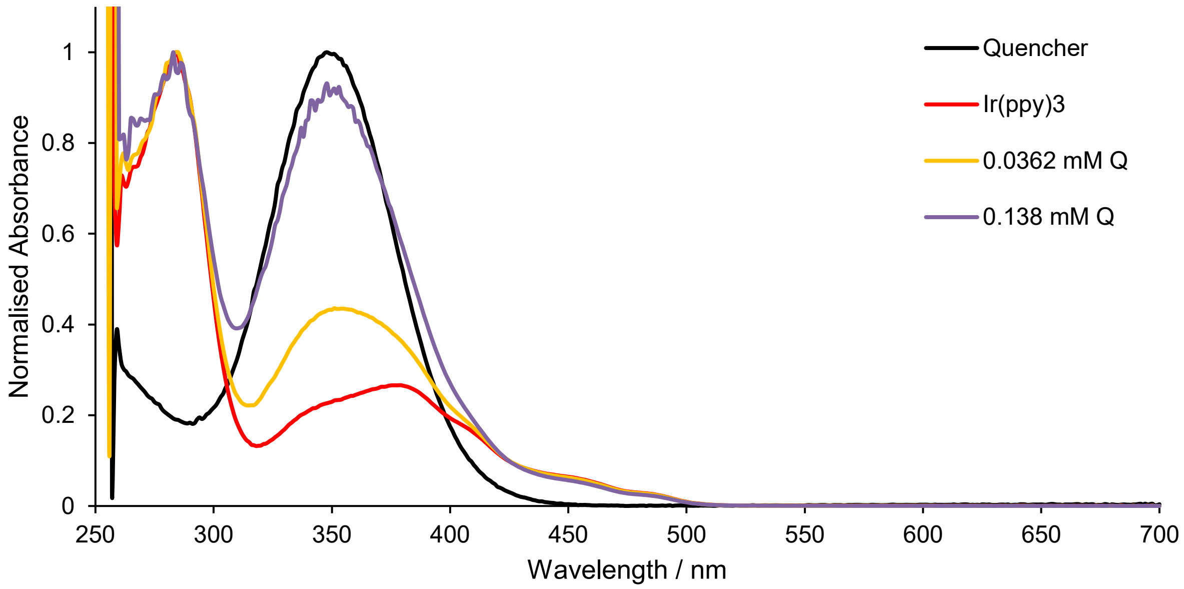

Eosin Y disodium salt (Na_2EY): A 10 µM solution of the catalyst in dry protic DMSO was used with increasing concentrations of quencher **7a**. The spectra were normalised relative to the λ_{max} value of the Na_2EY signal. A bathochromic shift was observed with increasing concentrations of **7a**, which is indicative of a ground state interaction and could explain the shift in emission λ_{max} in the above fluorescence quenching studies. This ground state interaction could result from an electron donor–acceptor (EDA) complex formed between the electron-rich aromatic system in eosin Y and the electron deficient alkene of **7a**.[8]

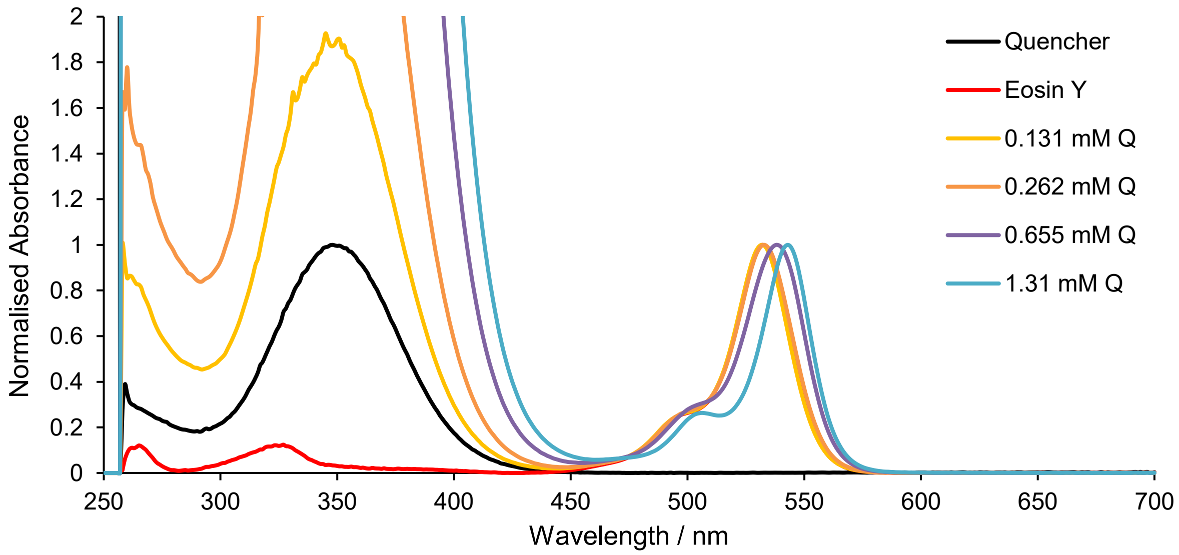

5. DFT CALCULATIONS

5.1. Calculated Triplet Energy of 7a

Calculations were undertaken with Gaussian09W on a standard PC [Intel(R) Core(TM) i3-8100, 8.00 GB RAM].[9,10]

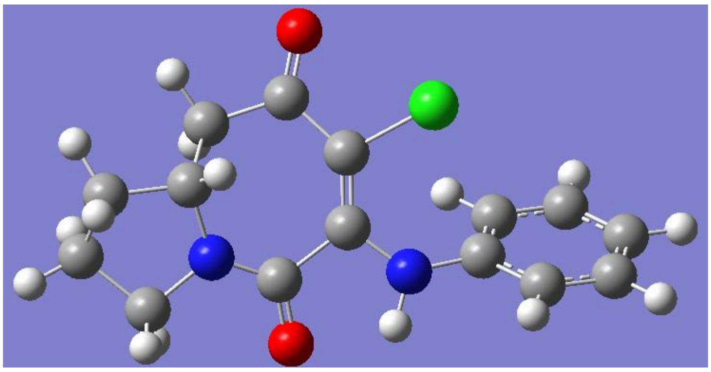

Route – td=(triplets,nstates=1,root=1) b3lyp/3-21g geom=connectivity

Charge = 0 Multiplicity = 1

Total Energy = -1293.8588 au

Triplet Energy = 2.4468 eV (236 kJ/mol)

Optimised Coordinates

C	1.17673	-1.30064	0.03390
C	0.00616	-0.30788	-0.16239
C	2.52755	1.41704	0.58118
C	0.08631	1.03210	-0.16376
C	1.14681	1.95581	0.27372
H	2.55006	0.96401	1.56534
H	3.17503	2.28231	0.58212
C	3.51711	-1.91580	0.17427
H	3.67156	-2.53676	-0.69908
H	3.23476	-2.54398	1.00297
N	2.44551	-0.92779	-0.09203
O	0.87184	-2.46899	0.25877
O	0.94175	3.14635	0.38377
Cl	-1.36139	1.97419	-0.74327
N	-1.11130	-1.05732	-0.35365
H	-0.89845	-2.03869	-0.38516
C	-2.47935	-0.75723	-0.07926
C	-2.86725	-0.15501	1.10607
C	-3.43926	-1.18170	-0.98029
C	-4.20824	0.03659	1.37575
H	-2.12442	0.17219	1.80374

C	-4.78208	-1.00219	-0.70092
H	-3.12823	-1.63728	-1.89828
C	-5.17092	-0.38881	0.47587
H	-4.50064	0.51254	2.29029
H	-5.51898	-1.33277	-1.40532
H	-6.21048	-0.24202	0.69008
C	4.73029	-1.01731	0.45054
H	4.71456	-0.68051	1.47946
H	5.66668	-1.52276	0.26141
C	2.96673	0.39796	-0.46261
H	2.59610	0.68546	-1.43766
C	4.49589	0.16878	-0.50552
H	5.04116	1.05819	-0.22463
H	4.78794	-0.11454	-1.50943

6. SPECTROSCOPIC DATA

^1H NMR (400 MHz, DMSO-d_6) of **6**

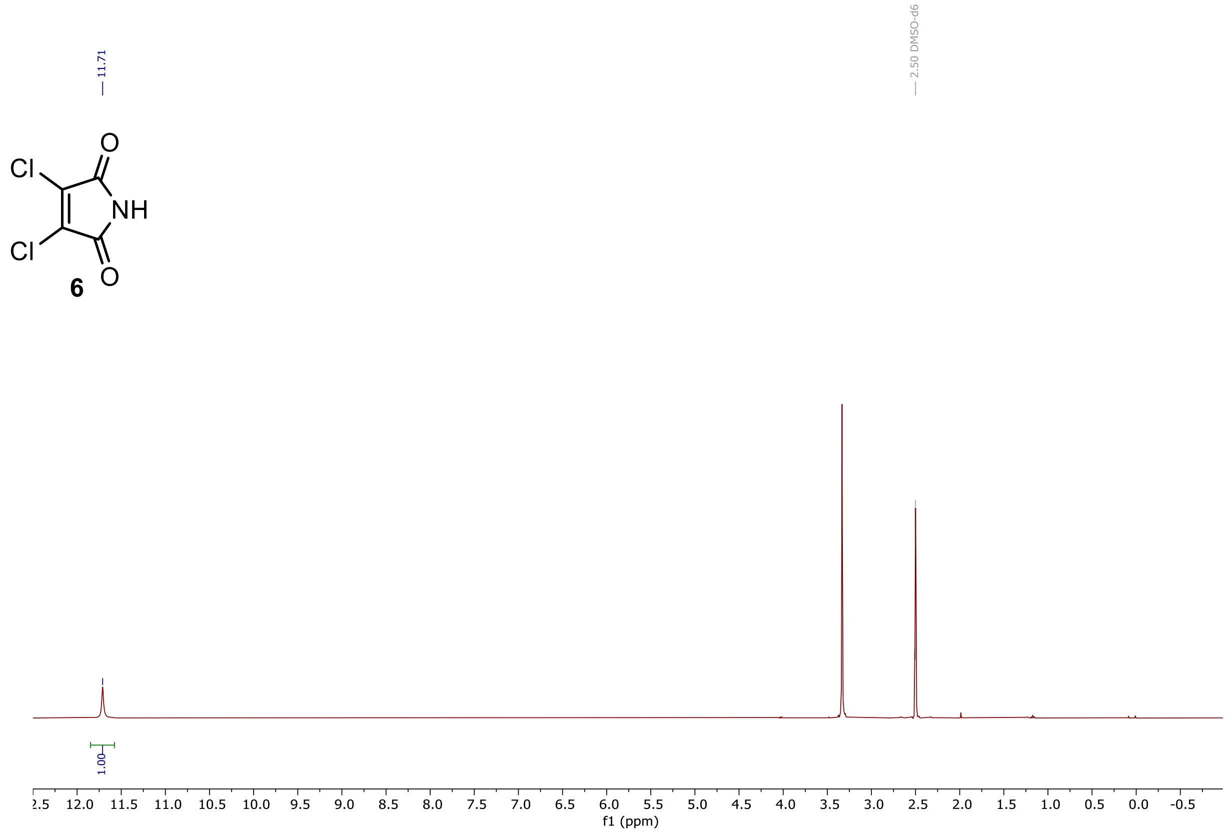

^{13}C NMR (101 MHz, DMSO-d_6) of **6**

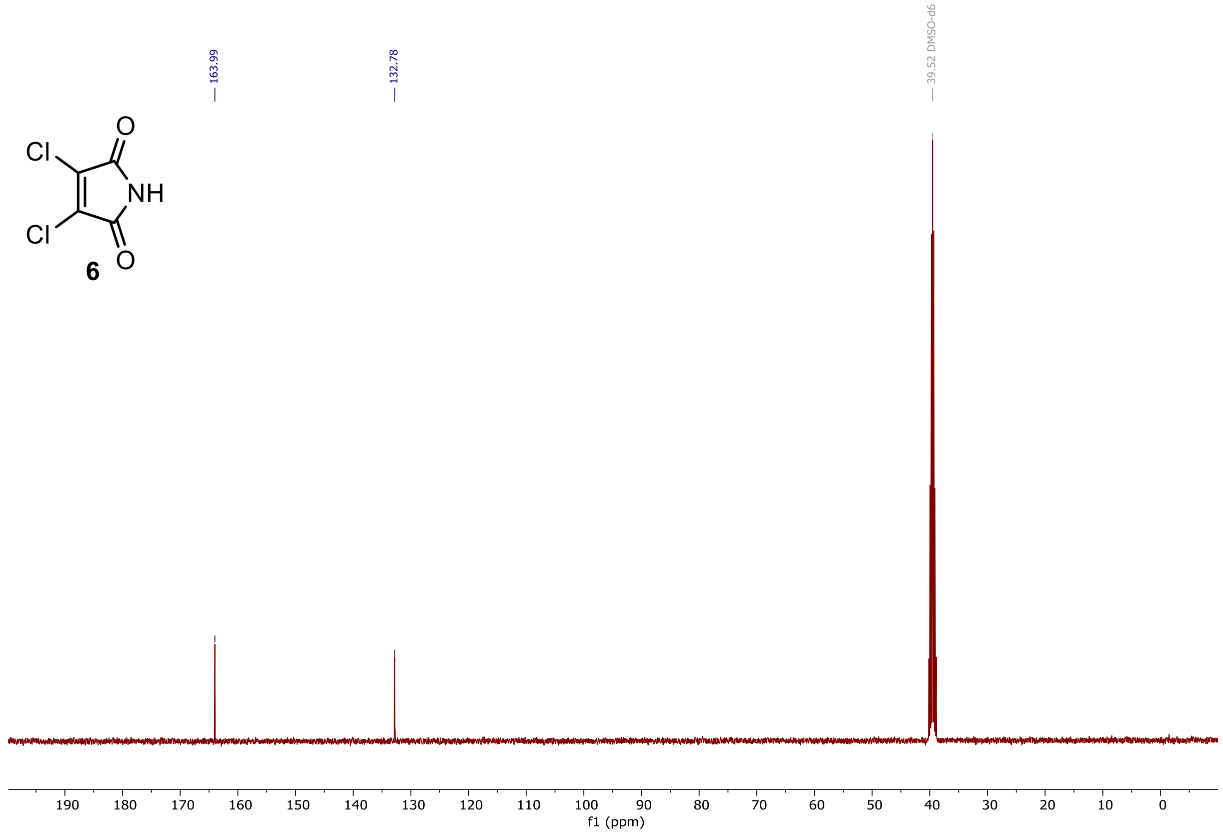

^{1}H NMR (400 MHz, $CDCl_3$) of **5**

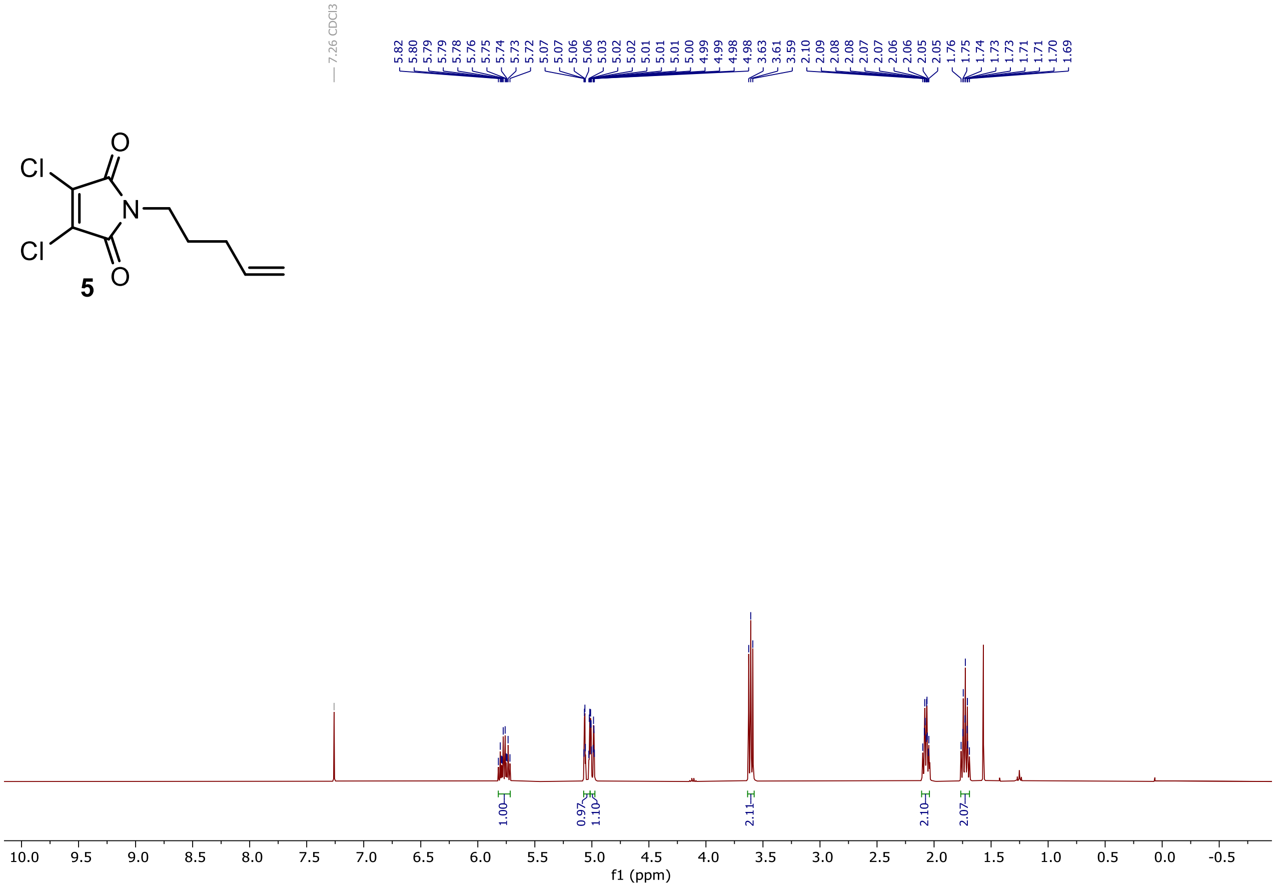

^{13}C NMR (101 MHz, $CDCl_3$) of **5**

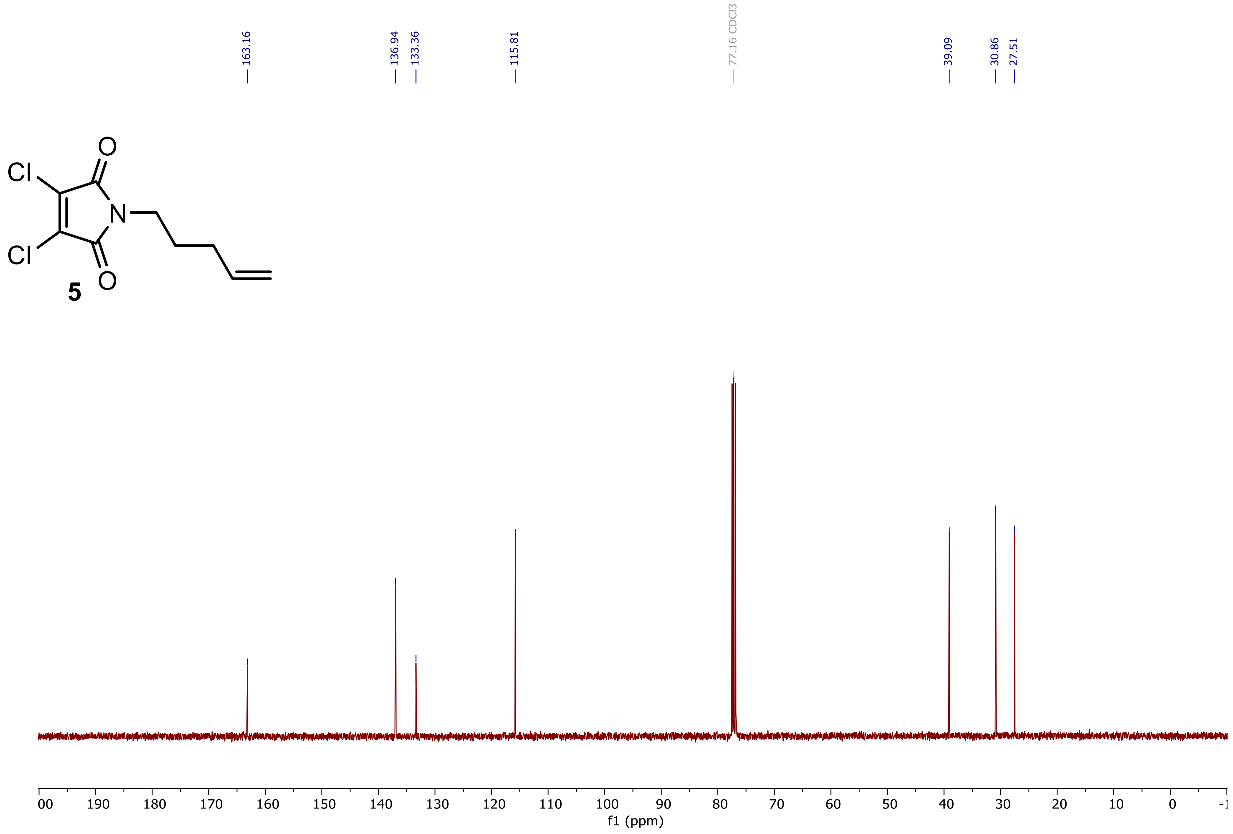

^{1}H NMR (400 MHz, $CDCl_3$) of **3**

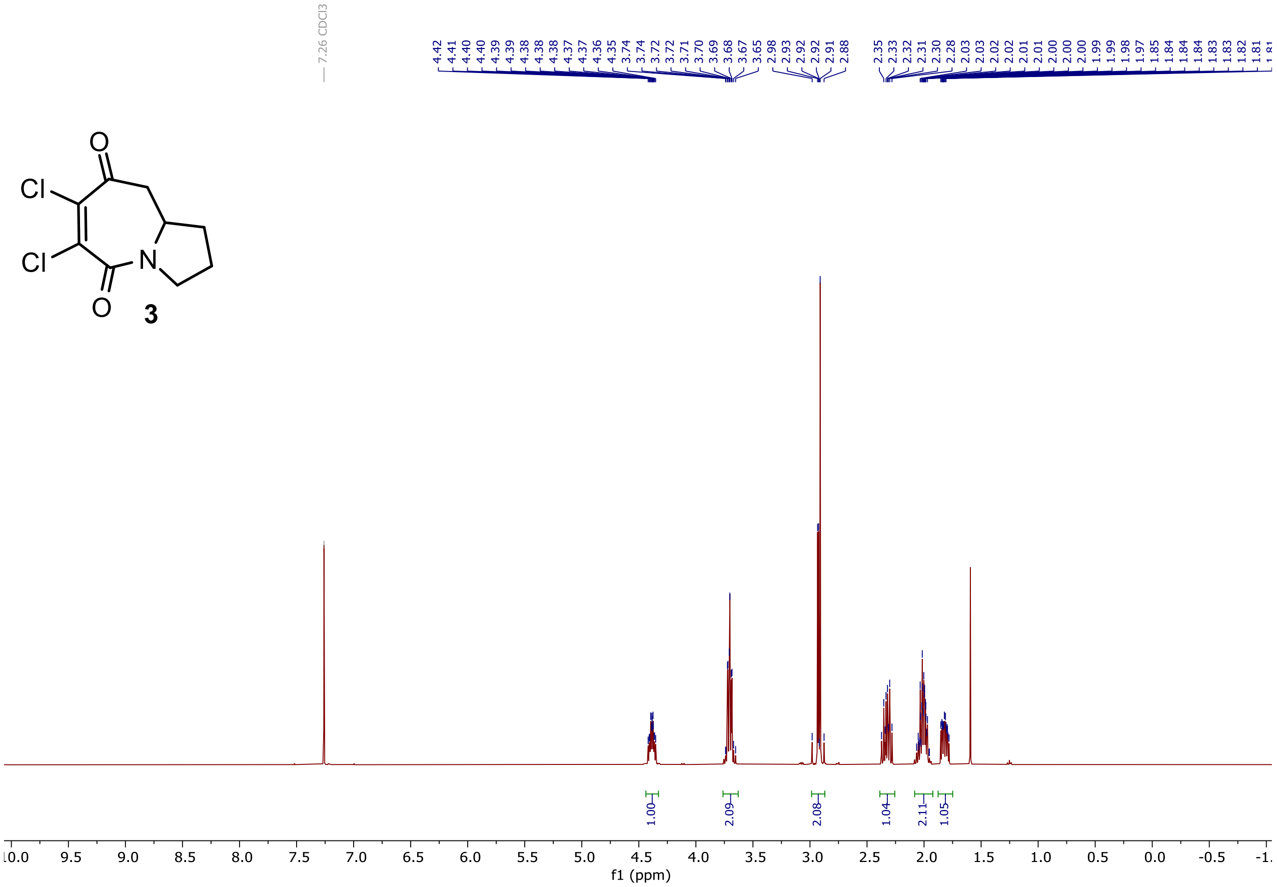

^{13}C NMR (101 MHz, $CDCl_3$) of **3**

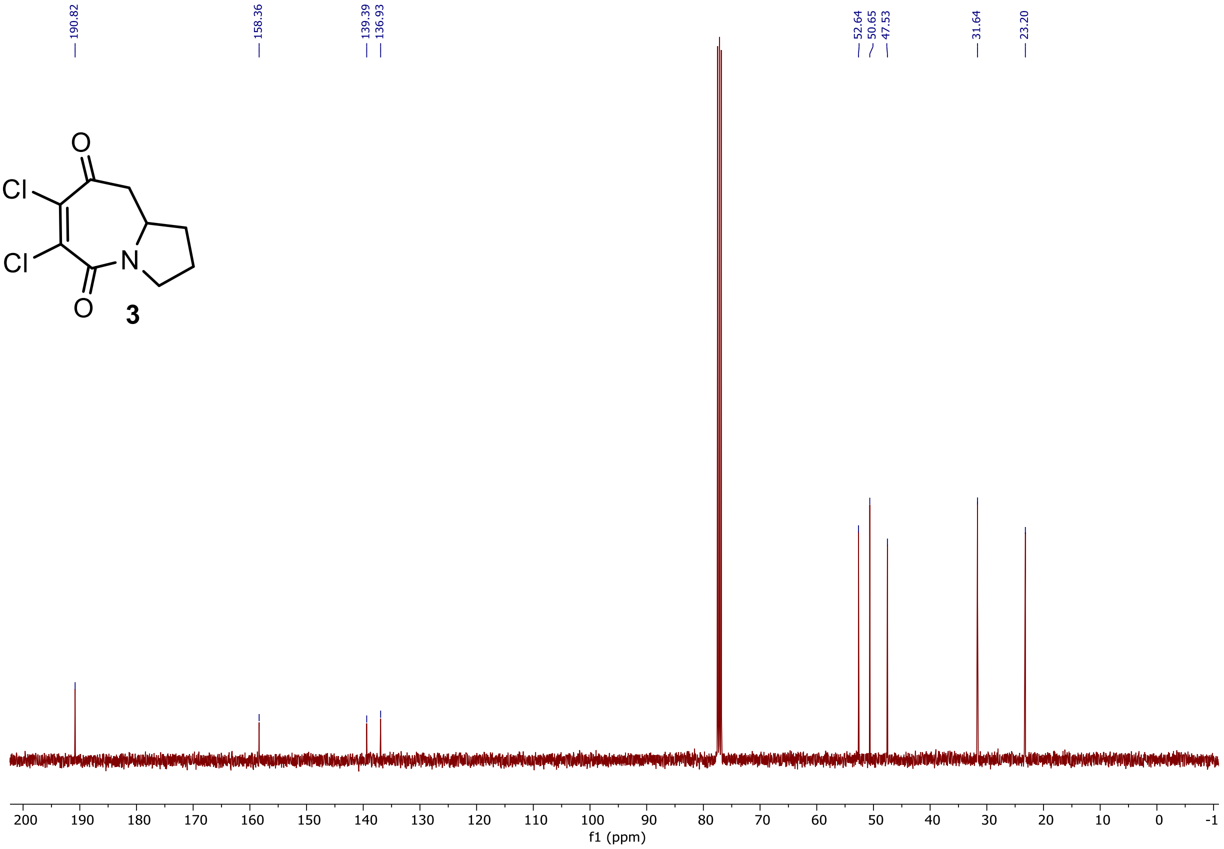

^{1}H NMR (400 MHz, $CDCl_3$) of **7a**

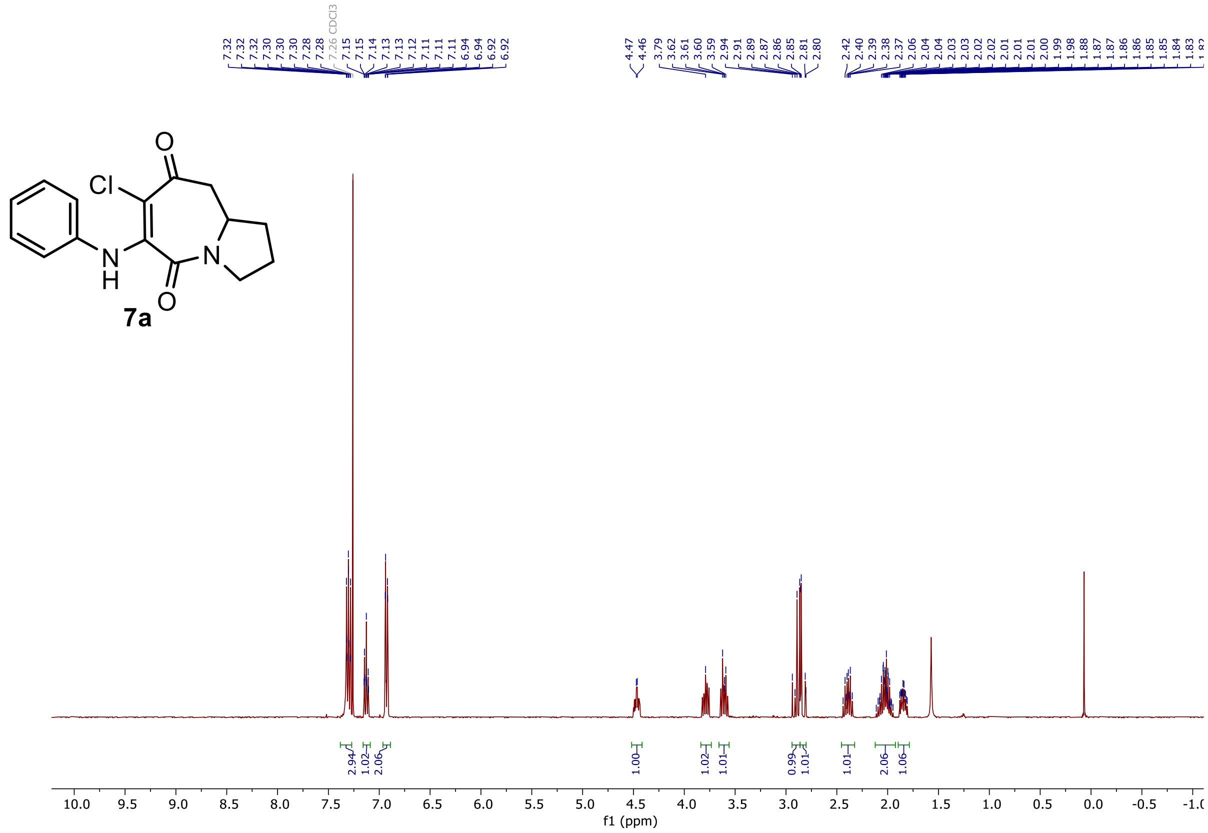

^{13}C NMR (101 MHz, $CDCl_3$) of **7a**

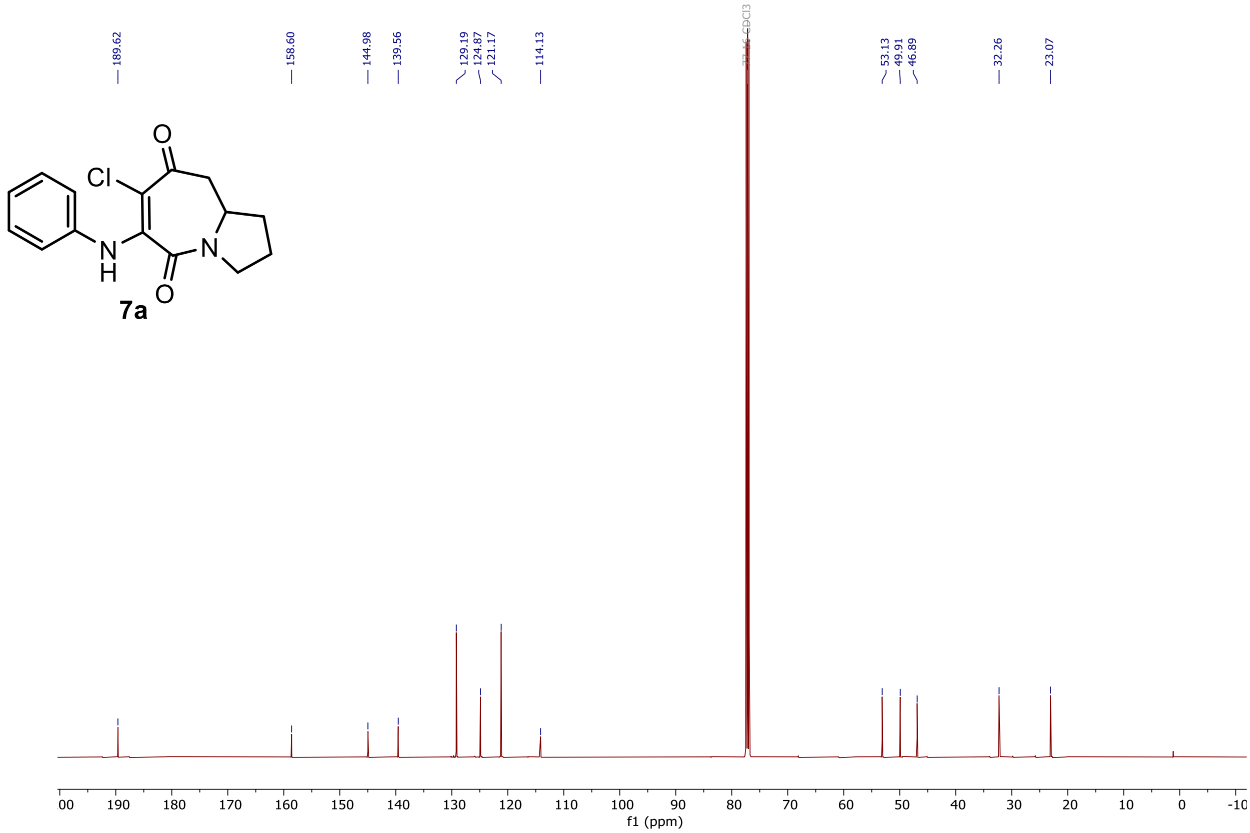

^{1}H NMR (400 MHz, $CDCl_3$) of **7b**

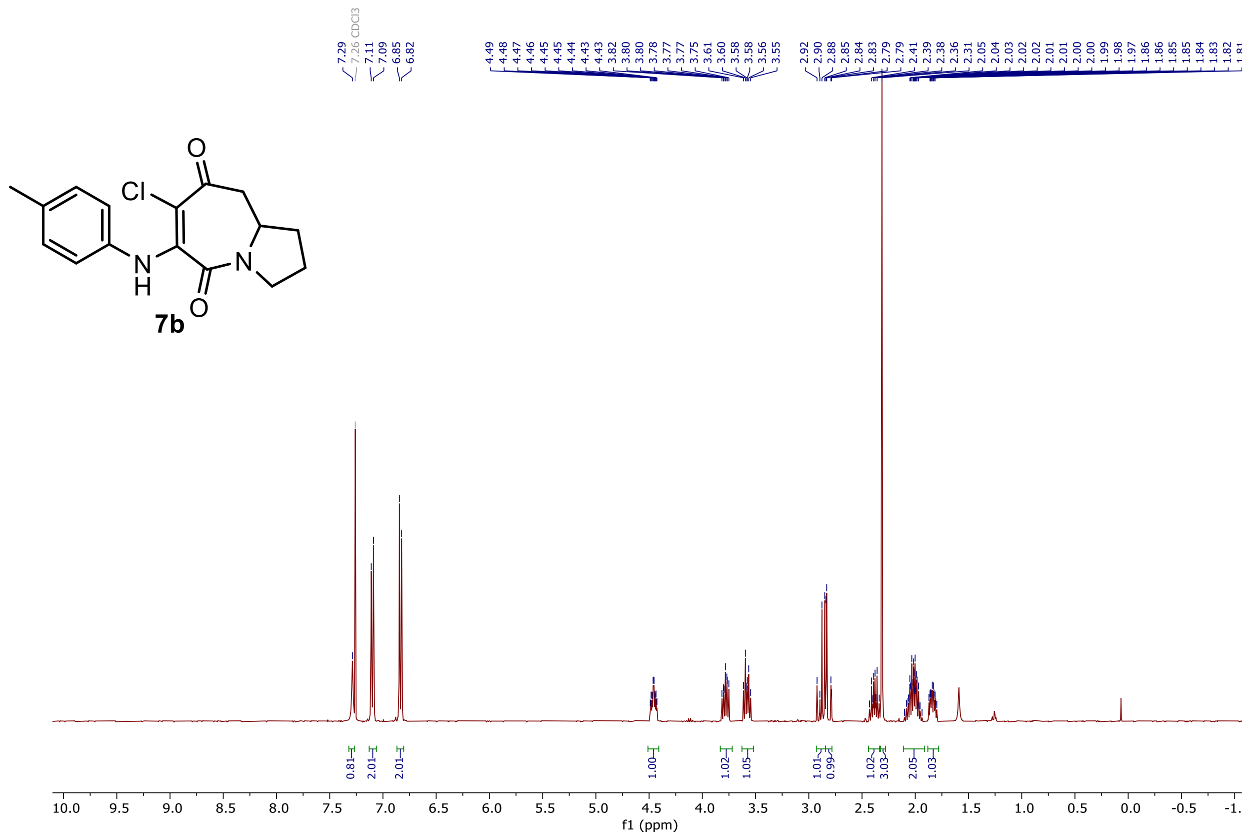

^{13}C NMR (101 MHz, $CDCl_3$) of **7b**

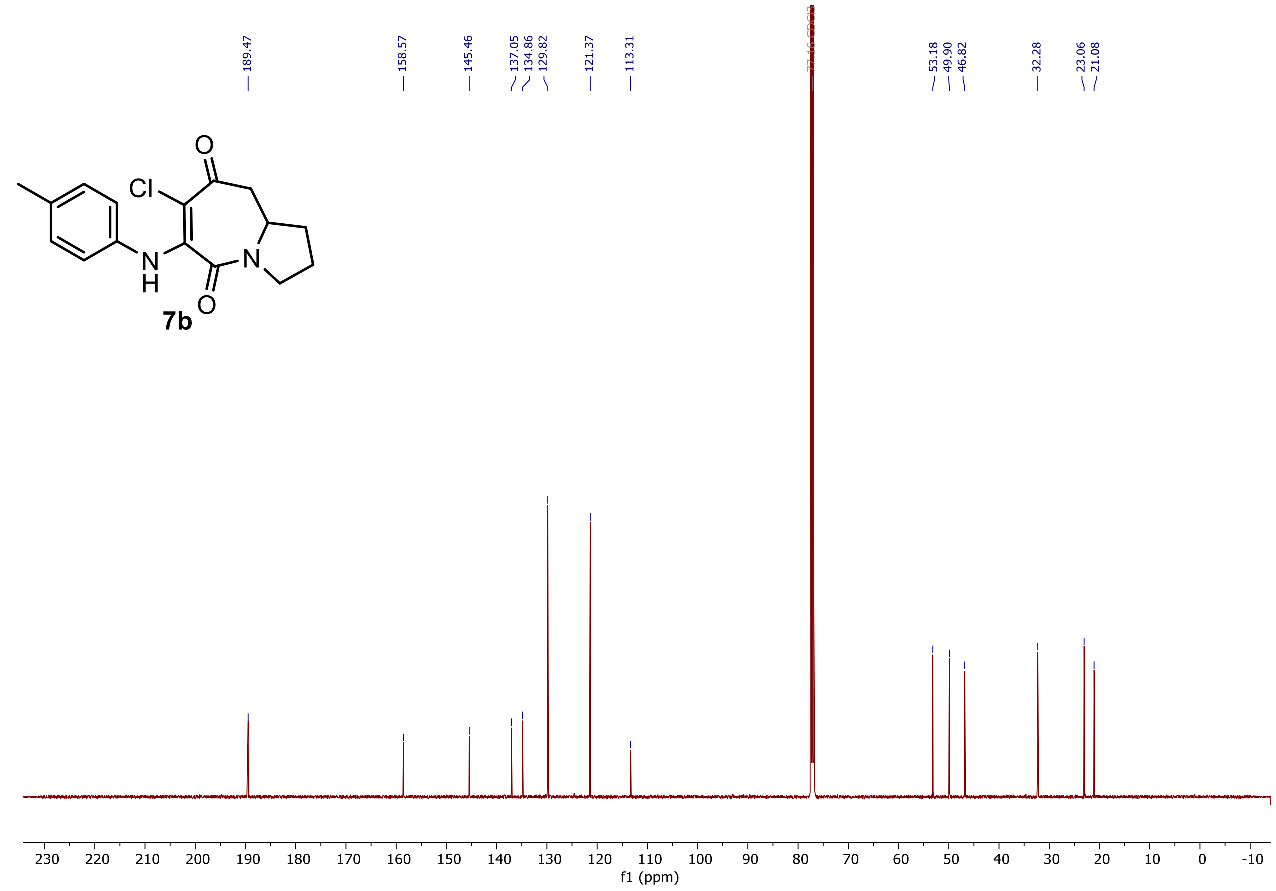

^{1}H NMR (400 MHz, $CDCl_3$) of **7c**

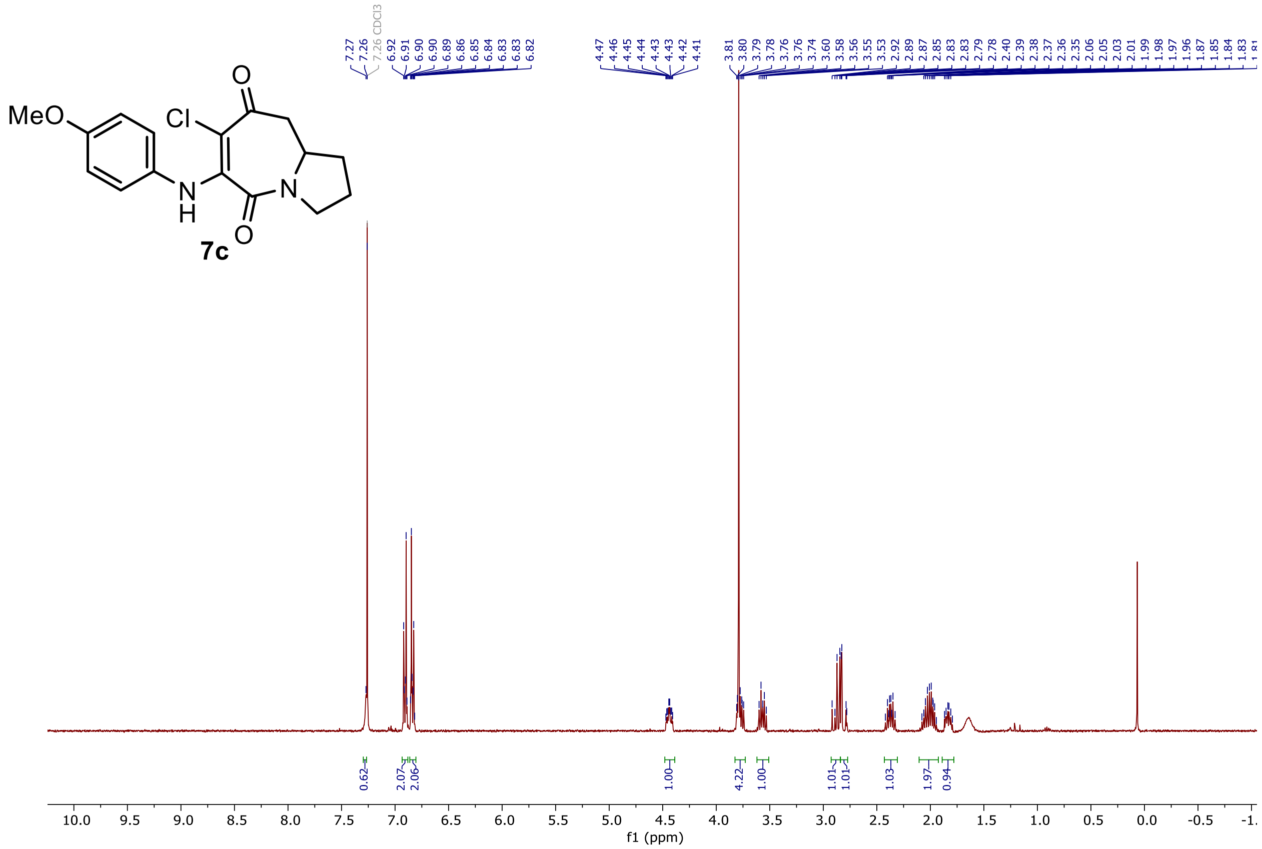

^{13}C NMR (101 MHz, $CDCl_3$) of **7c**

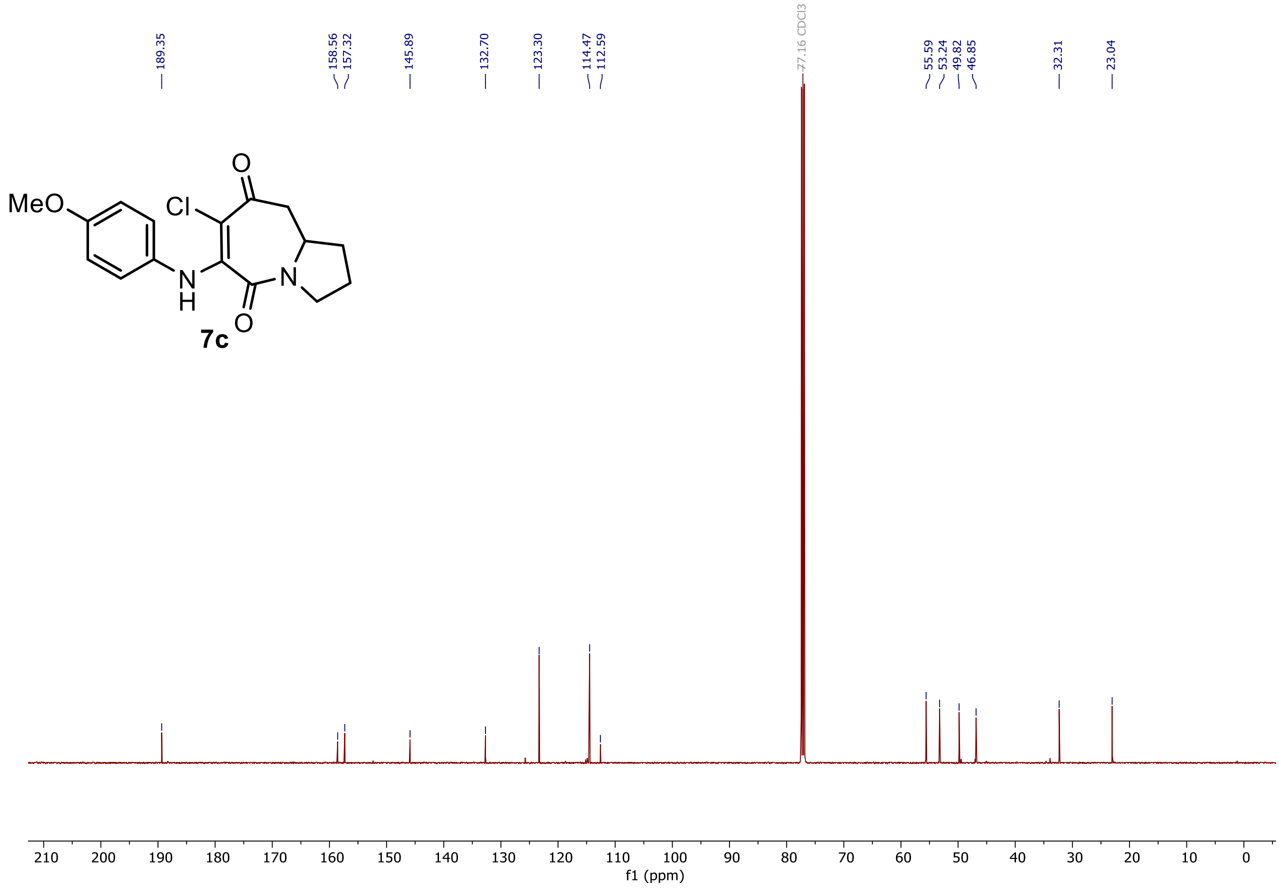

^{1}H NMR (400 MHz, $CDCl_3$) of **7d**

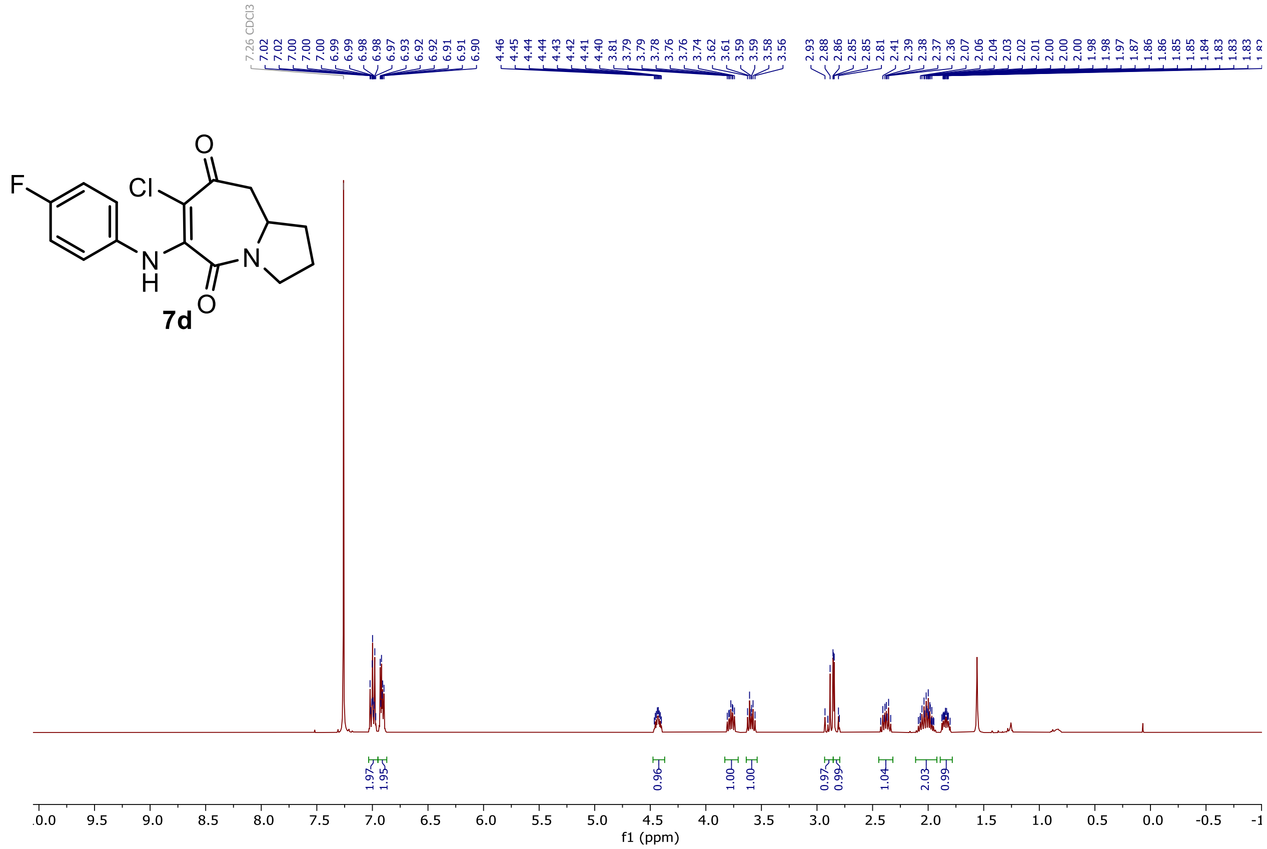

^{13}C NMR (126 MHz, $CDCl_3$) of **7d**

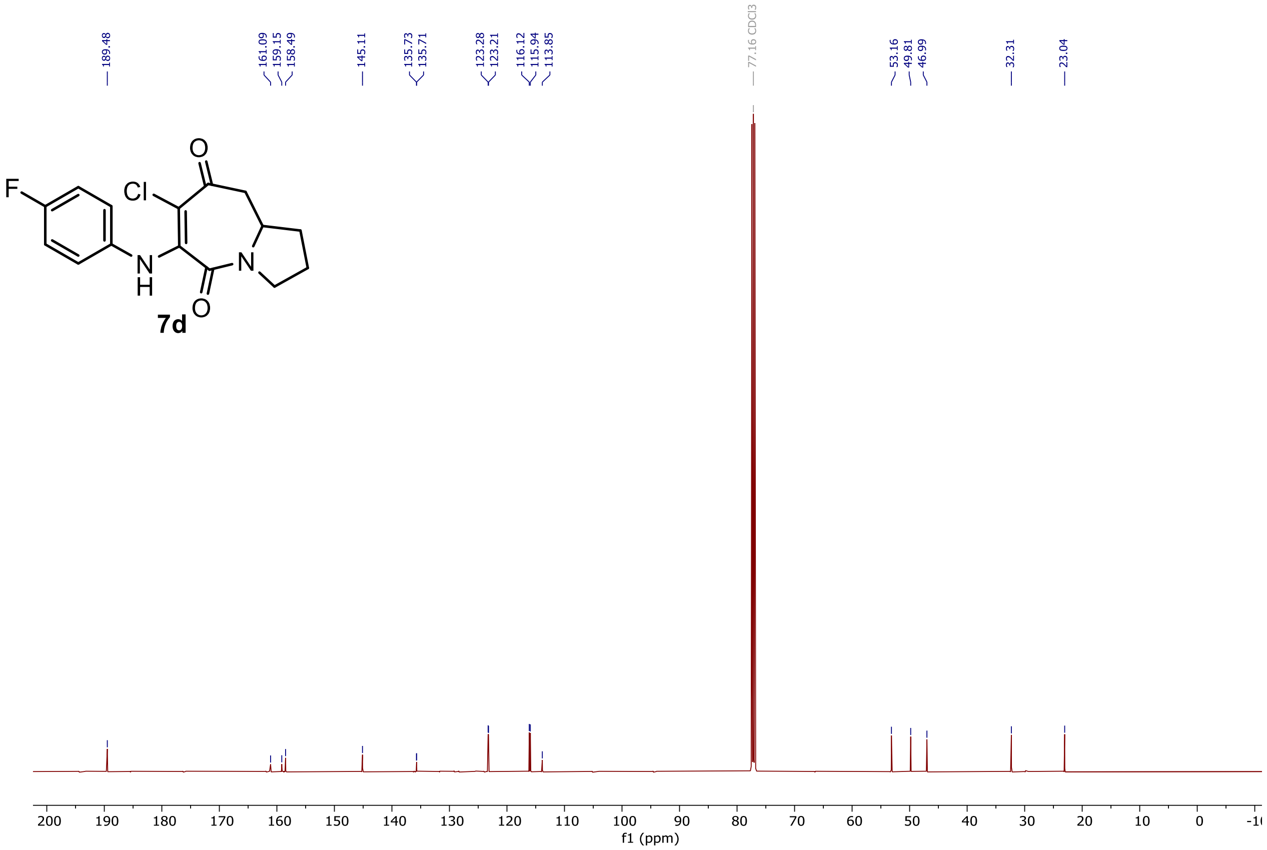

^{19}F NMR, ^{1}H decoupled (337 MHz, $CDCl_3$) of **7d**

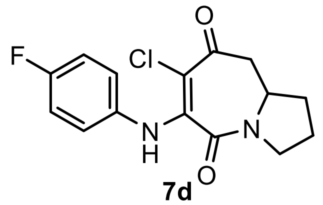

7d

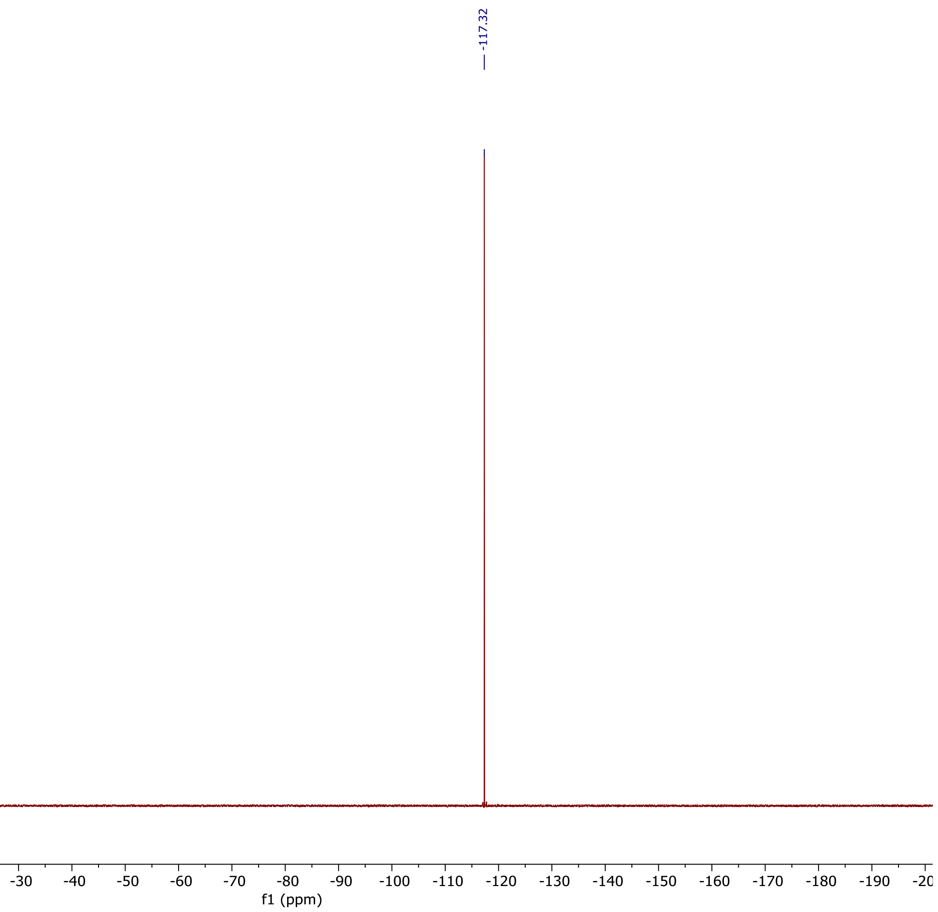

^{1}H NMR (400 MHz, $CDCl_3$) of **7e**

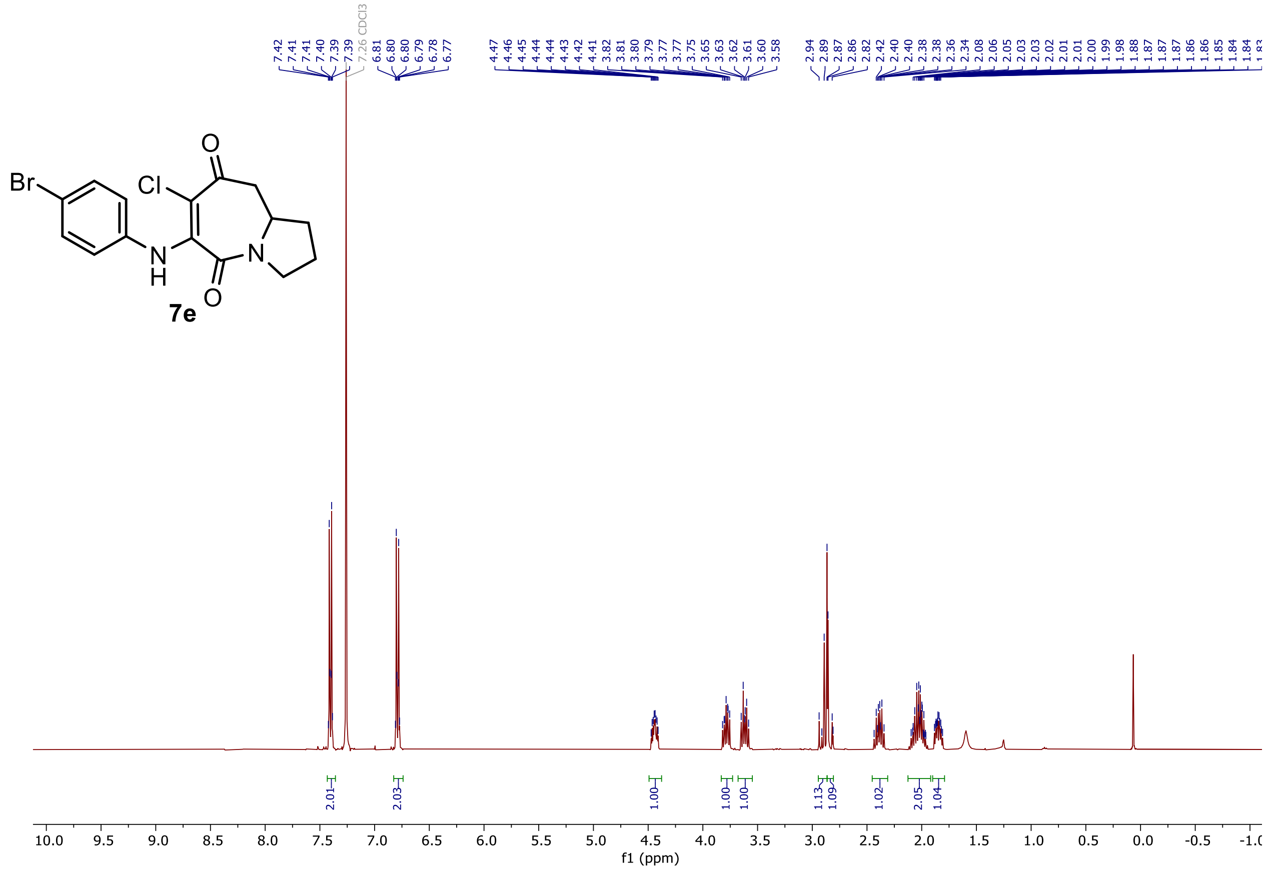

^{13}C NMR (126 MHz, $CDCl_3$) of **7e**

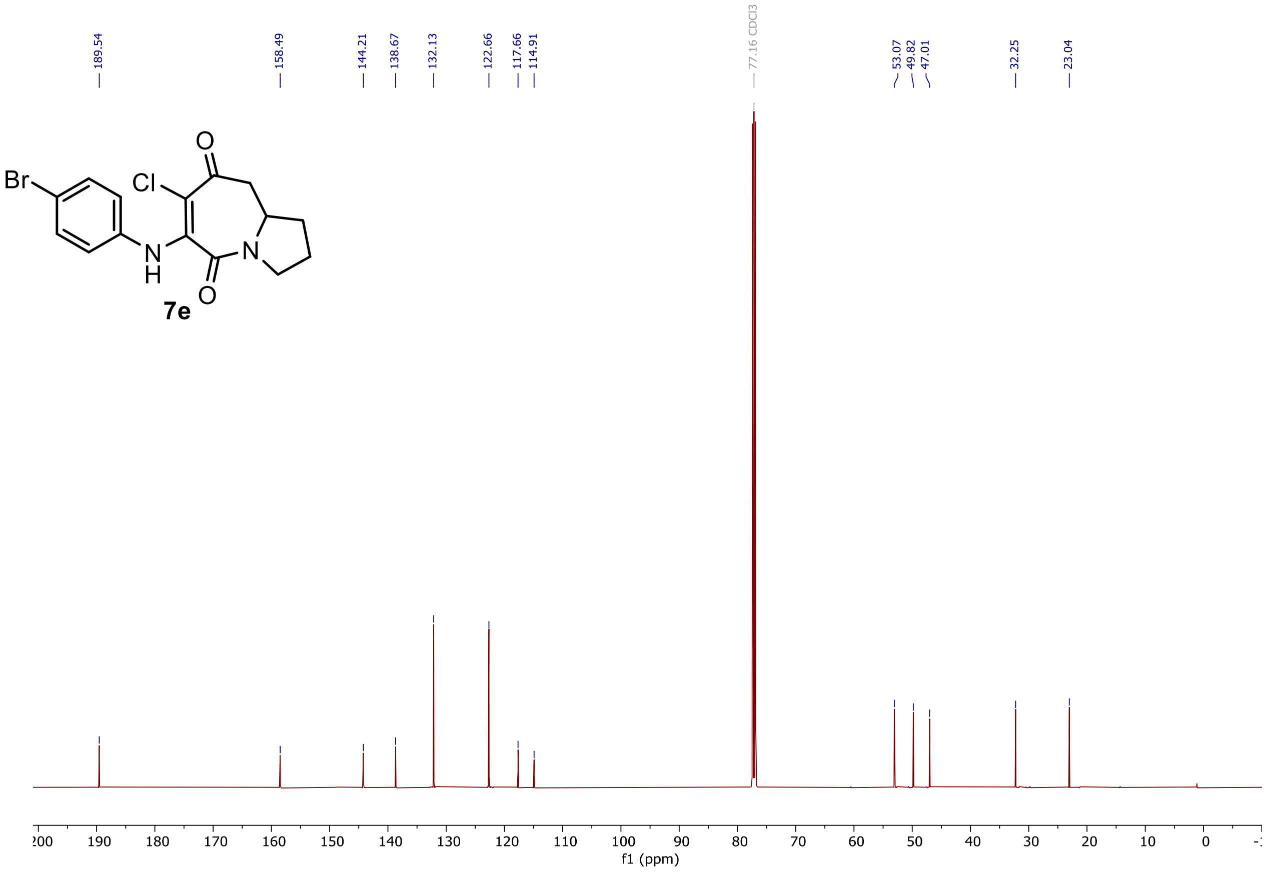

^{1}H NMR (400 MHz, $CDCl_3$) of **7f**

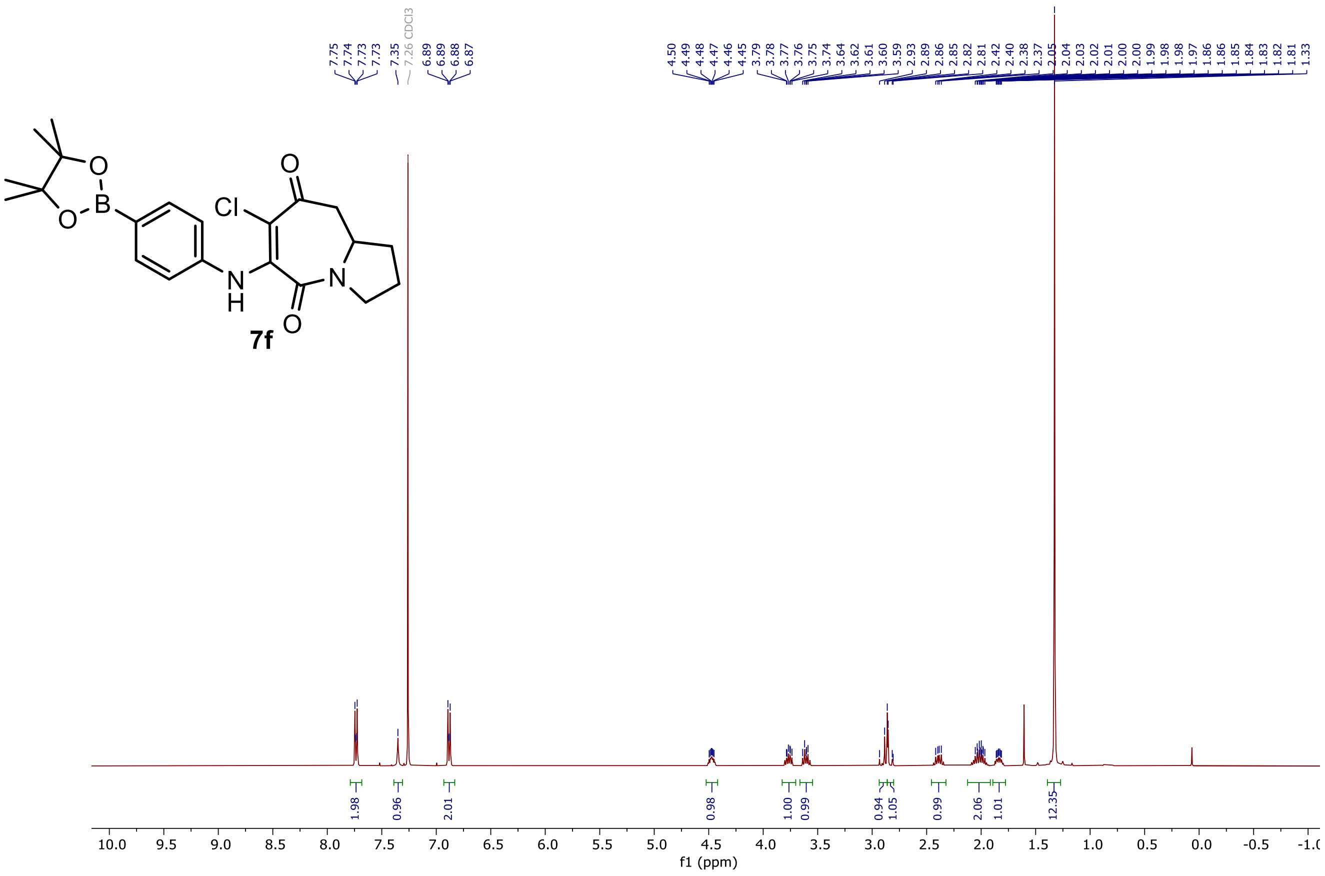

^{13}C NMR (126 MHz, $CDCl_3$) of **7f**

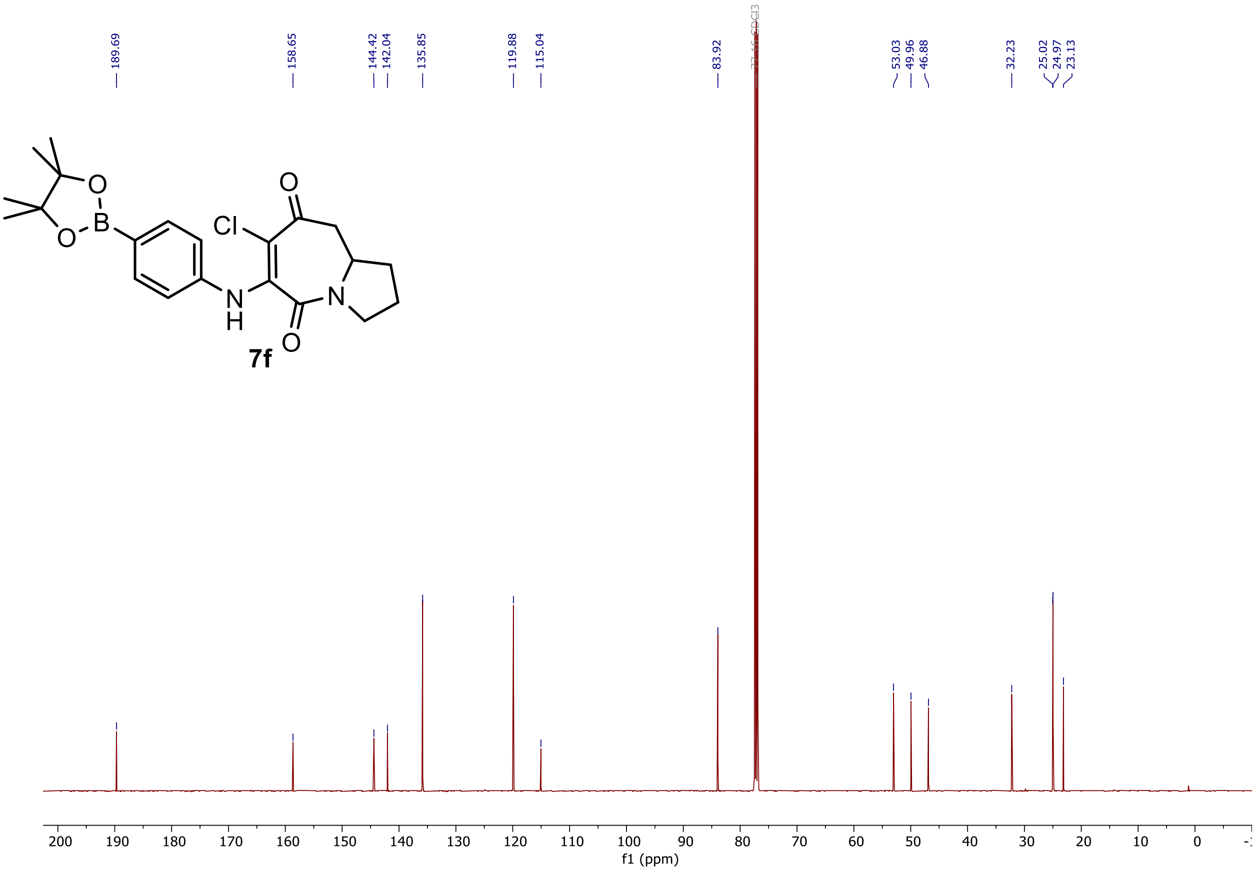

^{11}B NMR (128 MHz, $CDCl_3$) of **7f**

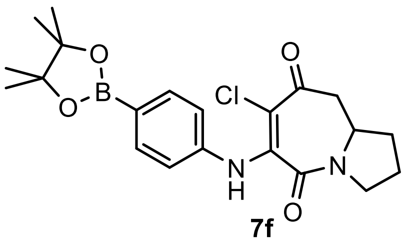

7f

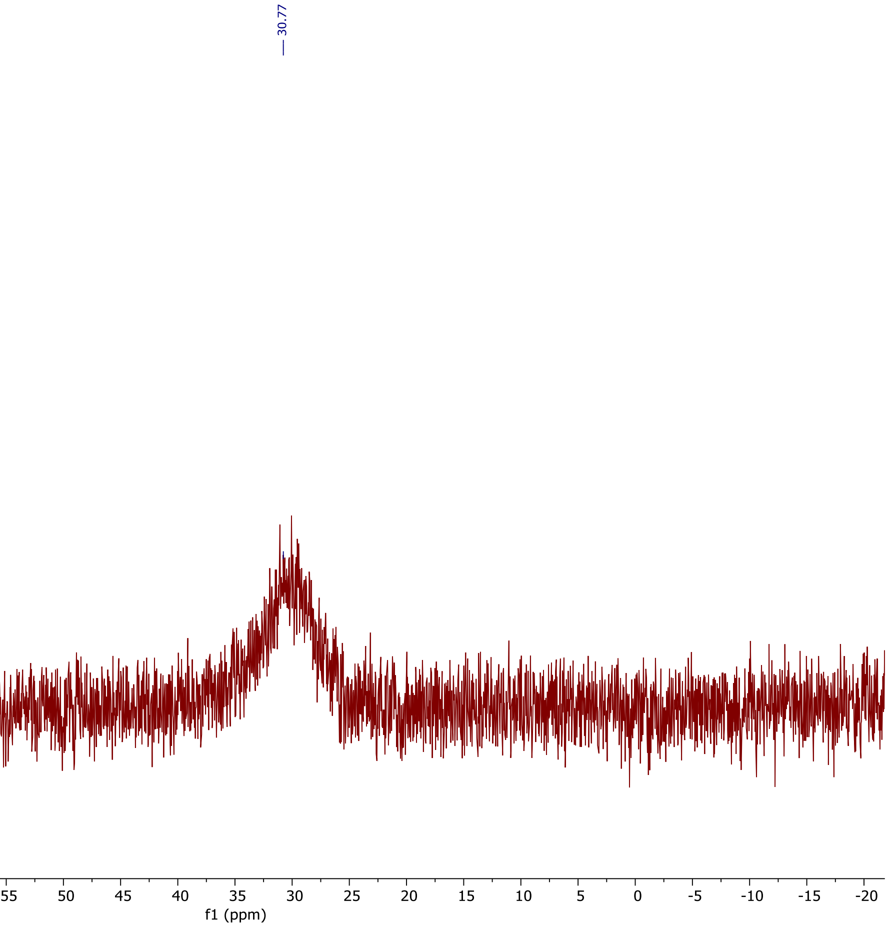

^{1}H NMR (400 MHz, DMSO-d_6) of **7g**

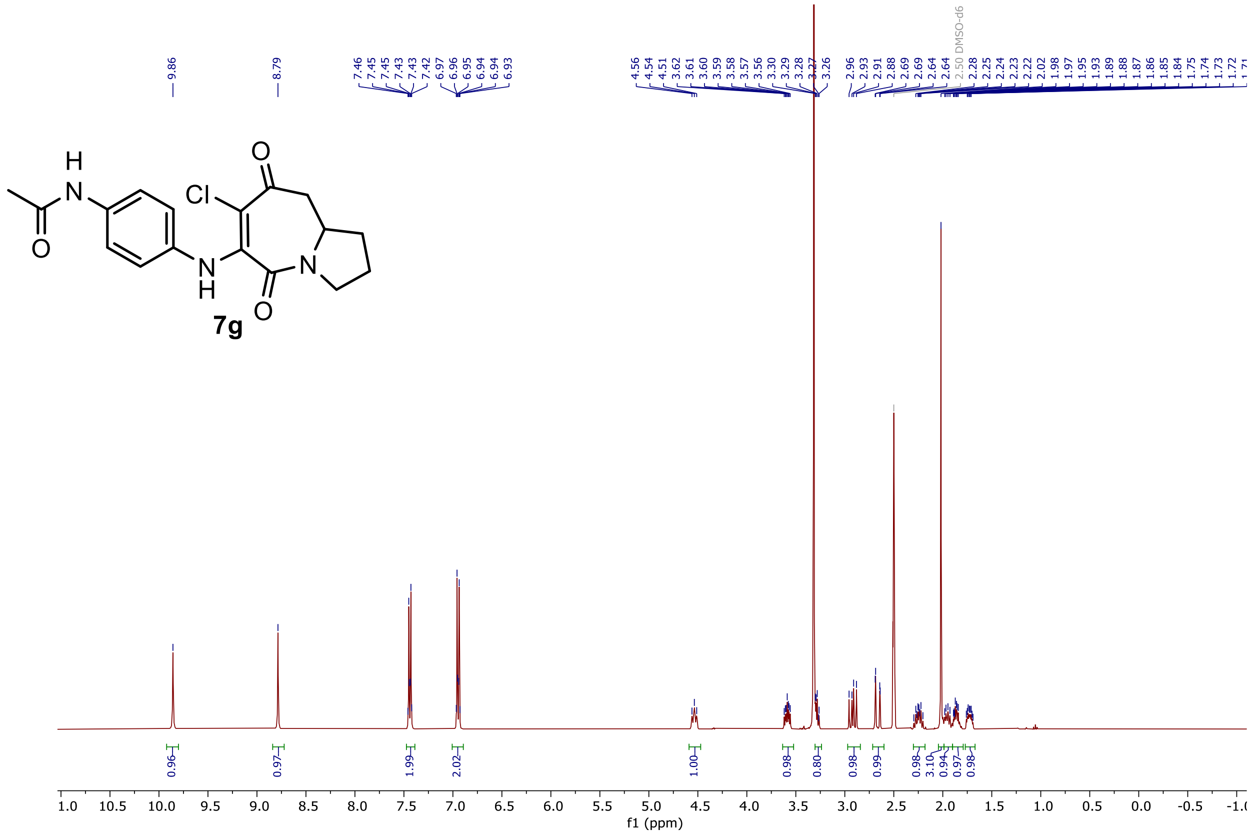

^{13}C NMR (126 MHz, DMSO-d_6) of **7g**

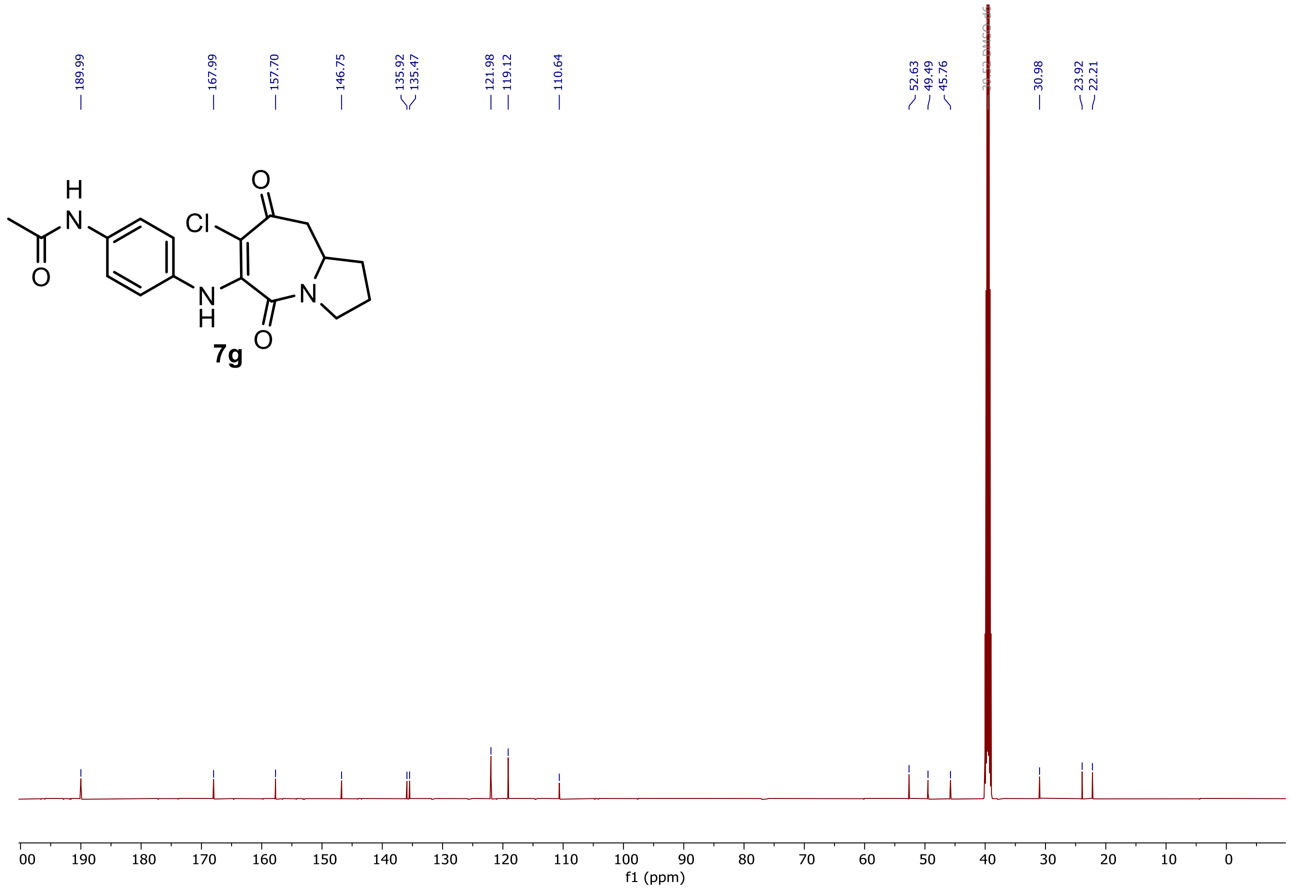

^{1}H NMR (400 MHz, $CDCl_3$) of **7h**

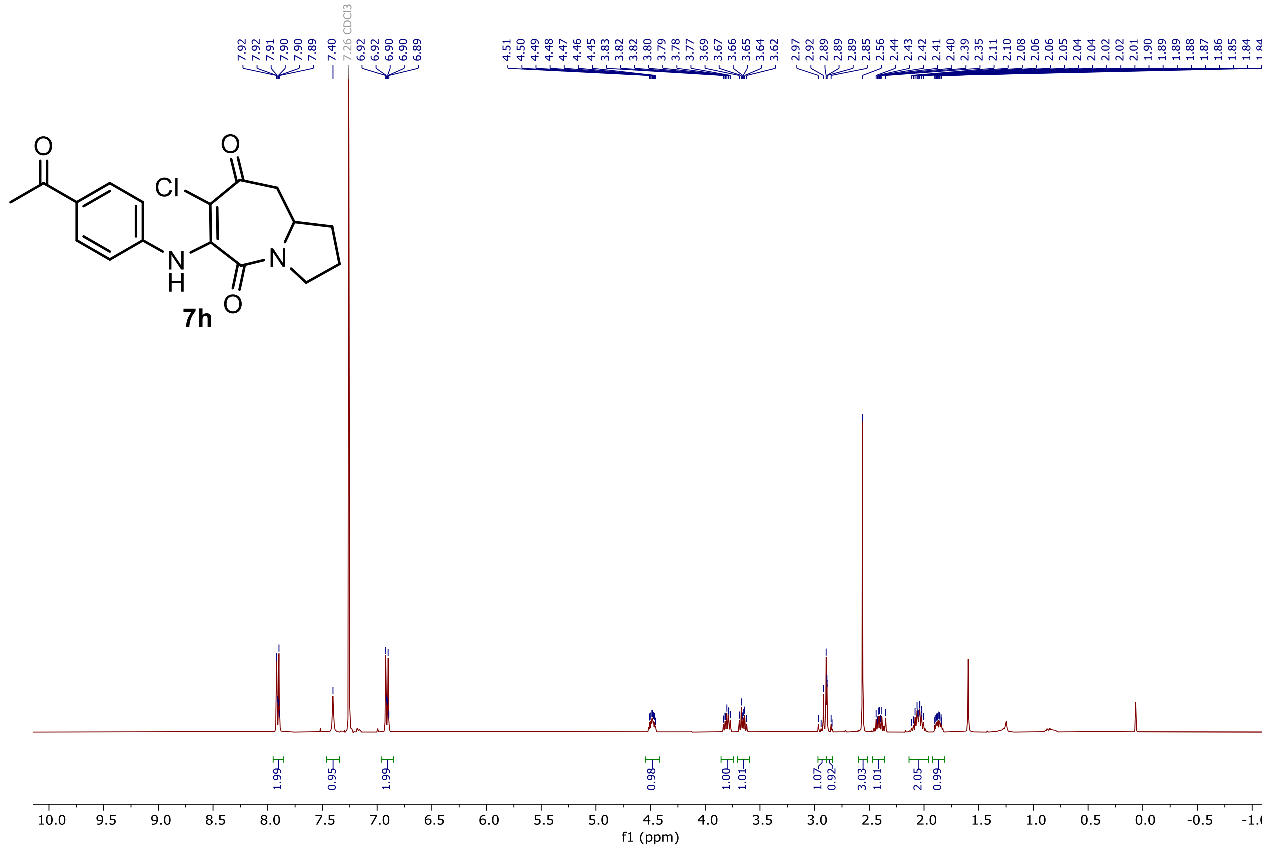

^{13}C NMR (126 MHz, $CDCl_3$) of **7h**

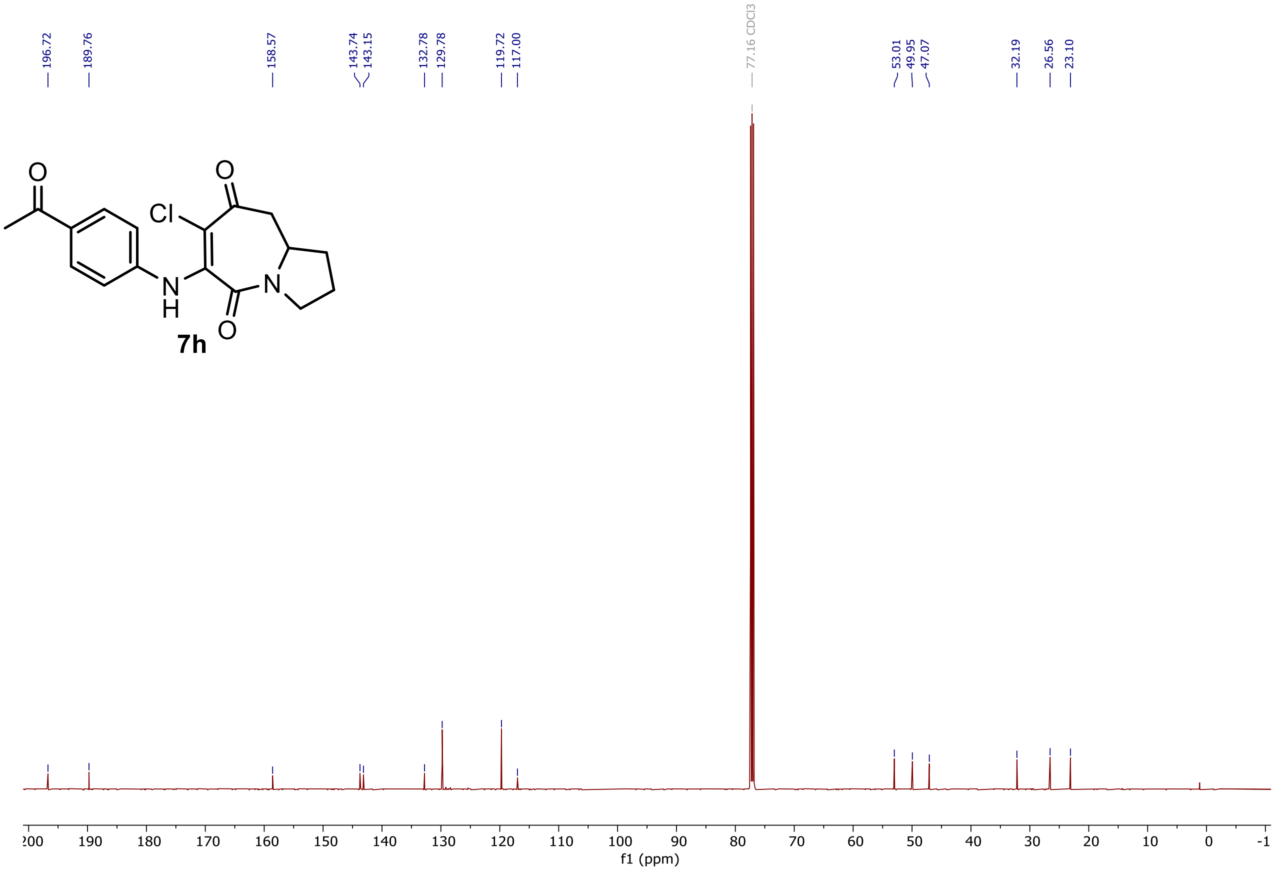

^{1}H NMR (400 MHz, $CDCl_3$) of **7i**

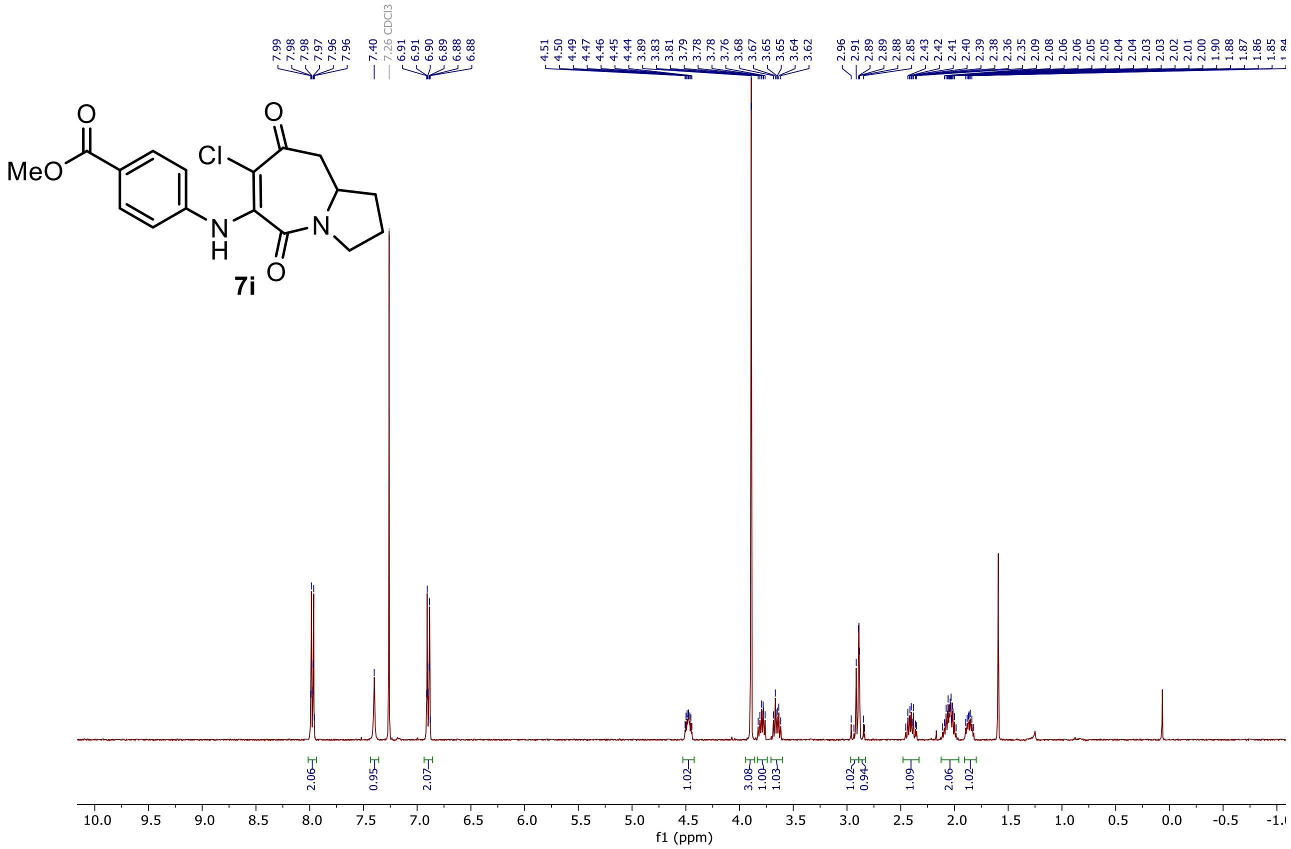

^{13}C NMR (126 MHz, $CDCl_3$) of **7i**

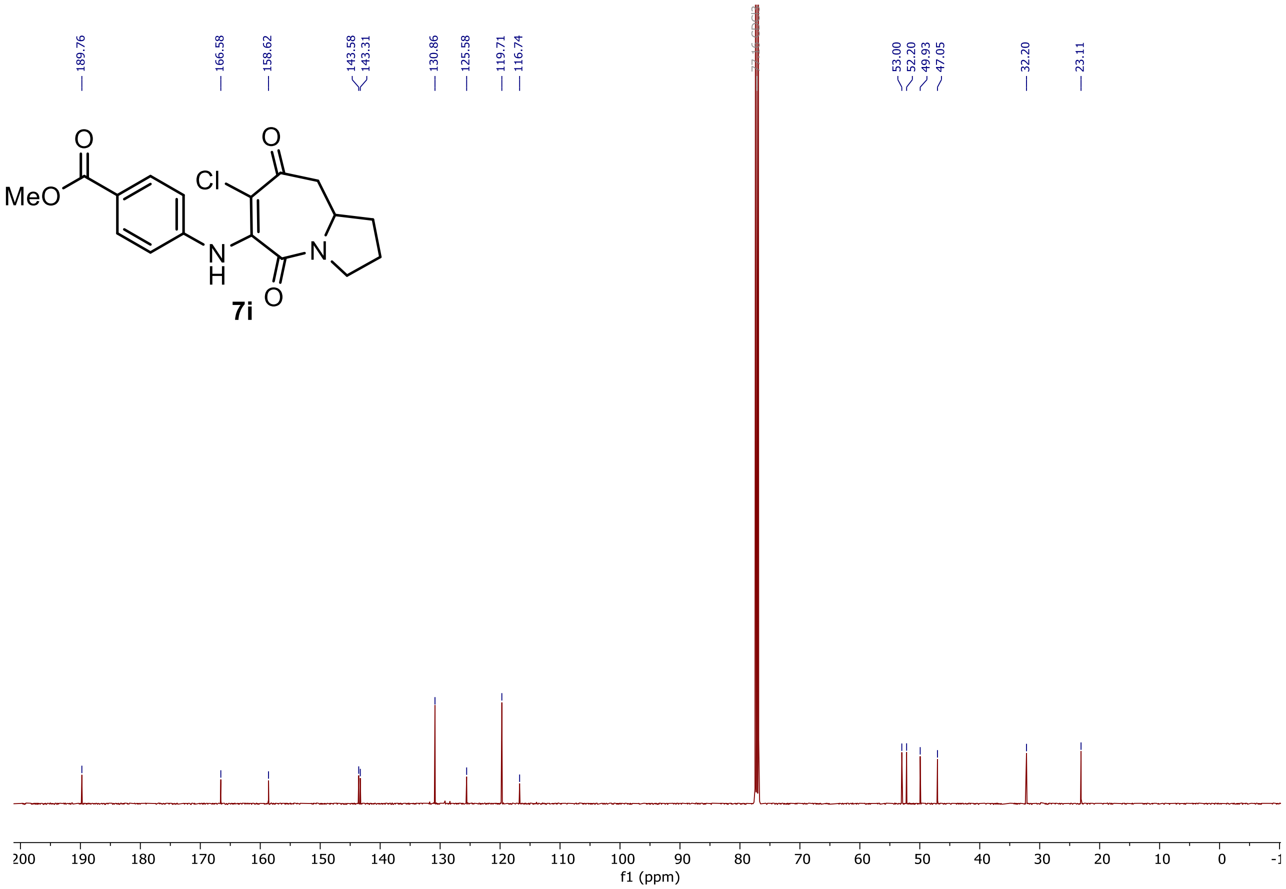

^{1}H NMR (500 MHz, $CDCl_3$) of **7j**

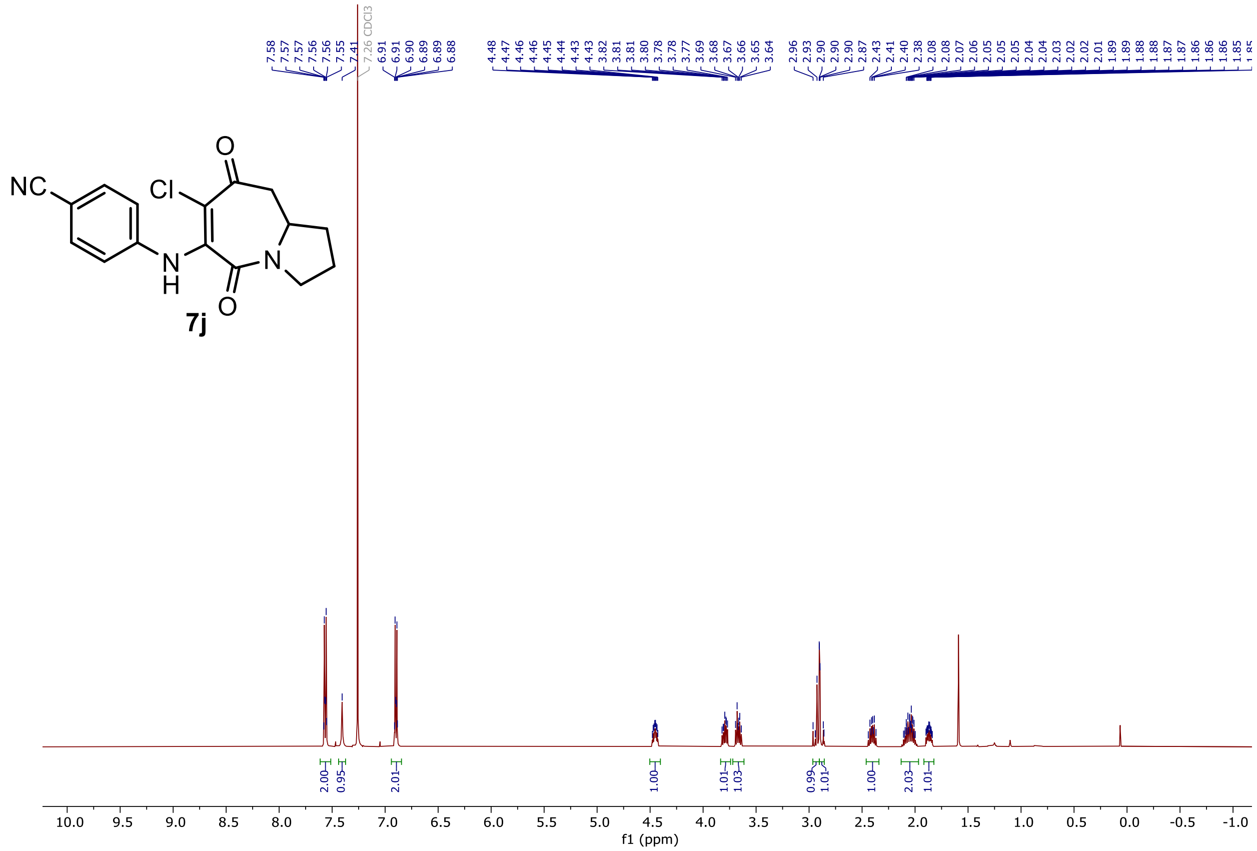

^{13}C NMR (126 MHz, $CDCl_3$) of **7j**

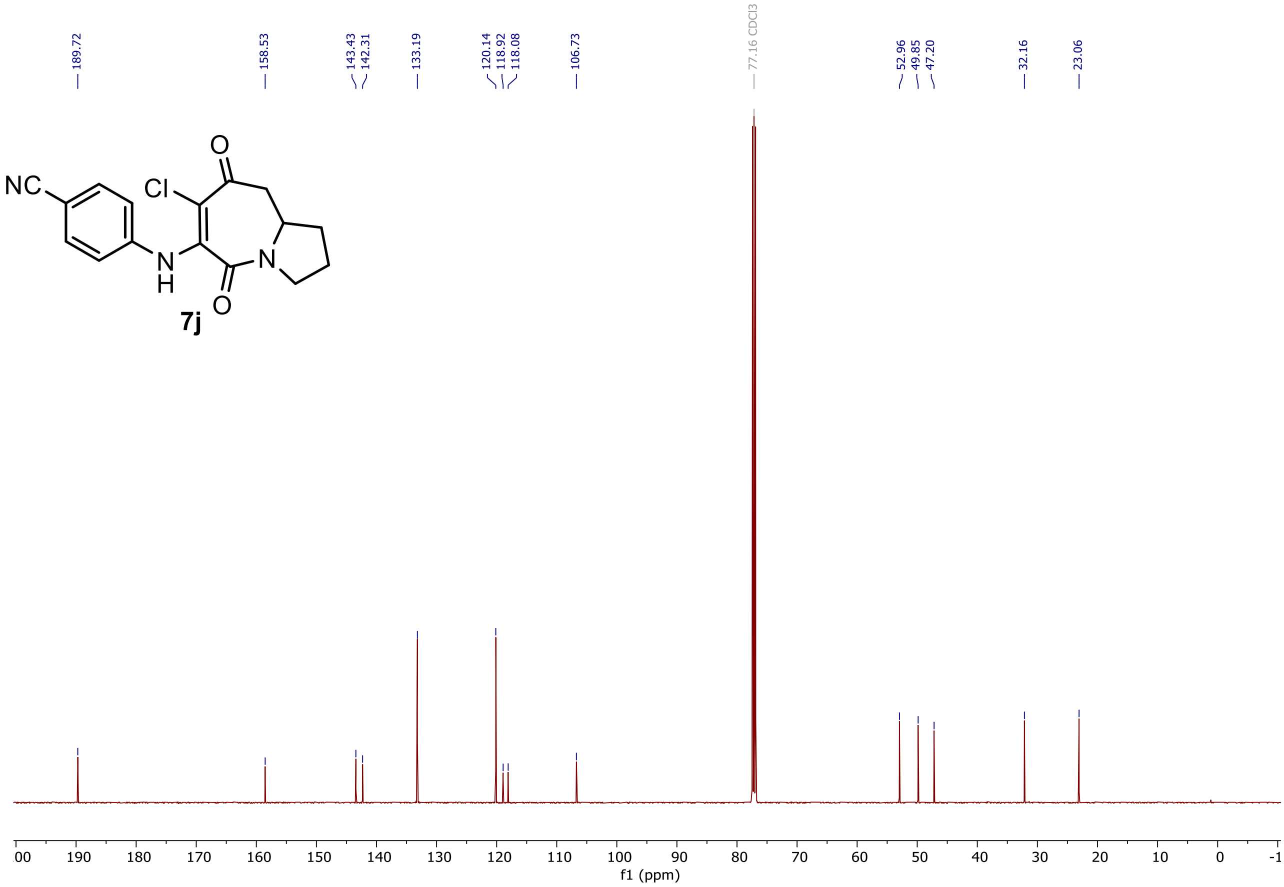

^{1}H NMR (400 MHz, $CDCl_3$) of **7k**

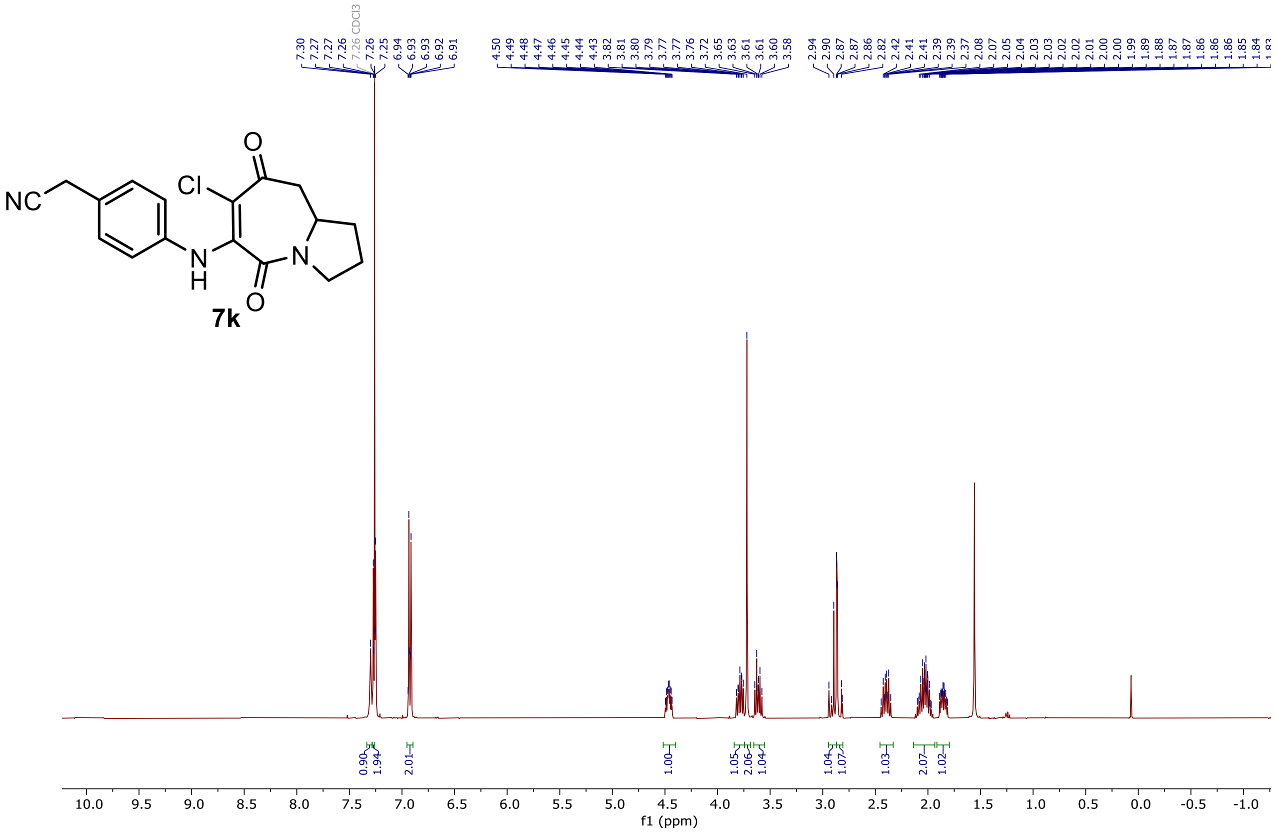

^{13}C NMR (126 MHz, $CDCl_3$) of **7k**

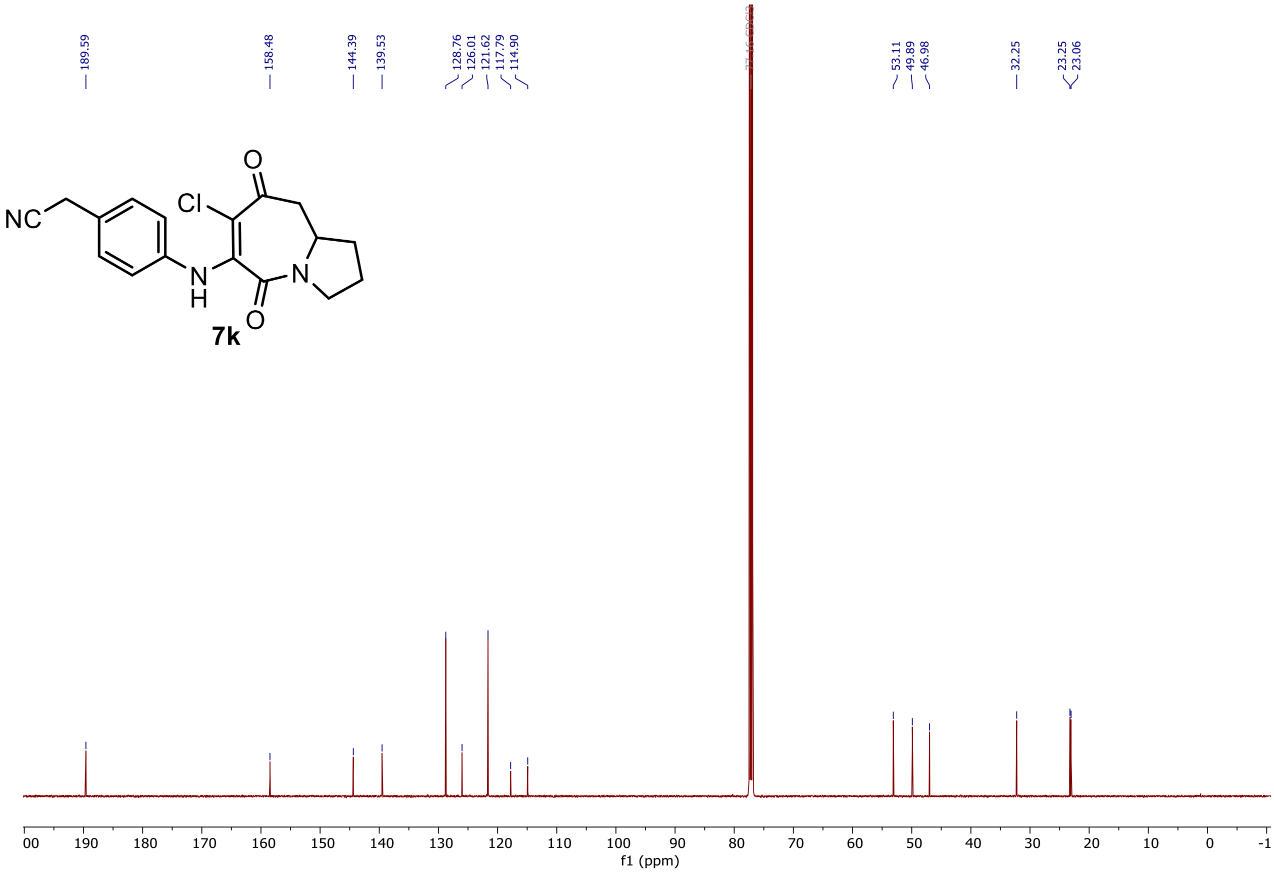

^{1}H NMR (400 MHz, DMSO-d_6) of **7l**

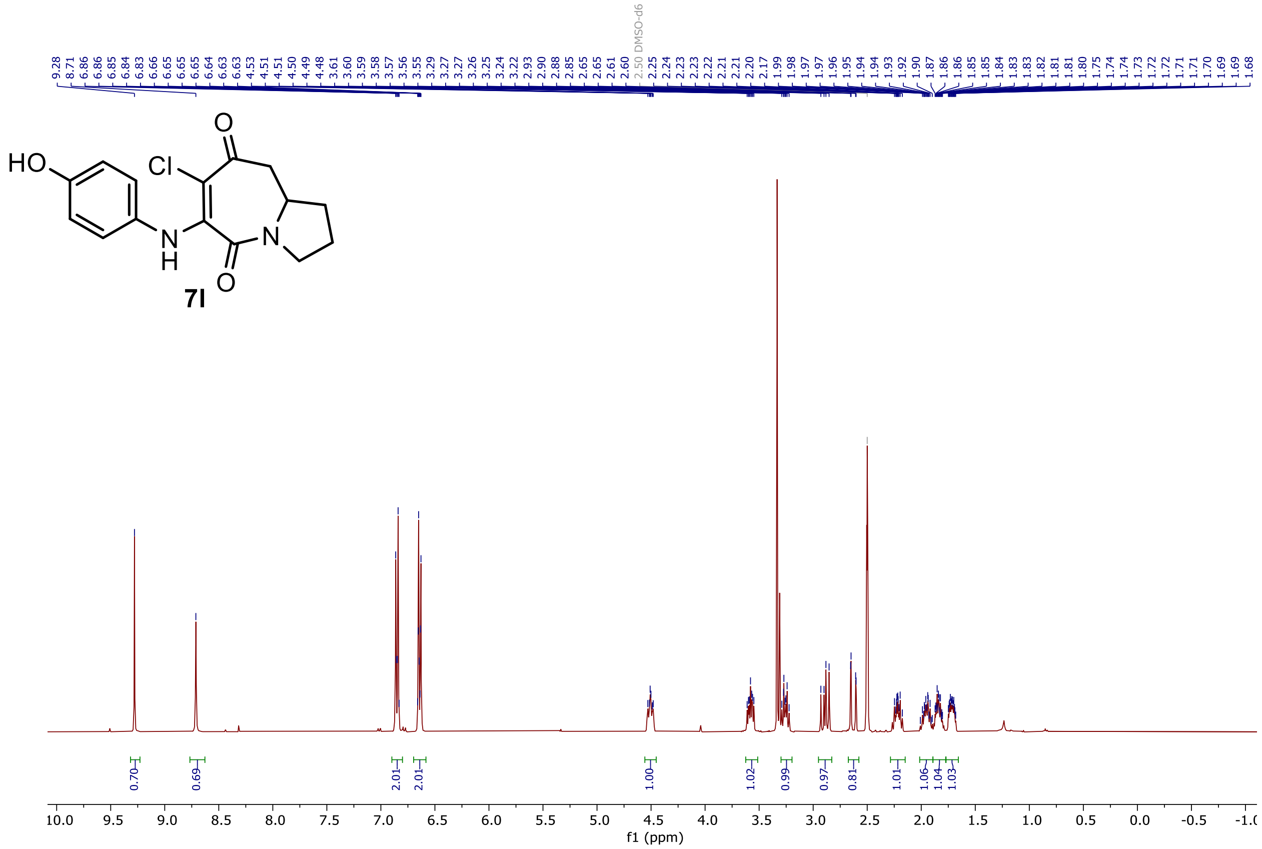

^{13}C NMR (101 MHz, DMSO-d_6) of **7l**

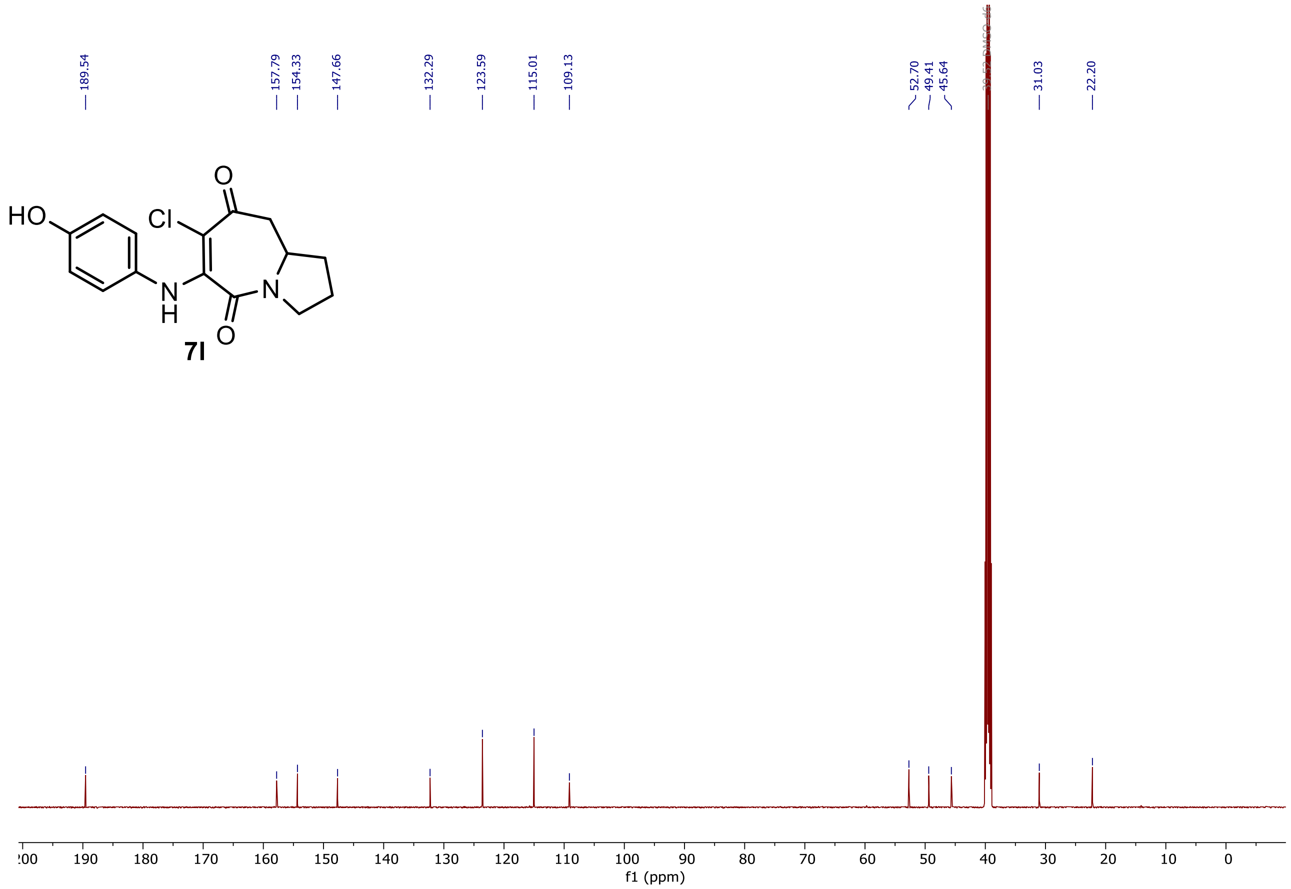

^{1}H NMR (400 MHz, CDCl$_3$) of **7m**

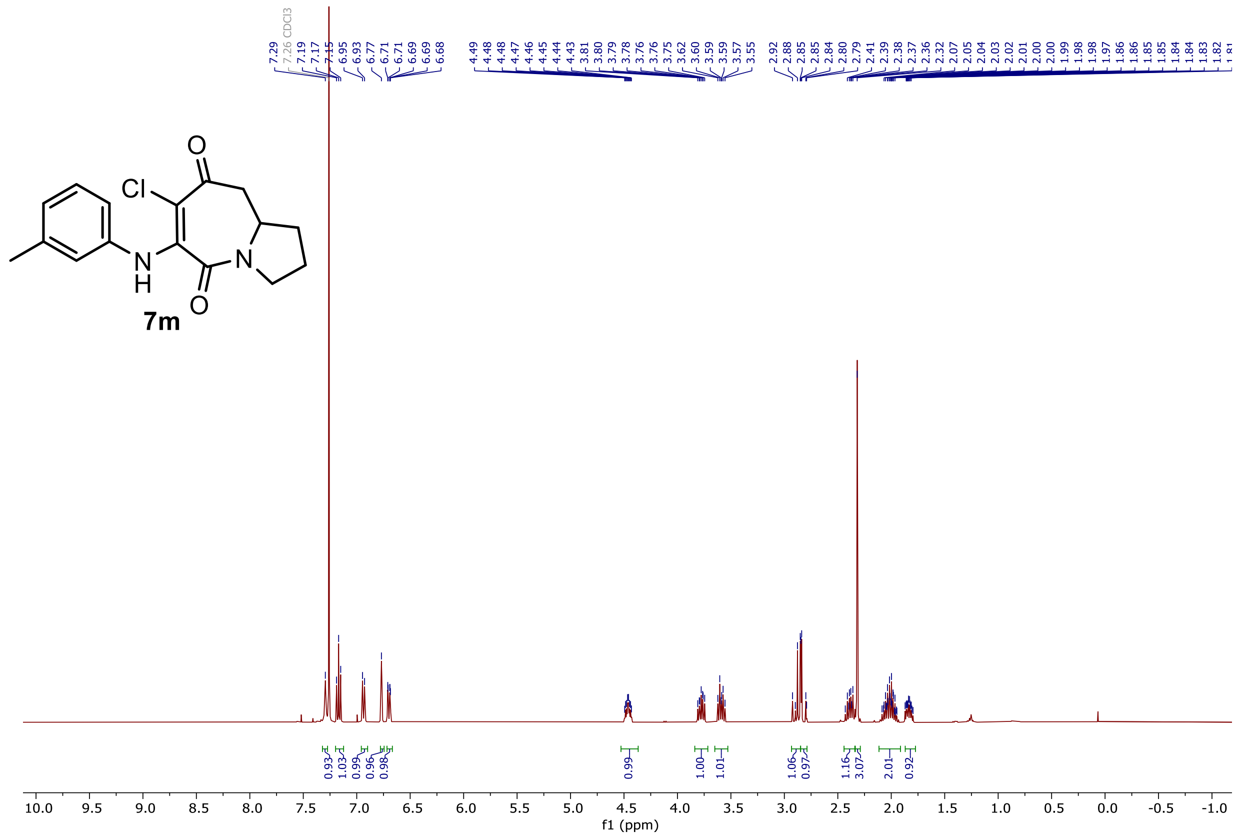

^{13}C NMR (126 MHz, CDCl$_3$) of **7m**

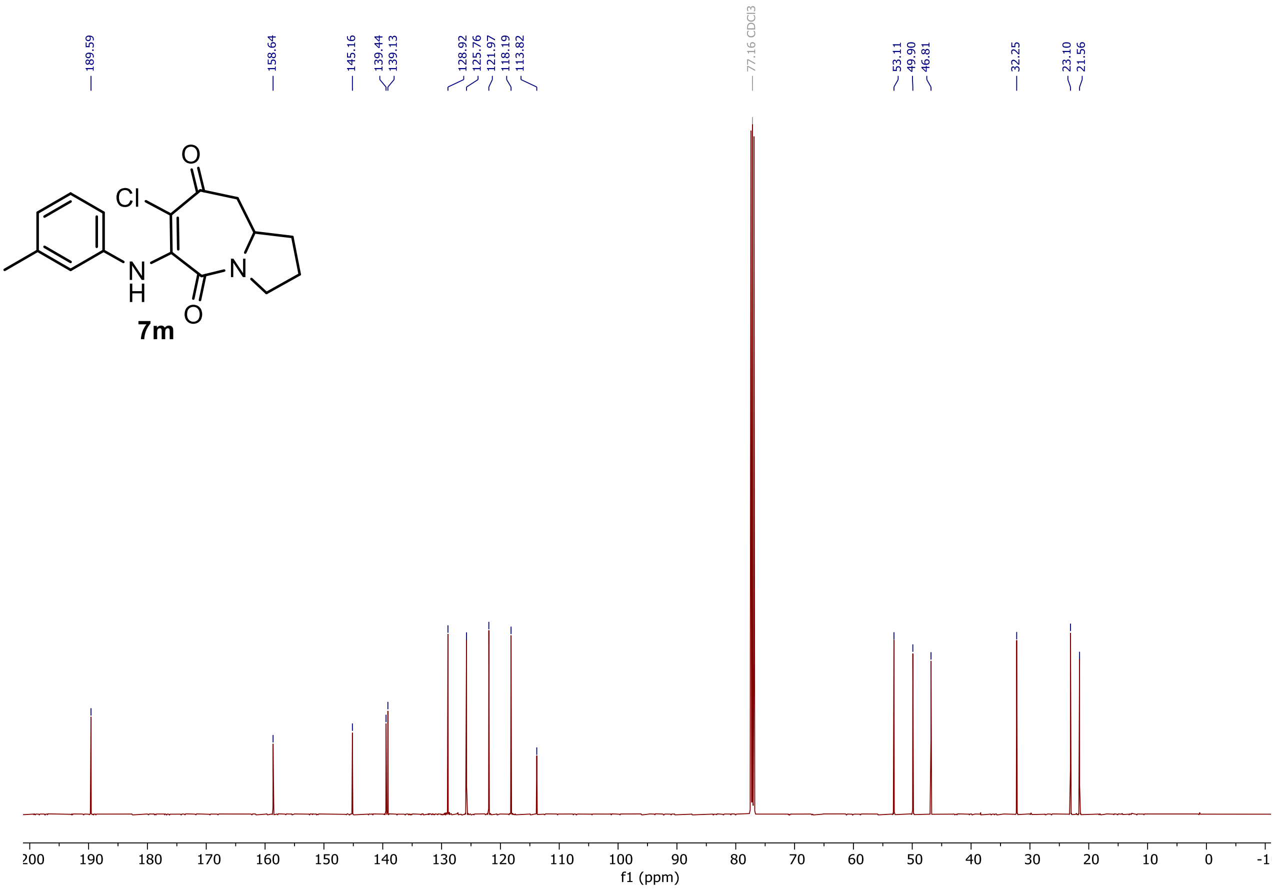

^{1}H NMR (500 MHz, $CDCl_3$) of **7n**

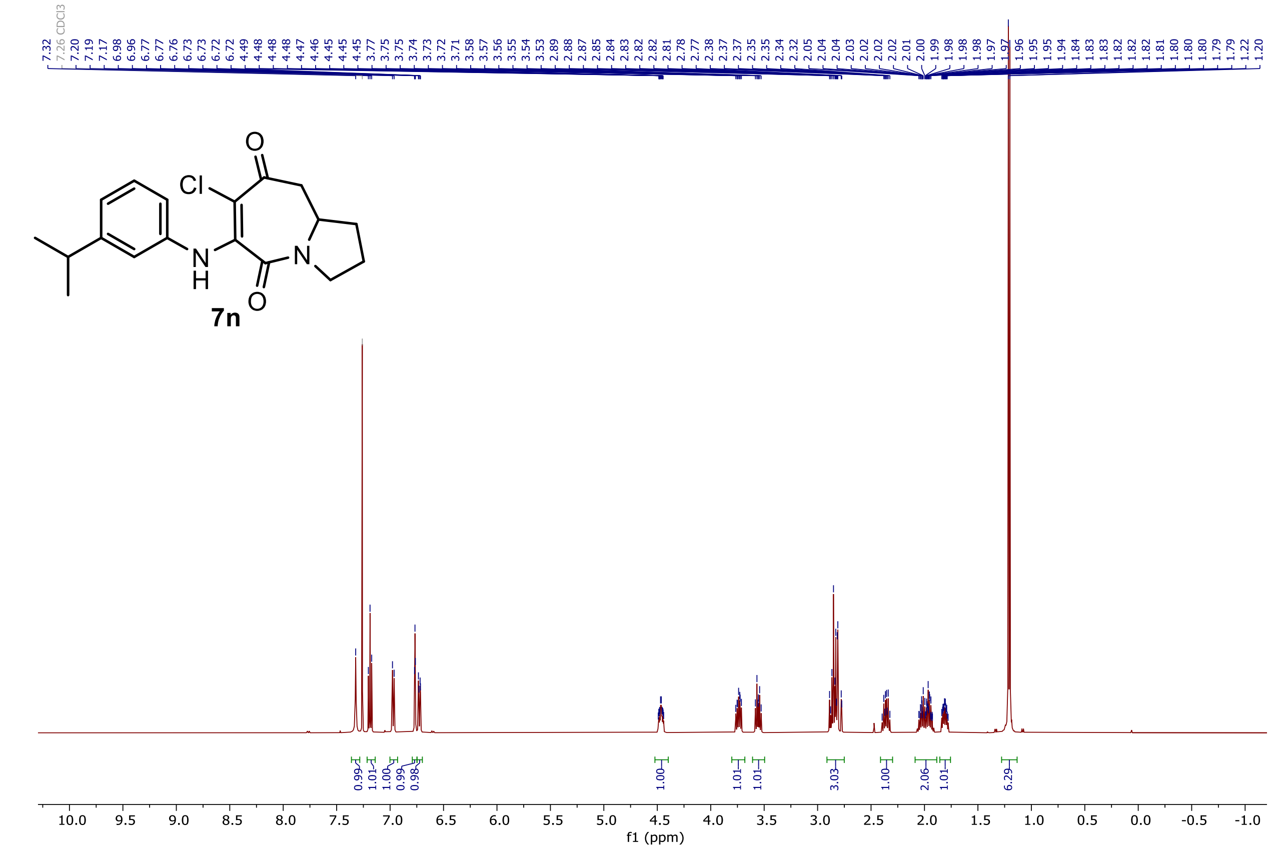

^{13}C NMR (126 MHz, $CDCl_3$) of **7n**

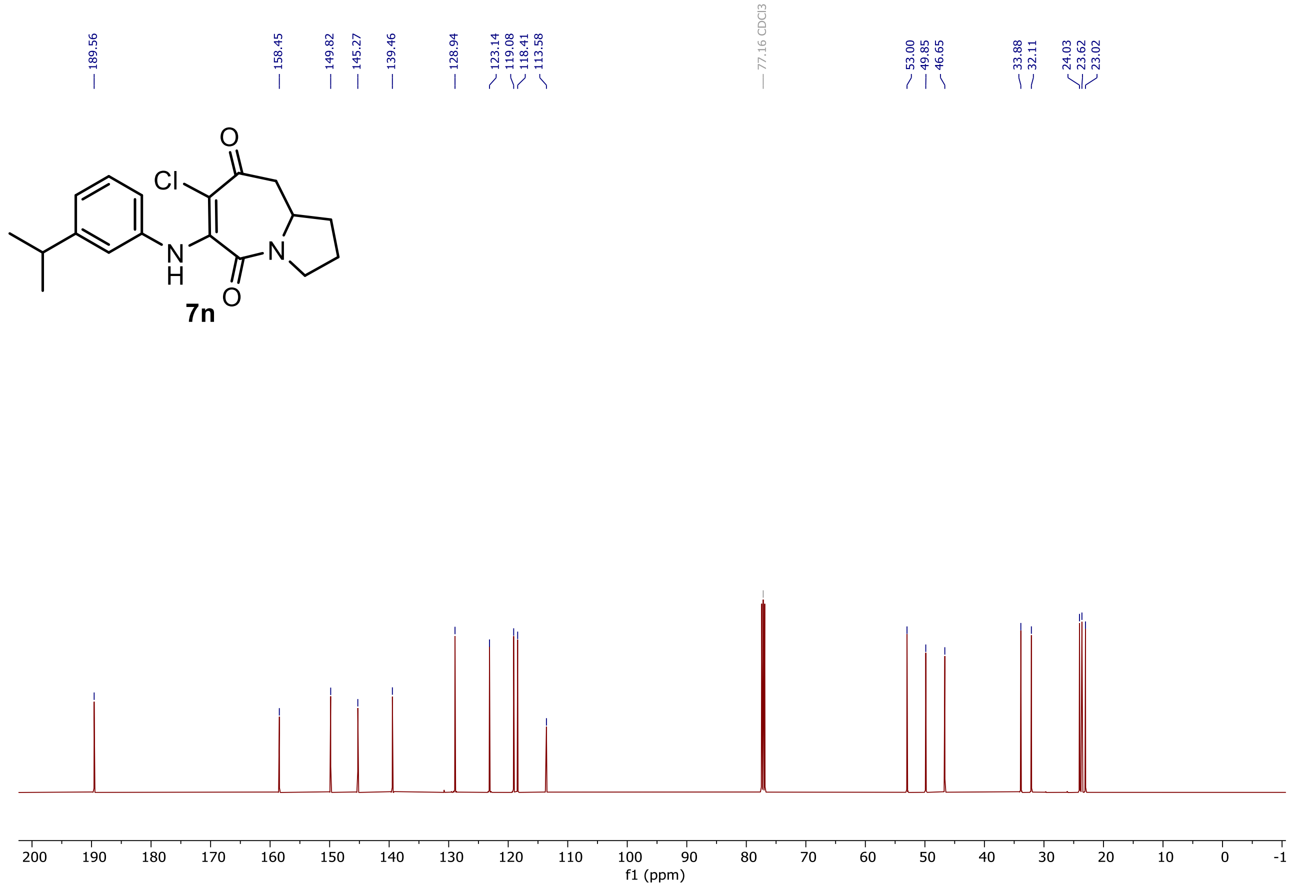

^1H NMR (400 MHz, $CDCl_3$) of **7o**

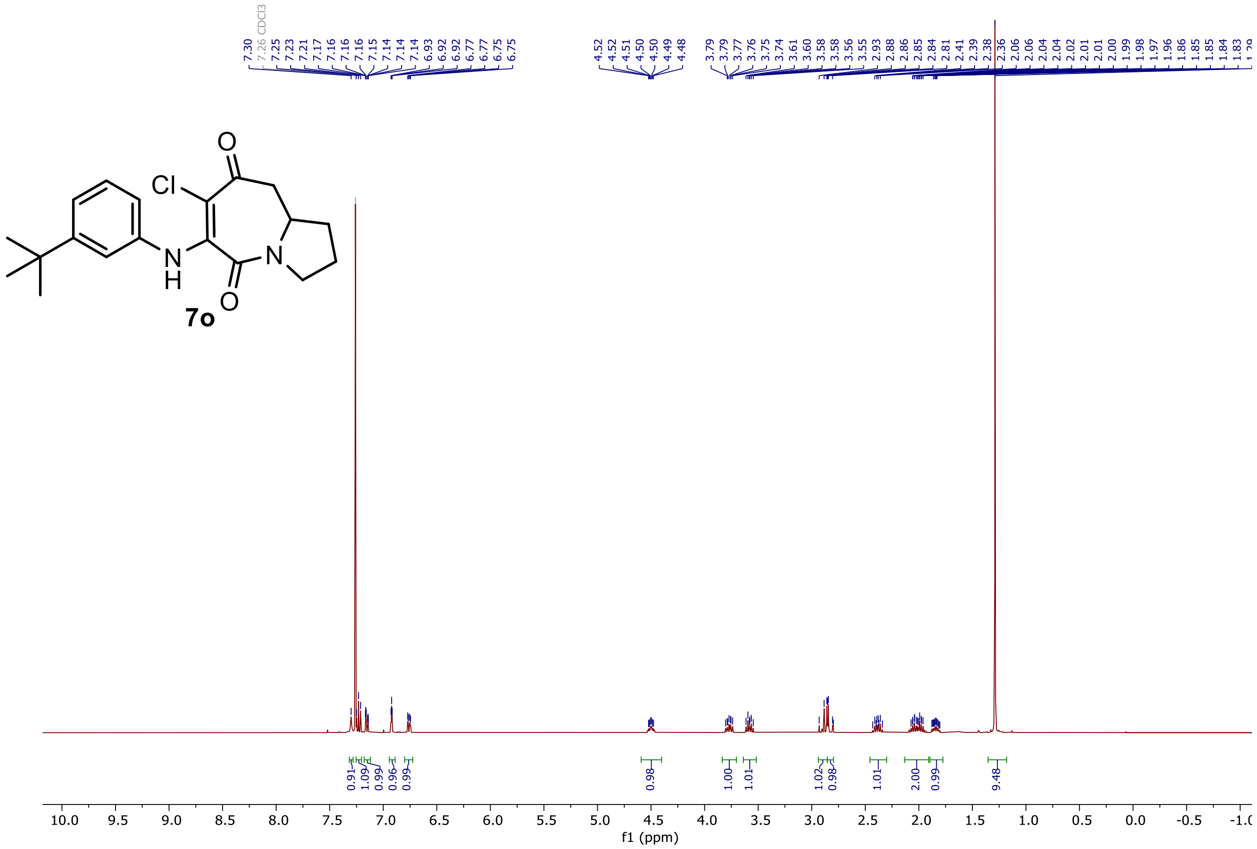

^{13}C NMR (126 MHz, $CDCl_3$) of **7o**

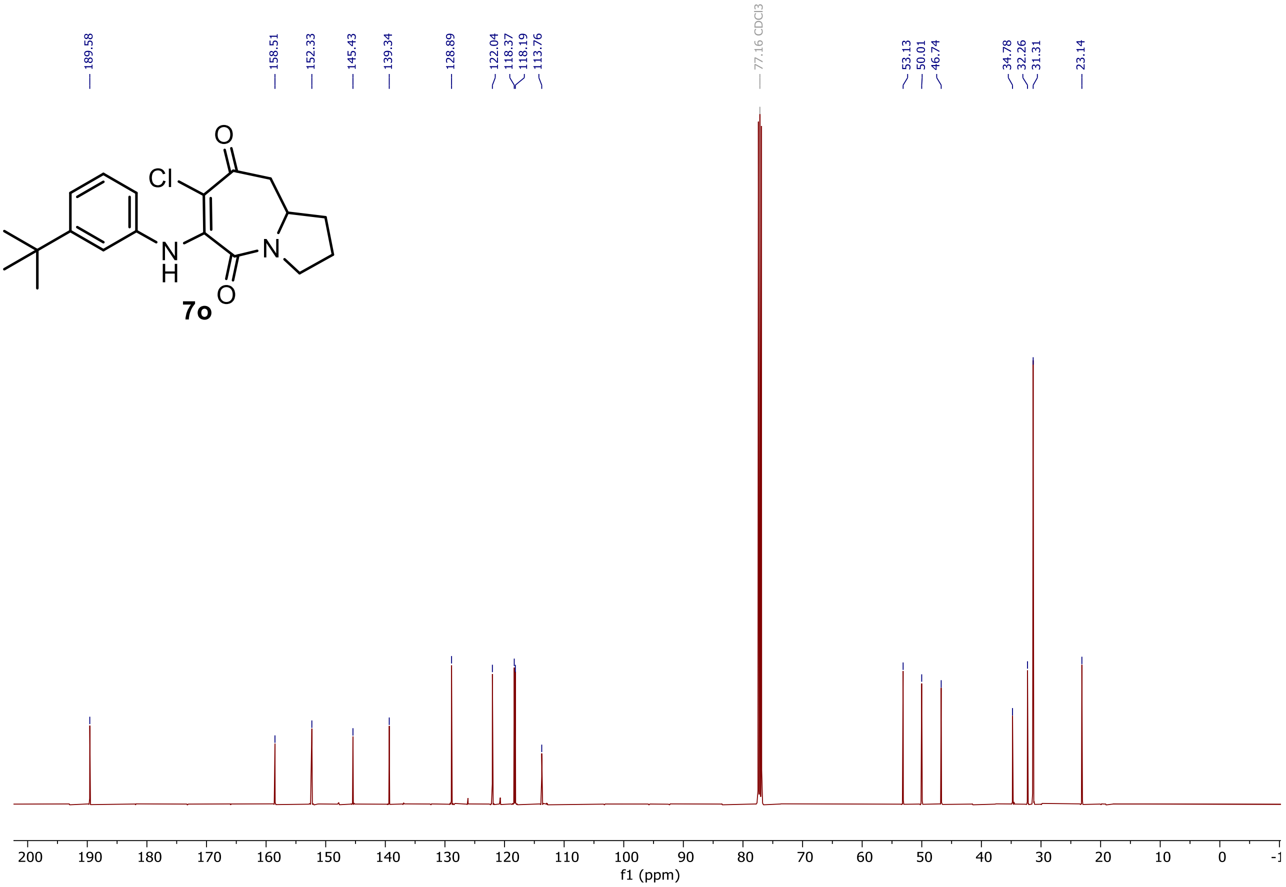

^{1}H NMR (400 MHz, $CDCl_3$) of **7p**

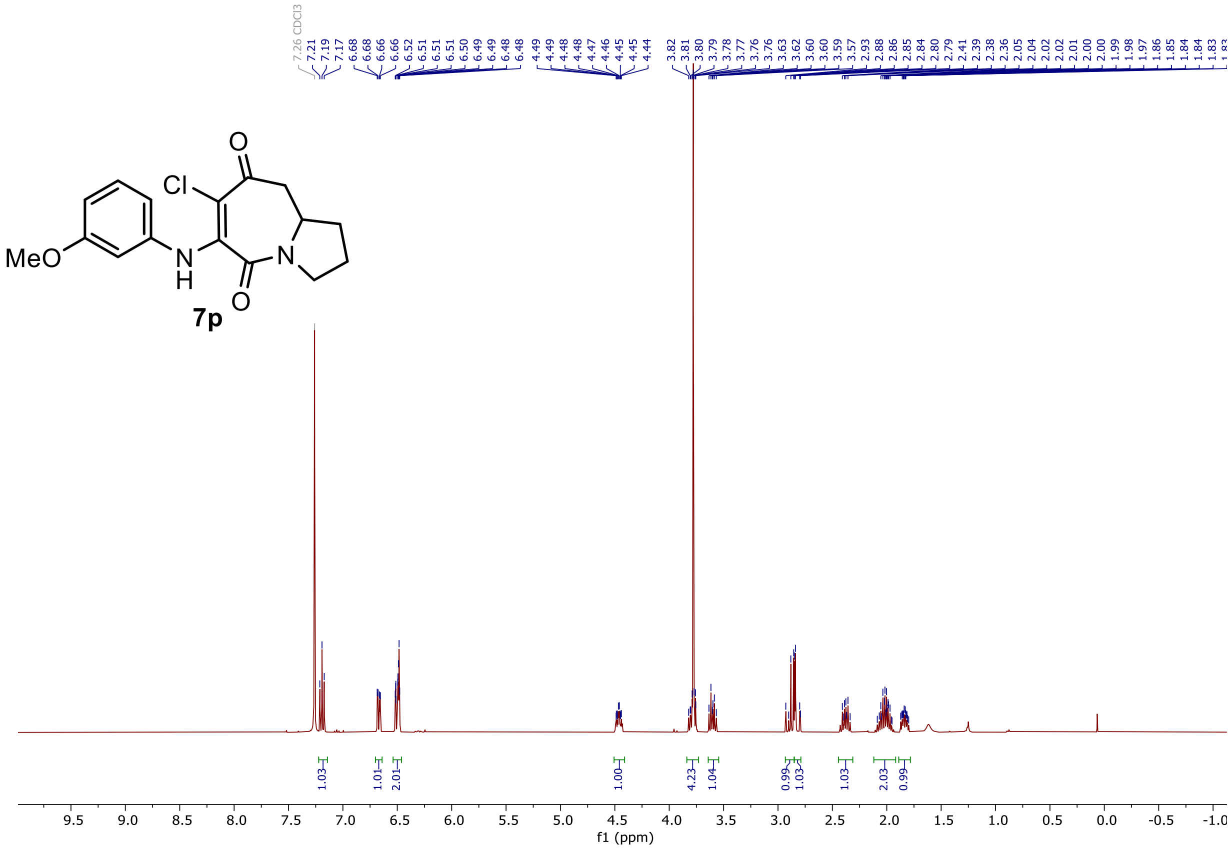

^{13}C NMR (126 MHz, $CDCl_3$) of **7p**

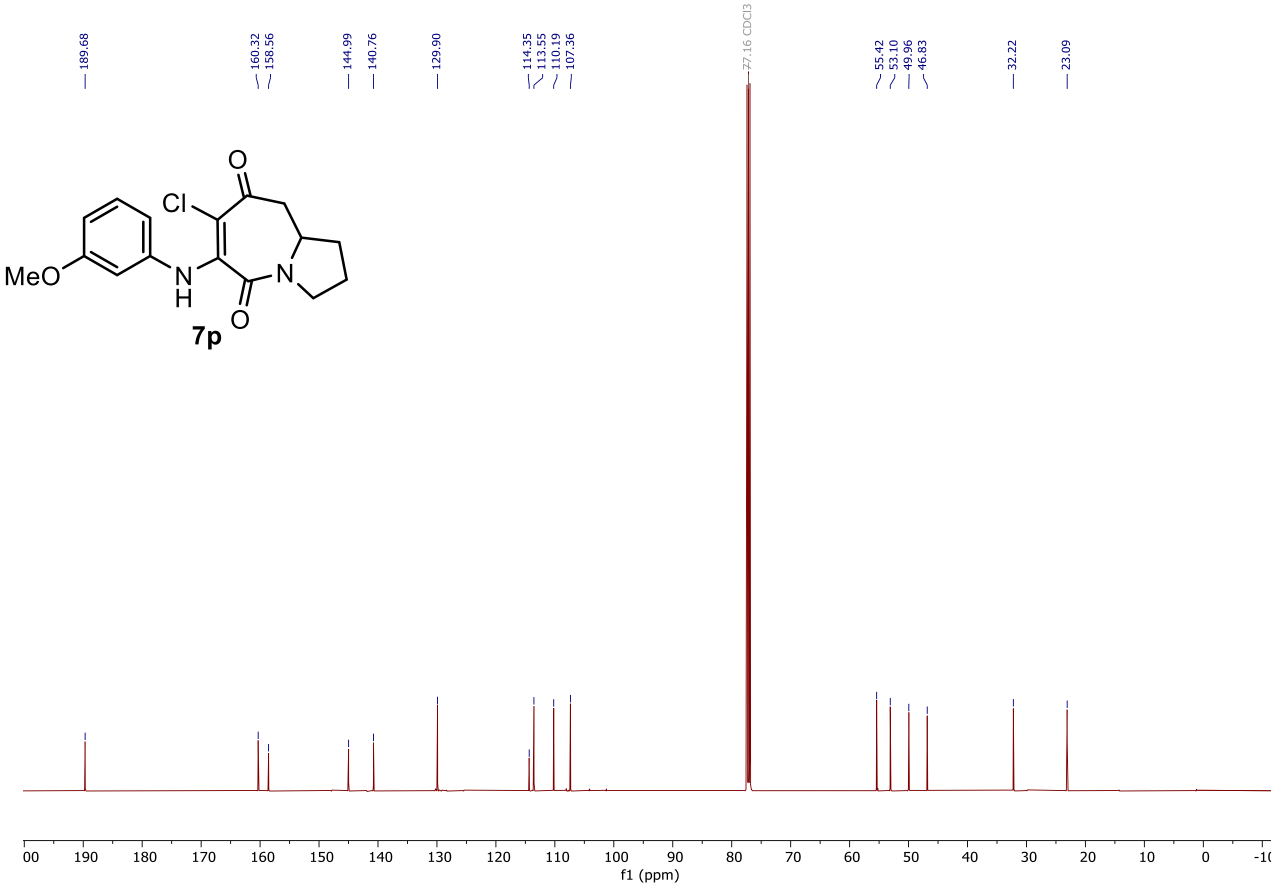

^{1}H NMR (400 MHz, $CDCl_3$) of **7q**

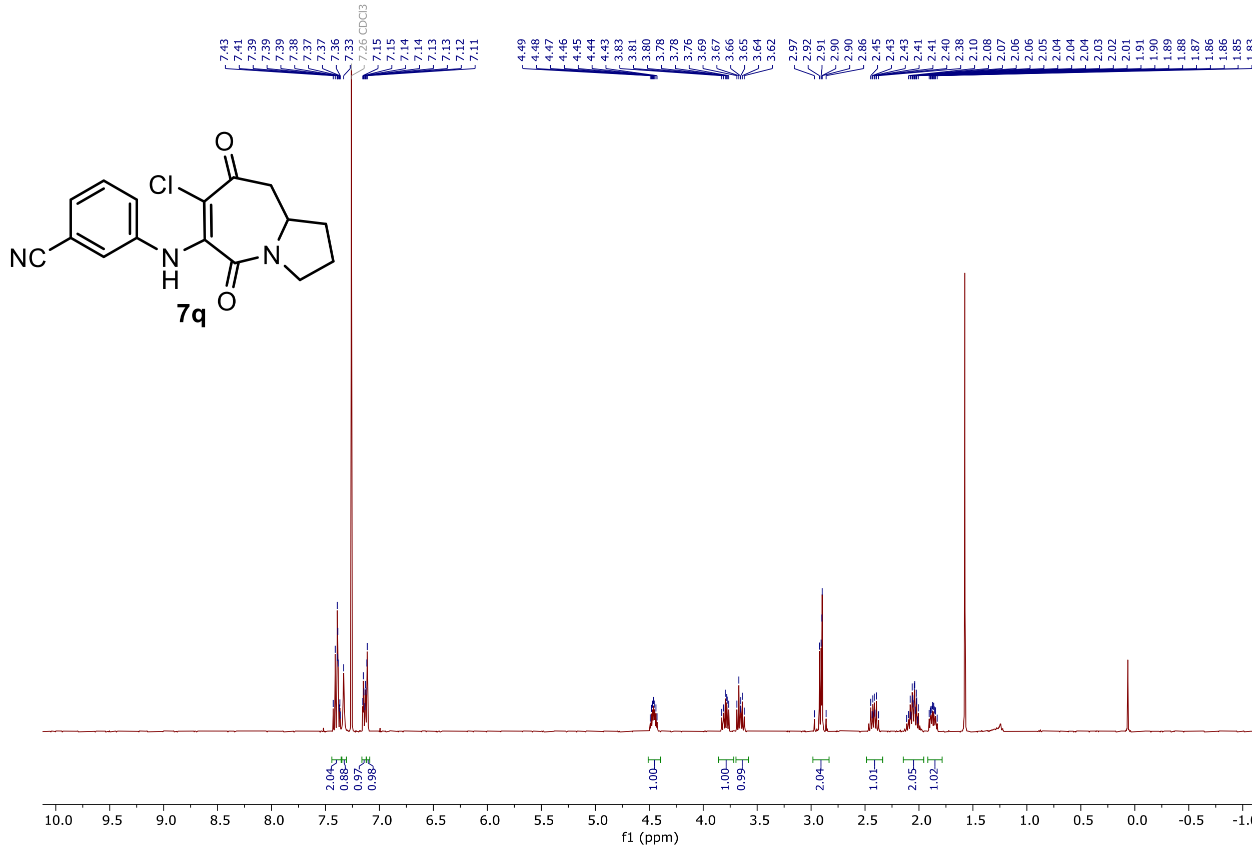

^{13}C NMR (126 MHz, $CDCl_3$) of **7q**

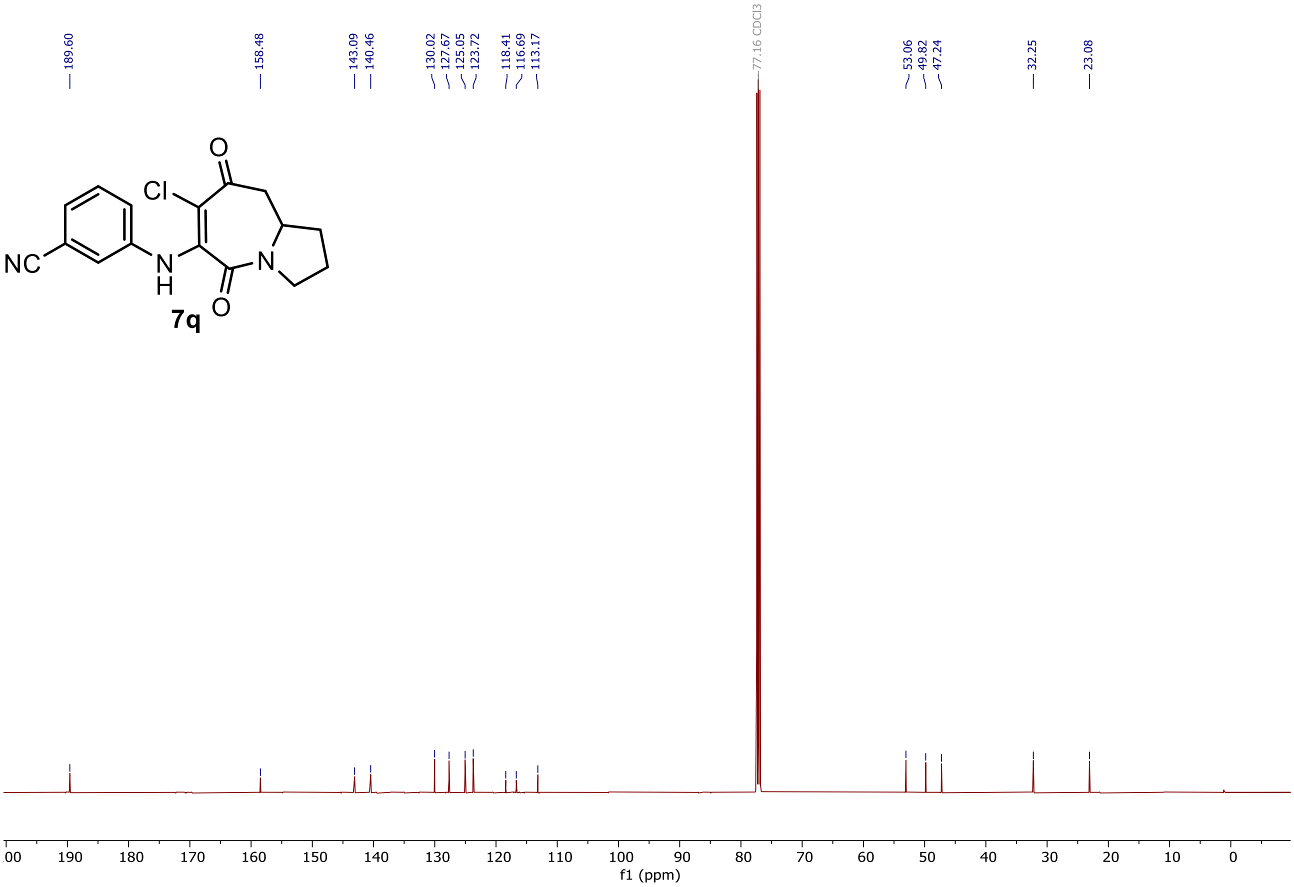

^{1}H NMR (400 MHz, $CDCl_3$) of **7r**

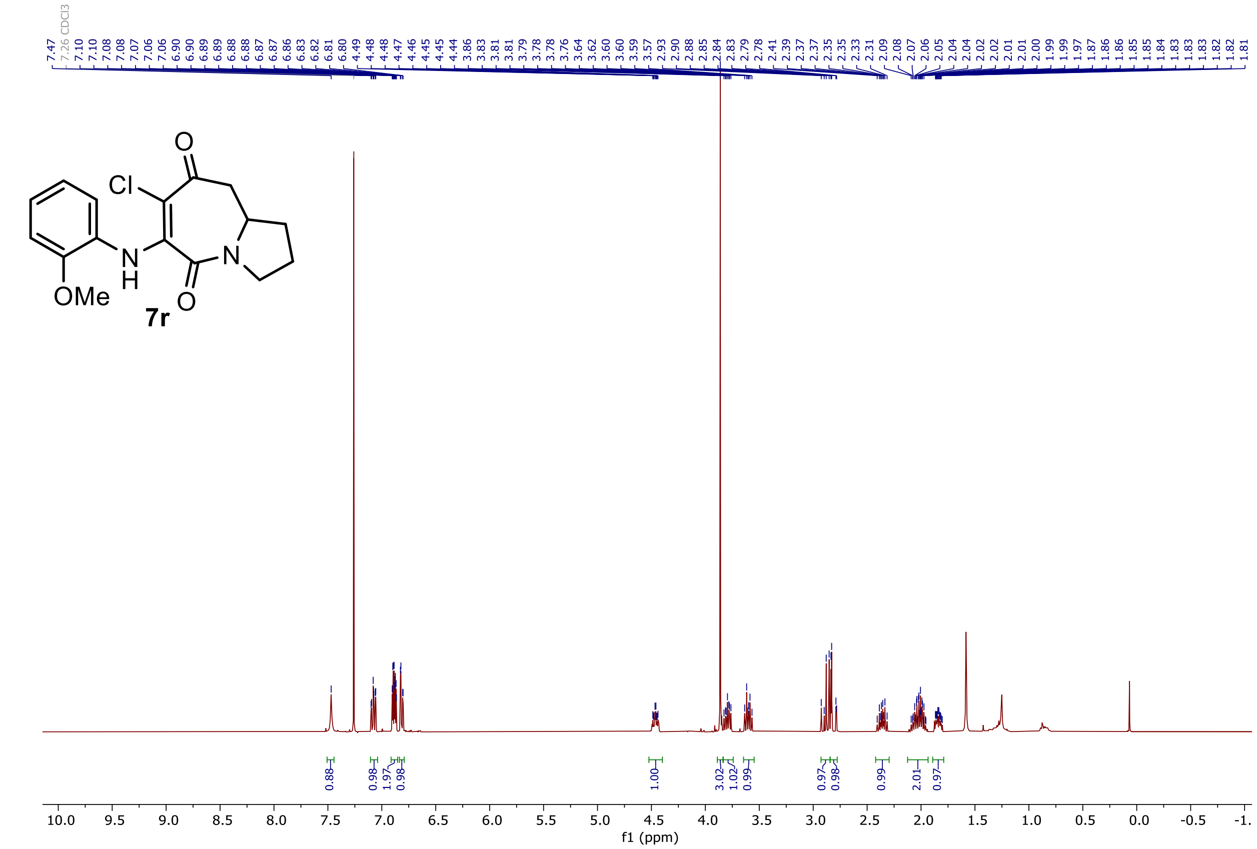

^{13}C NMR (126 MHz, $CDCl_3$) of **7r**

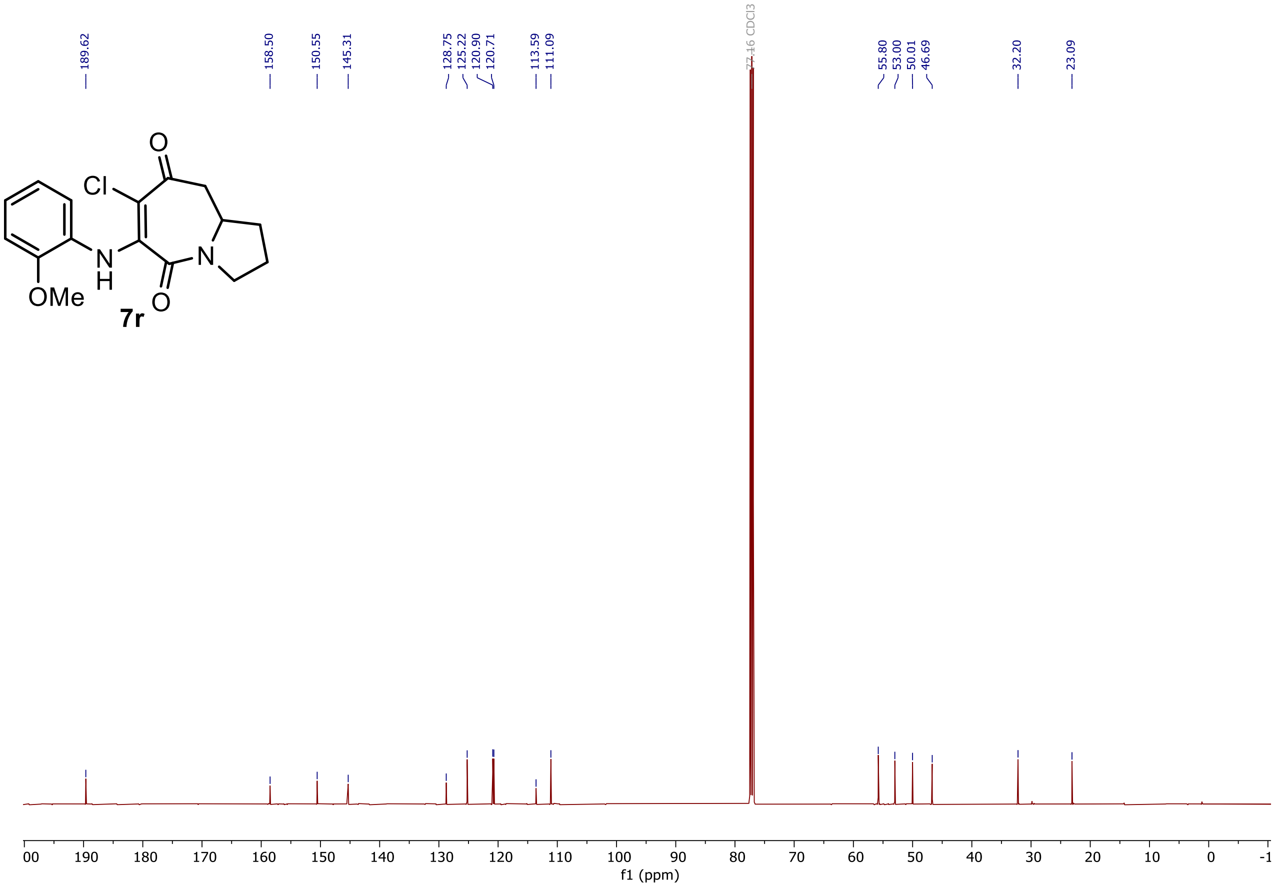

^{1}H NMR (400 MHz, DMSO-d_6) of **2a**

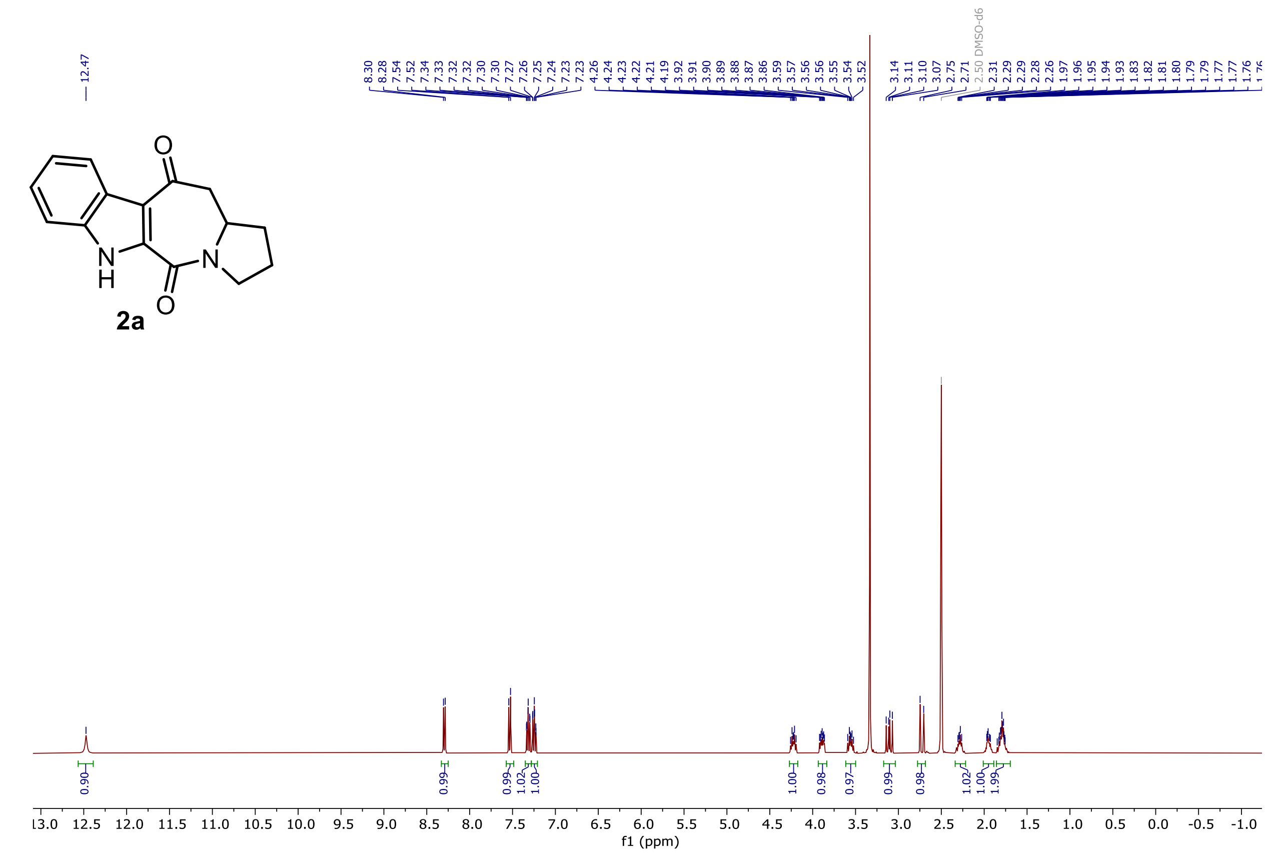

^{13}C NMR (151 MHz, DMSO-d_6) of **2a**

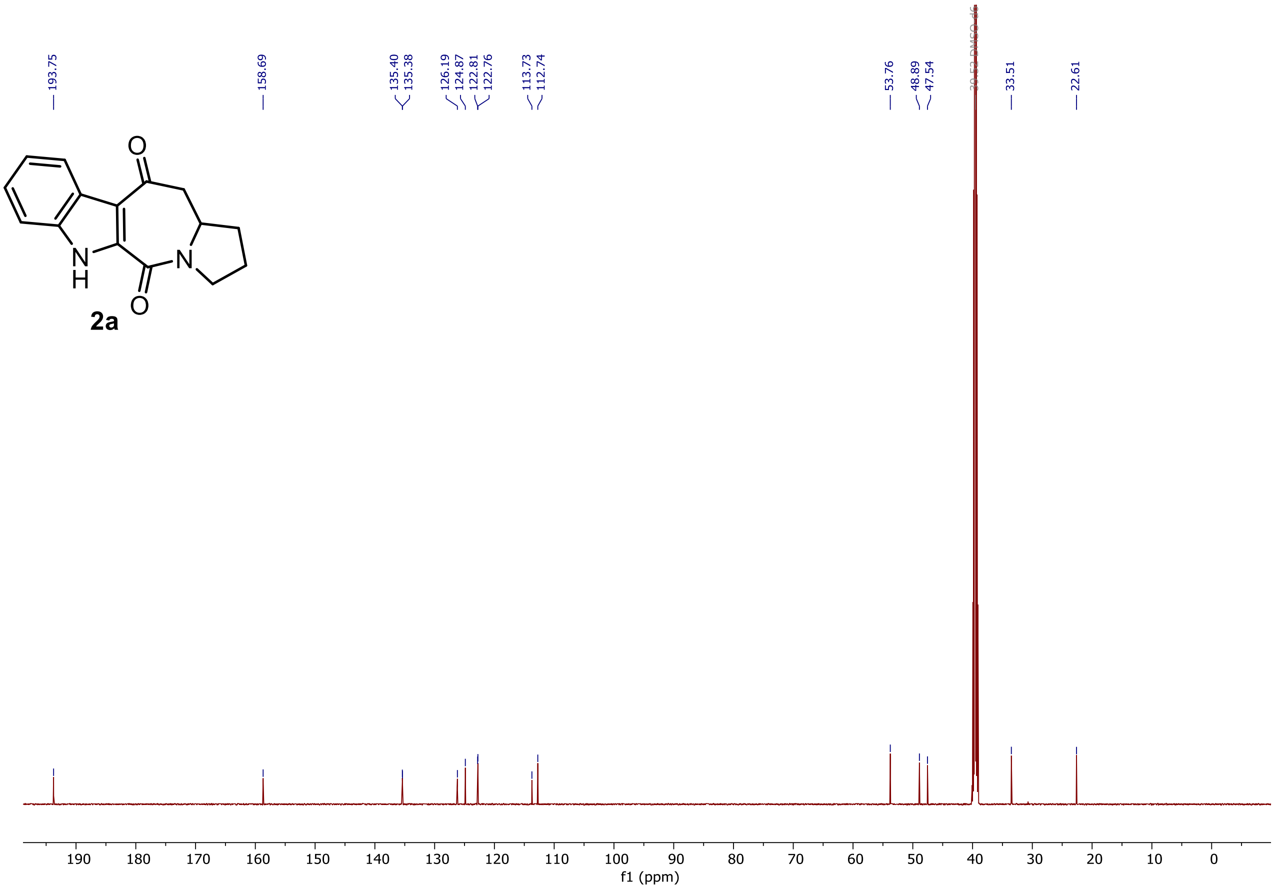

^{1}H NMR (500 MHz, DMSO-d_6) of **2b**

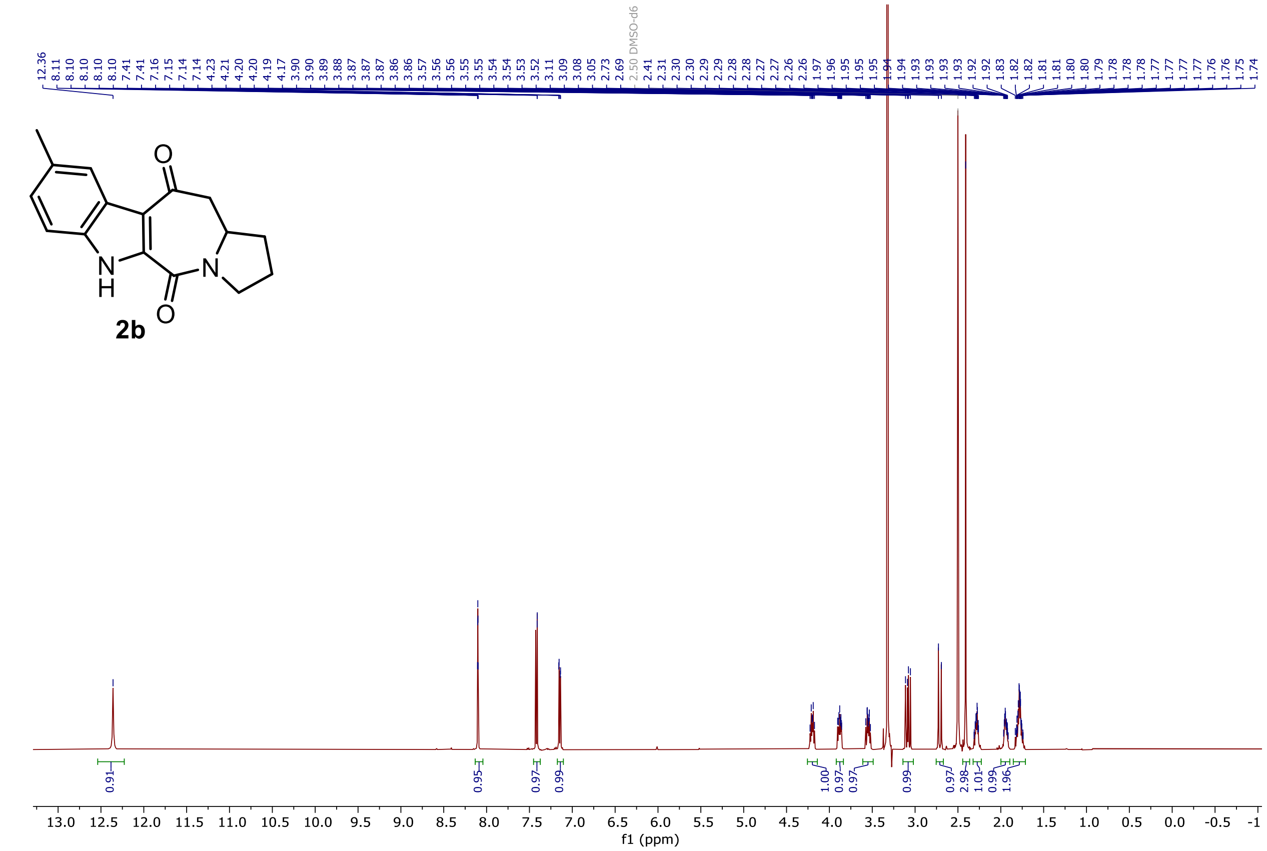

^{13}C NMR (126 MHz, DMSO-d_6) of **2b**

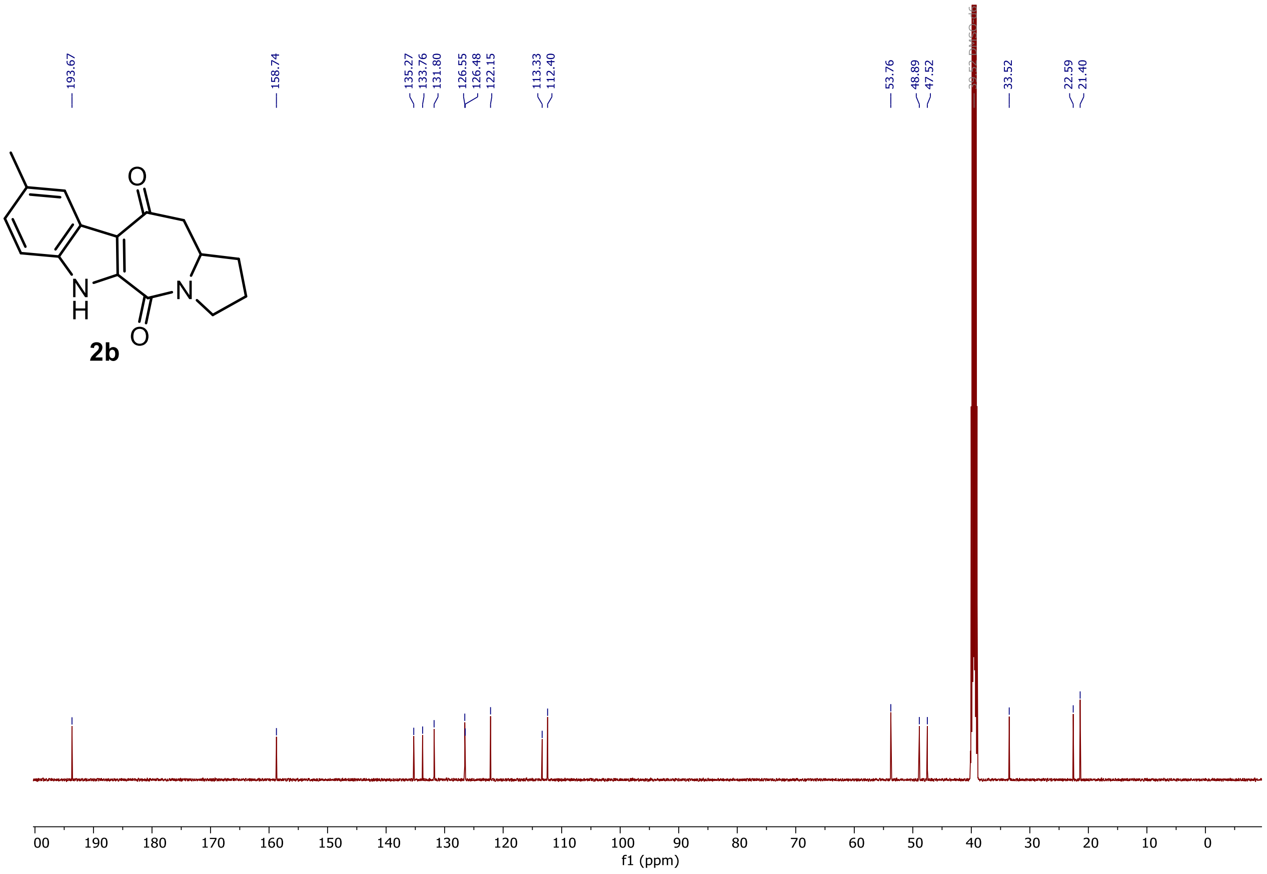

^{1}H NMR (500 MHz, DMSO-d_6) of **2c**

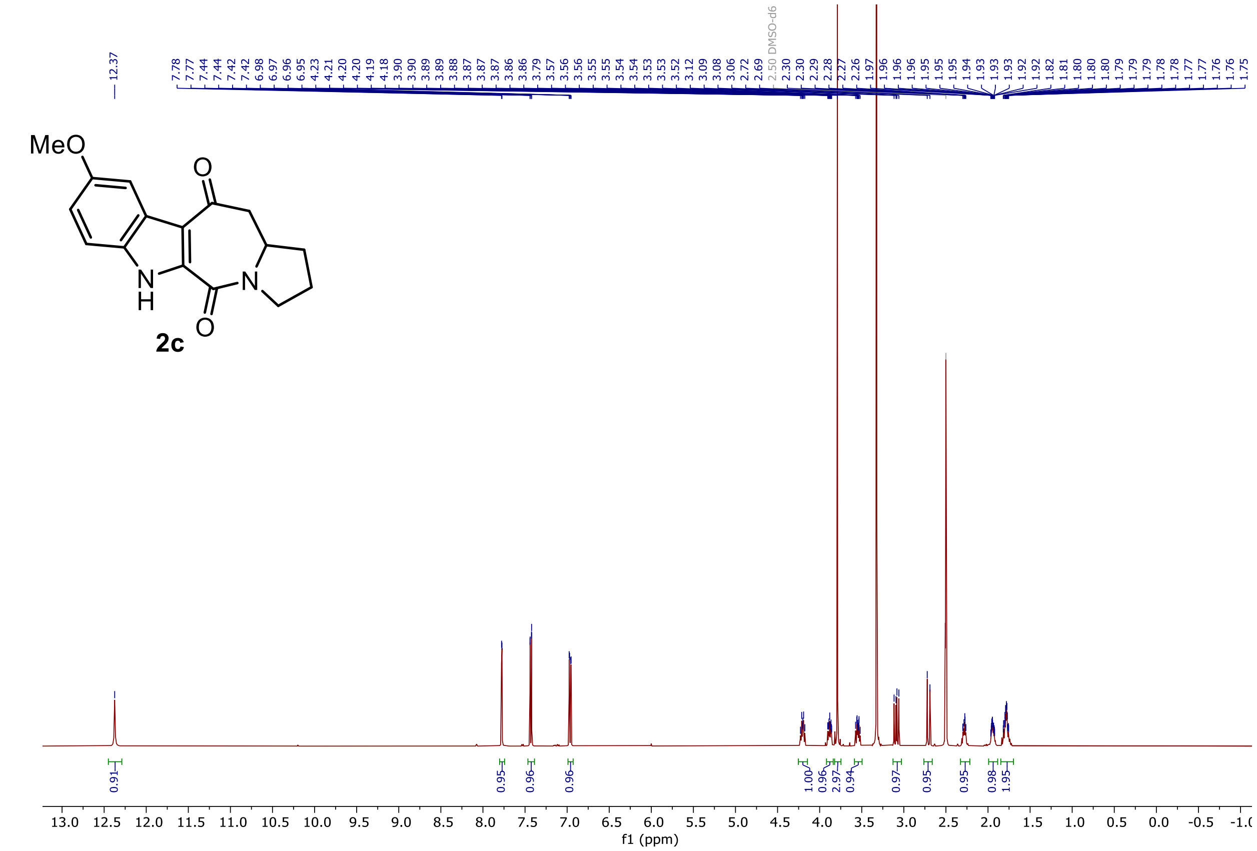

^{13}C NMR (126 MHz, DMSO-d_6) of **2c**

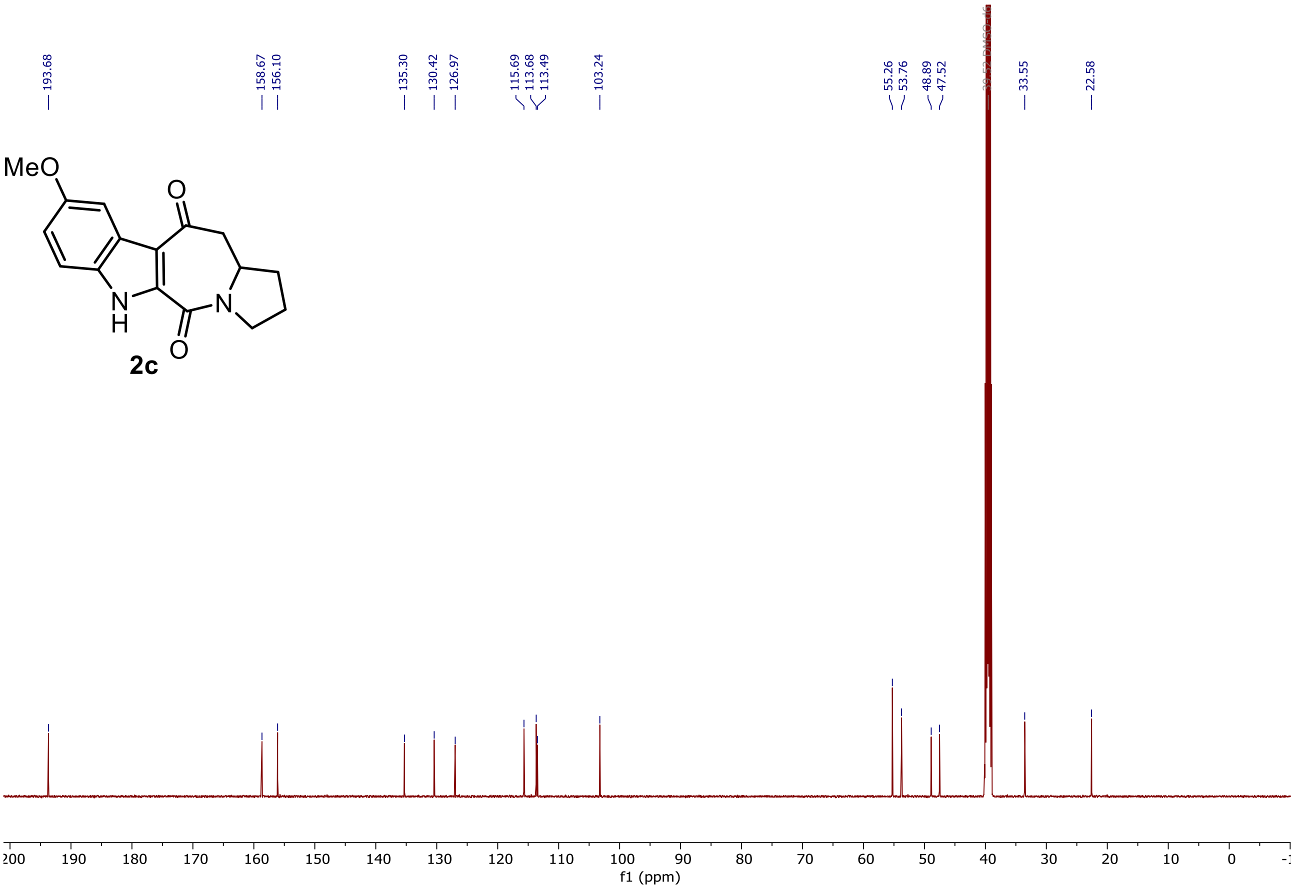

^{1}H NMR (400 MHz, DMSO-d_6) of **2d**

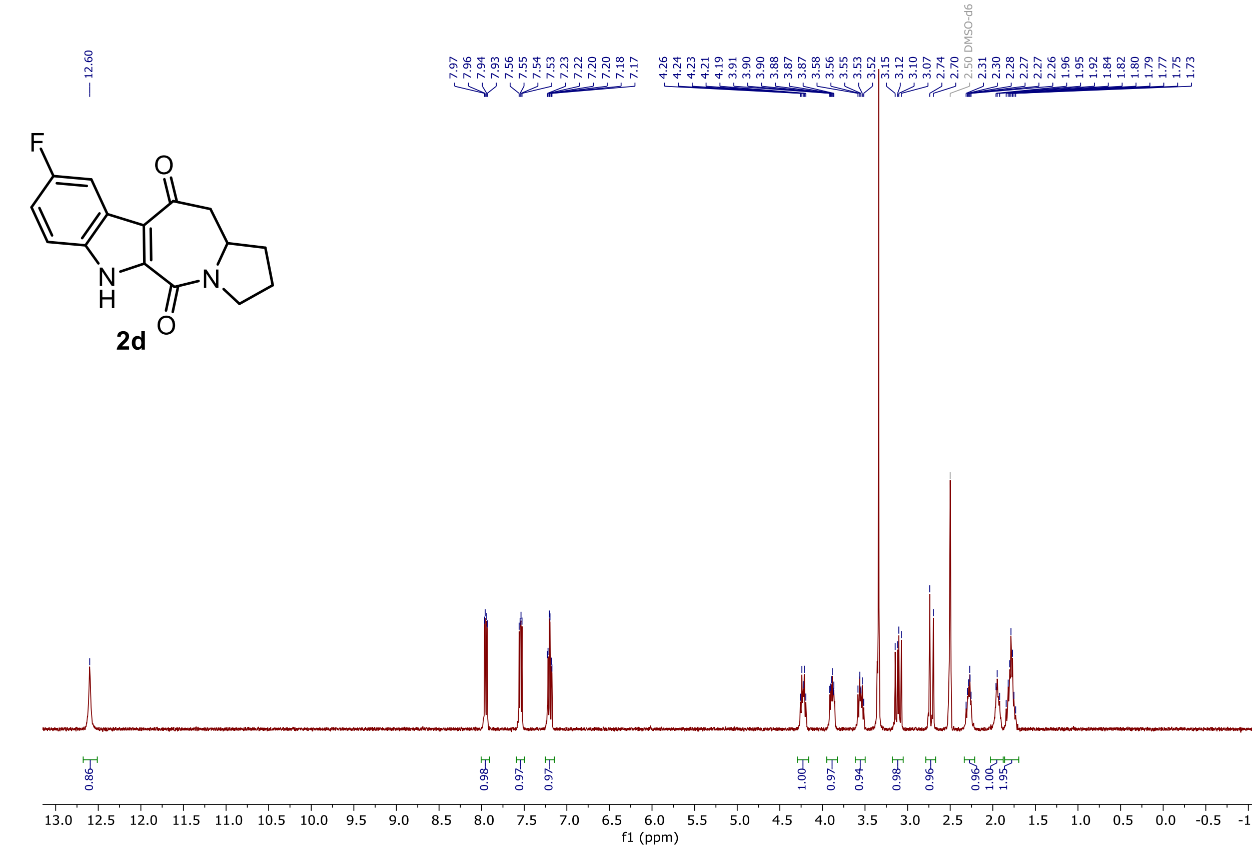

^{13}C NMR (126 MHz, DMSO-d_6) of **2d**

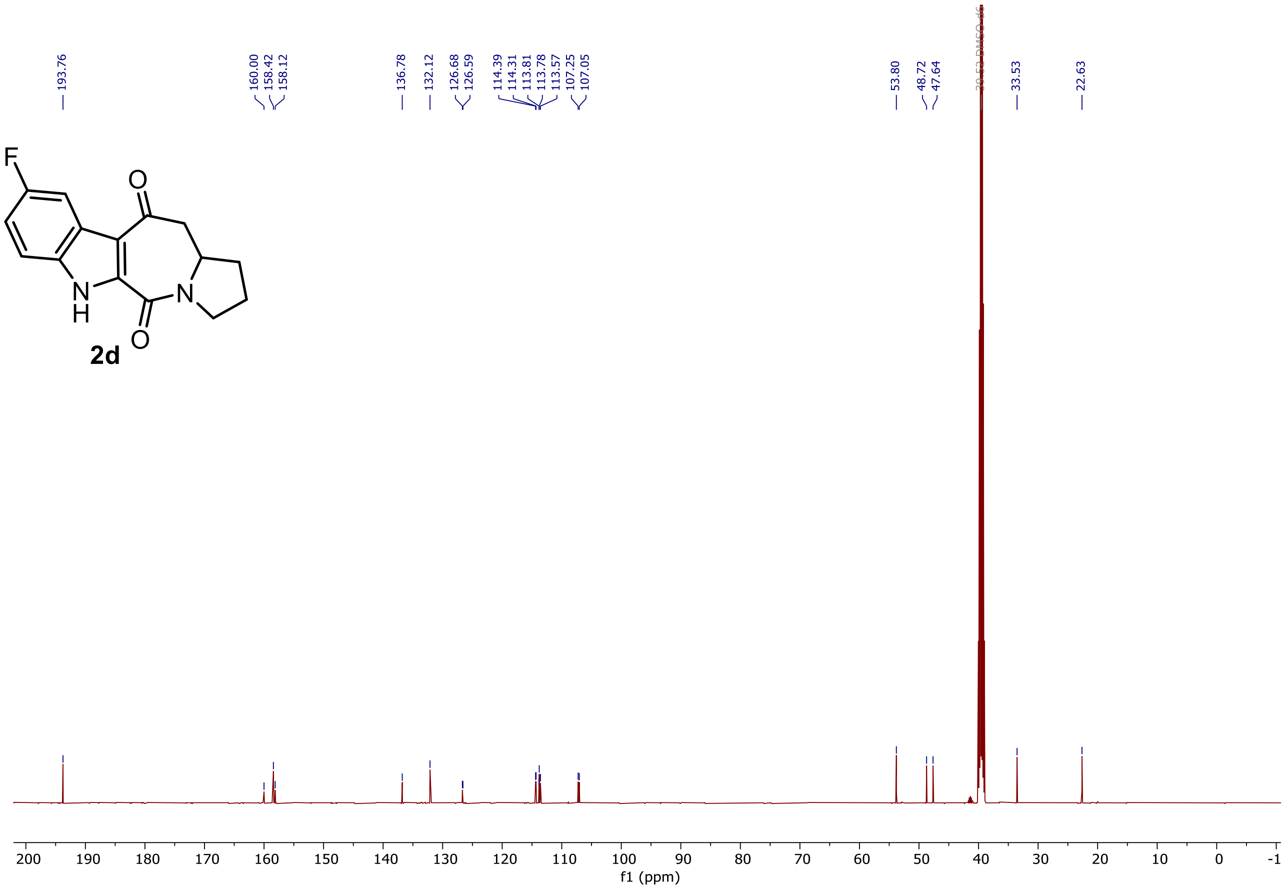

^{19}F NMR, ^{1}H decoupled (337 MHz, DMSO-d_6) of **2d**

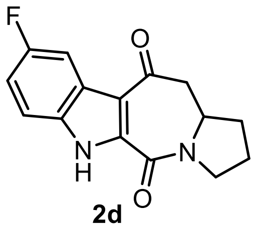

2d

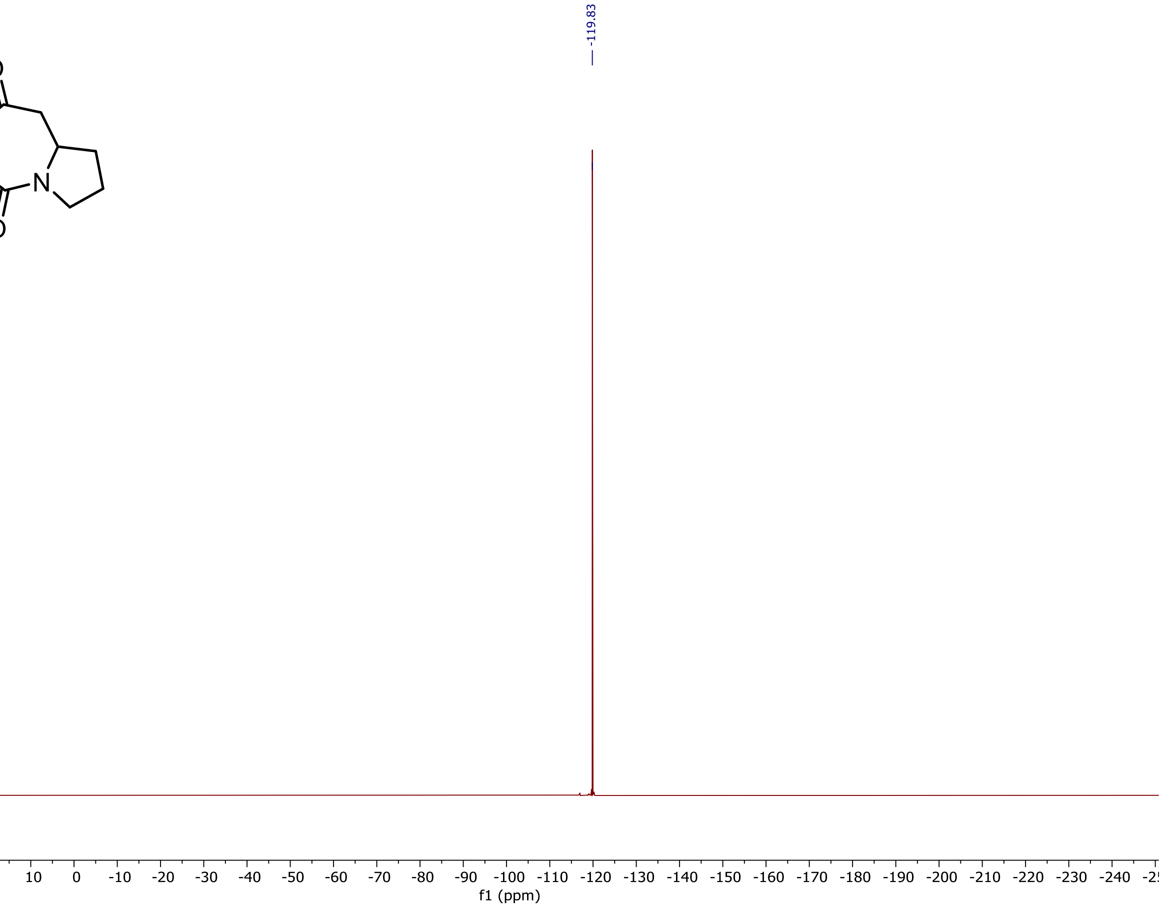

^{1}H NMR (400 MHz, DMSO-d_6) of **2e**

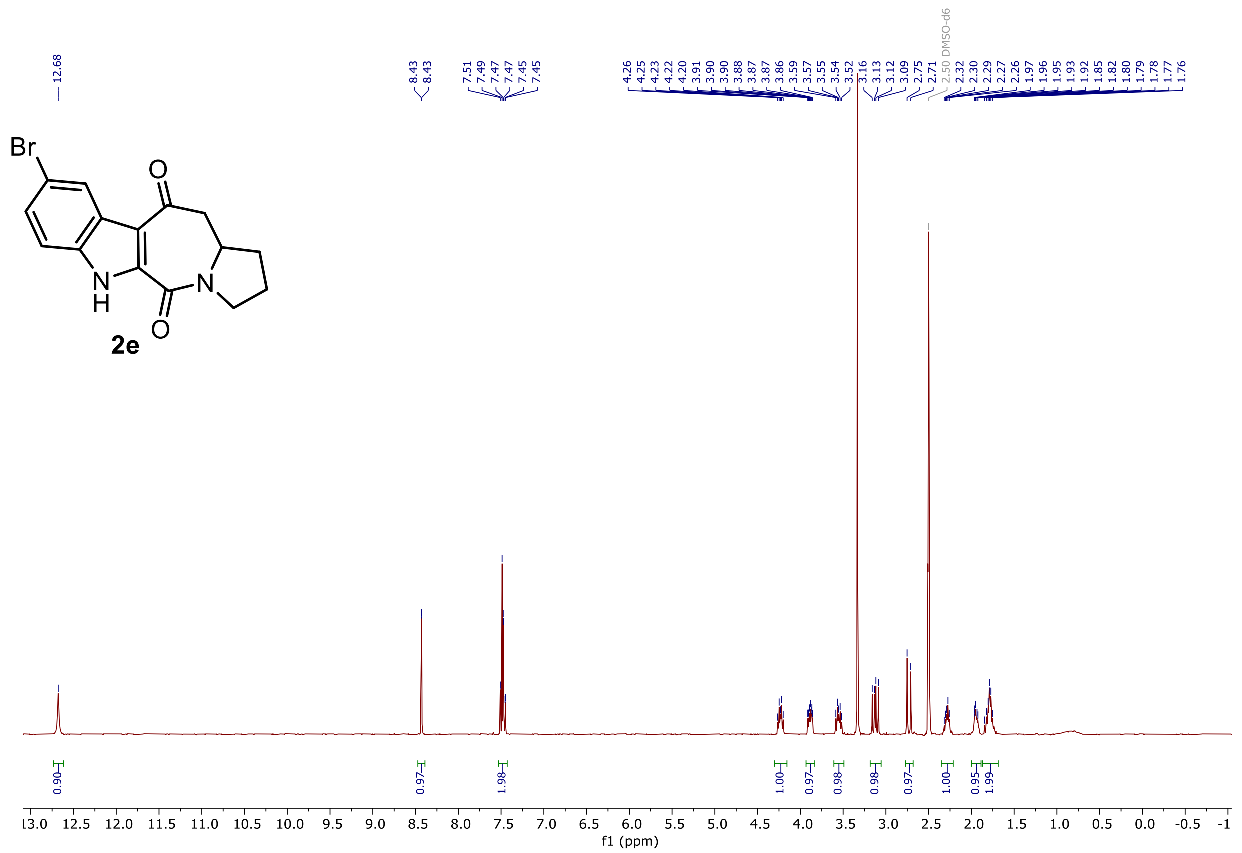

^{13}C NMR (126 MHz, DMSO-d_6) of **2e**

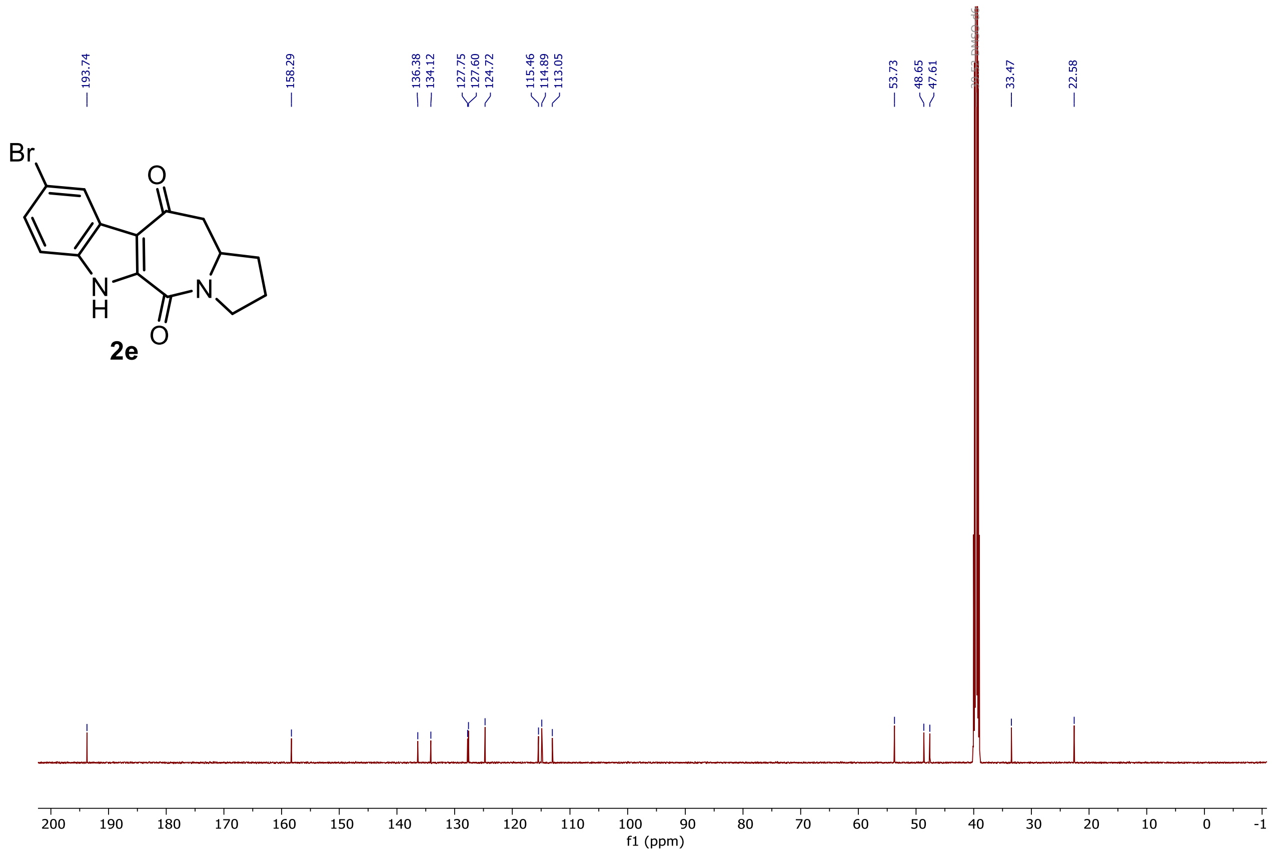

^{1}H NMR (400 MHz, DMSO-d_6) of **2f**

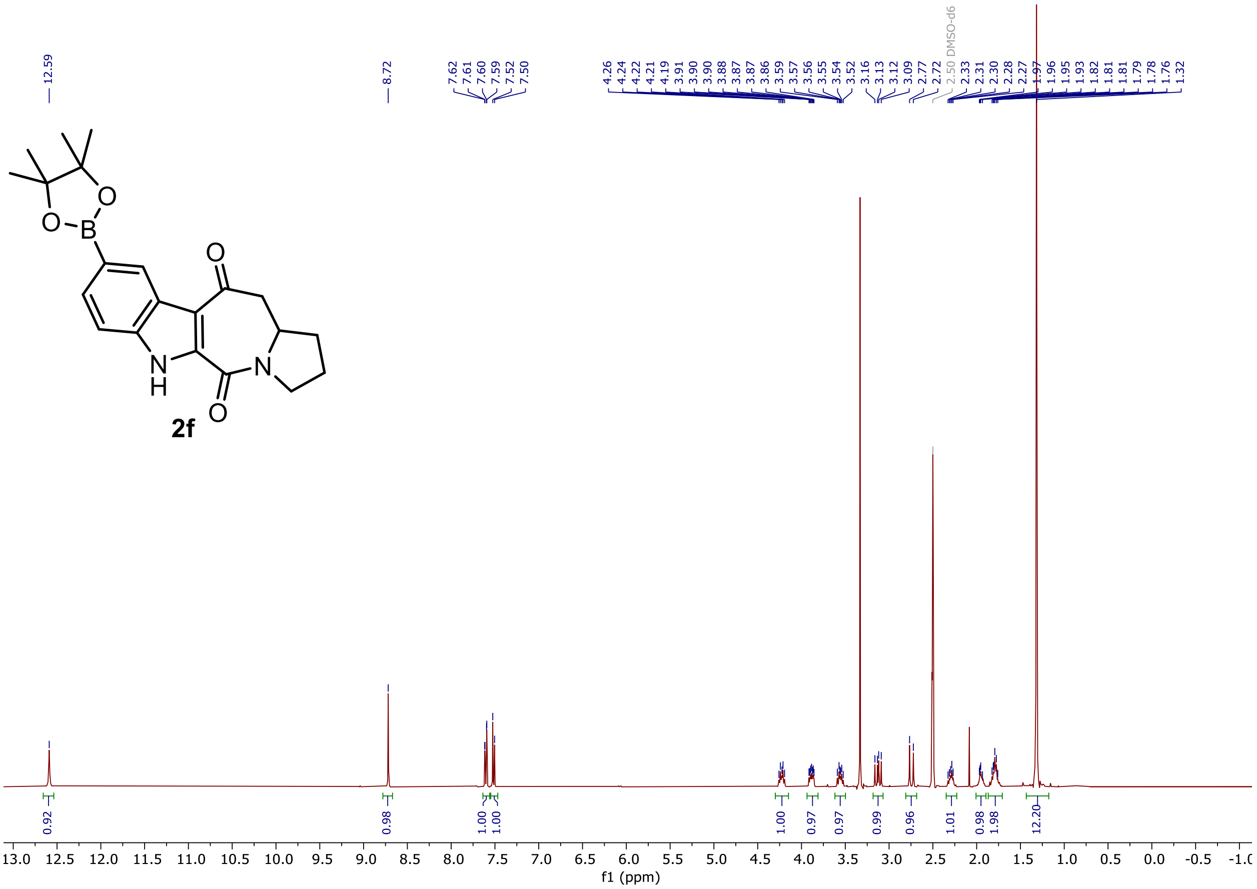

^{13}C NMR (126 MHz, DMSO-d_6) of **2f**

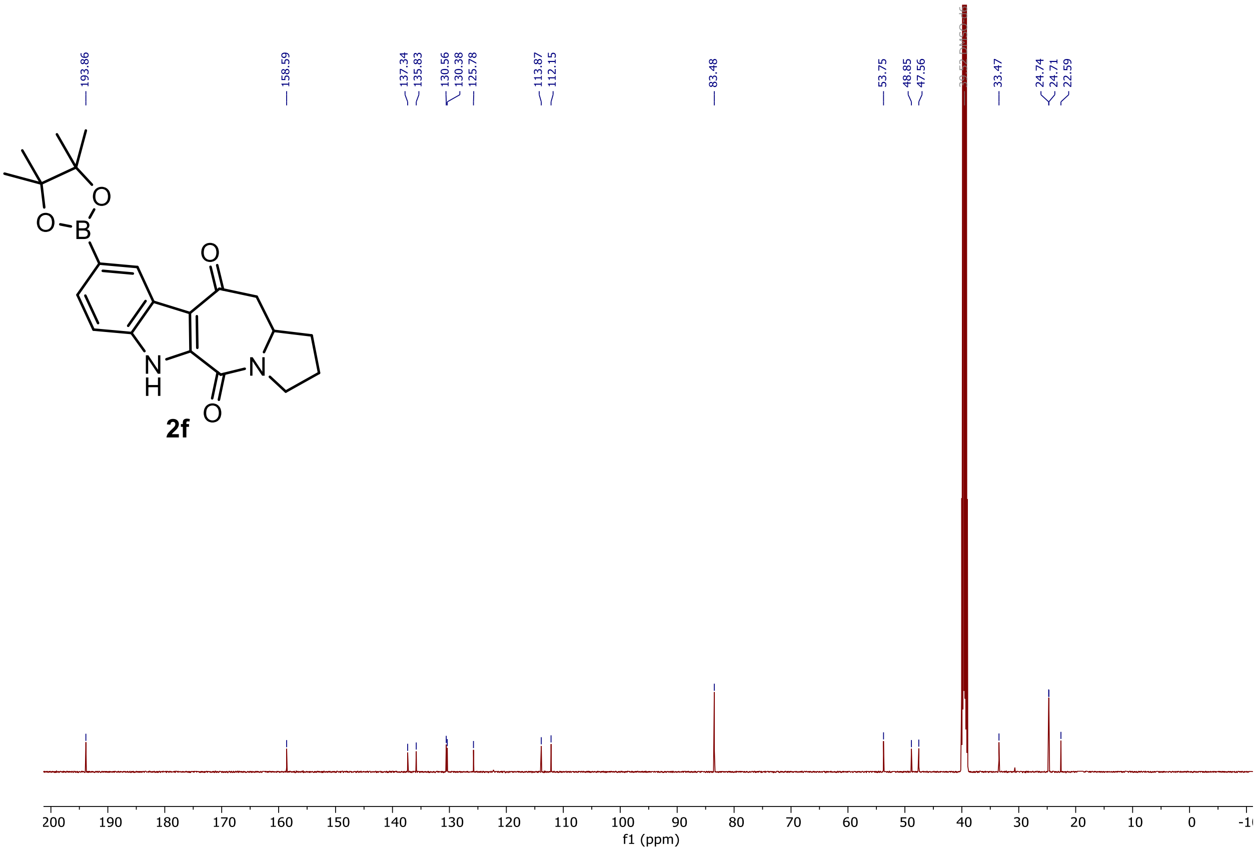

^{11}B NMR (128 MHz, DMSO-d_6) of **2f**

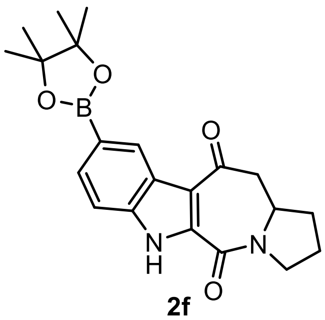

2f

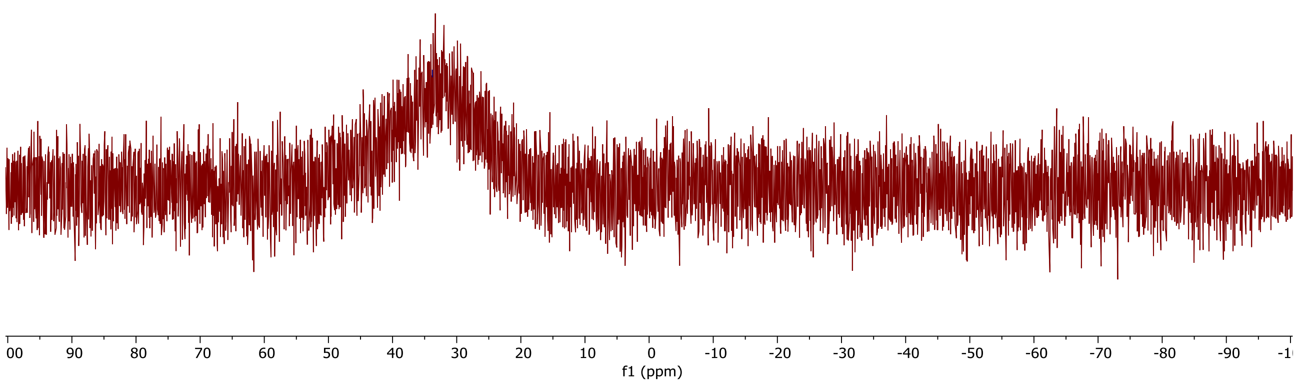

^{1}H NMR (400 MHz, DMSO-d_6) of **2g**

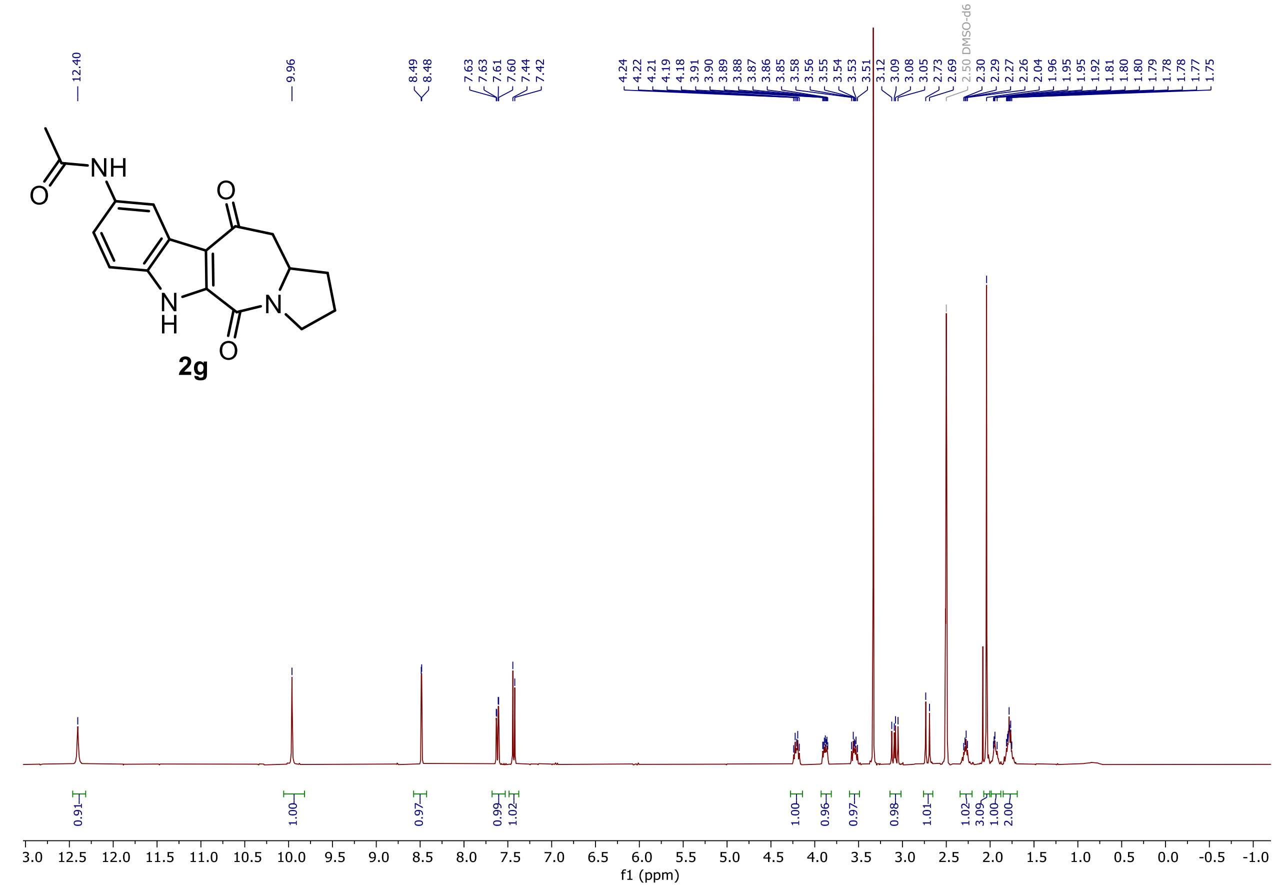

^{13}C NMR (126 MHz, DMSO-d_6) of **2g**

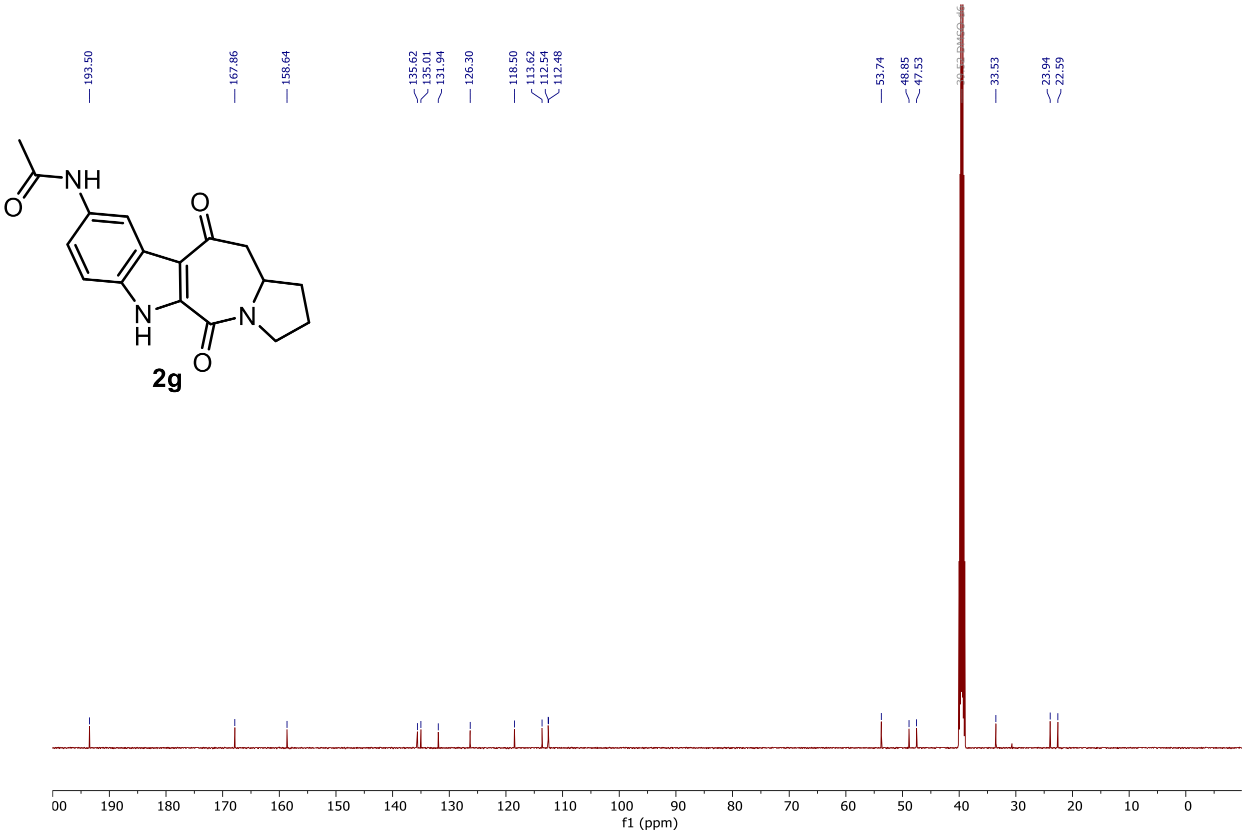

^{1}H NMR (400 MHz, DMSO-d_6) of **2h**

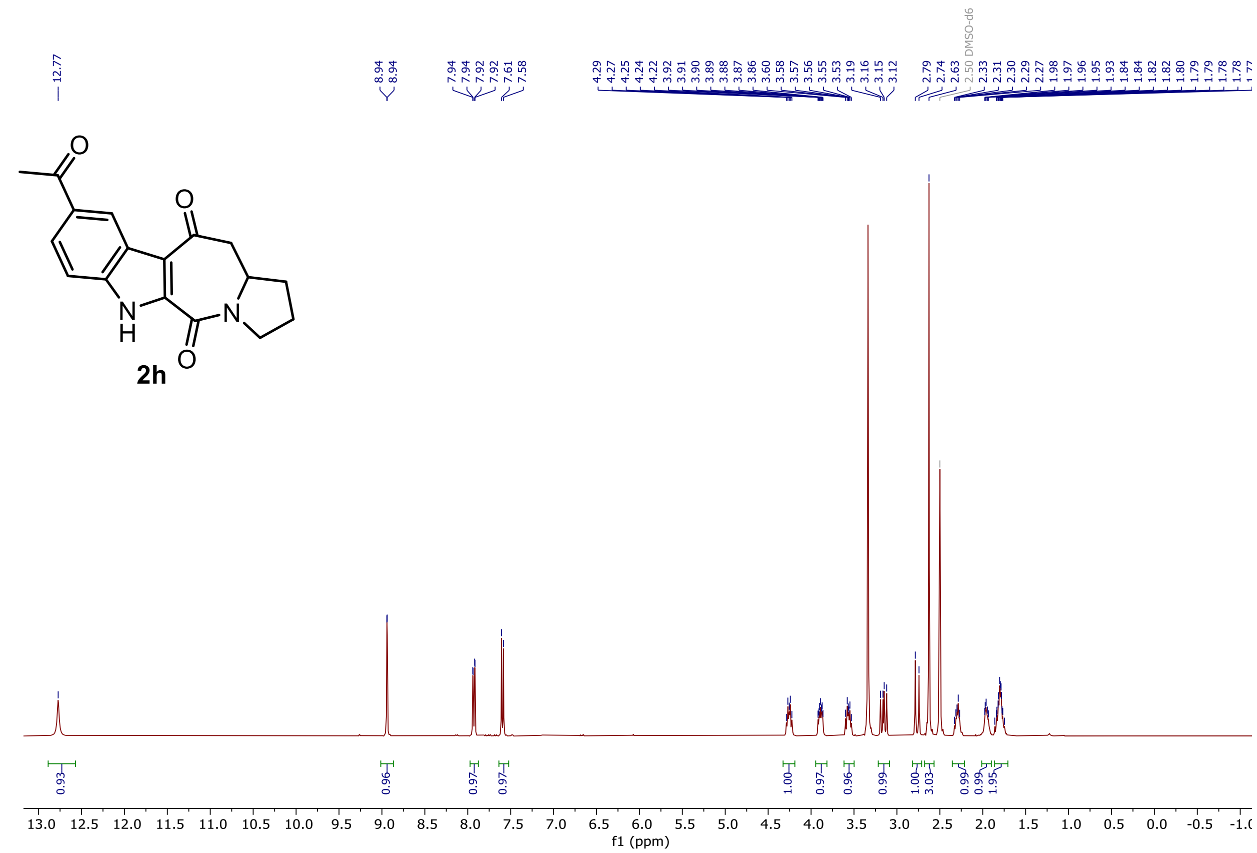

^{13}C NMR (126 MHz, DMSO-d_6) of **2h**

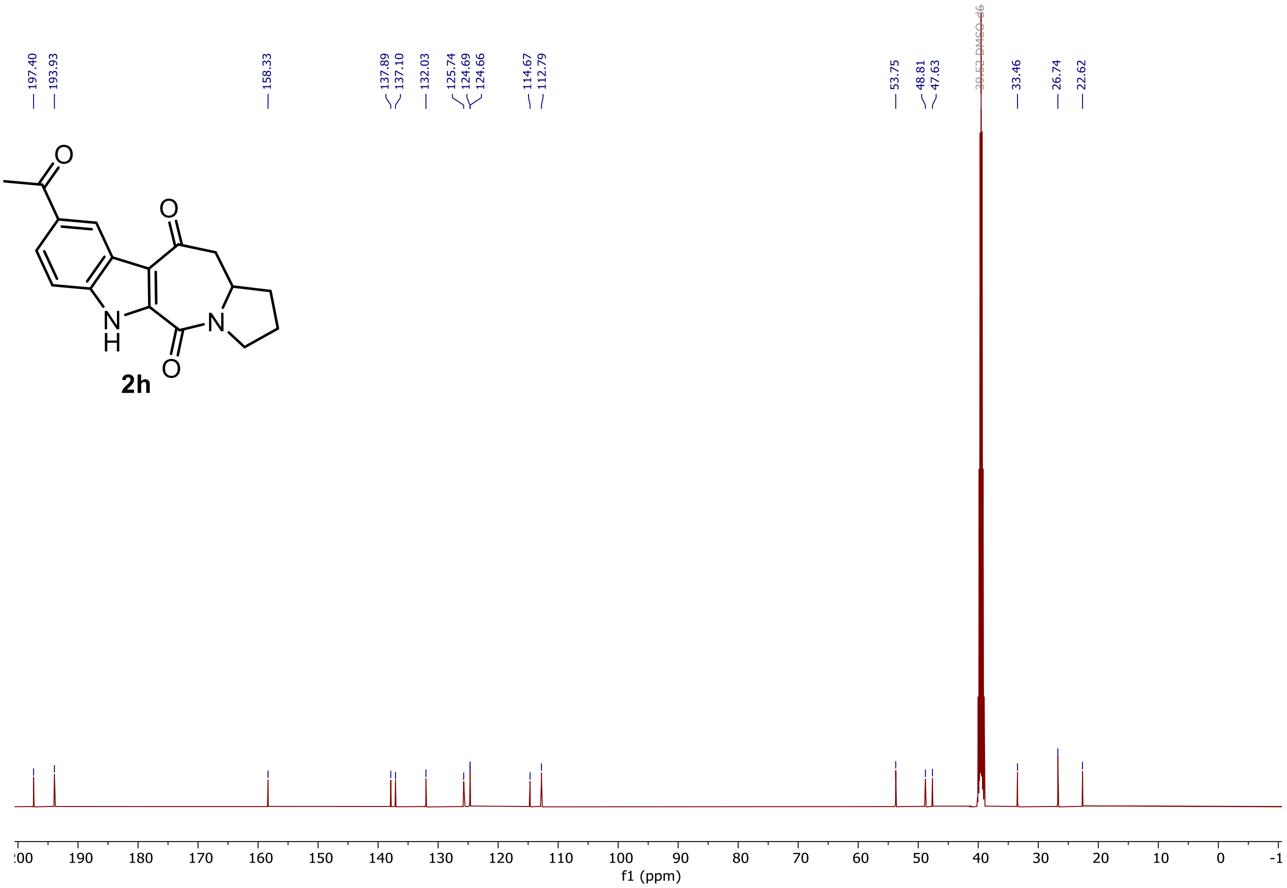

^{1}H NMR (500 MHz, DMSO-d_6) of **2i**

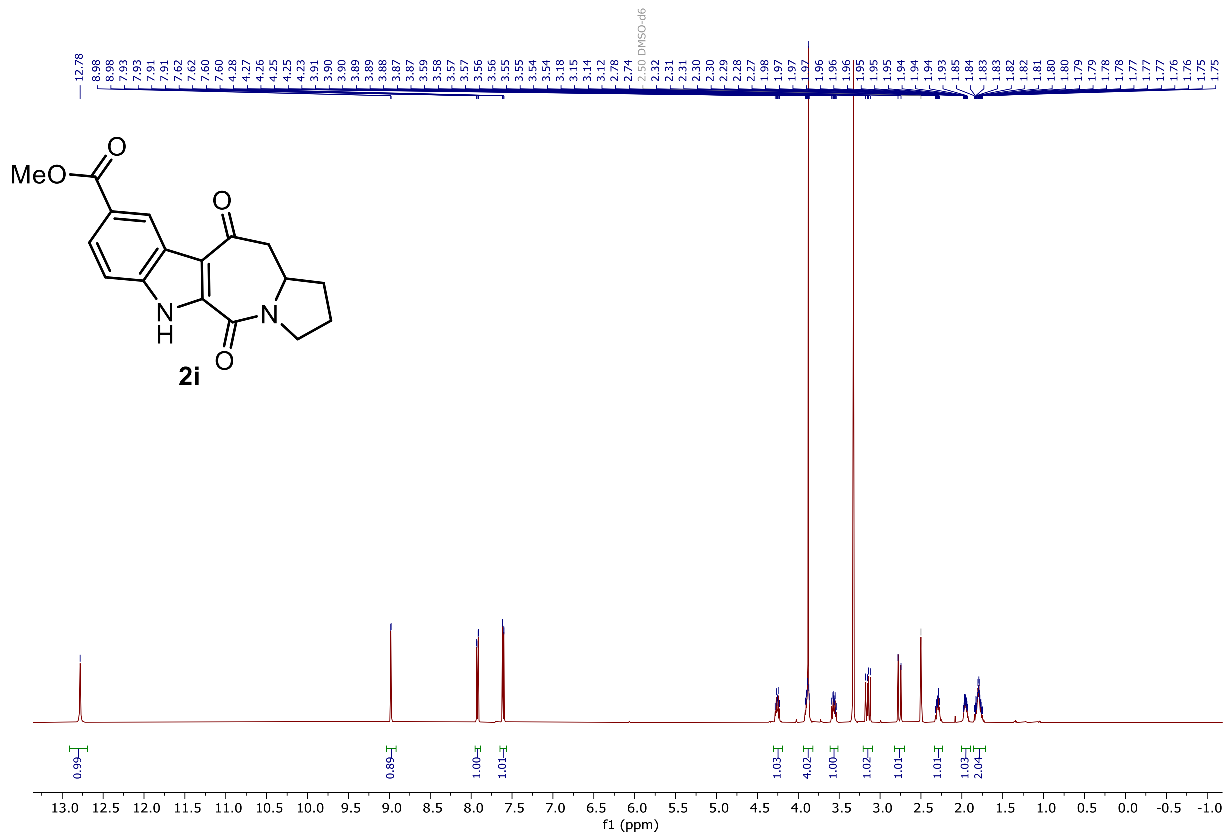

^{13}C NMR (126 MHz, DMSO-d_6) of **2i**

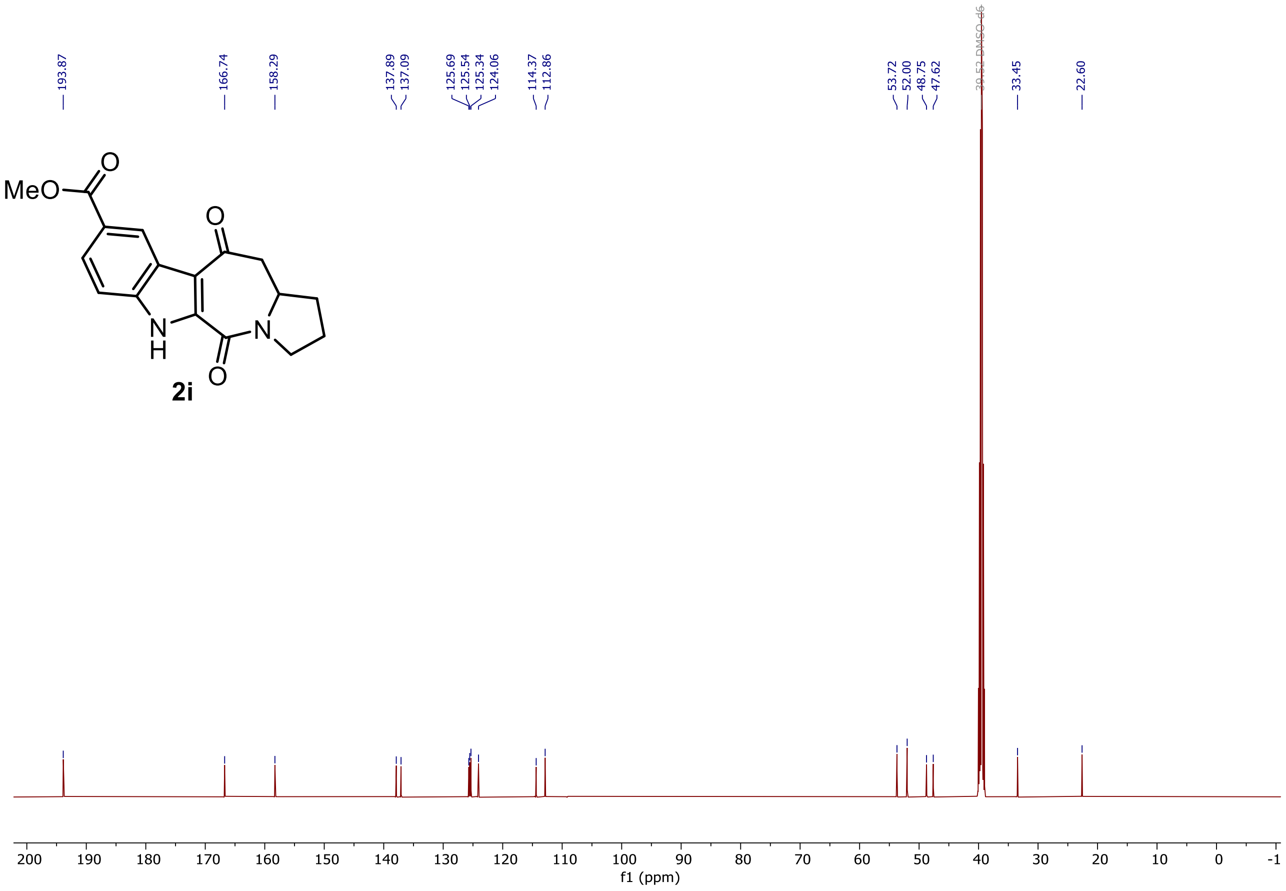

^1H NMR (400 MHz, DMSO-d_6) of **2j**

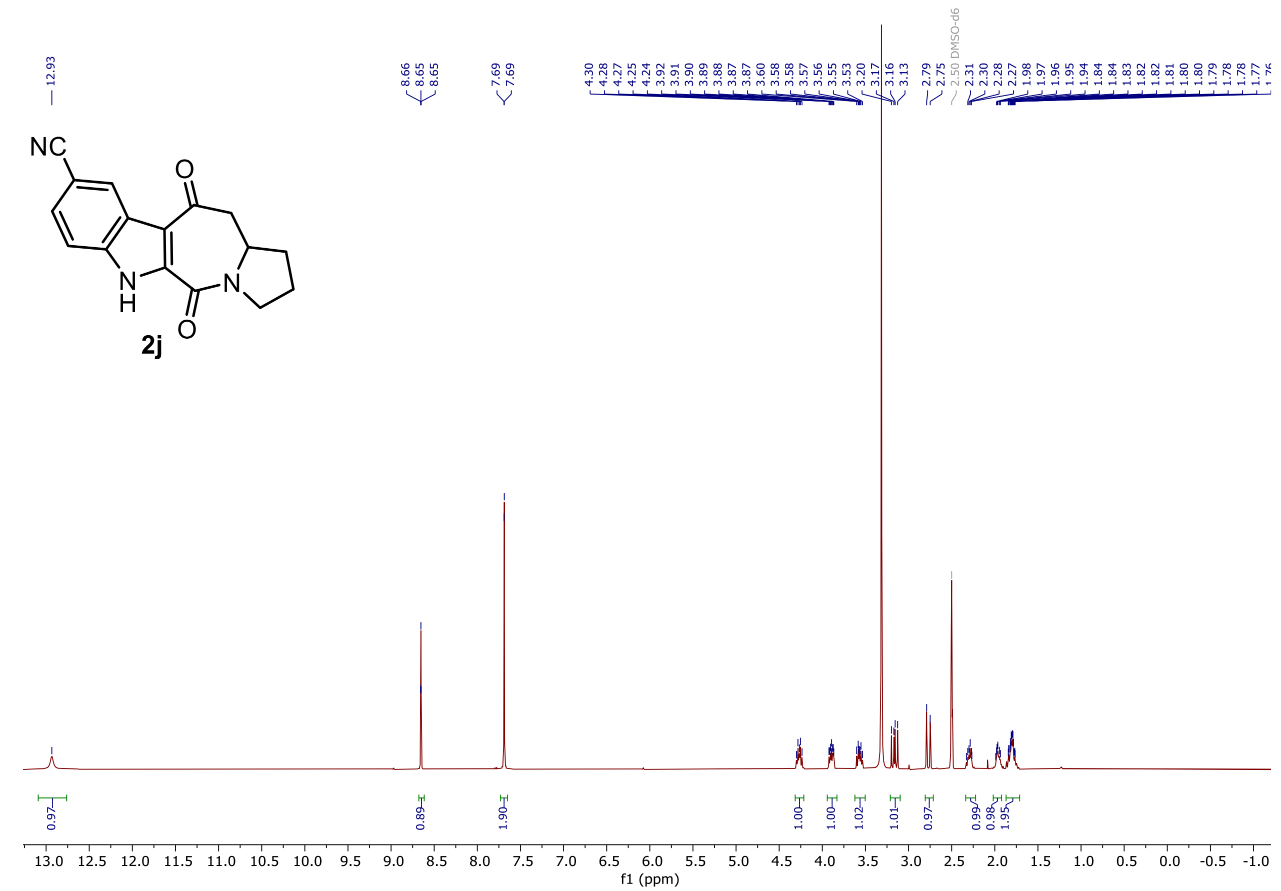

^{13}C NMR (126 MHz, DMSO-d_6) of **2j**

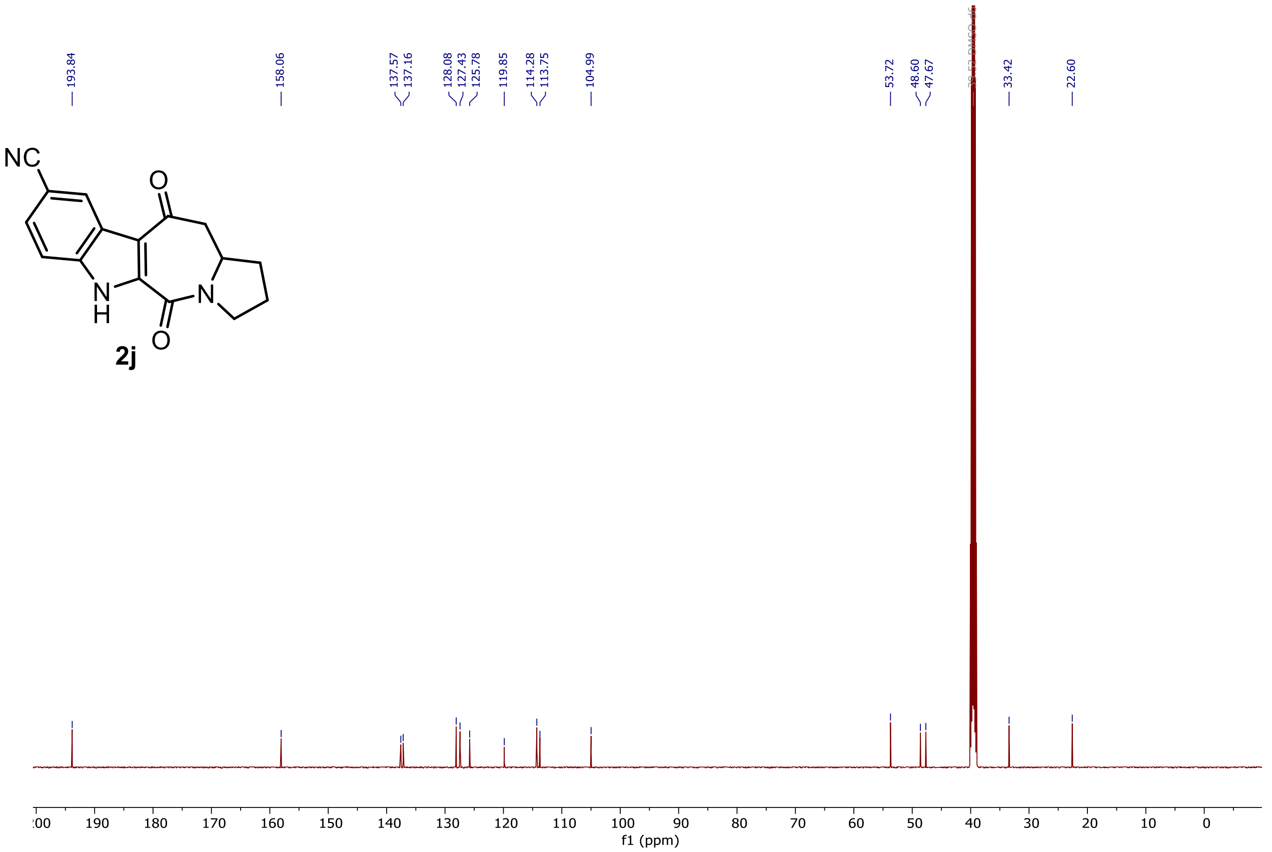

^{1}H NMR (500 MHz, DMSO-d_6) of **2k**

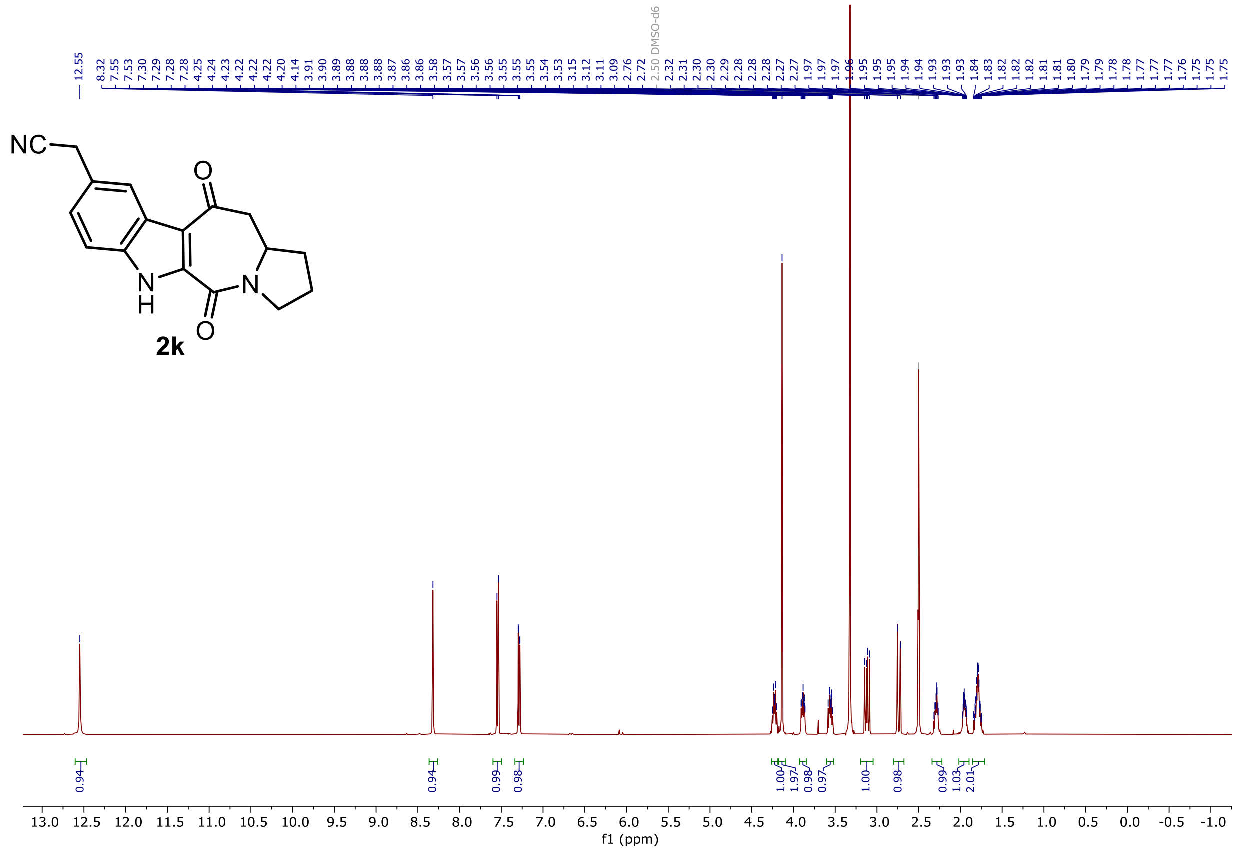

^{13}C NMR (126 MHz, DMSO-d_6) of **2k**

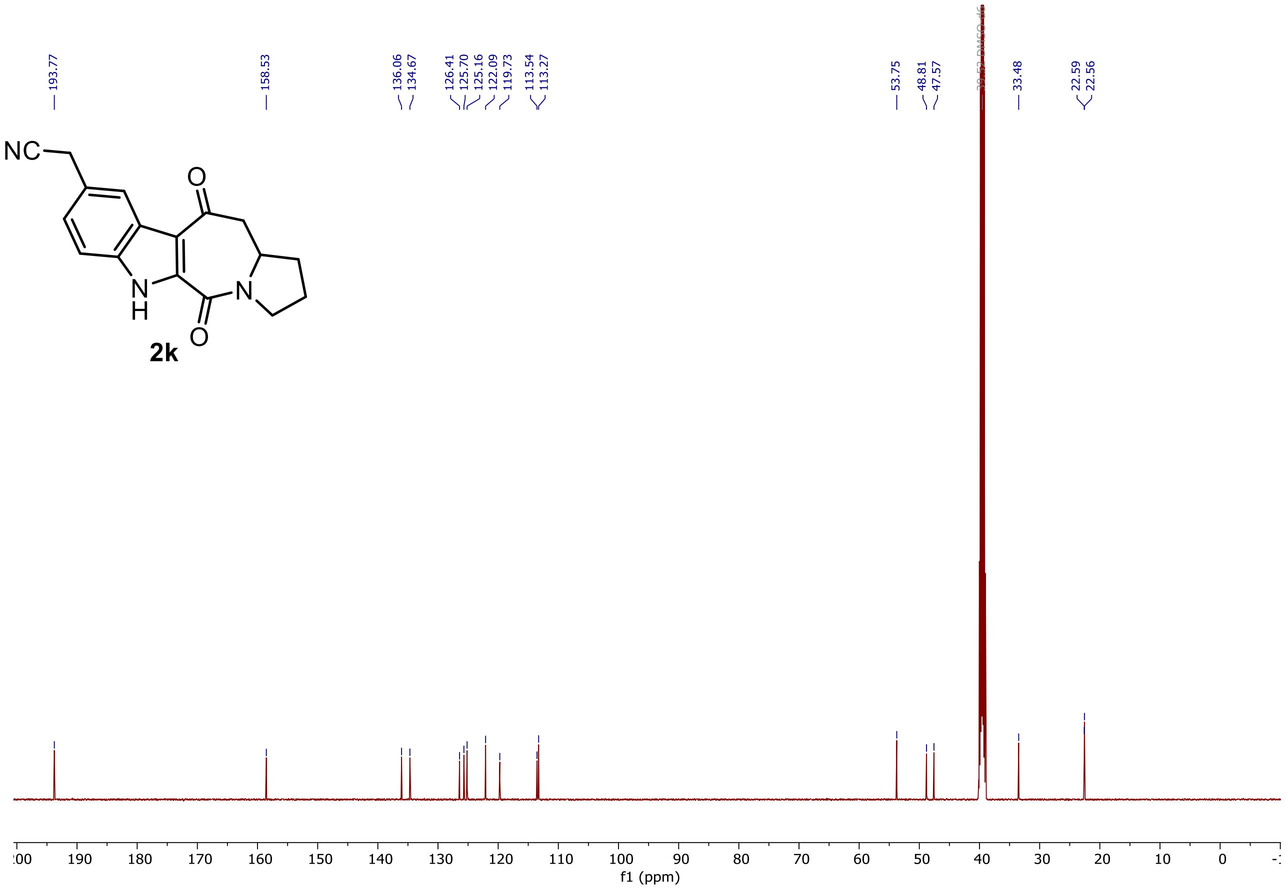

^{1}H NMR (400 MHz, $CDCl_3$) of **7l’**

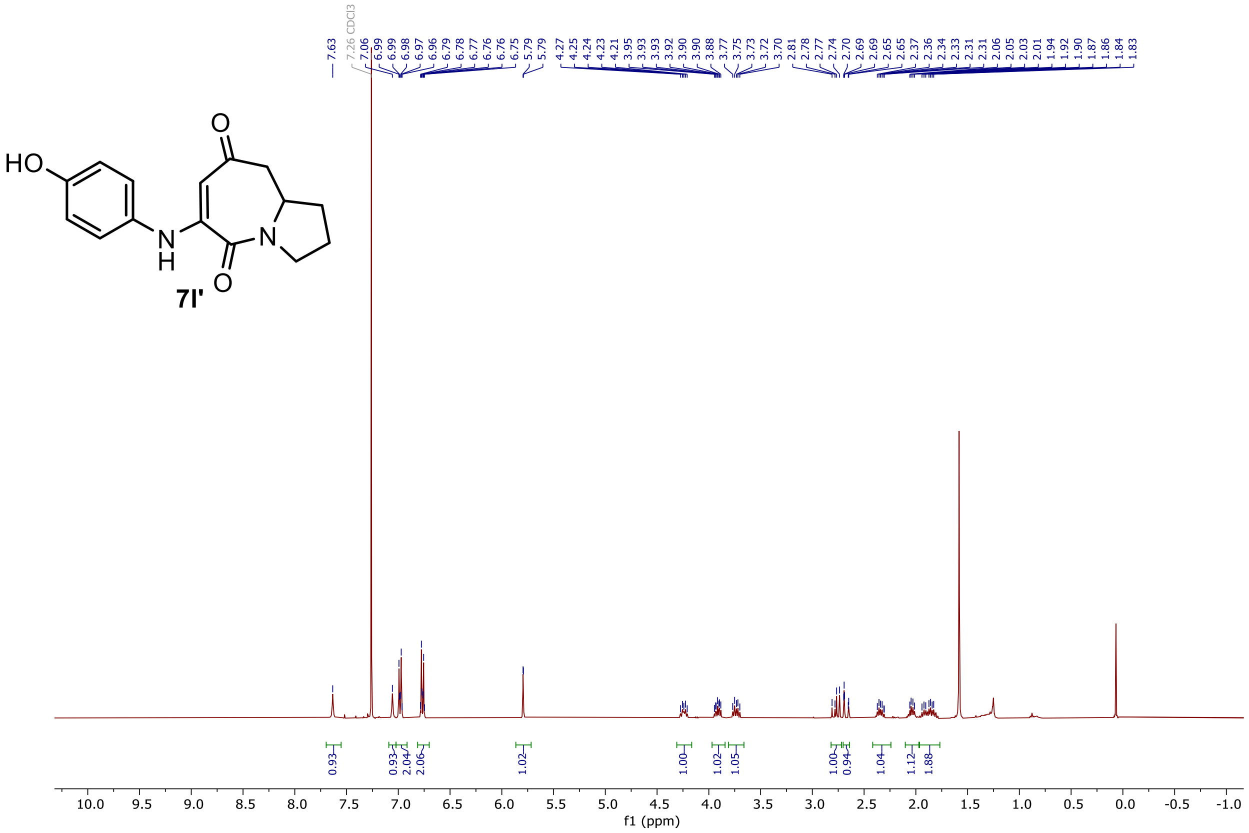

^{13}C NMR (151 MHz, $CDCl_3$) of **7l’**

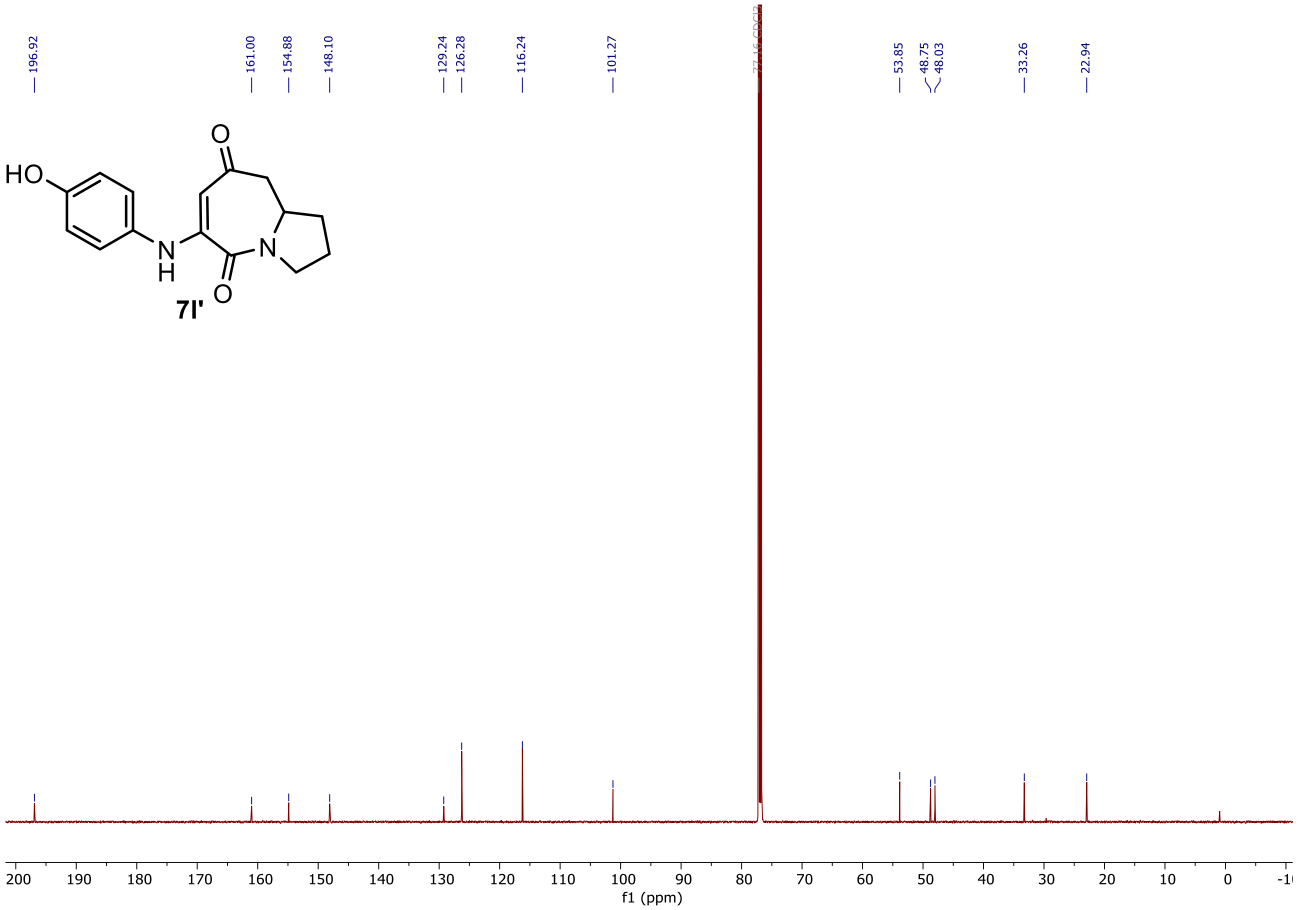

^{1}H NMR (400 MHz, DMSO-d_6) of **2m**

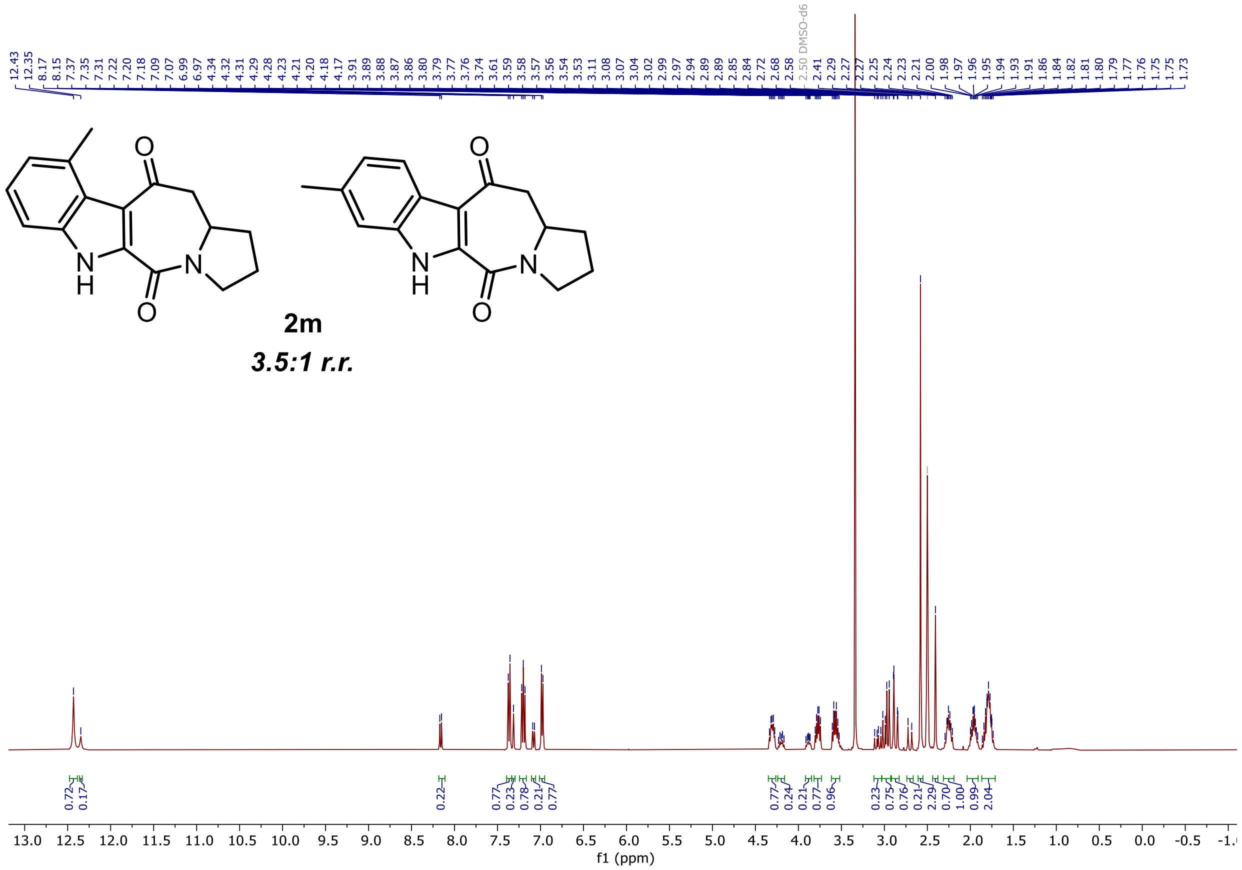

^{13}C NMR (151 MHz, DMSO-d_6) of **2m**

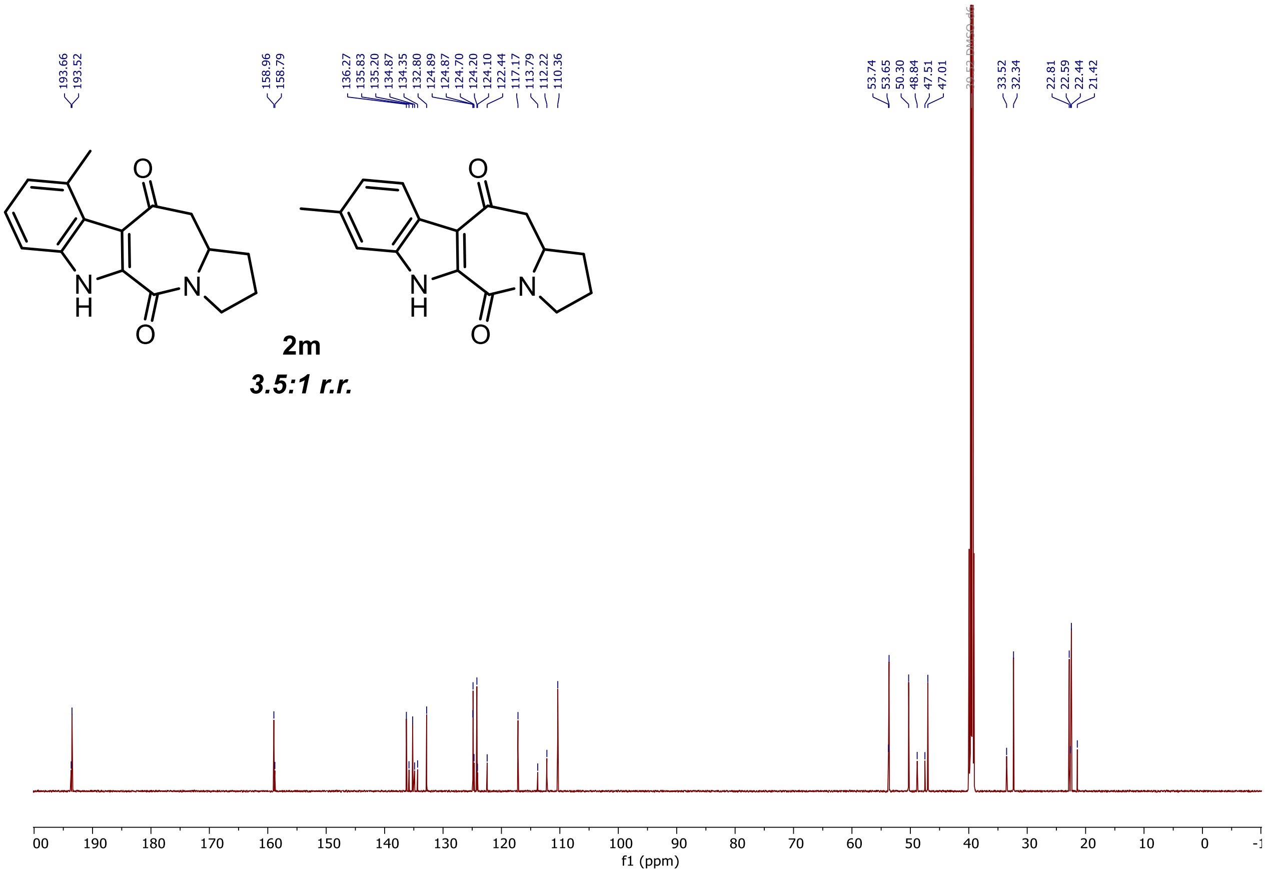

^{1}H NMR (400 MHz, DMSO-d_6) of **2n**

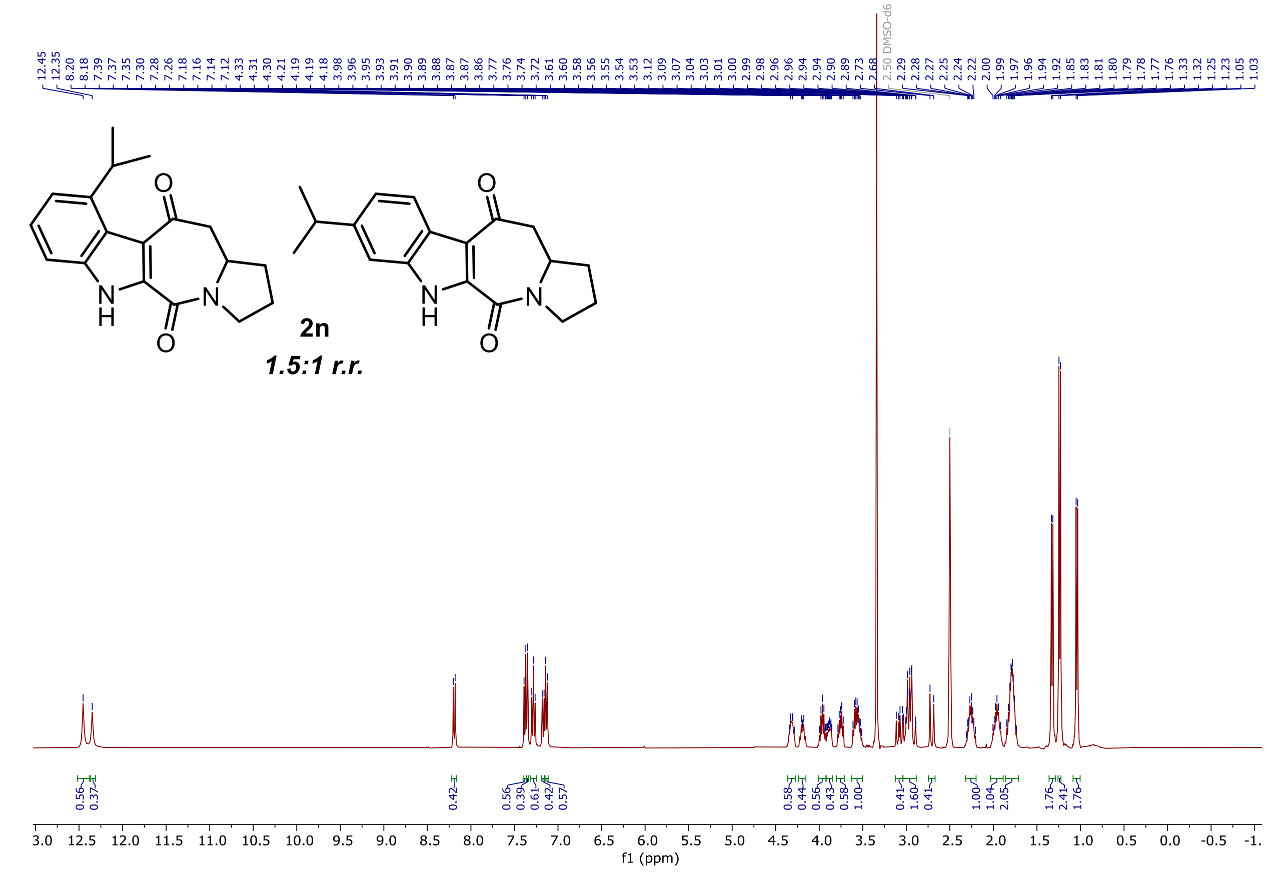

^{13}C NMR (151 MHz, DMSO-d_6) of **2n**

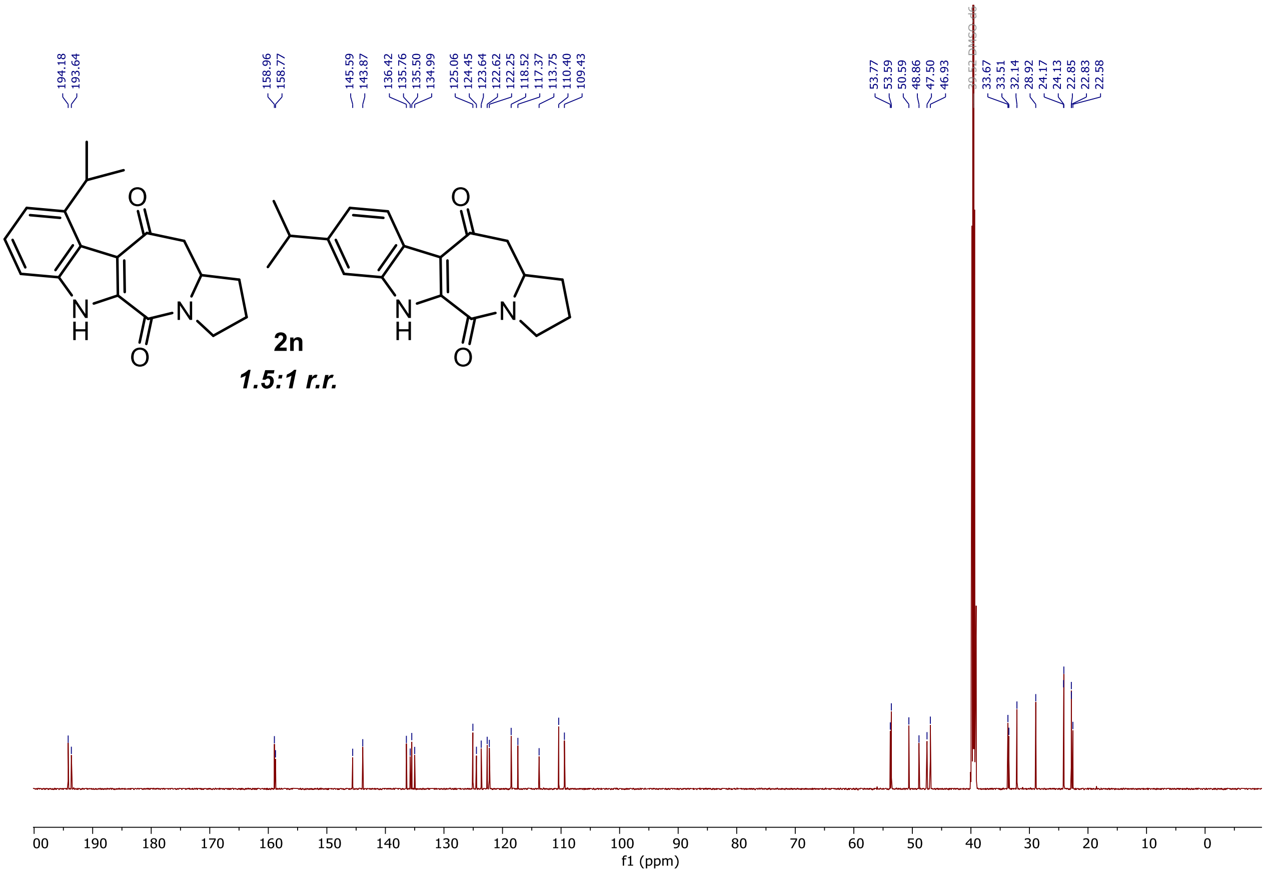

^{1}H NMR (400 MHz, DMSO-d_6) of **2o**

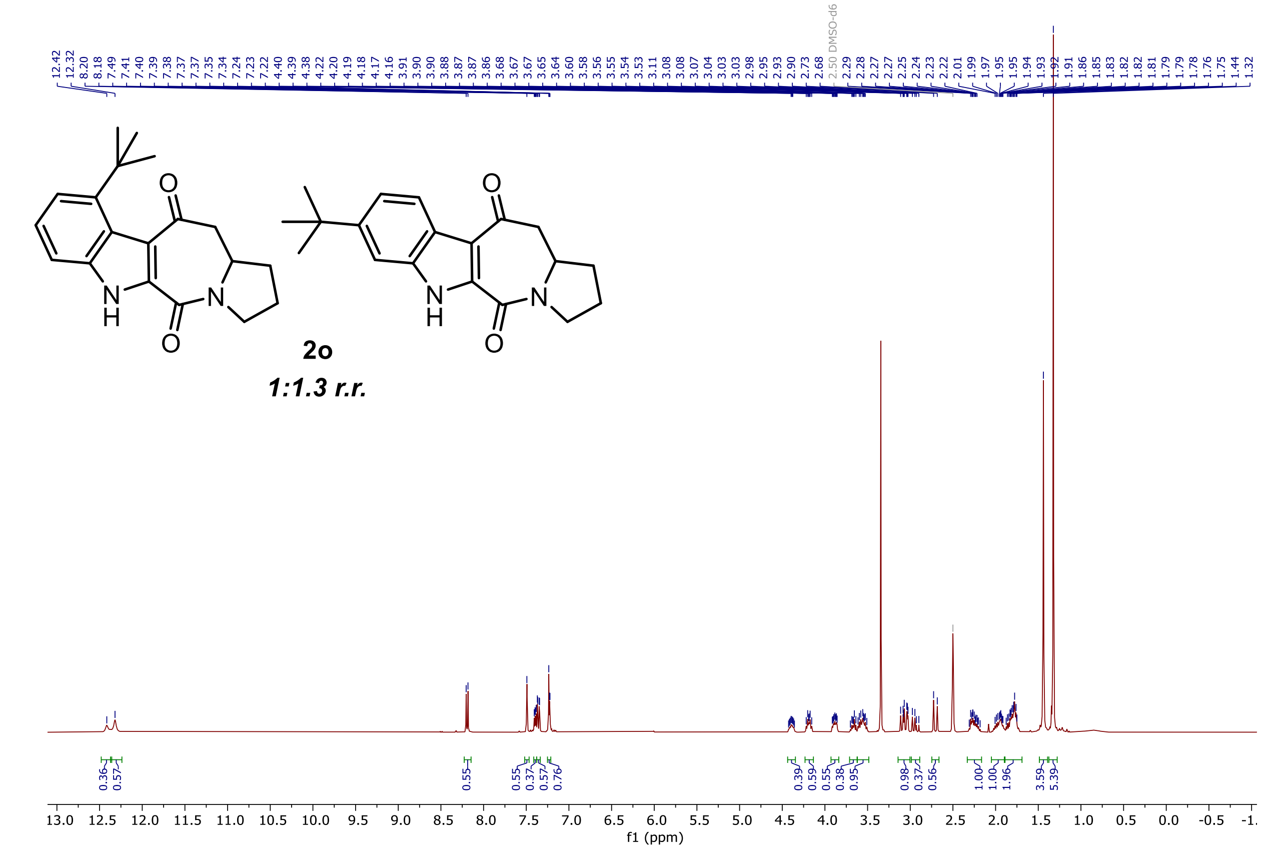

^{13}C NMR (151 MHz, DMSO-d_6) of **2o**

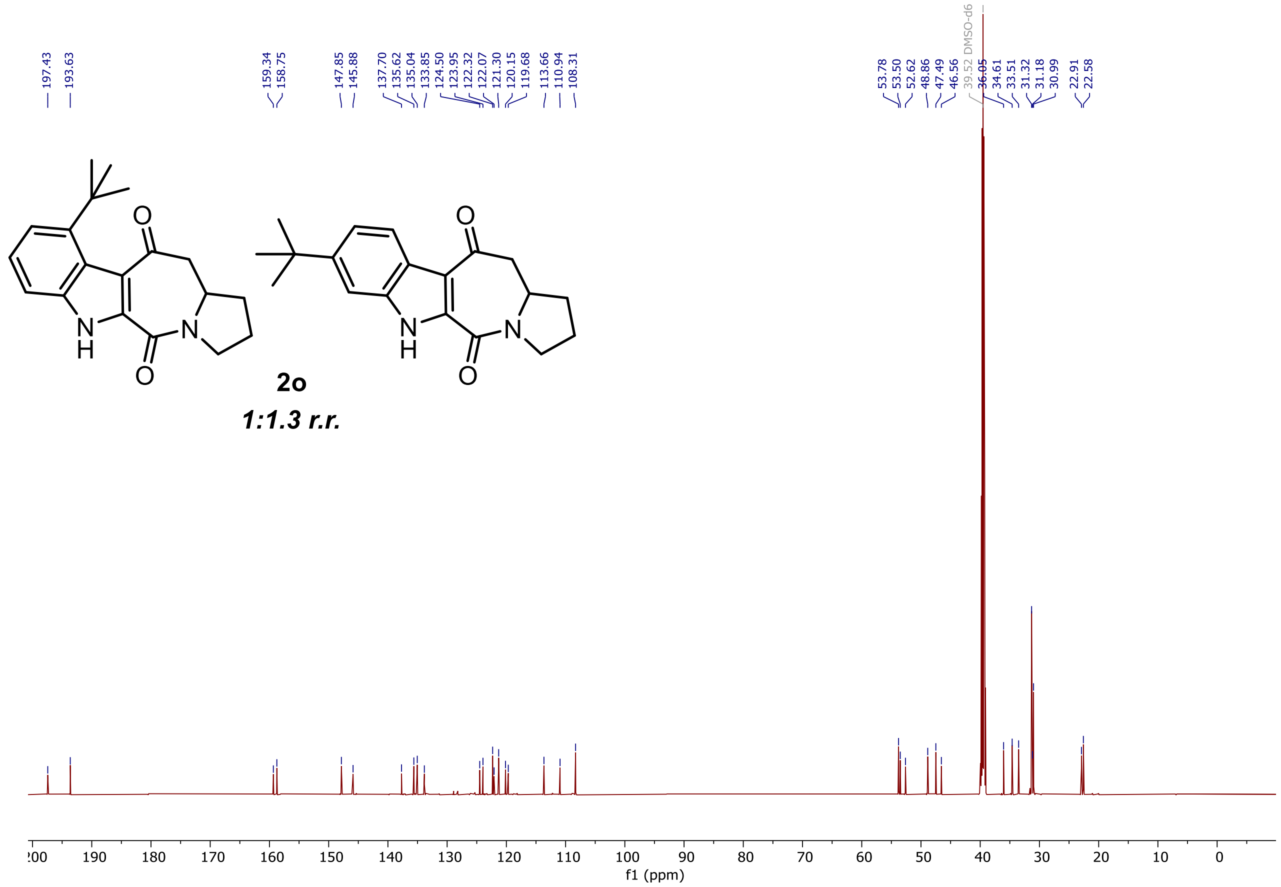

^{1}H NMR (400 MHz, DMSO-d_6) of **2p**

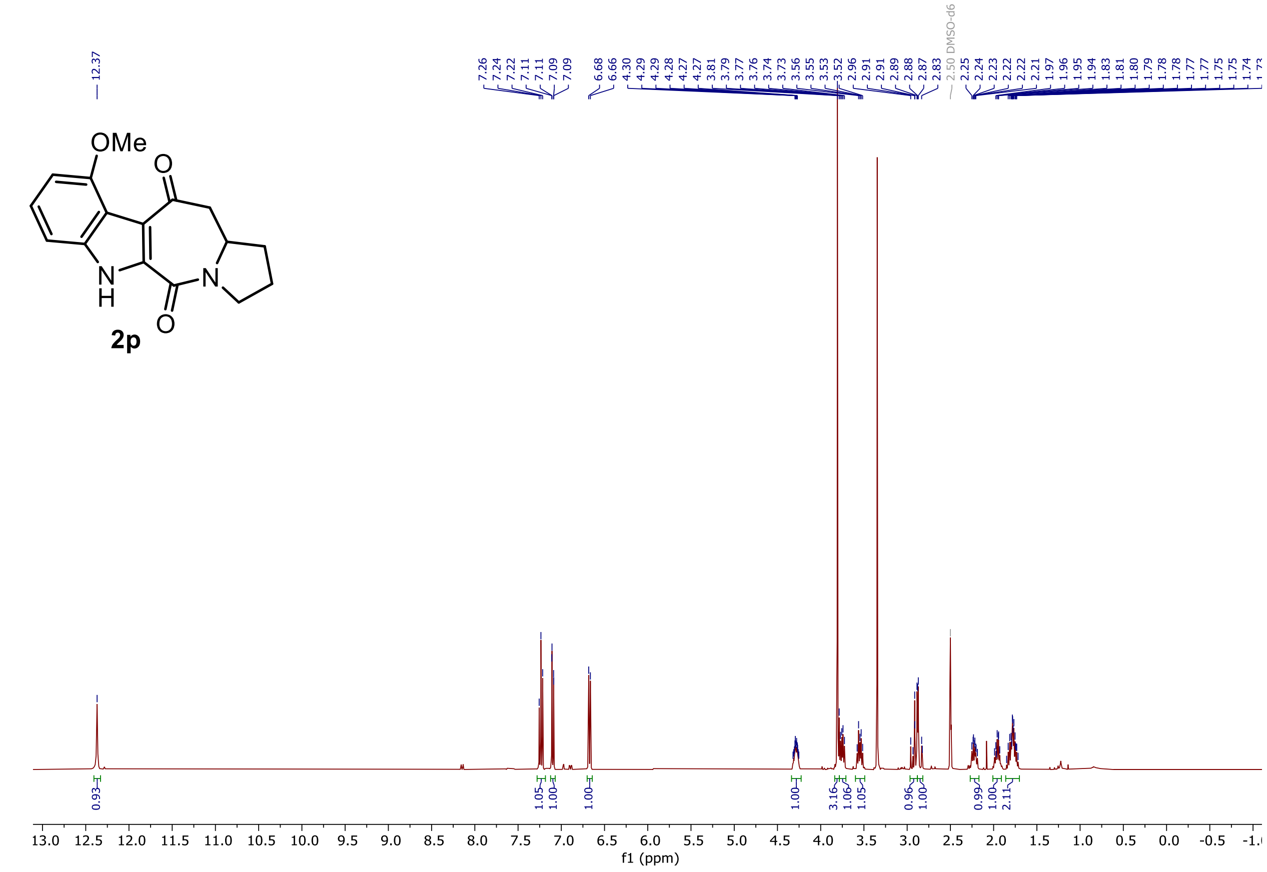

^{13}C NMR (151 MHz, DMSO-d_6) of **2p**

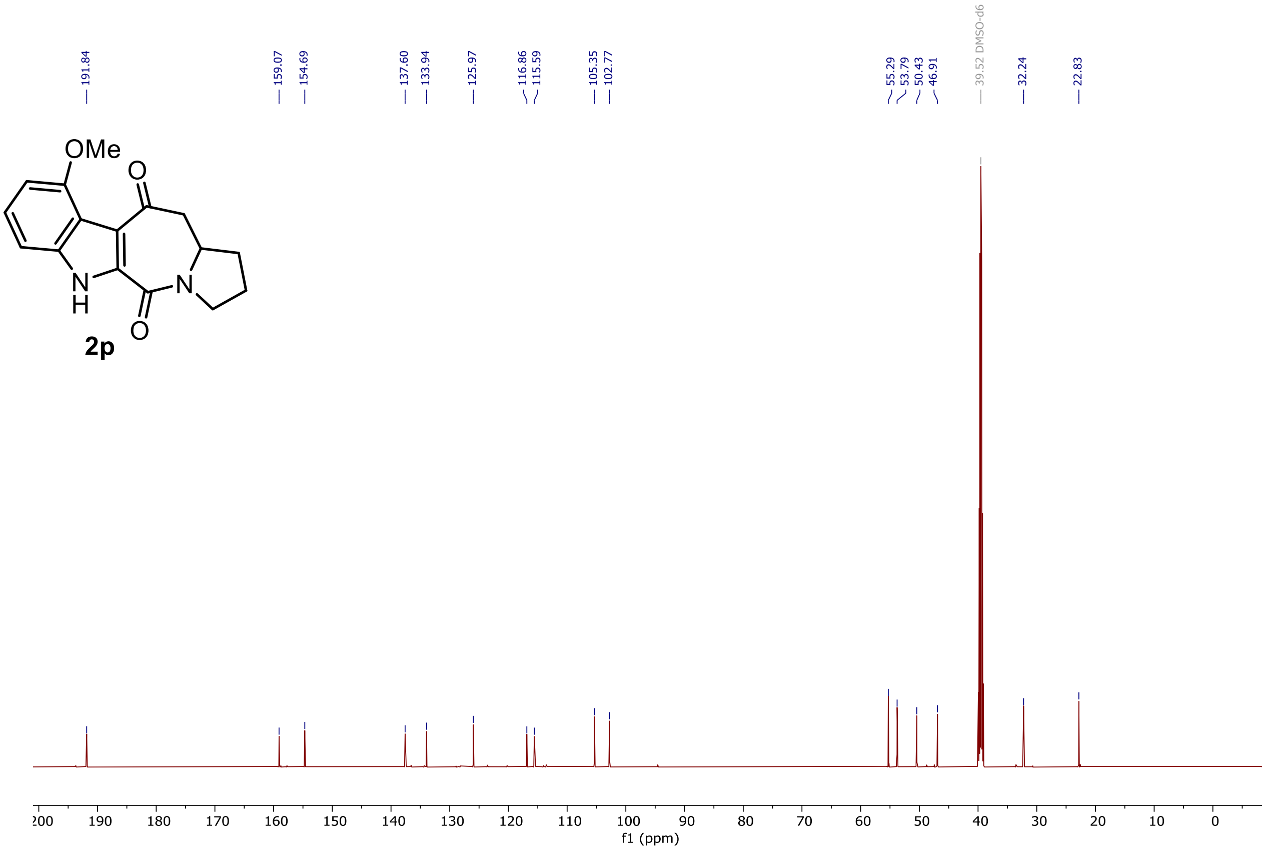

^1H NMR (400 MHz, DMSO-d_6) of **2p'**

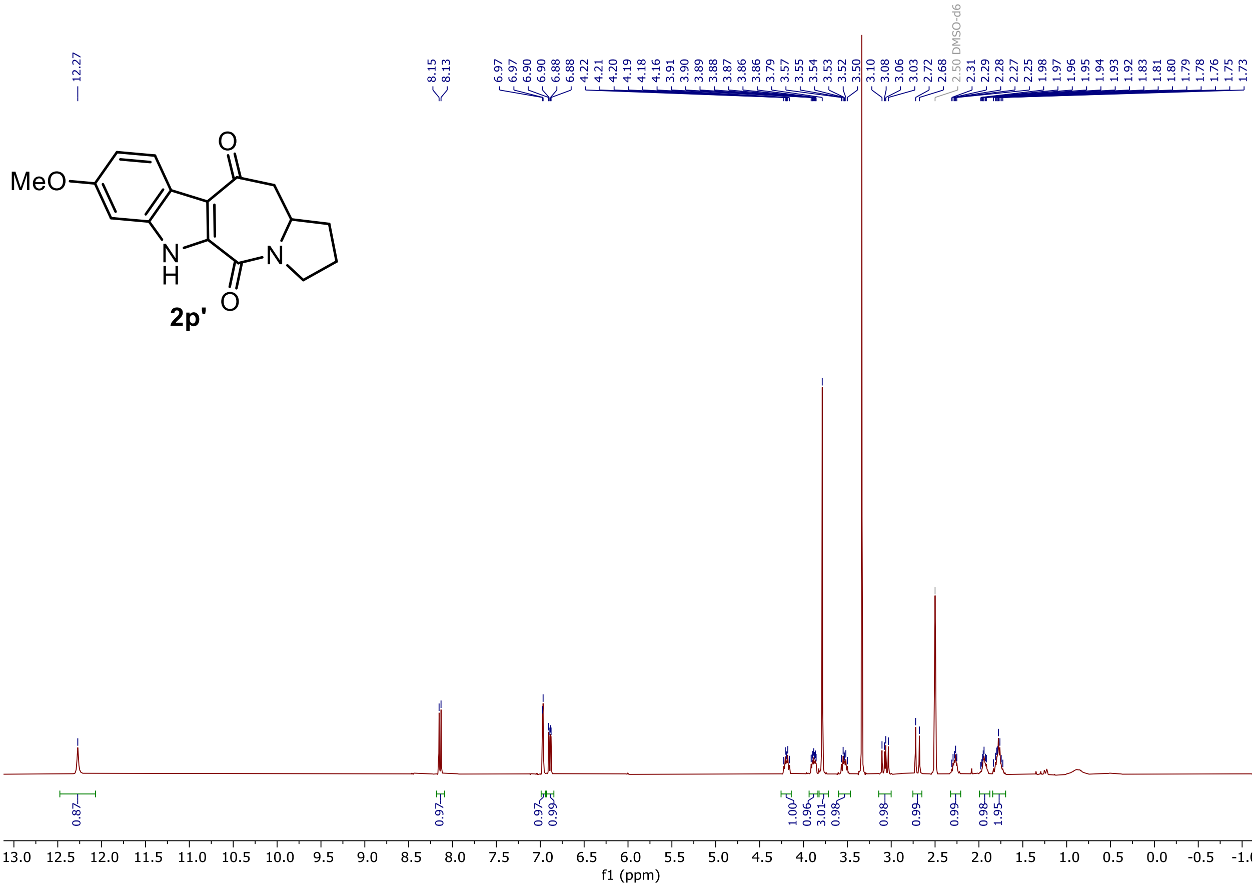

^{13}C NMR (151 MHz, DMSO-d_6) of **2p'**

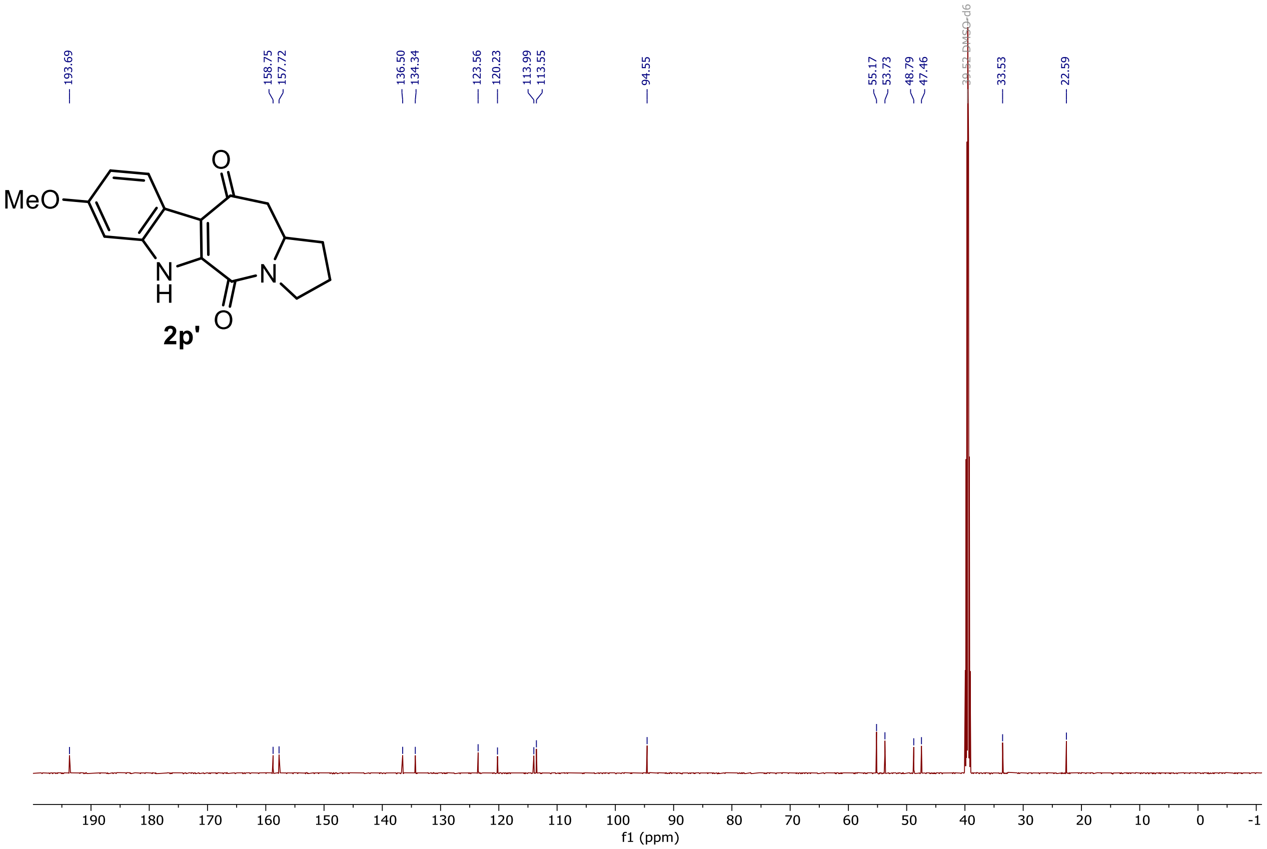

^1H NMR (400 MHz, DMSO-d_6) of **2q**

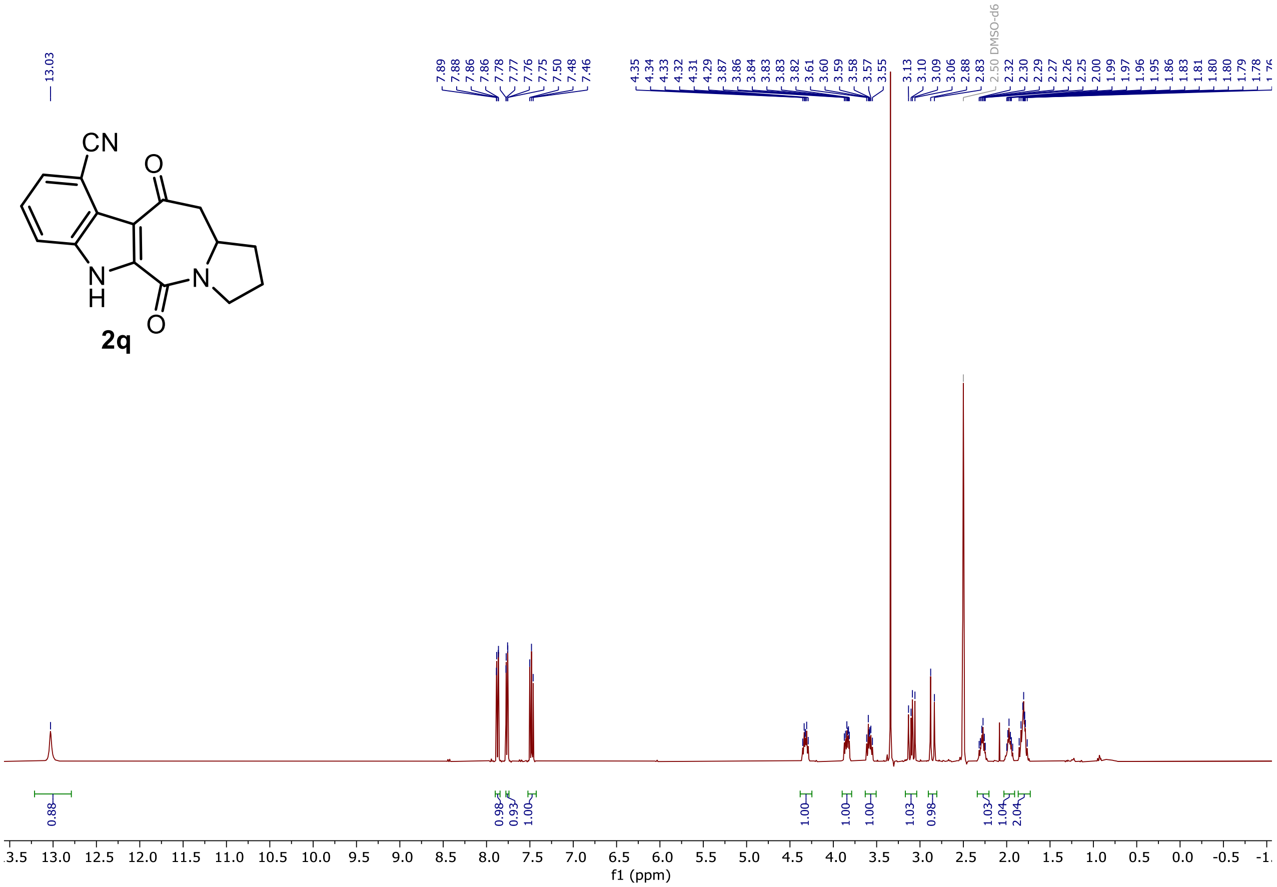

^{13}C NMR (126 MHz, DMSO-d_6) of **2q**

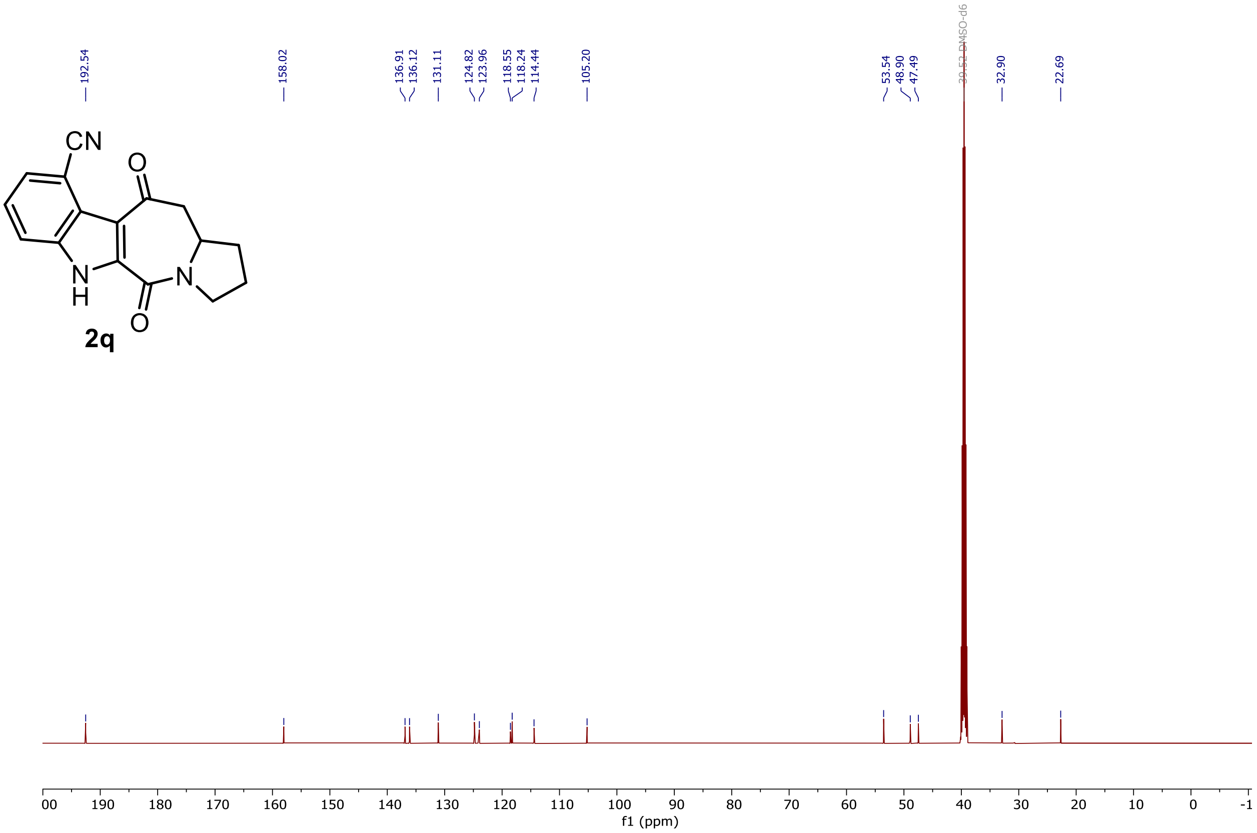

^{1}H NMR (400 MHz, DMSO-d_6) of **2q'**

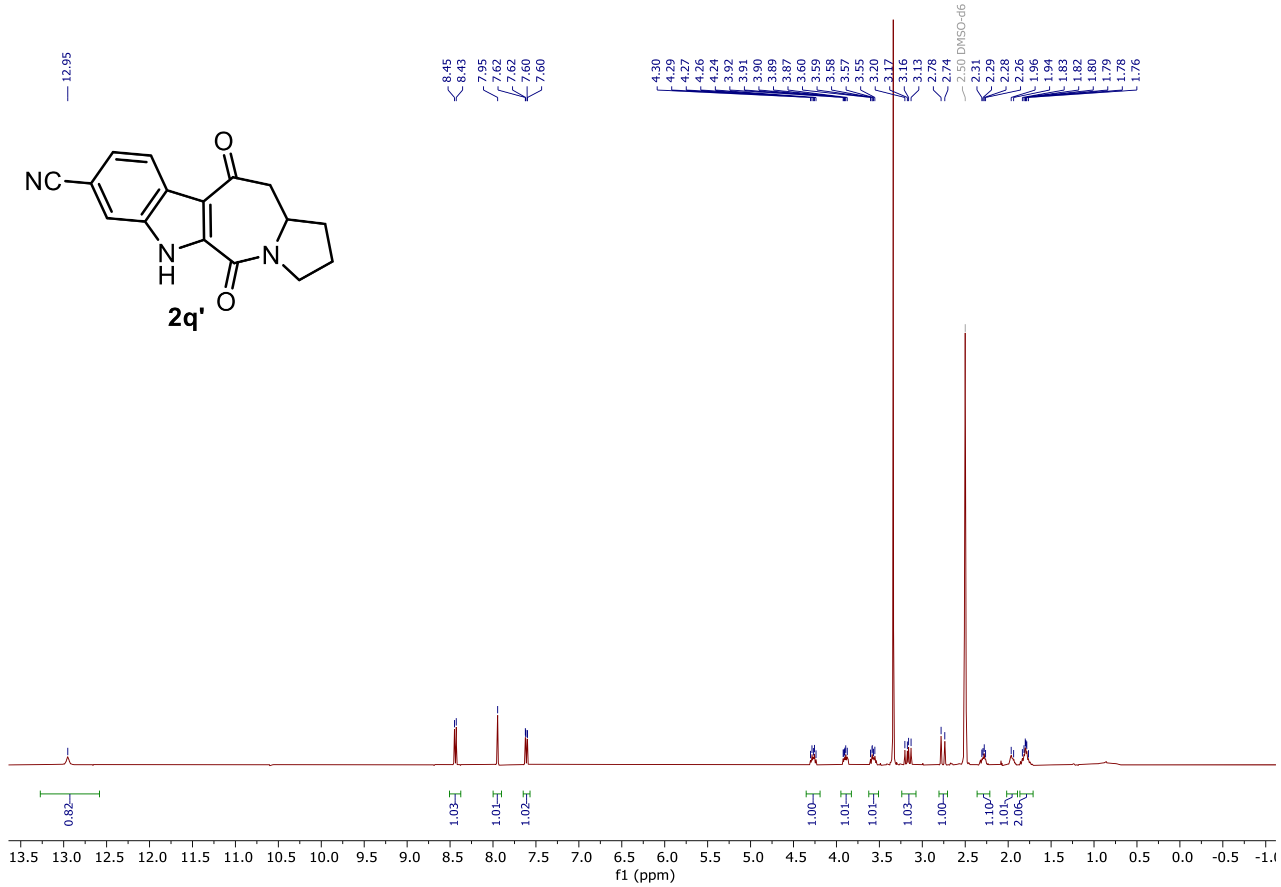

^{13}C NMR (151 MHz, DMSO-d_6) of **2q'**

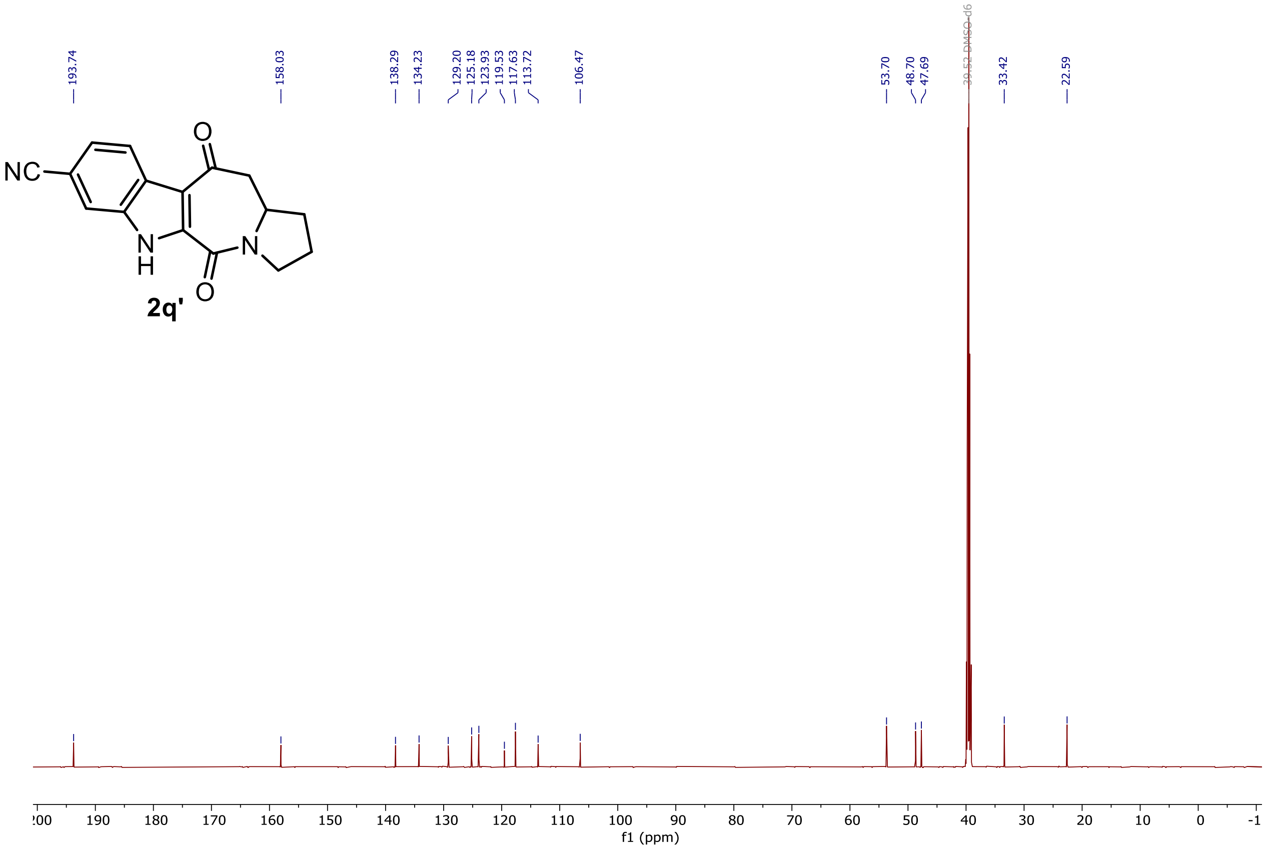

^{1}H NMR (400 MHz, DMSO-d_6) of **2r**

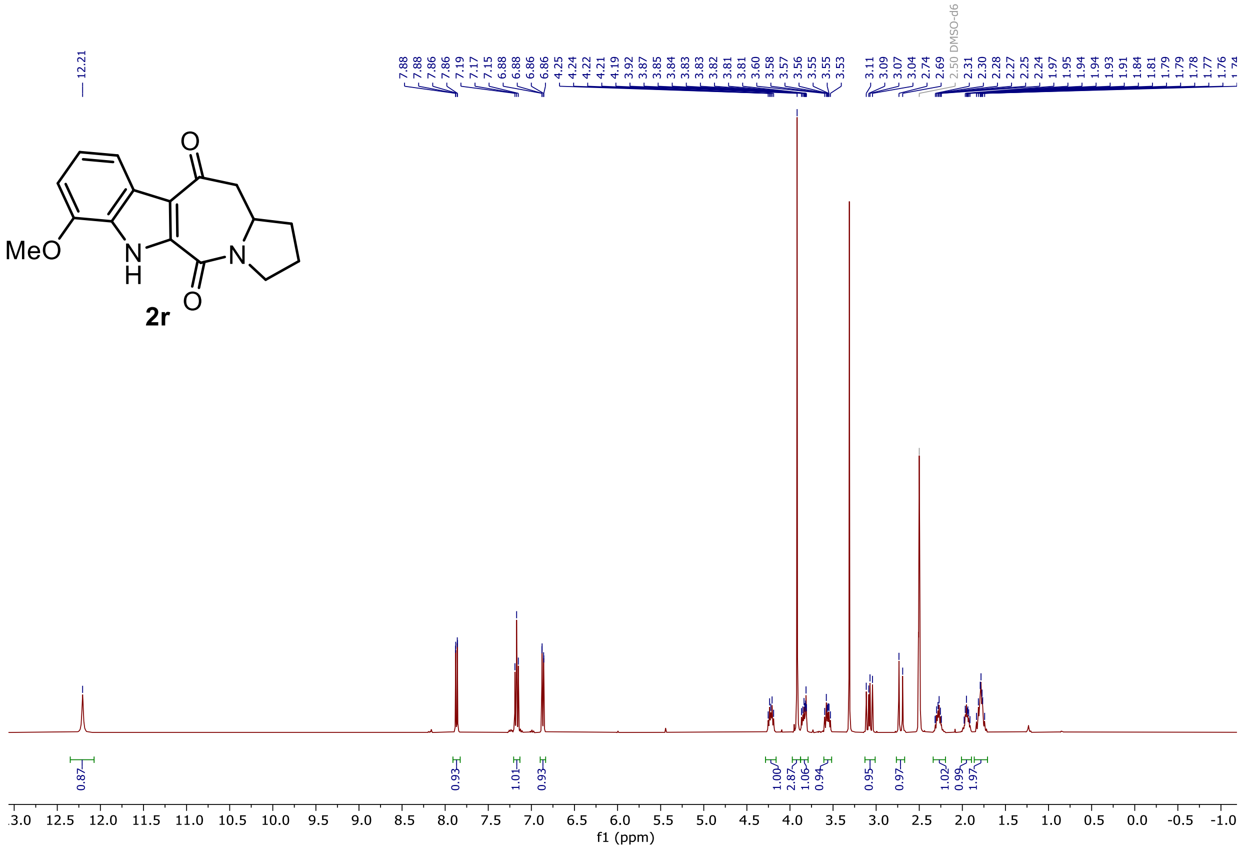

^{13}C NMR (126 MHz, DMSO-d_6) of **2r**

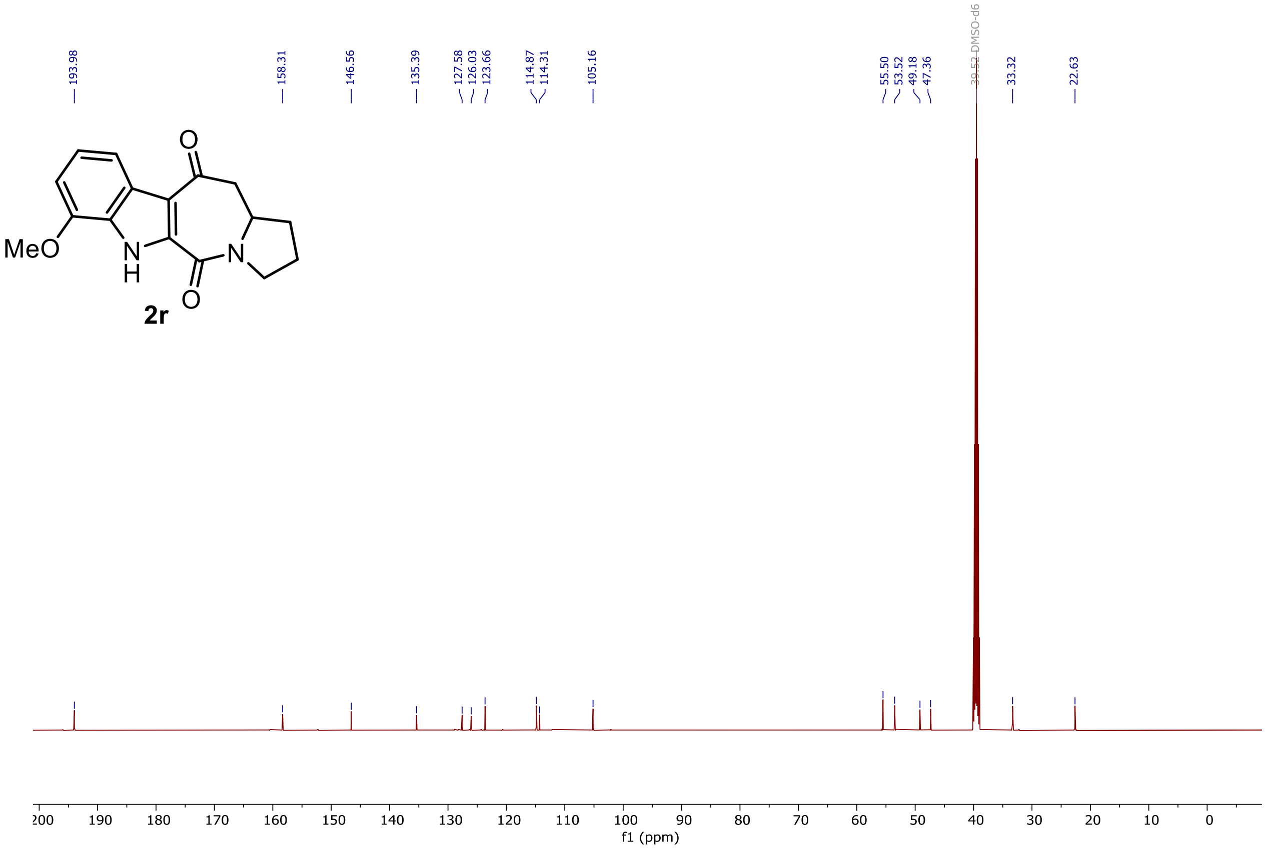

7. REFERENCES

(1) Elliott, L. D.; Knowles, J. P.; Koovits, P. J.; Maskill, K. G.; Ralph, M. J.; Lejeune, G.; Edwards, L. J.; Robinson, R. I.; Clemens, I. R.; Cox, B.; Pascoe, D. D.; Koch, G.; Eberle, M.; Berry, M. B.; Booker-Milburn, K. I., Batch Versus Flow Photochemistry: a Revealing Comparison of Yield and Productivity. *Chem. Eur. J.* **2014**, *20*, 15226.

(2) Booker-Milburn, K. I.; Anson, C. E.; Clissold, C.; Costin, N. J.; Dainty, R. F.; Murray, M.; Patel, D.; Sharpe, A., Intramolecular Photocycloaddition of N-Alkenyl Substituted Maleimides: A Potential Tool for the Rapid Construction of Perhydroazaazulene Alkaloids. *Eur. J. Org. Chem*. **2001**, 1473.

(3) Bruker, SAINT+ v8.38A Integration Engine, Data Reduction Software, Bruker Analytical X-ray Instruments Inc., Madison, WI, USA 2015.

(4) Bruker, SADABS 2014/5, Bruker AXS area detector scaling and absorption correction, Bruker Analytical X-ray Instruments Inc., Madison, Wisconsin, USA 2014/5.

(5) Sheldrick, G. M., A Short History of SHELX. *Acta Crystallographica Section A* **2008**, *64*, 112.

(6) Sheldrick, G. M., Crystal Structure Refinement with SHELXL. *Acta Crystallographica Section C-Structural Chemistry* **2015**, *71*, 3.

(7) Dolomanov, O. V.; Bourhis, L. J.; Gildea, R. J.; Howard, J. A. K.; Puschmann, H., OLEX2: a Complete Structure Solution, Refinement and Analysis Program. *J. Appl. Crystallogr.* **2009**, 42, 339.

(8) Quint, V.; Morlet-Savary, F.; Lohier, J.-F.; Lalevée, J.; Gaumont, A.-C.; Lakhdar, S. Metal-Free, Visible Light-Photocatalyzed Synthesis of Benzo[*b*]phosphole Oxides: Synthetic and Mechanistic Investigations. *J. Am. Chem. Soc.* **2016**, *138*, 7436.

(9) Frisch, M. J.; Trucks, G. W.; Schlegel, H. B.; Scuseria, G. E.; Robb, M. A.; Cheeseman, J. R.; Scalmani, G.; Barone, V.; Mennucci, B.; Petersson, G. A.; Nakatsuji, H.; Caricato, M.; Li, X.; Hratchian, H. P.; Izmaylov, A. F.; Bloino, J.; Zheng, G.; Sonnenberg, J. L.; Hada, M.; Ehara, M.; Toyota, K.; Fukuda, R.; Hasegawa, J.; Ishida, M.; Nakajima, T.; Honda, Y.; Kitao, O.; Nakai, H.; Vreven, T.; Montgomery, J. J. A.; Peralta, J. E.; Ogliaro, F.; Bearpark, M.; Heyd, J. J.; Brothers, E.; Kudin, K. N.; Staroverov, V. N.; Keith, T.; Kobayashi, R.; Normand, J.; Raghavachari, K.; Rendell, A.; Burant, J. C.; Iyengar, S. S.; Tomasi, J.; Cossi, M.; Rega, N.; Millam, J. M.; Klene, M.; Knox, J. E.; Cross, J. B.; Bakken, V.; Adamo, C.; Jaramillo, J.; Gomperts, R.; Stratmann, R. E.; Yazyev, O.; Austin, A. J.; Cammi, R.; Pomelli, C.; Ochterski, J. W.; Martin, R. L.; Morokuma, K.; Zakrzewski, V. G.; Voth, G. A.; Salvador, P.; Dannenberg, J. J.; Dapprich, S.; Daniels, A. D.; Farkas, O.; Foresman, J. B.; Ortiz, J. V.; Cioslowski, J.; Fox, D. J., Gaussian 09, Revision D.01, Gaussian, Inc., Wallingford CT, 2013.

(10) Elliott, Luke D.; Kayal, S.; George, M. W.; Booker-Milburn, K. Rational Design of Triplet Sensitizers for the Transfer of Excited State Photochemistry from UV to Visible. *J. Am. Chem. Soc.* **2020**, *142*, 14947.
